# Supplementary material for: Quantifying the carbon footprint of clinical trials: guidance development and case studies
Source: BMJ Open. 2024 Jan 24;14(1):e075755. doi: 10.1136/bmjopen-2023-075755 (PMC10823997; doi:10.1136/bmjopen-2023-075755)
Supplement: Supplementary data [file bmjopen-2023-075755supp001.pdf]

FUNDED BY NIHR | National Institute for Health and Care Research

LIVERPOOL CLINICAL TRIALS CENTRE LCTC

ICR

Enabling lower carbon clinical trials: Development and prototype testing of a method to quantify the carbon footprint of clinical trials to inform future lower carbon clinical trial design

Detailed Guidance and method to calculate the carbon footprint of a clinical trial

Background

Around 15 years ago, the Sustainable Trials Study Group concluded that clinical trials contribute substantially to greenhouse gas emissions, notably through energy use in research premises and air travel[1].

In addition, a study conducted in 2009 of 12 UK pragmatic randomised trials involving an average of 402 participants showed that the average carbon emission generated by the trials was 78.4 tonnes[2]. Multiplying this total by the 350,000 national and international trials registered on ClinicalTrials.gov, this would estimate that emissions attributable to all global clinical trials to be about 27·5 million tonnes of carbon dioxide equivalent[3].

Since then, the urgency of the threat from the climate crisis has increased exponentially and the World Health Organization calls climate change the single biggest health threat facing humanity[4]. Planned climate action is not sufficient to prevent the current warming predictions; humanity must limit warming to 1.5 degrees and reach net-zero by 2040 to avoid the worst consequences of climate change.

As a first step to reduce the environmental impact of clinical trials, a method to quantify the carbon footprint of a clinical trial and associated processes is required.

Introduction

This guidance provides information on how to carbon footprint a clinical trial for the purposes of the NIHR-funded project 'enabling lower carbon clinical trials.'

Within the guidance, clinical trial processes have been sub-divided into the following modules:

1. Trial set up
2. CTU emissions
3. Trial specific meetings and travel
4. Treatment intervention
5. Data collection and exchange
6. Trial Supplies and equipment
7. Trials specific patient assessments
8. Samples
9. Laboratory
10. Trial close out

The above list is not exhaustive, and it is expected that further activities and modules may need to be added to account for specialist processes in all clinical trial types and as knowledge around life cycle analysis increases.

NB: analysis of data does not need to be calculated separately, it is covered by the emissions attributed to trial staff FTE in "CTU emissions" and calculations included within "Data Collection and exchange".

Griffiths J, *et al. BMJ Open* 2024; 14:e075755. doi: 10.1136/bmjopen-2023-075755

FUNDED BY NIHR | National Institute for Health and Care Research

Calculating carbon footprint

A carbon footprint is a measure of greenhouse gases, usually quoted in kg or tonnes of carbon dioxide equivalent (CO_2e). To calculate the carbon footprint of a particular clinical trial process, both 'activity data' and 'emission factors' are required.

An 'activity' could be anything from electricity consumption to materials, travel and food. Activity data quantifies the amount of that activity e.g. distance travelled, kWh used etc.

An emission factor, also known as a conversion factor, "is a coefficient which allows you to convert the activity data into greenhouse gas emissions. It is the average emission rate of a given source, relative to units of activity or process/processes."[5]

To calculate a carbon footprint of a trial, the activity data will need to be provided by the trial management team and multiplied by the emission factors provided in this guidance document. Two types of activity data may be used:

- Primary data: data collected first-hand from specific activities within the studied clinical trial process i.e., the data collected where you can determine the amount of the activity taking place, for example electricity in kWh used by a building or the weight of an IMP shipment and the distance it travels.

- Secondary data: activity data that is not collected from specific activities within the studied clinical trial because you cannot determine or measure the exact quantity of the activity taking place, for example the number of m^2 occupied by an office worker or hospital worker. Secondary data may take the form of average, or typical, information about an activity from a published study or other source and will be provided in this guidance document e.g., average m^2 occupied by an office worker or average distance travelled.

Primary activity data are preferred for all activity data used in each module. However, secondary data may be used where primary activity data is unavailable or difficult to obtain.

It is important to avoid double-counting activities i.e., modules must not include activities already covered by other modules in the clinical trial process map. A data collection tool is provided alongside this guidance to aid in this process and help avoid double-counting.

Scope

This guidance describes a method to calculate the carbon footprint of a UK, academic clinical trial.

It can be applied to trials with international participation, however as emission factors vary between countries, and those provided within this guidance are UK-specific, country-specific emission factors may be required. Proxy emission factors which have been approved for use can be used where required and the source country of an emission factor will be stated where applicable.

The tool is intended to calculate the carbon footprint of the processes specific to the clinical trial, defined as the data required to analyse the trial endpoints and the research activities over and above standard of care.

The guidance is intended for use as a tool to inform sustainable decision-making in the design of clinical trials, rather than a tool to calculate the absolute footprint of a clinical trial or compare environmental performance of one trial over another.

Griffiths J, *et al. BMJ Open* 2024; 14:e075755. doi: 10.1136/bmjopen-2023-075755

FUNDED BY NIHR | National Institute for Health and Care Research

Limitations

There are a number of emission factors that can be used for a particular process and activity data can be calculated in a number of ways. Therefore, life cycle analysis produces variation in its results, dependent on choices made by the individual performing the calculations.

We have endeavoured to include an explanation or justification for the choice of emission factors used. In addition, the emission factors have been selected as the most applicable and up to date factors that are freely available for public use. It is important to note that more up to date factors, or forecasted emission factors, may be available, but they are subject to licensing requirements and are not publicly available. The source of all factors used is included for reference.

This guidance accounts only for the greenhouse gas emissions. It does not include other metrics that are also important to consider when evaluating sustainability and the potential trade-offs, for example water use, land use, waste and those relating to social and economic impacts.

Assumptions

- The eventual aim of this tool is to be used prospectively during the design phase of a trial, before trial funding is secured. However, the tool will not capture the carbon footprint associated with work conducted during this period i.e., prior to confirmation of funding award. The tool can also be used retrospectively on clinical trials which are complete.
- The tool only calculates emissions of processes which have been funded and defined within the trial protocol i.e., future planned work which has not yet been funded or that will be defined outside of the protocol are not included.
- The tool only calculates the emissions of patient participation where it exceeds or is additional to routine care, where it is required to establish an endpoint, the patient population or eligibility. Use of the trial SOECAT or the costing included in the initial funding application (if the trial predates use of SOECAT) is encouraged, to consistently define the investigations considered in addition to routine care and/or part of the research question.
- For all translational/optional/research samples and sub studies, the tool does not calculate emissions associated with analysis performed by central laboratories or collaborators, but does calculate emissions for activities defined in the protocol, such as collecting the sample or data from patients, initial processing at participating sites and shipment of samples or data to the site of subsequent analysis.
- This guidance will only appraise trials with UK based trial management (sites may be international but the trial must be overseen by a UK Clinical Trials Unit/Sponsor/ Research team)
- The carbon footprint associated with the manufacture of a trial intervention e.g. IMPs and Medical Devices is not included in this guidance.
- The tool does not calculate the carbon footprint of waste associated with a particular clinical trial. Concerning clinical trial consumables, activity data is based on the quantities purchased. The only exception to this is the destruction of unused IMP at participating sites, as this an activity specifically undertaken for a clinical trial which has an associated carbon footprint.
- The tool does not calculate the carbon footprint of hospital and laboratory staff commuting.
- Carbon emissions generated by ethics and regulatory approval bodies are not within the scope of this guidance and will not be calculated.

NB: more module specific assumptions can be found throughout the document where applicable.

Griffiths J, *et al. BMJ Open* 2024; 14:e075755. doi: 10.1136/bmjopen-2023-075755

FUNDED BY NIHR | National Institute for Health and Care Research

LIVERPOOL CLINICAL TRIALS CENTRE LCTC

ICR

1. Trial set up

This module includes the following activities:

1.1 Production of trial documentation to be sent to sites or participants
1.2 Provision/postage of trial documentation to sites
1.3 Provision/postage of documentation to participants by CTU or participating sites
1.4 Provision/postage of incentives to participant

1.1. Production of trial documentation

For production of trial documentation, the carbon footprint of both the printing and materials must be calculated.

Printing: The number of pages must be multiplied by 0.005 to produce a weight in kilograms, and the weight multiplied by the relevant emission factor provided below.

- Black and white: kg (paper) x 0.22438 = kgCO_2e
- Colour: kg (paper) x 0.31786 = kgCO_2e

1292	use, printer, laser jet, b/w, per kg printed paper	electronics	services	kg	RER	0.22438
1293	use, printer, laser jet, b/w, printing per h	electronics	services	h	CH	0.37213
1294	use, printer, laser jet, b/w, printing per h	electronics	services	h	RER	0.39065
1295	use, printer, laser jet, colour, per kg printed paper	electronics	services	kg	CH	0.2027
1296	use, printer, laser jet, colour, per kg printed paper	electronics	services	kg	RER	0.31786

NB**:** If you are unable to calculate the number of pages, you may assume that there are 150 pages in a small ring binder and 500 pages in a large lever arch folder.

Assumption: 1 piece of paper weighs around 5g/0.005kg

Emission factor source: Ecoinvent, version 2.2, 2011 [6] (RER = European emission factor)

Materials (paper): For the carbon footprint of paper production**,** the number of pages must be multiplied by 0.005 to produce a weight, and the weight multiplied by the emission factor for paper manufacture provided below.

Kg (paper) x 0.919 = kg CO_2e

Paper and board: paper	Gov.UK	2021	kg	0.919

Emission factor source: Greenhouse gas reporting: conversion factors 2021, GOV.UK [7]

Materials (folders): As trial documentation is often sent to sites in folders, provided below are estimated weights of folders. The total weight in kg will then need be multiplied by the emission factor for cardboard (provided below).

53	Paper and board: board	Gov.UK	2021	kg	0.821

CF of the folder materials = Kg x 0.821 = kg CO_2e

Lever arch: Assumption: Weight of lever arch = 0.5kg

Ring binder: Assumption: Weight of ring binder = 0.3kg Eastlight A4 Black Ring Binder - EA54121 (staples.co.uk)

Emission factor source: Greenhouse gas reporting: conversion factors 2021, GOV.UK [7]

FUNDED BY NIHR | National Institute for Health and Care Research

LIVERPOOL CLINICAL TRIALS CENTRE LCTC

ICR

1.2, 1.3, 1.4 Provision of trial materials

For provision of trial materials to sites by post/courier you will need to calculate the total weight of the materials (in tonnes) and multiply this by the distance they travel (in km) to get tonne.km. The t.km is then multiplied by the emission factor for either road or air freight provided below.

Road freight:

Mass (tonnes) x distance (km) = t.km

t.km x emission factor = (kg CO_2e)

Emission factor for road freight = 0.19443

1942	2724	transport, lorry 20-28t, fleet average	transport systems	road	tkm	CH	0.19443

Emission factor source: Ecoinvent, version 2.2, 2011 [6]

Air freight:

Mass (tonnes) x distance (km) = t.km

t.km x most suitable emission factor from highlighted column = (kg CO_2e)

			With RF				Without RF			
Activity	Type	Unit	kg CO_2e	kg CO_2	kg CH_4	kg N_2O	kg CO_2e	kg CO_2	kg CH_4	kg N_2O
Freight flights	Domestic, to/from UK	tonne.km	4.49363	4.46949	0.00188	0.02226	2.3765	2.35236	0.00188	0.02226
	Short-haul, to/from UK	tonne.km	2.30229	2.2908	0.00008	0.01141	1.21717	1.20568	0.00008	0.01141
	Long-haul, to/from UK	tonne.km	1.0189	1.01381	0.00004	0.00505	0.53867	0.53358	0.00004	0.00505
	International, to/from non-UK	tonne.km	1.0189	1.01381	0.00004	0.00505	0.53867	0.53358	0.00004	0.00505

NB: 'With RF' values are recommended. RF (combustion and radiative forcing) includes the indirect and direct emissions.

NB: Always use values relating to 'kgCO_2e'

For air freight you need to add the WTT (well-to-tank) value to get the final total for freight flight. WTT can be calculated by multiplying the t.km used in the first calculation by the correlating WTT conversion factor provided below.

Tonne.km x correlating WTT conversion factor

			With RF	Without RF
Activity	Type	Unit	kg CO_2e	kg CO_2e
WTT- freight flights	Domestic, to/from UK	tonne.km	0.49186	0.49186
	Short-haul, to/from UK	tonne.km	0.25210	0.25210
	Long-haul, to/from UK	tonne.km	0.11157	0.11157
	International, to/from non-UK	tonne.km	0.11157	0.11157

Emission factor source: Greenhouse gas reporting: conversion factors 2021, GOV.UK [7]

Assumption: If unknown, for delivery of trial supplies to patients or GP, use 17.4km as distance from hospital to patient, or hospital to GP. Source: BMJ 2009;339:b4187 [2]

Griffiths J, *et al. BMJ Open* 2024; 14:e075755. doi: 10.1136/bmjopen-2023-075755

FUNDED BY NIHR | National Institute for Health and Care Research

LIVERPOOL CLINICAL TRIALS CENTRE LCTC

ICR

2. CTU Emissions

This module includes the following activities:

2.1. Energy consumption at CTU
2.2. Heating
2.3. Trial team commuting

2.1. Energy consumption at CTU (according to staff FTE)

Often, the carbon footprint associated with energy consumption by the CTU cannot be calculated directly because the space or building will be used for other trials not being appraised and non-trial activities. Emissions are therefore estimated based on average per person emissions and the FTE of the trial employees.

According to the UK Employment Destiny Guide, public sector office space is 12 m^2 per FTE. This is multiplied by 59 kWh/m^2, the median electricity intensity for offices, to produce a per person per year usage. The emissions attributed to CTU energy consumption can then be calculated by multiplying the per person usage by the electricity emission factor provided. The calculation is exemplified below.

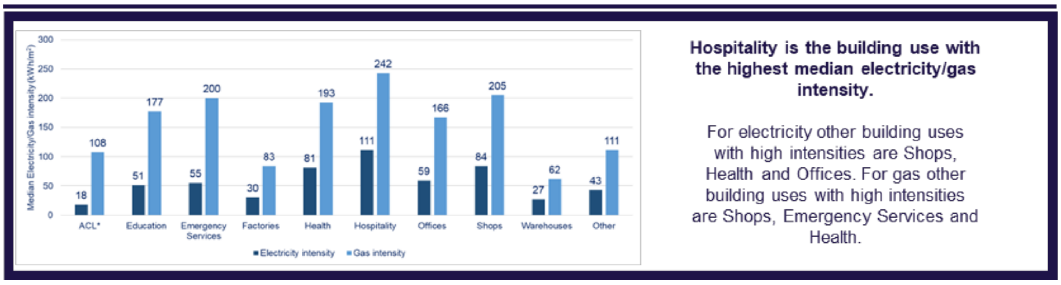

Calculation

$12m^2$ x 59 kWh/m^2 = 708 kWh per FTE per year

Electricity emission factor = 0.273

Name	Source	Year	Text	Unit	kg CO_2e	kg CO_2e	kg CO_2e	kg CO_2e	kg CO_2e/refrence flow unit
Electricity	Gov.UK	2021		kWh		0.21233	0.00489	0.05529	0.273

708 kWh x 0.273 = 193.3 kgCO_2e per FTE per year

Finally, multiply 193.3 kgCO_2e by the number of years and FTE applicable.

Assumption: According to the UK EMPLOYMENT DENSITY GUIDE, 3rd edition November 2015, office space per FTE is 12 m^2 (public sector) [8]

Office benchmark data source: The Non-Domestic National Energy Efficiency Data-Framework 2022 (England and Wales) (publishing.service.gov.uk)[9]

Electricity emission factor source: Greenhouse gas reporting: conversion factors 2021, GOV.UK [7]

Griffiths J, et al. BMJ Open 2024; 14:e075755. doi: 10.1136/bmjopen-2023-075755

FUNDED BY NIHR | National Institute for Health and Care Research

LIVERPOOL CLINICAL TRIALS CENTRE LCTC

ICR

2.2 Heating

For heating, the calculation follows the same method as above. The 12m^2 per person is multiplied by the office heating benchmark and then by the natural gas conversion factor provided below.

Office building heating benchmark: **167.27** (kWh/m^2)

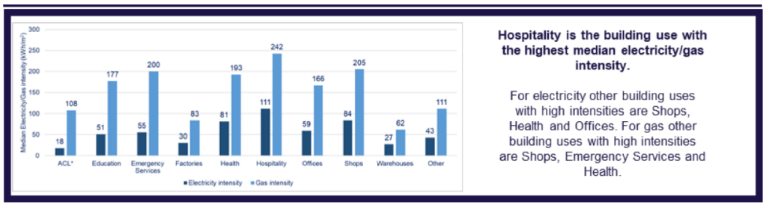

Natural gas conversion factor: **0.215**

Activity	Referen	EF	Comment	Refrence flow	Scope 1/Direc	Scope 2	Scope 3 T&D	Scope 3 WTT	Total Carbon intensity
Name	Source	Year	Text	Unit	kg CO_2e	kg CO_2e	kg CO_2e	kg CO_2e	kg CO_2e/refrence flow unit
Electricity	Gov.UK	2021		kWh		0.21233	0.00489	0.05529	0.273
Burning (hetaing) oil	Gov.UK	2021		litres	2.54014			0.52807	3.068
Natural gas	Gov.UK	2021		kWh (Gross CV)	0.18316			0.03135	0.215
LPG	Gov.UK	2021		litres	1.55709			0.18383	1.741

Calculation

- 12 m^2 x 167.27 kWh/m^2 = 2007.24 kWh per FTE per year
- 2007.24 kWh x 0.215 = 431.56 kg CO_2e per FTE per year

Multiply 431.56 kg CO_2e by the number of years and FTE applicable

Assumption: If the heating source is unknown, assume that the heating source is natural gas.

Assumption: According to the UK EMPLOYMENT DENSITY GUIDE, 3rd edition November 2015, office space per FTE is 12 m^2 public sector. [8]

Office benchmark data source: The Non-Domestic National Energy Efficiency Data-Framework 2022 (England and Wales) (publishing.service.gov.uk) [9]

Natural gas emission factor source: Greenhouse gas reporting: conversion factors 2021, GOV.UK [7]

Homeworking

Homeworking (office equipment + heating)	per FTE Working Hour	0.34075

Multiply the total number of FTE working hours by the conversion factor (0.34075) to calculate kgCO_2e (includes electricity use from office equipment and heating).

Emission factor source: Greenhouse gas reporting: conversion factors 2022, GOV.UK [10]

FUNDED BY NIHR | National Institute for Health and Care Research

LIVERPOOL CLINICAL TRIALS CENTRE LCTC

ICR

2.3. Trial team commuting

The emissions attributed to the trial team commuting is calculated using average commuting statistics. Using these statistics, the distance travelled by each mode of transport was calculated and multiplied by the relevant emission factor.

The average distance of a commuting journey was 8.8 miles (14km) in 2013/14. Multiplied by 220 commuting days, this results in a total distance of 6160km travelled per year.

Car (used for 68% of the 6160km distance) – 68% of 6160km = 4188.8km

4188.8 x 0.223 = 934.1 kgCO_2e

Large car petrol	Gov.UK	2021	km	0.27909	0.07833	0.357
Average car petrol	Gov.UK	2021	km	0.17431	0.04885	0.223
[illegible]	[illegible]	[illegible]	[illegible]	[illegible]	[illegible]	[illegible]

Rail (used for 10% of the 6160km distance) = 616 km x 0.043 = 26.5 kgCO_2e

Coach	Gov.UK	2021	passenger.km	0.02684	0.00646	0.033
National rail	Gov.UK	2021	passenger.km	0.03549	0.00733	0.043
International rail	Gov.UK	2021	passenger.km	0.00446	0.00074	0.005
[illegible]	[illegible]	[illegible]	[illegible]	[illegible]	[illegible]	[illegible]

Bus (used for 6% of the 6160km distance): 369.6km x 0.127 = 46.9 kgCO_2e

11	Local bus (not London)	Gov.UK	2021	passenger.km	0.11774	0.02868	0.146
12	Local London bus	Gov.UK	2021	passenger.km	0.07718	0.01885	0.096
13	Average local bus	Gov.UK	2021	passenger.km	0.10227	0.02494	0.127
14	Coach	Gov.UK	2021	passenger.km	0.02684	0.00646	0.033
15	[illegible]	[illegible]	[illegible]	[illegible]	[illegible]	[illegible]	[illegible]

Other (used for 5% of the 6160km distance): Assume 3% cycling so zero emissions, 1% motorbike, 1% taxi

Motorbike: 61.6km x 0.145 = 8.9kgCO_2e

London Underground	Gov.UK	2021	passenger.km	0.02781	0.00461	0.032
Average motorbike	Gov.UK	2021	km	0.11355	0.03134	0.145
Small car diesel	Gov.UK	2021	km	0.13758	0.03344	0.171
Medium car diiesel	Gov.UK	2021	km	0.16496	0.04018	0.205

Taxi: 61.6 x 0.185 = 11.4

[illegible]	[illegible]	[illegible]	[illegible]	[illegible]	[illegible]	[illegible]	[illegible]
8	LPG	Gov.UK	2021	litres	1.55709	0.18383	1.741
9	Regular taxi	Gov.UK	2021	passenger.km	0.14876	0.03632	0.185
10	Black cab	Gov.UK	2021	passenger.km	0.20416	0.05	0.254
11	[illegible]	[illegible]	[illegible]	[illegible]	[illegible]	[illegible]	[illegible]

For 1 FTE, emissions attributed to commuting for 1 year= 1027.8 kgCO_2e

Multiply by the number of years and FTE applicable

NB: The WTT (well-to-tank) has been included in the emission factors for all modes of transport and therefore does not need to be added manually in this case.

Assumptions:

- 220 days spent commuting. This was calculated by subtracting 10 days sick and 30 days paid leave from the 260 workdays in a year.
- 6160 km is the total distance an employee will commute per year. This was calculated by multiplying 28km (14km per commuting journey) by 220 days.

Sources:

- Transport Statistics Great Britain: 2021 - GOV.UK (www.gov.uk) [11]
- Commuting trends in England 1988 - 2015 (publishing.service.gov.uk) [12]
- Emission factor source: GOV.UK Greenhouse gas reporting, conversion factors 2021 [7]

FUNDED BY NIHR | National Institute for Health and Care Research

LIVERPOOL CLINICAL TRIALS CENTRE LCTC

ICR

3. Trial specific meetings and travel

This module includes the following activities:

3.1. Visits and travel to site
3.2. Travel to meetings
3.3. Hotel stays
3.4. Sustenance

3.1, 3.2. Visits and travel to site, travel to meetings

For business travel by bus, taxi and rail, the activity data is captured in passenger km (p.km). The number of passengers is multiplied by the distance travelled (km), then by the relevant emission factor.

Number of passengers x total distance (km) = p.km

p.km x suitable emission factor = (kg CO_2e)

Rail

Activity	Type	Unit	kg CO_2e	kg CO_2	kg CH_4	kg N_2O
Rail	National rail	passenger.km	0.03549	0.0351	0.00007	0.00032
	International rail	passenger.km	0.00446	0.00441	0.00002	0.00003
	Light rail and tram	passenger.km	0.02861	0.02832	0.00011	0.00018
	London Underground	passenger.km	0.02781	0.02753	0.0001	0.00018

For business travel you need to **add** the WTT (well-to-tank) value to get the final total. WTT can be calculated by multiplying the p.km used in the first calculation by the correlating WTT conversion factor provided below.

passenger.km x correlating WTT conversion factor

Activity	Type	Unit	kg CO_2e
WTT- rail	National rail	passenger.km	0.00892
	International rail	passenger.km	0.00116
	Light rail and tram	passenger.km	0.00745
	London Underground	passenger.km	0.00724

NB: Always use values relating to 'kgCO_2e'
NB: Distances can be calculated using google maps

Assumption: Distances calculated from CTU to destination

Emission factor source: Greenhouse gas reporting: conversion factors 2021, GOV.UK [7]

Emission factors for all modes can be found in the orange 'business travel' tabs of [conversion-factors-2021-full-set-advanced-users.xlsm (live.com)]

Griffiths J, *et al. BMJ Open* 2024; 14:e075755. doi: 10.1136/bmjopen-2023-075755

FUNDED BY NIHR | National Institute for Health and Care Research — LIVERPOOL CLINICAL TRIALS CENTRE LCTC — ICR

3.1, 3.2. Visits and travel to site, travel to meetings

For business travel by air, activity data is also captured in passenger km (p.km). The number of passengers is multiplied by the distance travelled (km), then by the relevant emission factor.

Number of passengers x total distance (km) = p.km

p.km x suitable emission factor = (kg CO_2e)

An emission factor must be chosen from the following categories:

- Domestic
- Short Haul International (≤3700 km) – Average/Economy/Business
- Long Haul International (>3700 km) – Average/Economy/Business
- International (travel between non-UK countries)

Activity	Haul	Class	Unit	kg CO_2e
Flights	Domestic, to/from UK	Average passenger	passenger.km	0.24587
	Short-haul, to/from UK	Average passenger	passenger.km	0.15353
		Economy class	passenger.km	0.15102
		Business class	passenger.km	0.22652
	Long-haul, to/from UK	Average passenger	passenger.km	0.19309
		Economy class	passenger.km	0.14787
		Premium economy class	passenger.km	0.23659
		Business class	passenger.km	0.42882
		First class	passenger.km	0.59147
	International, to/from non-UK	Average passenger	passenger.km	0.18362
		Economy class	passenger.km	0.140625
		Premium economy class	passenger.km	0.225
		Business class	passenger.km	0.40781
		First class	passenger.km	0.56251

For business travel you need to add the WTT (well-to-tank) value to get the final total. WTT can be calculated by multiplying the p.km used in the first calculation by the correlating WTT conversion factor provided below.

passenger.km x correlating WTT conversion factor

				With RF	Without RF
Activity	Haul	Class	Unit	kg CO_2e	kg CO_2e
WTT- flights	Domestic, to/from UK	Average passenger	passenger.km	0.02691	0.02691
	Short-haul, to/from UK	Average passenger	passenger.km	0.01681	0.01681
		Economy class	passenger.km	0.01654	0.01654
		Business class	passenger.km	0.0248	0.0248
	Long-haul, to/from UK	Average passenger	passenger.km	0.02114	0.02114
		Economy class	passenger.km	0.01619	0.01619
		Premium economy class	passenger.km	0.02591	0.02591
		Business class	passenger.km	0.04696	0.04696
		First class	passenger.km	0.06477	0.06477
	International, to/from non-UK	Average passenger	passenger.km	0.02011	0.02011
		Economy class	passenger.km	0.0154	0.0154
		Premium economy class	passenger.km	0.02464	0.02464
		Business class	passenger.km	0.04466	0.04466
		First class	passenger.km	0.06159	0.06159

NB: Always use values relating to 'kgCO_2e'
NB: Distances can be calculated using google maps
NB: 'With RF' values are recommended. RF (combustion and radiative forcing) includes the indirect and direct emissions

Assumption: travellers departed from the nearest airport to their place of work and flew directly to the airport of the city to which they were travelling.
Emission factor source: Greenhouse gas reporting: conversion factors 2021, GOV.UK [7]

Emission factors for all modes can be found in the orange 'business travel' tabs of [conversion-factors-2021-full-set-advanced-users.xlsm (live.com)]

FUNDED BY NIHR | National Institute for Health and Care Research

LIVERPOOL CLINICAL TRIALS CENTRE LCTC

ICR

3.2 Travel to meetings

Teleconferencing: for meetings which are conducted by teleconferencing, multiply the number of people and hours by the figure provided below.

A standard videoconferencing service has a carbon footprint of 157 g CO_2e/hr. When the video is switched off, videoconferencing uses 6.9 g CO_2e/hr.

Emission factor source: Turn off that camera during virtual meetings, environmental study says - Purdue University News [13]

3.3 Hotel Stays

To calculate the emissions attributed to hotel stays, the number of hotel rooms is multiplied by the length of stay (in number of nights) and by the conversion factor for the appropriate country to give the associated emissions.

Each country has a different emission factor. The emission factor for the UK is shown below, other countries can be found at conversion-factors-2021-full-set-advanced-users.xlsm (live.com)

	Country	Unit	kg CO_2e
Hotel stay	UK	Room per night	13.9
	UK (London)	Room per night	13.8

NB: A ‘room per night’ accounts for use of the room and does not differentiate for number of travellers staying in the room.

Source: Greenhouse gas reporting: conversion factors 2021, GOV.UK [7]

Alternatively, you may use a cost-based method by multiplying the total cost in the funding application by the emission factor below:

Cost (£) x 0.358 = kg CO_2e

Wholesale distribution	Gov.UK	2020	converted fron	£	0.375
Retail distribution	Gov.UK	2020	converted fron	£	0.277
Hotels, catering, pubs e	Gov.UK	2020	converted fron	£	0.358
Railway transport[5]	Gov.UK	2020	converted fron	£	0.678
Road transport[5]	Gov.UK	2020	converted fron	£	0.690
Water transport[5]	Gov.UK	2020	converted fron	£	1.428
Air transport[5]	Gov.UK	2020	converted fron	£	2.089

Emission factor source: Gov.UK Government conversion factors for company reporting of greenhouse gas emissions 2012 - Annex 13 with consideration of 2020 inflation rates. [14]

Griffiths J, *et al. BMJ Open* 2024; 14:e075755. doi: 10.1136/bmjopen-2023-075755

FUNDED BY NIHR | National Institute for Health and Care Research

LIVERPOOL CLINICAL TRIALS CENTRE LCTC

ICR

3.4. Sustenance

For the carbon footprint associated with meeting lunches or hotel dinners, multiply the quantity by the relevant emission factor provided below.

Meeting lunches or hotel dinners (vegetarian) = 2.6 kgCO_2e per meal per person

Meeting lunches or hotel dinners (meat) = 5.92 kgCO_2e per meal per person

Emission factor source = WWF, 2018 Food_in_a_warming_world_report.PDF [15]

Griffiths J, *et al. BMJ Open* 2024; 14:e075755. doi: 10.1136/bmjopen-2023-075755

4. Intervention

This module includes guidance on the following different types of intervention. Pick the most applicable intervention type from:

4.1 Physical (IMP)
4.2 Clinical (Non-IMP)
4.3 Other (not captured above)

4.1 Physical (an IMP)

4.1.1 Movement of IMP from manufacturing site to distribution/packaging site

4.1.2 Movement of IMP from distribution/packaging site to participating sites or direct to participant

4.1.3 Materials required for the packaging and shipment of IMP

4.1.4 Destruction of overage

Assumptions: Calculations do not include manufacture of IMP.

4.2 Clinical (non-IMP)

NB: not all calculations will be relevant to all interventions.

4.2.1 Movement (shipment) of the intervention, or resources required to deliver the intervention
4.2.2 Materials required for the shipment of the intervention
4.2.3 Utilities required for delivery of the intervention
4.2.4 Activities or resources required/relating to delivery of the intervention

Assumptions: Calculations do not include manufacture of device/machinery/equipment delivering the intervention.

4.3 Other (Not captured above)

NB: not all calculations will be relevant to all interventions, depending on the specific intervention.

4.3.1 Movement of the intervention to the participant or participating site
4.3.2 Materials required for packaging and shipment of the intervention
4.3.3 Materials or resources required for delivery of the intervention
4.3.4 Travel required to facilitate delivery of the intervention

Griffiths J, *et al. BMJ Open* 2024; 14:e075755. doi: 10.1136/bmjopen-2023-075755

FUNDED BY NIHR | National Institute for Health and Care Research

LIVERPOOL CLINICAL TRIALS CENTRE LCTC

ICR

4.1. Physical (an IMP)

4.1.1.,4.1.2. Movement of intervention to participating site or direct to participant

For freight, please refer to section 1.2, 1.3, 1.4 for the calculation and emission factors. If the delivery is not ambient, follow the guidance provided below.

Refrigerated freight

Increase the total kgCO_2e associated with freight by 15% for samples transported at temperatures of 2-8 degrees.

Frozen (dry ice) freight

When calculating the carbon footprint of frozen shipments, make sure to consider the emissions attributable to the dry ice, both in terms of:

1. Weight - If the total weight of the posted package is not available, when estimating the weight of the sample and box, make sure to include the additional weight due to the dry ice (add/include in normal calculation of weight x distance x emission factor).
2. Emissions of dry ice manufacture: for 1 kg of dry ice, you need to account for 2.22 kg of liquid CO_2 using the Ecoinvent 2.2 data below. E.g., 2.22kg x 0.81605 = **1.81kg CO_2e per 1 kg dry ice** produced/used.

261	443	carbon black, at plant	chemicals	inorganics	kg	GLO	2.3658
262	444	carbon dioxide liquid, at plant	chemicals	inorganics	kg	RER	0.81605
263	445	carbon monoxide, CO, at plant	chemicals	inorganics	kg	RER	1.5539
6949	446	cerium concentrate, 60% cerium oxide, at plant	chemicals	inorganics	kg	CN	8.309

If the amount of dry ice used in frozen shipments is unknown, use an estimated 1kg of dry ice per sample box. Ensure this additional weight is included in the freight calculation and 1.81KgCO_2e in the total carbon footprint of the freight.

Assumption: use 1 kg of dry ice per sample box in the absence of established activity data.

Emission factor source: Consultation /estimation by Environmental Resource Management

4.1.3. Materials involved in the packaging and shipment of the intervention

Shipping boxes

For single use (SU) cold storage boxes multiply the number required by 25.2 kgCO_2e

For reusable cold storage boxes, multiply the number required by 2.2 kgCO_2e

Sample Average Shipping Box (85% ambient / 15% frozen)	1.34	kg CO_2e	per cold storage box	The International Journal of Life Cycle Assessment, Goellner et al. Vol 19, pp 611–619 (2014)
Sample Cold Storage Box Manufacture (SU)	25.2	kg CO_2e	per cold storage box	The International Journal of Life Cycle Assessment, Goellner et al. Vol 19, pp 611–619 (2014)
Sample Cold Storage Box Manufacture (Reuse)	2.2	kg CO_2e	per cold storage box	The International Journal of Life Cycle Assessment, Goellner et al. Vol 19, pp 611–619 (2014)

Emission factor source: The International Journal of Life Cycle Assessment, Goellner et al. Vol 19, pp 611-619 (2014) [16]

For cardboard: Kg (cardboard) x 0.821 = kgCO_2e

For polystyrene: kg (polystyrene) x 3.778 = kgCO_2e

Greenhouse gas reporting: conversion factors 2021, GOV.UK [7]

FUNDED BY NIHR | National Institute for Health and Care Research

LIVERPOOL CLINICAL TRIALS CENTRE LCTC

ICR

4.1.4. Destruction of overage

For the destruction of overage, such as the incineration of IMP, multiply the weight in kg of the material being destroyed by the emission factor provided below.

Kg of waste x 2.4252 = kgCO_2e

2958	disposal, hazardous waste, 25% water, to hazardous waste incineration	waste management	hazardous waste incineration	kg	CH	2.4252

Emission factor source: Ecoinvent, version 2.2, 2011 (CH = SWITZERLAND) [6]

4.2. Clinical (e.g. radiotherapy, device, surgery)

NB: not all calculations will be relevant to all interventions. **This section of the method will be further developed as we carbon footprint more trials, so please inform us if your protocol specifies an activity that has not been included, and we will help to determine the associated carbon footprint.**

4.2.1 Movement of intervention, or materials required to deliver the intervention

Please refer to section 1.2 and 4.1.1, 4.1.2.

4.2.2 Materials involved in the shipment of the intervention

Please refer to section 4.1.3.

4.2.3 Utilities required for delivery of the intervention

Please refer to section 7.3 to calculate the emissions attributed to hospital utilities if required to deliver the intervention.

4.2.4 Activities or resources required/relating to delivery of the intervention

Please refer to section 7.2 for consumables, surgery and other activities that may be relevant to the delivery of the intervention, but please take care to avoid double counting.

To calculate the emissions attributed to incineration, please refer to section 4.1.4.

4.3. Other

NB: not all calculations will be relevant to all interventions. **This section of the method will be further developed as we carbon footprint more trials, so please inform us if your protocol specifies an activity that has not been included, and we will help to determine the associated carbon footprint.**

4.3.1 Movement of intervention, or materials required to deliver the intervention

Please refer to section 1.2 and 4.1.1, 4.1.2.

FUNDED BY NIHR | National Institute for Health and Care Research

LIVERPOOL CLINICAL TRIALS CENTRE LCTC

ICR

4.3.2 Materials required for packaging and shipment of the intervention

Please refer to section 4.1.3

4.3.3 Materials or resources required for delivery of the intervention

For printing and paper, please refer to section 1.1.

4.3.4 Travel required to facilitate delivery of the intervention

Please refer to section 3.1, 3.2 for travel.

Griffiths J, *et al. BMJ Open* 2024; 14:e075755. doi: 10.1136/bmjopen-2023-075755

FUNDED BY NIHR | National Institute for Health and Care Research

LIVERPOOL CLINICAL TRIALS CENTRE LCTC

ICR

5 Data collection and exchange

This module includes the following activities:

5.1. Data collection and query exchange between CTU and sites
5.2. Data sent direct from participants to CTU or participating sites
5.3. Data from labs to CTU
5.4. Data from other collaborators to CTU

NB: Analysis of data does not need to be calculated separately, it is covered by the emissions attributed to trial staff FTE in "CTU emissions" and by calculations within "Data Collection and exchange".

5.1. Data collection and query exchange between CTU and sites

CRFs

For postage of paper CRFs, please refer to section 1.2 (freight).

Web-based data entry at sites, e.g. CRF completion, will be accounted for in the time a hospital worker spends on the trial and the carbon footprint of the trial databases (See 5.2).

Scans copied to CD

To estimate the emissions attributed to copying patient scans to a CD, add the carbon footprint of CD manufacture to the carbon footprint of computer use.

The carbon footprint of manufacturing a CD = 0.83 kg $C0_2e$ per CD (833 g $C0_2e$)

Source: Journal of Industrial Ecology, "The Energy and Climate Change Impacts Of Different Music Delivery Methods". Weber et al. Vol 14, Issue 5, pg. 754-769 (2010) [17]

The carbon footprint of copying the scans on to a CD using a computer = 0.18079 kg $C0_2e$ per hour

1269	use, computer, desktop with LCD monitor, active mode	electronics	services	h	RER	0.18079

Emission factor source: Ecoinvent, version 2.2, 2011 [6]

Email traffic

An email without an attachment = 10g CO_2e. Double this for an email with a one-megabyte attachment.

NB: this is an estimate of all emails exchanged between CTU and participating sites throughout the study lifetime, including data query resolution emails.

Source: [Carbon footprint of your emails | mail.com blog] [18]

Griffiths J, *et al. BMJ Open* 2024; 14:e075755. doi: 10.1136/bmjopen-2023-075755

FUNDED BY NIHR | National Institute for Health and Care Research

LIVERPOOL CLINICAL TRIALS CENTRE LCTC

ICR

5.1. (continued)

Data collection via electronic trial databases/systems

The combination of transmitting data and storing it in a data centre requires between 3 to 7 kWh per gigabyte[19]. Therefore multiply 5 kWh by the electricity emission factor (0.273) to calculate the kg CO_2e per GB per year.

5 kwh x 0.273 = 1.365 kg CO_2e per GB per year

Assumption: data storage requires 5 kWh.

Source: Carbon and the Cloud. Hard facts about data storage. | by Stanford Magazine | Stanford Magazine | Medium[19]

5.3. Data sent direct from participants to CTU/participating sites

For paper questionnaires, please refer to section 1.1. for the carbon footprint of producing the materials and section 1.2. for postage (freight).

Electronic questionnaire

For completion of an electronic questionnaire, you must account for both the use of a device to complete the questionnaire and the carbon footprint of data storage and transmission associated with web surfing.

For completion using a **desktop computer**: 0.18079 kg CO_2e per hour

1269	use, computer, desktop with LCD monitor, active mode	electronics	services	h	RER	0.18079

For completion using a **laptop**: 0.028719 kg CO_2e per hour

1284	use, computer, laptop, active mode	electronics	services	h	RER	0.028719

Computer and laptop emission factor source: Ecoinvent, version 2.2, 2011 [6]

For completion using a **tablet**: 0.027397 kg CO_2e per hour

For completion using a **smartphone**: 0.015068 kg CO_2e per hour

Tablet and smartphone emission factor source: Examining the Carbon Footprint of Devices - Sustainable Software (microsoft.com) [20]

Web surfing = 9.441 g CO2e/hr (10 mins = 1.57 g CO_2e)

Source: Resources, Conservation and Recycling, "The overlooked environmental footprint of increasing Internet use". Obringer et al. Vol 167 (2021) [21]

5.4. Data from labs to CTU

For data collection via electronic trial database systems estimate 1.365 kgCO_2e per GB per year.

Source: Carbon and the Cloud. Hard facts about data storage. | by Stanford Magazine | Stanford Magazine | Medium[19]

Griffiths J, *et al. BMJ Open* 2024; 14:e075755. doi: 10.1136/bmjopen-2023-075755

FUNDED BY NIHR | National Institute for Health and Care Research

5.5. Data from other collaborators to CTU

Data linkage

For data linkage, multiple the cost spent by the emission factor provided below.

Computer services – 0.149 x unit (£) = kgCO_2e

42	Real estate activities	Gov.UK	2020	converted fron	£					0.085
43	Renting of machinery e	Gov.UK	2020	converted fron	£					0.232
44	Computer services	Gov.UK	2020	converted fron	£					0.149
45	Research and developn	Gov.UK	2020	converted fron	£					0.216
46	Legal, consultancy and	Gov.UK	2020	converted fron	£					0.121

Emission factor source: Gov.UK Government conversion factors for company reporting of greenhouse gas emissions 2012 - Annex 13 with consideration of 2020 inflation rates. [13]

Griffiths J, *et al. BMJ Open* 2024; 14:e075755. doi: 10.1136/bmjopen-2023-075755

FUNDED BY NIHR | National Institute for Health and Care Research

LIVERPOOL CLINICAL TRIALS CENTRE LCTC

ICR

6 Trial supplies and equipment

This module includes the following activities:

6.1. Equipment used by CTU
6.2. Equipment and supplies used by participating sites supplied by CTU
6.3. Equipment and supplies provided to participants specifically for the trial

6.1. Equipment used by CTU

For office machinery and computers purchased for the trial, multiply the cost by the emission factor provided below.

£ x 0.387 = $kgCO_2e$

17	Machinery and equipme	Gov.UK	2020 converted from £					0.512
18	Office machinery and co	Gov.UK	2020 converted from £					0.387
19	Electrical machinery	Gov.UK	2020 converted from £					0.452

Emission factor source: Gov.UK Government conversion factors for company reporting of greenhouse gas emissions 2012 - Annex 13 with consideration of 2020 inflation rates. [13]

6.2. Equipment and supplies used by participating sites supplied by CTU

For the shipment of equipment to participating sites, please refer to section 1.2.

For the use of a centrifuge, please refer to section 9.2., for a fridge or freezer please refer to section 9.3.

Please inform us if your protocol specifies any equipment or supplies that have not been included and we will help to determine the associated carbon footprint.

6.3. Equipment and supplies provided to participants specifically for the trial

Smartphone: For a smartphone, account for 55 $kgCO_2e$ from manufacture and add 5.5 $kgCO_2e$ per year of usage.

Source: Examining the Carbon Footprint of Devices - Sustainable Software (microsoft.com) [19]

Tablet: For a tablet, account for 119 $kgCO_2e$ from manufacture and add 10kg CO_2e per year of usage. Assume a maximum lifetime of 3 years, therefore 30 kg CO_2e is the total possible carbon footprint that can be attributed to use.

Source: Examining the Carbon Footprint of Devices - Sustainable Software (microsoft.com) [19]

Wearables/smart watch: For a smart watch, account for 30.1 kg CO_2e for manufacture and add 1.633 kg CO_2e per year of usage. Assume a maximum lifetime of 3 years, therefore 4.9 kg CO_2e is the total possible carbon footprint that can be attributed to use.

Source: Apple Watch SE Product Environmental Report [22]

To calculate the carbon footprint associated with shipment of the devices, please refer to section 1.2.

Griffiths J, et al. BMJ Open 2024; 14:e075755. doi: 10.1136/bmjopen-2023-075755

FUNDED BY NIHR | National Institute for Health and Care Research

7 Trial specific patient assessments

This module includes the following activities:

7.1. Travel of patients for study in visits in addition to standard of care (eligibility/screening assessments, trial-specific assessments and procedures)
7.2. Materials and activities required for study assessments in addition to standard of care
7.3. Utilities required for study assessments according to trial staff FTE

7.1. Travel of patients for study in visits that are in addition to standard of care (eligibility/screening assessments, trial-specific assessments and procedures)

Patient travel to elective care (e.g. Hospital)

1 journey by patient associated with attendance at elective care = 2.9 kg CO_2e

Therefore 1 visit to elective care (2 journeys – out and back) = 5.8 kgCO_2e

Patient travel to primary care (e.g. GP)

Emissions associated with attendance at GP surgery (journey out and back) = 1.12 kgCO_2e

Emission factors source: SHC care pathway calculator [23]

7.2. Materials and activities required for study assessments that are in addition to standard of care

This includes everything that happens to the patient in the protocol schedule of assessments which is over and above routine care. This is not an exhaustive list; please inform us if your protocol specifies an activity that has not been included, and we will help to determine the associated carbon footprint.

Surgery

A 1-hour surgery = 53 kg CO_2e

A 30-minute surgery = 26.5 kg CO_2e

Emission factor source: SHC care pathway calculator [23]

Bed days

Low intensity (general ward) = 37.9 kg CO_2e

High intensity (ICU) = 103 kg CO_2e

Emission factor source: SHC care pathway calculator [23]

Scans

1 MRI = 24.7 kg CO_2e

Emission factor source: SHC care pathway calculator [23]

1 CT scan = 9.2 kg CO_2e, 1 CXR = 0.8 kgCO_2e

Emission factor source: The carbon footprint of hospital diagnostic imaging in Australia (thelancet.com) [24]

Griffiths J, et al. BMJ Open 2024; 14:e075755. doi: 10.1136/bmjopen-2023-075755

FUNDED BY NIHR | National Institute for Health and Care Research

LIVERPOOL CLINICAL TRIALS CENTRE LCTC

ICR

7.2. Materials and activities required for study assessments (continued)

Radiotherapy

To calculate the carbon footprint of radiotherapy, multiply the total treatment power in kWh by the electricity emission factor.

Full course of breast radiotherapy (15 sessions) = **5.7 kgCO_2e**

Calculation: 1.4 kWh x 0.273 x 15 = 5.7 kgCO_2e

Full course prostate radiotherapy (20 sessions) = **15.3 kgCO_2e**

Calculation: 2.8 kWh x 0.273 x 20 = 15.3 kgCO_2e

Assumption: 15 sessions required for breast radiotherapy. Source: Radiotherapy for breast cancer - Macmillan Cancer Support [25]

Assumption: 20 sessions required for prostate radiotherapy. Source: Radiotherapy for advanced prostate cancer | Prostate Cancer UK [26]

Benchmark data source: PO-1058 Estimating the carbon footprint of the radiotherapy pathway and changes in response to COVID-19 main.pdf (nih.gov) [27]

Emission factor source: GOV.UK Greenhouse gas reporting, conversion factors 2021 [7]

Consumables

For a trial appointment where consumables such as gloves are required, account for **0.30 kgCO_2e per patient per appointment.**

Emission factor source: SHC care pathway calculator guidance GP consultation module, 2015 [28]

Blood investigations

CO_2 e emissions for haematology tests:
- 82 g/test (95% CI, 73-91 g/test) for coagulation profile
- 116 g/test (95% CI, 101-135 g/test) for full blood examination.

CO_2 e emissions for biochemical tests:
- 0.5 g/test CO_2 e (95% CI, 0.4-0.6 g/test) for C-reactive protein (low because typically ordered with urea and electrolyte assessment)
- 49 g/test (95% CI, 45-53 g/test) for arterial blood gas assessment
- 99 g/test (95% CI, 84-113 g/test) for urea and electrolyte assessment.

NB: These emissions include the materials and consumables required for sample collection, phlebotomy and analysis, as well as power consumption by pathology analysers.

Emission factor sourced from: The carbon footprint of pathology testing. Scott McAlister, Alexandra L Barratt, Katy JL Bell and Forbes McGain. Med J Aust 2020; 212 (8): 377-382. Published online: 4 May 2020 **The carbon footprint of pathology testing - McAlister - 2020 - Medical Journal of Australia - Wiley Online Library** [29]

FUNDED BY NIHR | National Institute for Health and Care Research

LIVERPOOL CLINICAL TRIALS CENTRE LCTC

ICR

7.3. Utilities required for study assessments that are in addition to standard of care

The carbon footprint associated with energy consumption by hospital staff cannot be calculated directly because a hospital and the equipment and staff within in it are used for many other non-trial activities. Emissions are therefore estimated based on average per person emissions and the FTE of the trial hospital staff.

This is calculated by multiplying the average space occupied by a hospital staff member (16.5 m^2) by the kWh used per m^2 of a hospital (81 kWh/m^2). The kWh per FTE per year is then multiplied by the electricity emission factor provided below to calculate the carbon footprint attributed per hospital staff FTE. Finally, multiply by the number of years and FTE applicable**.** The calculation is exemplified below.

Calculation

16.5 m^2 x 81 kWh/m^2 = 1336.5 kWh per FTE per year

- Electricity emission factor = 0.273

1336.5 kWh x 0.273 = **364.9 kgCO_2e per FTE per year**

Multiply 364.9 kgCO_2e by the number of years and FTE applicable - Use the trial SOECAT or the costing included in the initial funding application (if predates use of SOECAT) to establish the FTE or total number of hours required by hospital staff for the trial. If using the number of hours, please follow the below method to establish the FTE required for the calculation.

Number of hours in SOECAT / 1762.5 = FTE required for trial.

e.g. if 449.25 total hospital staff hours required for trial.

449.25 / 1762.5 = 0.25.   Therefore 25% of 1 hospital staff FTE required

364.9 kgCO_2e x 0.25 = 91.2 kgCO_2e

Assumption: The FTE of a nurse/hospital staff is 1762.5 hours. The standard full-time working week for NHS staff is 37.5 hours (1950 hours - 35 days a year off = 1762.5 hours).

Assumption: each health care professional occupies 16.5m^2 room, source HBN 12 (england.nhs.uk) page 32 [30]

Hospital benchmark data source: The Non-Domestic National Energy Efficiency Data-Framework 2022 (England and Wales) (publishing.service.gov.uk). [9]

Electricity emission factor source: Greenhouse gas reporting: conversion factors 2021, GOV.UK [7]

Griffiths J, *et al. BMJ Open* 2024; 14:e075755. doi: 10.1136/bmjopen-2023-075755

FUNDED BY NIHR | National Institute for Health and Care Research — LIVERPOOL CLINICAL TRIALS CENTRE LCTC — ICR

7.3. Utilities required for study assessments that are in addition to standard of care (continued)

Heating

For heating, the calculation follows the same method as above. The 16.5m^2 per person is multiplied by the hospital heating benchmark and then by the natural gas conversion factor provided below.

Hospital building heating benchmark: **193.28** kWh per year per sqm floor area (kWh/m^2)

Natural gas conversion factor: **0.215**

Activity	Referen	EF	Comment	Refrence flow	Scope 1/Direc	Scope 2	Scope 3 T&D	Scope 3 WTT	Total Carbon intensity
Name	Source	Year	Text	Unit	kg CO_2e	kg CO_2e	kg CO_2e	kg CO_2e	kg CO_2e/refrence flow unit
Electricity	Gov.UK	2021		kWh		0.21233	0.00489	0.05529	0.273
Burning (hetaing) oil	Gov.UK	2021		litres	2.54014			0.52807	3.068
Natural gas	Gov.UK	2021		kWh (Gross CV)	0.18316			0.03135	0.215
LPG	Gov.UK	2021		litres	1.55709			0.18383	1.741

16.5 x 193.28 = 3189.1 kWh per FTE per year
3189.1 x 0.215 = 685.7 kgCO_2e per FTE per year

Multiply 685.7 kgCO_2e by the number of years and FTE applicable

Assumption: each health care professional occupies 16.5m^2 room, source HBN 12 (england.nhs.uk) page 32 [30]

Assumption: If the heating source is unknown, assume that the heating source is natural gas.

Hospital benchmark data source: The Non-Domestic National Energy Efficiency Data-Framework 2022 (England and Wales) (publishing.service.gov.uk) [9]

Natural gas emission factor source: Greenhouse gas reporting: conversion factors 2021, GOV.UK [7]

Griffiths J, *et al. BMJ Open* 2024; 14:e075755. doi: 10.1136/bmjopen-2023-075755

FUNDED BY NIHR | National Institute for Health and Care Research

LIVERPOOL CLINICAL TRIALS CENTRE LCTC

ICR

8 Samples

This module includes the following activities:

- **8.1. Materials involved**
- **8.2. Movement of sample kits from manufacturer to CTU**
- **8.3. Movement of sample kits from CTU/distributor to participating sites**
- **8.4. Movement of sample from participating sites to central laboratory**

8.1. Material involved

The emissions attributed to sample collection consumables for common blood tests are included in the blood investigations section of 7.2.

Below is a list of commonly used materials used in sample collection and distribution and their equivalent emission factor. Multiply the kg of the material by the yellow highlighted number (the emission factor) to produce a kgCO_2e.

Paper and board: board	Gov.UK	2021	kg	0.821
Paper and board: mixed	Gov.UK	2021	kg	0.881
Paper and board: paper	Gov.UK	2021	kg	0.919
Plastics: average plastics	Gov.UK	2021	kg	3.116
Plastics: average plastic film	Gov.UK	2021	kg	2.574
Plastics: average plastic rigid	Gov.UK	2021	kg	3.277
Plastics: HDPE (incl. forming)	Gov.UK	2021	kg	3.270
Plastics: LDPE and LLDPE (incl. forming)	Gov.UK	2021	kg	2.601
Plastics: PET (incl. forming)	Gov.UK	2021	kg	4.032
Plastics: PP (incl. forming)	Gov.UK	2021	kg	3.105
Plastics: PS (incl. forming)	Gov.UK	2021	kg	3.778
Plastics: PVC (incl. forming)	Gov.UK	2021	kg	3.413
Glass	Gov.UK	2021	kg	1.403

Example: 100 x 10ml PET blood tubes (such as Streck), weight 5kg.

5kg x 4.032 (emission factor for PET) = 20.2 kgCO_2e

Example: 100 slide mailing containers made of polypropene, weight 1.02 kg

1.02kg x 3.105 = 3.2 kgCO_2e

Emission factor source: Greenhouse gas reporting: conversion factors 2021, GOV.UK [7]

For 8.2., 8.3., and 8.4, please refer to section 1.2. for freight and 4.1.1 for refrigerated or frozen freight.

Griffiths J, *et al. BMJ Open* 2024; 14:e075755. doi: 10.1136/bmjopen-2023-075755

FUNDED BY NIHR | National Institute for Health and Care Research — LIVERPOOL CLINICAL TRIALS CENTRE LCTC — ICR

9 Laboratory

This module includes the following activities:

9.1. Emissions attributed to lab utilities according to staff FTE
9.2. Materials/equipment/consumables used in processing and analysis of samples
9.3. Storage of samples e.g., utilities and ultra-low temperature freezer

9.1. Laboratory utilities according to staff FTE

The carbon footprint associated with energy consumption by laboratory staff cannot be calculated directly because a laboratory, and the equipment and staff within in it, are used for many other non-trial activities. Emissions are therefore estimated based on average per person emissions and the FTE of the trial laboratory staff.

This is calculated by multiplying the average space occupied by a laboratory staff member (40 m^2) by the kWh used per m^2 of a laboratory (160 kWh/m^2). The kWh per FTE per year is then multiplied by the electricity emission factor provided below to calculate the carbon footprint attributed to 1 FTE for 1 year. Finally, multiply by the number of years and FTE applicable**.** The calculation is exemplified below. Use the trial SOECAT or the costing included in the initial funding application (if predates use of SOECAT) to establish the FTE or total number of hours required by laboratory staff.

Calculation

$40m^2$ x 160kWh = 6400 kWh per FTE per year

- Electricity emission factor = 0.273

Name	Source	Year	Text	Unit	kg CO_2e	kg CO_2e	kg CO_2e	kg CO_2e	kg CO_2e/refrence flow unit
Electricity	Gov.UK	2021		kWh		0.21233	0.00489	0.05529	0.273

6400 kWh x 0.273 = 1747.2 kgCO_2e per FTE per year

Multiply 1747.2 kgCO_2e by the number of years and FTE applicable

Heating

For heating, the calculation follows the same method as above. The $40m^2$ per person is multiplied by the laboratory heating benchmark and then by the natural gas conversion factor provided below.

Laboratory fossil thermal typical benchmark: **160** kWh per year per sqm floor area (kWh/m^2)

Natural gas conversion factor**: 0.215**

Activity	Referen	EF	Comment	Refrence flow	Scope 1/Direc	Scope 2	Scope 3 T&D	Scope 3 WTT	Total Carbon intensity
Name	Source	Year	Text	Unit	kg CO_2e	kg CO_2e	kg CO_2e	kg CO_2e	kg CO_2e/refrence flow unit
Electricity	Gov.UK	2021		kWh		0.21233	0.00489	0.05529	0.273
Burning (hetaing) oil	Gov.UK	2021		litres	2.54014			0.52807	3.068
Natural gas	Gov.UK	2021		kWh (Gross CV)	0.18316			0.03135	0.215
LPG	Gov.UK	2021		litres	1.55709			0.18383	1.741

$40m^2$ x 160kWh = 6400 kWh per FTE per year

6400 kWh x 0.215 = 1376 kgCO_2e per FTE per year

Multiply 1376 kgCO_2e by the number of years and FTE applicable

Assumption: For R&D, $40m^2$ required per FTE according to UK EMPLOYMENT DENSITY GUIDE, 3rd edition November 2015 [8]

Assumption: If the heating source is unknown, assume that the heating source is natural gas.

Laboratory benchmark data source: Health Technical Memorandum 07-02: EnCO2de 2015 – making energy work in healthcare (england.nhs.uk) [31]

Emission factor source: Greenhouse gas reporting: conversion factors 2021, GOV.UK [7]

Griffiths J, *et al. BMJ Open* 2024; 14:e075755. doi: 10.1136/bmjopen-2023-075755

FUNDED BY NIHR | National Institute for Health and Care Research
LIVERPOOL CLINICAL TRIALS CENTRE LCTC
ICR

9.2. Materials/equipment/consumables used in processing and analysis of samples

To avoid double counting, use of equipment will be included in lab staff FTE if calculated.

However, if the trial does not involve a central lab, but there is still sample processing on site, please see below. For storage of samples, please see section 9.3.

To calculate the emissions of a piece of equipment, multiply the power consumption in Watts by hours used to get a kWh value. Depending on the equipment, this can often be found in the specifications of a listed product. Finally multiply kWh by the electricity emission factor (0.273).

Example: use of a 310-Watt centrifuge for 15 minutes.

310 Watts x 0.25 (hours) = 77.5 kWh

- 77.5 kWh x 0.273 = 21.2 kg CO_2e

Consider the centrifuge capacity and multiply by the number of uses required.

Emission factor source: Greenhouse gas reporting: conversion factors 2021, GOV.UK [7]

9.3. Storage and destruction of biological samples

For storage of samples in a fridge/-20 freezer or an ultra-low temperature freezer, the kWh usage per day is multiplied by 365 to calculate the kWh per year. This is then multiplied by the electricity emission factor and the number of years the samples are stored. However, you will need to make an assumption about the amount of space in the freezer that the trial samples take up.

Fridge/-20 freezer

- 3kWh/day x 365 days = 1095 kWh per year
- 1095 x 0.273 = 298.9 kgCO_2e per year
- Multiply by number of years stored
- This total is for the whole fridge/-20 freezer for 1 year.

-80 freezer

- 22kWh/day x 365 days = 8030 kWh per year
- 8030 x 0.273 = 2192.2 kgCO_2e per year
- Multiply by number of years stored

This total is for the whole freezer for 1 year. You will need to make an assumption about the amount of space in the freezer that the trial samples take up.

Example: if the samples take up a third of the freezer space - 2192.2 kg CO_2e x 0.333 = 723.4 kg CO_2e

Assumptions: A -80°C freezer uses 22 kWh/day, a -20°C freezer uses 3 kWh/day (Source: Did You Know? - International Laboratory Freezer Challenge) [32]

Emission factor source: Greenhouse gas reporting: conversion factors 2021, GOV.UK [7]

Griffiths J, *et al. BMJ Open* 2024; 14:e075755. doi: 10.1136/bmjopen-2023-075755

10 Trial close out

This module includes the following activities:

10.1. Storage and archiving of essential trial documentation and data
10.2. Storage and destruction of biological samples
10.3. Return of equipment and supplies from participating sites to CTU

Griffiths J, *et al. BMJ Open* 2024; 14:e075755. doi: 10.1136/bmjopen-2023-075755

FUNDED BY NIHR | National Institute for Health and Care Research

LIVERPOOL CLINICAL TRIALS CENTRE LCTC

ICR

10.1. Storage and archiving of essential trial documentation and data

For the storage of archived documentation, estimate the m^2 required for archiving. This estimate will then be multiplied by the most suitable energy benchmark from the list provided below; choose the type of building most similar to the one the documents are stored in e.g. office/lab/warehouse/health building. Finally multiply by the electricity emission factor.

Energy benchmarks:

- office = 59 kWh/m^2
- laboratory = 160 kWh/m^2
- warehouse = 27 kWh/m^2
- health building = 81 kWh/m^2

Calculation
m^2 required x benchmark = kWh

- Electricity conversion factor = 0.273

kWh x 0.273 = kg CO_2e for 1 year of storage

Multiply by number of years necessary.

Heating
For heating, use the same method as above. Estimate the m^2 used and multiply by the corresponding heating benchmark (i.e., if 'laboratory' was used above then select the same for this calculation). Multiply by the emission factor for natural gas provided below.
Benchmarks:

- office = 167.27 kWh/m^2
- laboratory = 160 kWh/m^2
- warehouse = 62 kWh/m^2
- health building = 193.28 kWh/m^2

Calculation
m^2 required x benchmark = kWh

- Natural gas conversion factor: **0.215**

kWh x 0.215 = kgCO_2e for 1 year of storage

Multiply by number of years necessary.

Assumption: If the heating source is unknown, make assumption the heating source is natural gas.

Benchmark data source: The Non-Domestic National Energy Efficiency Data-Framework 2022 (England and Wales) (publishing.service.gov.uk) [9]

Emission factor source: Greenhouse gas reporting: conversion factors 2021, GOV.UK [7]

Griffiths J, *et al. BMJ Open* 2024; 14:e075755. doi: 10.1136/bmjopen-2023-075755

FUNDED BY NIHR | National Institute for Health and Care Research

LIVERPOOL CLINICAL TRIALS CENTRE LCTC

ICR

10.1. Storage and archiving of essential trial documentation and data (continued)

For electronic data or documentation storage, estimate 1.365 $kgCO_2e$ per GB per year.

Source: Carbon and the Cloud. Hard facts about data storage. | by Stanford Magazine | Stanford Magazine | Medium[19]

10.2. Storage and destruction of biological samples

See section 9.3. for storage of refrigerated or frozen samples.

See section 10.1 for storage of ambient samples.

10.3. Return of equipment and supplies from participating sites to CTU

See section 1.2. for freight.

Griffiths J, *et al. BMJ Open* 2024; 14:e075755. doi: 10.1136/bmjopen-2023-075755

FUNDED BY NIHR | National Institute for Health and Care Research LIVERPOOL CLINICAL TRIALS CENTRE LCTC ICR

References

[1] Sustainable Trials Study Group. Towards sustainable clinical trials. *BMJ* 2007;334:671
[2] Lyle K, Dent L, Bailey S, et al. Carbon cost of pragmatic randomised controlled trials: retrospective analysis of sample of trials. *BMJ* 2009;339:b4187
[3] Making clinical trials sustainable [Internet]. The Sustainable Healthcare Coalition. [cited 2023 May 11]. Available from: https://shcoalition.org/clinical-trials/
[4] Climate change and health [Internet]. World Health Organization. 2021 October 30 [cited 2023 May 11]. Available from: https://www.who.int/news-room/fact-sheets/detail/climate-change-and-health
[5] What is an emission factor? [Internet]. Climfoot-project.eu. [cited 2023 May 11]. Available from: https://climfoot-project.eu/en/what-emission-factor
[6] Ecoinvent, version 2.2. 2011. Available from: https://ecoinvent.org/the-ecoinvent-database/data-releases/ecoinvent-version-2/
[7] Greenhouse gas reporting: conversion factors [Internet]. GOV.UK 2021. [cited 2023 May 11]. Available from: https://www.gov.uk/government/publications/greenhouse-gas-reporting-conversion-factors-2021
[8] EMPLOYMT DENSITY GUIDE (3rd Edition) [Internet]. Homes & Communities Agency. November 2015 [cited 2023 May 11]. Available from: https://www.kirklees.gov.uk/beta/planning-policy/pdf/examination/national-evidence/NE48_employment_density_guide_3rd_edition.pdf
[9] The Non-Domestic National Energy Efficiency Data-Framework (England and Wales) [Internet]. Department for Business, Energy & Industrial Strategy. 2022 [cited 2023 May 11]. Available from: https://assets.publishing.service.gov.uk/government/uploads/system/uploads/attachment_data/file/1086903/non_domestic_need_data_framework_2022.pdf
[10] Greenhouse gas reporting: conversion factors 2022 [Internet]. Gov.uk. 2022 [cited 2023 May 11]. Available from: https://www.gov.uk/government/publications/greenhouse-gas-reporting-conversion-factors-2022
[11] Transport Statistics Great Britain: 2021 [Internet]. GOV.UK (www.gov.uk). 2021 December 16 [cited May 11 2023]. Available from: https://www.gov.uk/government/statistics/transport-statistics-great-britain-2021/transport-statistics-great-britain-2021
[12]Commuting trends in England 1988-2015 [Internet]. GOV.UK. 2017 November 7 [cited 2023 May 11]. Available from: https://www.gov.uk/government/publications/commuting-trends-in-england-1988-to-2015
[13] Kayla Wiles. Turn off that camera during virtual meetings, environmental study says [Internet]. Purdue University News. 2021 January 14 [cited 2023 May 11]. Available from: https://www.purdue.edu/newsroom/releases/2021/Q1/turn-off-that-camera-during-virtual-meetings,-environmental-study-says.html
[14] Gov.UK Government conversion factors for company reporting of greenhouse gas emissions 2012 – Annex 13 (with consideration of 2020 inflation rates)
[15] WWF. 2018. Food in a warming world [Internet]. [cited 2023 May 11]. Available from: https://www.wwf.org.uk/sites/default/files/2018-03/Food_in_a_warming_world_report.PDF?source=post_page---------------------------
[16] Goellner et al. The International Journal of Life Cycle Assessment. Vol 19, pp 611-619 (2014)
[17] Weber et al. Journal of Industrial Ecology, "The Energy and Climate Change Impacts Of Different Music Delivery Methods". Vol 14, Issue 5, pg. 754-769 (2010)
[18] What's the carbon footprint of an email? [Internet]. Mail.com blog. 202 April 21 [cited 2023 May 11]. Available from: https://www.mail.com/blog/posts/email-carbon-footprint/9/
[19]Justin Adamson. Carbon and the Cloud. Hard facts about data storage [Internet]. Stanford Magazine. 2017 May 15 [cited 2023 May 11]. Available from: https://medium.com/stanford-magazine/carbon-and-the-cloud-d6f481b79dfe
[20] Srilatha Manne. Examining the Carbon Footprint of Devices [Internet]. Microsoft.com. 2020 November 23 [cited 2023 May 11. Available from: https://devblogs.microsoft.com/sustainable-software/examining-the-carbon-footprint-of-devices/
[21] Obringer, R., Rachunok, B., Maia-Silva, D., Arbabzadeh, M., Nateghi, R., & Madani, K. (2021). The overlooked environmental footprint of increasing Internet use. *Resources, Conservation and Recycling*, *167*, [105389]. https://doi.org/10.1016/j.resconrec.2020.105389
[22] Apple watch SE. Product Environmental Report – Apple [Internet]. [cited 2023 May 11]. Available from: https://www.apple.com/by/environment/pdf/products/watch/Apple_Watch_SE_PER_sept2020.pdf
[23] Care pathways carbon footprint calculator [Internet]. Sustainable Healthcare Coalition. [cited 2023 May 11]. Available from: https://shcpathways.org/full-calculator/
[24] McAlister S, McGain F, Petersen M, Story D, Charlesworth K, Ison G, Barratt A. The carbon footprint of hospital diagnostic imaging in Australia. Lancet Reg Health West Pac. 2022 May 3;24:100459. doi: 10.1016/j.lanwpc.2022.100459. PMID: 35538935; PMCID: PMC9079346.
[25] Radiotherapy for breast cancer [Internet]. Macmillan.org. [cited 2023 May 11]. Available from: https://www.macmillan.org.uk/cancer-information-and-support/treatments-and-drugs/radiotherapy-for-breast-cancer
[26] Radiotherapy for advanced prostate cancer [Internet]. Prostate Cancer UK. [cited 2023 May 11]. Available from: https://prostatecanceruk.org/prostate-information-and-support/treatments/radiotherapy-for-advanced-prostate-cancer
[27] J. Cummings, C. Taylor, R. Chuter1. PO-1058 Estimating the carbon footprint of the radiotherapy pathway and changes in response to COVID-19. Radiotherapy and oncology. 2022. DOI:10.1016/S0167-8140(22)03022-5

FUNDED BY NIHR | National Institute for Health and Care Research

LIVERPOOL CLINICAL TRIALS CENTRE LCTC

ICR

[28] SHC care pathway calculator guidance, GP consultation module, page 18, 2015. Available from: https://shcoalition.org/sustainable-care-pathways-guidance/

[29] McAlister, S., Barratt, A.L., Bell, K.J. and McGain, F. (2020), The carbon footprint of pathology testing. Med. J. Aust., 212: 377-382. https://doi.org/10.5694/mja2.50583

[30] Health Building Note (HBN) 12, page 32. NHS Estates. 2004 [cited 2023 May 11]. Available from: https://www.england.nhs.uk/wp-content/uploads/2021/05/HBN_12.pdf

[31] Health Technical Memorandum 07-02: EnCO2de 2015 – making energy work in healthcare. Department of Health. 2015 [cited 2023 May 11]. Available from: https://www.england.nhs.uk/wp-content/uploads/2021/05/HTM_07-02_Part_A_FINAL.pdf

[32] Freezer challenge blog. Did you know? [Internet]. Freezerchallenge.org. 2020 June 19 [cited 2023 May 11]. Available from: https://www.freezerchallenge.org/fc-blog/did-you-know

Acknowledgements

This work was produced through funding from the NIHR entitled **NIHR Clinical Trials Unit Support Funding Opportunity – Supporting efficient / innovative delivery of NIHR research**

Authors: Jessica Griffiths, Lisa Fox, Paula Williamson

On behalf of the Low Carbon Clinical Trials working group: Fiona Adshead, Rustam Al-Shahi Salman, Craig Anderson, Emma Bedson, Judith Bliss, Ana Boshoff, Xiaoying Chen, Denise Cranley, Peter Doran, Carrol Gamble, Kerenza Hood, Naomi McGregor, Carolyn McNamara, Elis Midha, Keith Moore, Alexis M Perkins, Sarah Pett, Matthew R Sydes.

Grateful thanks to Environmental Resources Management for their technical support and expertise in the development of the method.
