# Supplementary material for: Quantifying the carbon footprint of clinical trials: guidance development and case studies
Source: BMJ Open. 2024 Jan 24;14(1):e075755. doi: 10.1136/bmjopen-2023-075755 (PMC10823997; doi:10.1136/bmjopen-2023-075755)
Supplement: Supplementary data [file bmjopen-2023-075755supp003.pdf]

FUNDED BY NIHR | National Institute for Health and Care Research

LIVERPOOL CLINICAL TRIALS CENTRE LCTC

ICR

Enabling lower carbon clinical trials: Development and prototype testing of a method to quantify the carbon footprint of clinical trials to inform future lower carbon clinical trial design

Guidance and method to calculate the carbon footprint of a clinical trial

Data collation quick guide and worksheet

This guidance provides information on how to carbon footprint a clinical trial for the purposes of the NIHR-funded project 'enabling lower carbon clinical trials.'

Within the guidance, clinical trial processes have been sub-divided into the following modules:

1. Trial set up
2. CTU emissions
3. Trial specific meetings and travel
4. Treatment intervention
5. Data collection and exchange
6. Trial supplies and equipment
7. Trial specific patient assessments
8. Samples
9. Laboratory
10. Trial close out

This list is not exhaustive, and it is expected that further activities and modules may need to be added to account for specialist processes in all clinical trial types.

NB: analysis of data does not need to be calculated separately, it is covered by the emissions attributed to trial staff FTE in "CTU emissions" and calculations included within "Data Collection and exchange".

Griffiths J, *et al. BMJ Open* 2024; 14:e075755. doi: 10.1136/bmjopen-2023-075755

FUNDED BY NIHR | National Institute for Health and Care Research

LIVERPOOL CLINICAL TRIALS CENTRE LCTC

ICR

In addition to this quick guide and worksheet, we have produced a detailed guidance and method document defining the project scope, limitations and assumptions. The detailed guidance contains a more in depth look and explanation of the calculations found in this document, including emission factor and benchmark data sources, and should be referred to when using this worksheet.

Introduction to calculating carbon footprint

A carbon footprint is a measure of greenhouse gases, usually quoted in kg or tonnes of carbon dioxide equivalent (CO_2e). To calculate the carbon footprint of a particular clinical trial process, both 'activity data' and 'emission factors' are required.

An emission factor, also known as a conversion factor, "is a coefficient which allows you to convert activity data into greenhouse gas emissions. It is the average **emission** rate of a given source, relative to units of activity or process/processes."[1]

The activity data is provided by the user and multiplied by the emission factors provided in this guidance document.

Data collation quick guide and worksheet

This data collation quick guide should be used in conjunction with the "Enabling lower carbon clinical trials: Development and prototype testing of a method to quantify the carbon footprint of clinical trials to inform future lower carbon clinical trial design - Detailed Guidance and method to calculate the carbon footprint of a clinical trial". The guidance document provides the detailed explanation of how calculations should be considered and calculated. This quick guide should be used to collate the trial-specific processes, necessary activity data and to record the subsequent calculations. It is important to avoid double-counting activities i.e., modules must not include activities already covered elsewhere in the clinical trial process map. Please complete this worksheet for each trial to be carbon footprinted.

NB: We are using the term 'CTU' to describe the organisation that manages all aspects of central trial management. For some institutions some of those tasks maybe done by groups outside the CTU team e.g., sponsor office/CRO etc.

Griffiths J, *et al. BMJ Open* 2024; 14:e075755. doi: 10.1136/bmjopen-2023-075755

FUNDED BY NIHR | National Institute for Health and Care Research

LIVERPOOL CLINICAL TRIALS CENTRE LCTC

ICR

CASPS carbon footprint calculations

CASPS is an international phase II trial of CEDIRANIB in the treatment of patients with alveolar soft part sarcoma. The trial enrolled 47 patients across twelve sites in the UK, Spain, and Australia and involved an internationally shipped IMP, completion and shipment of paper CRF, on site visits for site initiation, monitoring visits and audits, additional hospital visits for patients for trial-specific patient assessments and provision of samples.

Module	Examples	Trial activity data	Calculation	Results
**1. Trial set-up**				
1.1. Production of trial documentation to be sent to sites or participants	E.g. Site Investigator File and contents, Site Pharmacy File and contents, CRF Folder and contents, PIS/Cs, GP letters	12 sites, 47 patients<br><br>SIF: 401 pages, 12 sent (4812 pages)<br>Site pharmacy file: 301 pages, 11 sent (3311 pages)<br>CRF folder: 76 pages in set up pack, 17 sent (1292 pages)<br>PIS/C 1 = 17 pages, 27 patients (799 pages)<br>PIS/C 2 = 16 pages, 47 patients (762)<br>Letters inc. GP = 8 pages, 47 patients (376)<br><br>Total = 11352 pages<br><br>Number of folders used to send trial documentation: estimate 23 large ring binders (0.5kg) and 18 small (0.3kg)	**Paper:**<br>[no. of page] x 0.005 = paper weight (kg)<br><br>b/w printing: Kg of paper x 0.22438 = $kgCO_2e$<br>Colour printing: Kg of paper x 0.31786 = $kgCO_2e$<br>Materials (paper): Kg of paper x 0.919 = $kgCO_2e$<br><br>Folders: Kg (of cardboard) x 0.821 = $kgCO_2e$<br>Assumption: Weight of lever arch = 0.5kg<br>Assumption: Weight of ring binder = 0.3kg<br><br>Paper:<br>11352 pages x 0.005 = 56.76kg<br>Printing: 56.76 kg x 0.22438 = 12.7 $kgCO_2e$<br>Materials: 56.76 kg x 0.919 = 52.2 $kgCO_2e$<br><br>Folders:<br>(23 x 0.5) + (18 x 0.3) = 16.9kg<br>16.9kg x 0.821 = 13.9 $kgCO_2e$	78.8 $kgCO_2e$

Griffiths J, *et al. BMJ Open* 2024; 14:e075755. doi: 10.1136/bmjopen-2023-075755

FUNDED BY NIHR | National Institute for Health and Care Research

LIVERPOOL CLINICAL TRIALS CENTRE LCTC

ICR

1.2. Provision/postage of trial documentation to sites<br><br>1.3. Provision/postage of documentation to participants by CTU or participating sites<br><br>1.4. Provision/postage of incentives to participant	E.g. Site Investigator File and contents, Site Pharmacy File and contents, CRF Folder and contents, PIS/Cs, GP letters	Weight and distance of deliveries (t.km):<br>UK sites<br>- RMH - 0.00519 tonnes x 24.1km = 0.125079 t.km<br>- Christie - 0.0092 x 355.7km = 3.275997 t.km<br>- Bristol - 0.0063 tonnes x 196.3 km= 1.23669 t.km<br>- UCLH - 0.00519 tonnes x 25.7km = 0.133383 t.km<br>- Newcastle - 0.008725 tonnes x 506.9km = 4.4227 t.km<br>- Nottingham - 0.00519 tonnes x 259.1km = 1.344729 t.km<br>Australia<br>0.00587 tonnes x (17018.5km+16544km) = 197 t.km<br><br>Spain<br>0.00519 x (1147km + 1244km + 1091km) = 18.07 t.km<br><br>GP letter<br>Weight of GP letter:<br>2x5g + envelope (7g) = 17g /0.000017tonnes<br>0.000017 tonnes x 47 patients = 0.000799 tonnes	Delivery weight (tonnes) x distance (km) = t.km<br><br>For road freight: t.km x 0.19443 = $kgCO_2e$<br><br>For air freight: t.km x required emission factor below = $kgCO_2e$<br>▪ Domestic (to/from UK) = 4.98549<br>▪ Short haul (to/from UK) = 2.55439<br>▪ Long-haul (to/from UK) = 1.13047<br>▪ International (to/from non-UK) = 1.13047<br><br>UK<br>Total t.km = 10.539289 x 0.19443 = 2.05 $kgCO_2e$<br><br>Australia<br>197 t.km x 1.13047 = 222.7 $kgCO_2e$<br><br>Spain<br>18.07 t.km x 2.55439 = 46.2 $kgCO_2e$<br><br>GP letter postage:<br>For delivery of trial supplies to patients or GP, if unknown, use 17.4km as distance from hospital to patient, or hospital to GP.<br><br>0.000799 tonnes x 17.4km = 0.014 t.km<br>0.014 t.km x 0.19443 = 0.002722 $kgCO_2e$	271 $kgCO_2e$

Griffiths J, *et al. BMJ Open* 2024; 14:e075755. doi: 10.1136/bmjopen-2023-075755

FUNDED BY NIHR | National Institute for Health and Care Research

LIVERPOOL CLINICAL TRIALS CENTRE LCTC

ICR

			6 letters sent to each patient - 0.002722 x 6 = 0.016 $kgCO_2e$*	
**2. CTU emissions**				
2.1. Energy consumption at CTU according to trial staff FTE	E.g. energy consumption per square metre of air-conditioned office space	Trial duration: 6 years<br><br>Trial staff FTE: UK - 1, Spain - 0.3, Australia - 0.5	**Energy consumption for 1 FTE for 1 year** = 193.28 $kgCO_2e$<br>Multiply by the number of years and FTE applicable<br><br>UK CTU emissions = 193.28 $kgCO_2e$ x 6 = 1159.7 $kgCO_2e$<br><br>Australia CTU<br>- location: Victoria<br>- Office building energy consumption = 231.39 kwh/m^2 source: baseline-energy-consumption-part_1-report-2012.pdf. energy split is 90% electricity, 10% gas. Therefore electricity = 208.251 kwh/m^2<br>- Energy usage per staff (per 12 m^2) = 12 m^2 x 208.251 kWh =2499.012 kwh<br>- Victoria electricity emission factor = 0.98 kg CO2-e/kWh (Source: EERS release 2020–21 (cleanenergyregulator.gov.au))<br>- 2499.012 kWh x 0.98 = 2449$kgCO_2e$ per FTE per year<br>- 2449 $kgCO_2e$ x 0.5 (FTE) x 6 years = 7347$kgCO_2e$	8854.6 $kgCO_2e$

* Assumption: as the international sites are also located in cities, the same patient to GP distance was used for Australia and Spain

Griffiths J, *et al. BMJ Open* 2024; 14:e075755. doi: 10.1136/bmjopen-2023-075755

FUNDED BY NIHR | National Institute for Health and Care Research   LIVERPOOL CLINICAL TRIALS CENTRE LCTC   ICR

			Spain trial staff<br>- UK benchmark data used as a proxy for average office building energy consumption because like the UK, Spain has a reasonably contribution from renewables.<br>- 193.284 $kgCO_2e$ x 6 x 0.3 = 347.9 $kgCO_2e$	
2.2. Heating	E.g. energy consumption at coordination centre attributed to heating (natural gas)	Trial duration: 6 years<br><br>Trial staff FTE: UK - 1, Spain - 0.3, Australia - 0.5	**Heating for 1 FTE for 1 year** = 431.56 $kgCO_2e$<br>Multiply by the number of years and FTE applicable<br>UK = 431.56 $kgCO_2e$ x 6 = 2589.4$kgCO_2e$<br><br>Australia<br>- In office buildings energy split is 90% electricity, 10% gas. Office gas intensity = 23.139 kwh/m^2<br>- Energy usage per staff (per 12 m^2) = 12m^2 x 23.139 kWh = 277.668 kWh<br>- 277.668 kWh = 1GJ<br>- Australian National Greenhouse Accounts Factors (dcceew.gov.au) Emission factor for natural gas: Scope 1 = 51.3 $kgCO_2e$/GJ, Scope 3 = 4.0 $kgCO_2e$/GJ<br>- (1x 51.53 $kgCO_2e$/GJ) + (1x4.0 $kgCO_2e$/GJ)<br>- = 55.53 $kgCO_2e$	3532.8 $kgCO_2e$

Griffiths J, et al. BMJ Open 2024; 14:e075755. doi: 10.1136/bmjopen-2023-075755

FUNDED BY NIHR | National Institute for Health and Care Research; LIVERPOOL CLINICAL TRIALS CENTRE LCTC; ICR

			- 55.53 $kgCO_2e$ x 0.5 (FTE) x 6 years = Total = 166.59 $kgCO_2e$<br>Spain<br>- UK benchmark and emission factor data used as a proxy for average office building energy consumption.<br>- 431.56 $kgCO_2e$ x 0.3 x 6 = 776.8 $kgCO_2e$	
2.3. Trial team commuting	E.g. Car, rail, bus, walking etc	Trial duration: 6 years<br><br>Trial staff FTE: UK - 1, Spain - 0.3, Australia - 0.5	For 1 FTE for 1 year, total average commuting emissions = 1027.8 $kgCO_2e$[†]<br>Multiply by the number of years and FTE applicable<br><br>UK = 1027.8 x 6 = 6166.8 $kgCO_2e$<br>Australia = 1027.8 $kgCO_2e$ x 0.5 x 6 years = 3083.4 $kgCO_2e$<br>Spain = 1027.8 $kgCO_2e$ x 0.3 x 6 years = 1850 $kgCO_2e$	11100.2 $kgCO_2e$
**3. Trial specific meetings and travel**				
3.1. Visits and travel to site<br>3.2. Travel to meetings	E.g. Feasibility, site initiation and monitoring visits, audits, inspections, Trial Management Group (TMG), Trial Steering Committee (TSC), Independent Data Monitoring Committee (IDMC),	Australia feasibility visit:<br>- Flight from Melbourne to Brisbane<br>- Estimated distance travelled = 1375 km<br>- Number of passengers = 2<br><br>Site Initiation visits:<br>Assumption: Rail used for all travel UK site visits	Number of passengers x total distance (km) = p.km<br><br>For national rail: p.km x 0.04441= $kgCO_2e$<br><br>For flights: p.km x relevant emission factor below:<br>- Domestic (average): 0.27278<br>- Short-haul (average) to/from UK: 0.17034	19433 $kgCO_2e$

[†] Assumption: the average commuting statistics for the UK were found to be similar to those for Spain and Australia and therefore also applied to the international CTUs.

Griffiths J, *et al. BMJ Open* 2024; 14:e075755. doi: 10.1136/bmjopen-2023-075755

	and investigator meetings, Patient and Public Involvement and Engagement (PPIE), conferences, scientific meetings etc	UK total p.km = 3868.6 p.km<br>Australia total p.km = 9736 p.km<br>Spain: 6 teleconferences, 34 total attendees<br><br><u>Monitoring visits:</u><br>UK total p.km = 12957.4 p.km<br>Australia total p.km = 15310 p.km<br>Spain total p.km = 3280 p.km<br><br><u>Audits</u><br>Long haul to/from UK flights = 29442 p.km<br>Short haul = 13464 p.km<br>International = 11444 p.km<br><br><u>Other</u><br>- In person visit to Spanish CTU, 2 ICR-CTSU travelled to Barcelona: 2x2x(1309km) = 5236 p.km<br>- RMH lab visit, 115.8 p.km<br><br><u>Meetings</u><br>Joint IDMC/TSC: all teleconferences, 5 meetings, 3 external attendees<br>TMG: 5 meetings, all teleconferences, 16 attendees<br><br><u>Conferences:</u>	- Long-haul (average) to/from UK: 0.21423<br>- International (average) to/from non-UK: 0.20373<br><br>NB: Distances may be calculated using google maps and calculated from CTU to destination<br><br>**Videoconferencing** = 157 grams CO_2e per hour.<br><br><u>Feasibility visits</u><br>Australia:<br>- 2 x (2 x 1375) = 5500 p.km<br>- 5500 p.km x 0.20373 = 1120.5 $kgCO_2e$<br><br><u>Site initiation and pharmacy visits</u><br>UK: 3868.6 p.km x 0.0441 = 171.8 $kgCO_2e$<br>1 teleconference , 4 attendees – 0.157 $kgCO_2e$/hr x 4 = 0.628 $kgCO_2e$<br>Australia: 9736 p.km x 0.20373 = 1983.5 $kgCO_2e$<br>Spain (all teleconference) = 34 x 0.157 $kgCO_2e$/hr = 5.3 $kgCO_2e$<br><br><u>Monitoring visits</u><br>UK: 12957.4 p.km x 0.04441 = 575.4 $kgCO_2e$<br>Australia: 11074 p.km x 0.20373 = 3119.1 $kgCO_2e$<br>Spain: 3280 p.km x 0.17034 = 558.7 $kgCO_2e$<br><br><u>Audits</u>	

Griffiths J, *et al. BMJ Open* 2024; 14:e075755. doi: 10.1136/bmjopen-2023-075755

FUNDED BY NIHR | National Institute for Health and Care Research — LIVERPOOL CLINICAL TRIALS CENTRE LCTC — ICR

		- 2 attendees to a conference in Bristol, p.km = 785.2<br>- 1 attendee to conference in Nottingham, p.km = 518.2	Long haul to/from UK flights = 29422 p.km x 0.21423 = 6303.1 $kgCO_2e$<br>Short-haul = 13464 p.km x 0.17034 = 2293.5 $kgCO_2e$<br>International = 11444 p.km x 0.20373 = 2331.5 $kgCO_2e$<br><br>Other<br>- ICR-CTSU trial staff visit to Barcelona: 5236 p.km x 0.17034 = 891.9 $kgCO_2e$<br>- Lab visit: 115.8p.km x 0.04441 = 5.14 $kgCO_2e$<br><br>Meetings<br>IDMC/TSC: 5x3x 0.157 $kgCO_2e$/hr = 2.4 $kgCO_2e$<br>TMG: 5 x 18 x 0.157 $kgCO_2e$/hr = 12.6 $kgCO_2e$<br><br>Conferences<br>- Bristol: 785.2 p.km x0.04441 = 34.9 $kgCO_2e$<br>- Nottingham: 518.2 p.km = 23 $kgCO_2e$	
3.3. Hotel stays	E.g. monitoring visits, audits, inspections etc	UK hotel stays: 30 stays<br>Assumption: Hotel stays for all visits to Christie, Nottingham and Newcastle (same day visits to RMH, Bristol and UCLH)<br><br>Visit to Spain CTU: 1 room, 7 nights<br><br>Australia: 1 room, 3 nights	For UK: number of hotel rooms x number of nights x 13.9 = $kgCO_2e$<br><br>For UK (London) = number of rooms x number of nights x 13.8 = $kgCO_2e$.<br><br>For other countries use conversion factors from orange 'hotel stay' tab: conversion-factors-2021-full-set-advanced-users.xlsm (live.com)	675.7 $kgCO_2e$

Griffiths J, *et al. BMJ Open* 2024; 14:e075755. doi: 10.1136/bmjopen-2023-075755

			Alternatively, you may use cost-based method:<br>£ spent x 0.358 = $kgCO_2e$<br><br>Trial calculations:<br>UK hotel stays for monitoring visits:<br>13.9 x 30 = 417 $kgCO_2e$<br><br>Visits to Spain CTU:<br>7 x 18.7 = 130.9 $kgCO_2e$<br><br>Australia hotel stays:<br>42.6 x 3 = 127.8 $kgCO_2e$	
3.4. Sustenance	E.g. meeting lunches, hotel dinners	Number of lunches or dinners:<br><br>Or<br><br>£ spent: £503.65	Meeting lunches or hotel dinners (vegetarian) = 2.6 $kgCO_2e$ per meal per person<br><br>Meeting lunches or hotel dinners (meat) = 5.92 $kgCO_2e$ per meal per person<br><br>Or cost-based approach = £ x 0.708 = $kgCO_2e$<br>£503.65 x 0.708 = 356.6 $kgCO_2e$	356.6 $kgCO_2e$
**4. Intervention[‡]**<br>**4.1. Physical (IMP)**<br>**4.2. Clinical (non-IMP)**<br>**4.3. Other (not captured above)**<br>**Please fill out the section most relevant to the intervention being investigated.**				
**4.1. Physical**				

[‡]As per assumptions detailed in the guidance, manufacture of the intervention is considered out of scope. This section defines all processes relating to providing and delivering the trial intervention that are over and above routine care.

FUNDED BY NIHR | National Institute for Health and Care Research

LIVERPOOL CLINICAL TRIALS CENTRE LCTC

ICR

4.1.1, 4.1.2. Movement of intervention, or materials required to deliver the intervention	E.g. movement of intervention from manufacturing site to distribution site, shipment of IMP to participating sites or direct to participant	NB: all shipping of IMP ambient<br><br>Movement of IMP from manufacturing site to QP site for QP declaration:<br>Assumption: 1 shipment<br>Assumption: 1 pill bottle contains 35 tablets - weight ~30g<br>Assumption: ~4000 IMP bottles shipped<br>Flight distance from Puerto Rico to QP site = 6697 km<br>Shipment weight = 4000x 30g = 120000g = 0.118 tonnes<br><br>Movement of IMP from QP site to distribution/packaging site:<br>Distance from QP site to distribution/packaging site = 352.4 km<br>Shipment weight = 0.118 tonnes<br>Movement of IMP from distribution/packaging site to participating sites:<br>UK deliveries: 7.7 t.km<br>Spain deliveries: 13 t.km<br>Australia deliveries: 184.6 t.km	Carry out freight calculation as described in section 1.2.<br><br>Movement of IMP from manufacturing site to Macclesfield for QP declaration:<br>▪ Long-haul (to/from UK) = 1.13047<br>▪ 0.118 tonnes x 6697km = 790.246 t.km<br>▪ 790.246 t.km x 1.13047 = 893.4 $kgCO_2e$<br><br>Movement of IMP from manufacturing site to distribution/packaging site (Fisher Clinical services):<br>▪ 0.118 tonnes x 352.4km = 41.5832 t.km<br>▪ 41.5832 t.km x 0.19443 = 8.1 $kgCO_2e$<br><br>Movement of IMP from distribution/packaging site to participating sites:<br>UK deliveries: 7.7 t.km x 0.19443 = 1.5 $kgCO_2e$<br>Spain deliveries:<br>▪ short - haul (to/from UK) = 2.55439<br>▪ 13 t.km x 2.55439 = 33.3 $kgCO_2e$<br>Australia deliveries:<br>▪ Long-haul (to/from UK) = 1.13047<br>▪ 184.6 t.km x 1.13047 = 208.7 $kgCO_2e$	1145 $kgCO_2e$
4.1.3. Materials required for the packaging and shipment of IMP	E.g. cardboard, cold storage boxes, polystyrene	Kg of cardboard/ polystyrene:	E.g. Single use sample cold storage box = 25.2 $kgCO_2e$ per box	Data for IMP shipment packaging

Griffiths J, *et al. BMJ Open* 2024; 14:e075755. doi: 10.1136/bmjopen-2023-075755

FUNDED BY NIHR | National Institute for Health and Care Research LIVERPOOL CLINICAL TRIALS CENTRE LCTC ICR

			E.g. Reusable sample cold storage box = 2.2 $kgCO_2e$ per box<br><br>E.g. Kg (cardboard) x 0.821 = $kgCO_2e$<br>E.g. Kg (polystyrene) x 3.778 = $kgCO_2e$	unobtainable from trial records. Item not included for CASPS.
4.1.4. Destruction of overage	E.g. incineration of IMP	Estimated weight of overage incinerated:<br>- Total bottles destroyed = 1464<br>- 1464 x 30g = 43.92 kg	Kg of waste x 2.4252 = $kgCO_2e$<br>43.92 kg x 2.4252 =106.5 $kgCO_2e$	106.5 $kgCO_2e$
**5. Data collection and exchange**<br>NB: analysis of data does not need to be calculated separately, it is covered by the emissions attributed to trial staff FTE in "CTU emissions" and calculations included within "Data Collection and exchange".				
5.1. Data collection and query exchange between CTU and sites	E.g. CRFs, EDC completion and query resolution, scans copied to CDs	CASPS hospital files<br>35 x large folders (500 pages)<br>- 500 x 35 = 17500<br>104 x small (150 pages)<br>- 104 x 150 = 15600<br>Total pages = 17500 + 15600 = 33100 pages<br><br>Scans<br>3 scans copied onto a CD per patient, 47 patients total<br>5 mins laptop time to burn CD per patient<br><br>Postage of scans:<br>UK: 0.278671 t.km	CASPS hospital files<br>33100 x 0.005kg = 165.5 kg<br>Paper (printing) = 165.5kg x 0.22438= 37 $kgCO_2e$<br>Paper (materials) = 165.5kg x 0.919 = 152.1 $kgCO_2e$<br><br>Scans<br>The carbon footprint of manufacturing a CD = 0.83 $kgCO_2e$ per CD<br>0.83 $kgCO_2e$ x 47 = 39.01 $kgCO_2e$<br><br>The carbon footprint of copying the scans on to a CD using a computer = 0.18079 $kgCO_2e$ per hour<br>5mins x 47 = 235 minutes = 3.91666667 hours	558.3 $kgCO_2e$

Griffiths J, *et al. BMJ Open* 2024; 14:e075755. doi: 10.1136/bmjopen-2023-075755

FUNDED BY NIHR | National Institute for Health and Care Research LIVERPOOL CLINICAL TRIALS CENTRE LCTC ICR

		Australia: 3.25 t.km<br>Spain: 0.28 t.km<br><br>CRFs<br>Postage of CRF booklets to sites:<br>UK sites, total t.km = 4.41 t.km<br>Australia sites, total t.km = 102.2 t.km<br>Spain sites, total t.km= 8.58963 t.km<br><br>Postage of CRFs from sites to CTU:<br>UK sites, total t.km = 6.37<br>Australia sites, total t.km = 132.9<br>Spain sites, total t.km = 13.8	0.18079 $kgCO_2e$ x 3.91666667 hours = 0.71 $kgCO_2e$<br><br>Postage of scans:<br>UK: 0.26115 t.km x 0.19443 = 0.05 $kgCO_2e$<br>Australia: 3.25 t.km x 1.13047 = 3.7 $kgCO_2e$<br>Spain: 0.28 t.km x 2.55439 = 0.72 $kgCO_2e$<br><br>CRFs<br>Postage of CRF booklets to sites:<br>UK: 4.41 t.km x 0.19443 = 0.86 $kgCO_2e$<br>Australia: 102.2 t.km x 1.13047 = 115.5 $kgCO_2e$<br>Spain: 8.58963 t.km x 2.55439 = 21.9 $kgCO_2e$<br><br>Postage of CRFs from sites to CTU:<br>UK: 6.37 t.km x 0.19443 = 1.2 $kgCO_2e$<br>Australia: 132.9 t.km x 1.13047 = 150.2 $kgCO_2e$<br>Spain: 13.8 t.km x 2.55439 = 35.3 $kgCO_2e$	
5.2. Data sent direct from participants to CTU or participating sites	E.g. Questionnaires, patient diaries, wearables	Estimated weight and distance of delivery (t.km):<br><br>Device used and time taken to complete electronic questionnaires:	For paper questionnaires, please refer to section 1.1. for the carbon footprint of producing the materials and section 1.2. for postage (freight).<br><br>For use of smart watches and other devices see section 6.3.<br><br>**Electronic questionnaires**<br>Add the emissions attributed to data storage and transmission to the emissions attributed to using a device to complete the questionnaire.	N/A

Griffiths J, *et al. BMJ Open* 2024; 14:e075755. doi: 10.1136/bmjopen-2023-075755

FUNDED BY NIHR | National Institute for Health and Care Research
LIVERPOOL CLINICAL TRIALS CENTRE LCTC
ICR

			Web surfing (data storage and transmission) = 9.441 g CO2e/hr (10 mins = 1.57 g CO_2e)<br><br>Choose from the below:<br>- Desktop computer = 0.18079 kg CO_2e per hour<br>- Laptop = 0.028719 kg CO_2e per hour<br>- Tablet = 0.027397 kg CO_2e per hour<br>- Smartphone = 0.015068 kg CO_2e per hour	
5.3. Data from labs to CTU<br>5.4. Data from other collaborators to CTU	E.g. Laboratory patient results, data linkage	GB required for data storage/transmission:<br>Duration of data storage:<br><br>Total £ spent on computer services:	Data storage and transmission = estimate 1.365 kg CO_2e per GB per year.<br><br>For computer services such as data linkage:<br>£ x 0.149 = kgCO_2e	N/A
**6. Trial supplies and equipment**				
6.1. Equipment used by CTU	E.g. computers, laptops, printers, software	Total £ spent on office machinery and computers for trial: £1350	For any new office machinery and computers purchased specifically for trial:<br>£ x 0.387 = kgCO_2e<br><br>£1350 x 0.387 = 522.45 kgCO_2e	522.45 kgCO_2e
6.2. Equipment and supplies used by participating sites supplied by CTU	E.g. centrifuge, fridge, freezer	Estimated weight and distance of deliveries (t.km):	For the shipment of equipment to participating sites, please refer to section 1.2.<br><br>For the use of a centrifuge, please refer to section 9.2., for a fridge or freezer please refer to section 9.3.	N/A

Griffiths J, *et al. BMJ Open* 2024; 14:e075755. doi: 10.1136/bmjopen-2023-075755

FUNDED BY NIHR | National Institute for Health and Care Research

LIVERPOOL CLINICAL TRIALS CENTRE LCTC

ICR

6.3. Equipment and supplies provided to participants specifically for the trial	E.g. wearables, smartphone, tablet	Number of devices and duration of their usage:<br>Estimated weight and distance of deliveries (t.km):	**Smartphone =** 55 kgCO_2e from manufacture and add 5.5 kgCO_2e per year of usage.<br>**Tablet =** 119 kgCO_2e from manufacture and add 10 kgCO_2e per year of usage.<br><br>**Wearables/smart watch** = 30.1 kg CO_2e for manufacture and add 1.633 kg CO_2e per year of usage.<br><br>**To calculate the carbon footprint associated with shipment of the devices, please refer to section 1.2.**	N/A
**7. Trial specific patient assessments**				
7.1. Patient travel for study visits that are in addition to standard of care	E.g. Eligibility and screening assessments, trial-specific assessments and procedures	Number of patients: 47<br>On average patients were on trial 72 weeks.<br><br>Number of times patient were required to travel (in addition to standard of care): 880 total patient visits (496 total visits by patients receiving IMP, 384 total visits by patients receiving placebo)	Emissions associated with one patient visit to hospital (UK) = 5.8 kgCO_2e (this includes both the out and back journeys)<br><br>Emissions associated with one patient visit to GP surgery (UK) = 1.12 kgCO_2e (this includes both the out and back journeys)<br><br>5.8 kgCO_2e x 880 = 5104 kgCO_2e	5104 kgCO_2e
7.2. Materials and activities required for study assessments that are in addition to standard of care	E.g. Laboratory tests, imaging assessments, clinical activities relating to intervention for	On average patients were on trial 72 weeks. Within that time:<br>- CT scans per patient<br>- 754 total visits requiring consumables	Consumables = 0.30 kgCO_2e per patient per trial appointment where consumables (such as gloves) required<br><br>▪ 1 MRI = 24.7 kg CO_2e<br>▪ 1 CT scan = 9.2 kgCO_2e	4117.8 kgCO_2e

FUNDED BY NIHR | National Institute for Health and Care Research
LIVERPOOL CLINICAL TRIALS CENTRE LCTC
ICR

	example administering of study drug, biopsy.		▪ 1 hour in surgery = 53 kg CO_2e<br>▪ 1 low intensity (general ward) bed day = 37.9 kg CO_2e<br>▪ 1 high intensity (ICU) bed day = 103 kgCO_2e<br>▪ 15 sessions of breast radiotherapy = 5.7 kg CO_2e<br>▪ 20 sessions of prostate radiotherapy = 15.3 kg CO_2e<br><br>Blood tests:<br>▪ 82 g CO_2e for coagulation profile<br>▪ 116 g CO_2e for full blood examination<br>▪ 49 g CO_2e for arterial gas assessment<br>▪ 99 g CO_2e for urea and electrolyte assessment<br>▪ 0.5 g CO_2e for C-reactive protein<br><br>**Please note that the above figures for blood tests include the materials and consumables required for sample collection, phlebotomy and analysis, as well as power consumption by pathology analysers.**<br><br>**Trial calculations:**<br><br>CT scans: 47 x 9 x 9.2 kgCO_2e = 3891.6 kgCO_2e<br>Consumables: 0.30 kgCO_2e x 754 = 226.2 kgCO_2e	
7.3. Utilities required for study assessments	E.g. energy consumption per	Trial staff FTE (FTEs added up over whole trial duration):	1 FTE 1 year = 364.9 kgCO_2e	2076.6 kgCO_2e

Griffiths J, *et al. BMJ Open* 2024; 14:e075755. doi: 10.1136/bmjopen-2023-075755

FUNDED BY NIHR | National Institute for Health and Care Research
LIVERPOOL CLINICAL TRIALS CENTRE LCTC
ICR

that are in addition to standard of care	square metre of hospital space according to trial staff FTE, taking into account time required for CRF completion and study assessments, consent etc	UK – 0.85<br>Spain – 0.25<br>Australia – 0.23<br>Australia benchmark and emission factor data:<br>- Australian average hospital energy intensity = 1676 MJ = 465.55 kWh/m2.<br>- Energy intensity per employee $16.5m^2$ x 465.55 = 7681.6 kWh<br>-50:50 Electricity/gas split, therefore 3840.8 kWh electricity, 3840.8 kWh natural gas<br>- Australia hospital sites located in NSW and QLD where electricity emission factor is 0.81 $kgCO_2e$/kWh.<br>- Australian National Greenhouse Accounts Factors (dcceew.gov.au)<br>Emission factor for natural gas: Scope 1 = 51.3 $kgCO_2e$/GJ, scope 3 = 13.1 $kgCO_2e$/GJ	Multiply by the number of years and FTE applicable<br><br>UK: 364.9 $kgCO_2e$ x 0.85 = 310.2 $kgCO_2e$<br>Spain: 364.9 $kgCO_2e$ x 0.25 = 91.2 $kgCO_2e$<br>Australia: 3840.8 kWh x 0.81 x 0.23 (FTE) = 715.5 $kgCO_2e$<br><br>Heating: 1 FTE, 1 year = 685.7 $kgCO_2e$<br>Multiply by the number of years and FTE applicable<br>UK: 685.7 $kgCO_2e$ x 0.85 = 582.8 $kgCO_2e$<br>Spain: 685.7 $kgCO_2e$ x 0.25 = 171.4$kgCO_2e$<br>Australia: 3840.8 kWh = 13.82688 GJ<br>- (13.82688 GJ x 51.53 $kgCO_2e$/GJ) + (13.82688 GJ x 13.1 $kgCO_2e$/GJ)<br>- = 893.6 $kgCO_2e$<br>- x 0.23 (FTE) = 205.5 $kgCO_2e$	
**8. Samples**				
8.1. Materials involved	E.g. sample collection kit and packaging for shipment	Kg of material:<br>- Safety kit (average plastic): 3kg<br>- Cryotubes (PP): 6.88kg<br>- Falcon tubes (PP): 6.88kg<br>- PAXgene tube (PET): 6kg<br>- Sea airbags (PET): 1.31kg<br>- Labels (PET): 1.6kg	The emissions attributed to sample collection consumables for common blood tests are included in the blood tests listed in section 7.2.<br><br>To calculate the carbon footprint of other common materials, multiply the weight in kg by the relevant emission factor below to produce $kgCO_2e$.	989.2 $kgCO_2e$

FUNDED BY NIHR | National Institute for Health and Care Research   LIVERPOOL CLINICAL TRIALS CENTRE LCTC   ICR

		- CTAD tubes (glass): 3.6335 kg<br>- CPT (glass): 3.2kg<br>- Jiffy bags (paper): 6kg<br>- Paper: 1.53kg<br>- Dry ice: 85kg<br>- Sample cold storage box: 29 single use boxes	- Average plastics: 3.116<br>- Plastics (average film): 2.754<br>- Plastics (Average rigid): 3.277<br>- Plastics (PP): 3.105<br>- Plastics (PET): 4.032<br>- Glass: 1.403<br>- Paper: 0.919<br>- Board: 0.821<br>- Dry ice: **1.81kg CO_2e for 1 kg dry ice** produced/used<br>- Sample cold storage box (SU): 25.2 kgCO_2e per cold storage box<br><br>Safety kit: 3kg x 3.116 = 9.35 kgCO_2e<br>Cryotubes + falcon tubes: 13.76kg x 3.105 = 42.7 kgCO_2e<br>PAXgene, sea airbags and labels: 8.9 1kg x 4.032 = 35.9 kgCO_2e<br>CTAD+CPT: 6.8225 kg x 3.413 = 9.57 kgCO_2e<br>Jiffy bags: 6kg x 0.881 (paper and board mixed) = 5.29 kgCO_2e<br>Paper: 1.53kg x 0.919 = 1.4 kgCO_2e<br>Paper (printing): 1.53kg x 0.22438 = 0.34 kgCO_2e<br>Dry ice: 85kg x 1.81 = 153.85 kgCO_2e<br>Sample boxes: 29 boxes x 25.2 kgCO_2e = 730.8 kgCO_2e	
8.2. Movement of sample kit materials from manufacturer to CTU	E.g. shipment of blood tubes for sample kits to CTU	8.2. Movement of sample kit materials from manufacturer to CTU	Please refer to section 1.2. for freight and 4.1. for refrigerated or frozen freight.	941.3 kgCO_2e

Griffiths J, *et al. BMJ Open* 2024; 14:e075755. doi: 10.1136/bmjopen-2023-075755

FUNDED BY NIHR | National Institute for Health and Care Research

LIVERPOOL CLINICAL TRIALS CENTRE LCTC

ICR

8.3. Movement of sample kits from CTU/distributor to participating Sites<br>8.4. Movement of samples from participating sites or patients to central laboratory.		- T.km = 11.9<br>8.3. Movement of sample kits from CTU/distributor to participating Sites:<br>▪ UK sites, total t.km = 11.9 t.km<br>▪ Australia, total t.km = 19.8528 t.km<br>▪ Spain, total t.km = 10.2 t.km<br>8.4. Movement of samples from participating sites to laboratory.<br>▪ UK sites, total t.km = 32.7 t.km<br>▪ Australia, total t.km = 663.65 t.km<br>▪ Spain, total t.km = 51.486 t.km<br>Movement of samples between laboratories:<br>- 0.8676 t.km	Movement of sample kit materials from manufacturer to CTU:<br>- 11.9 t.km X 0.19443 = 2.31 $kgCO_2e$<br><br>Movement of sample kits from CTU/distributor to participating Sites:<br>▪ UK sites:11.9 t.km x 0.19443 = 2.3 $kgCO_2e$<br>▪ Australia: 19.8528 t.km x 1.13047 = 22.4 $kgCO_2e$<br>▪ Spain: 10.2 t.km x 2.55439 = 26 $kgCO_2e$<br><br>8.4. Movement of samples from participating sites to laboratory.<br>- UK sites: 32.7 t.km x 0.19443 = 6.4 $kgCO_2e$<br>- Australia: 663.65 t.km x 1.13047 = 750.2 $kgCO_2e$<br>- Spain: 51.486 t.km x 2.55439 = 131.5 $kgCO_2e$<br><br>Movement of samples between laboratories:<br>- 0.8676 t.km x 0.19443 = 0.17 $kgCO_2e$	
**9. Laboratory**				
9.1. Emissions attributed to lab utilities according to staff FTE	E.g. energy consumption per square metre of laboratory space according to trial staff FTE	Trial duration:<br><br>Trial staff FTE:	**Electricity:**<br>1747.2 $kgCO_2e$ per FTE per year<br>Multiply 1747.2 $kgCO_2e$ by the number of years and FTE applicable<br><br>**Heating:**<br>1376 $kgCO_2e$ per FTE per year	N/A - No central labs within the scope of this trial. This module refers to the

			Multiply 1376 $kgCO_2e$ by the number of years and FTE applicable	site laboratories where initial processing of samples and sample storage occurs.
9.2. Materials/equipment /consumables used in processing and analysis of samples	E.g. centrifuges, refrigerators	Centrifuges required for processing the following samples:<br><br>CTAD samples<br>- Samples centrifuged for 15 mins (0.25 hours)<br>- 310 watts<br>- 310 x 0.25 = 77.5 kWh<br>- 313 samples<br><br>CPT samples<br>- Samples centrifuged for 45 mins (0.583 hours)<br>- 310 Watts<br>- 310 x 0.583 = 180.73 kWh<br>- 261 samples	To avoid double counting, use of equipment will be included in lab staff FTE if calculated.<br><br>If the trial does not involve a central lab, but there is still sample processing on site, please see below. For storage of samples, please see section 9.3.<br><br>To calculate the emissions of a piece of equipment, multiply the power consumption by hours used to get a kWh value. Finally multiply kWh by the electricity emission factor (0.273).<br><br>Consider the centrifuge capacity and multiply by the number of uses required.<br><br>Centrifuge – CTAD Samples:<br>- 77.5 kWh x 0.273 = 21.2 $kgCO_2e$ / 24 = 0.881$kgCO_2e$ per sample (Assumption: centrifuge capacity is 24 samples)<br>- 0.881 x 313 = 275.9 $kgCO_2e$<br><br>Centrifuge – CPT samples:	812.5 $kgCO_2e$

Griffiths J, *et al. BMJ Open* 2024; 14:e075755. doi: 10.1136/bmjopen-2023-075755

FUNDED BY NIHR | National Institute for Health and Care Research   LIVERPOOL CLINICAL TRIALS CENTRE LCTC   ICR

			- 180.73 kWh x 0.273 = 49.33929 $kgCO_2e$ / 24 = 2.1 $kgCO_2e$ per sample (Assumption: centrifuge capacity is 24 samples)<br>- 2.1 x 261 samples = 536.6 $kgCO_2e$<br><br>Total = 275.9 kgCO2e + 536.6 $kgCO_2e$ = 812.5 $kgCO_2e$	
9.3. Storage of samples	E.g. utilities and ultra-low temperature freezer	Storage of blood samples in fridge/freezer:<br>Length of storage = 10 years<br>Amount of refrigerator/freezer space required = 1/5 of a fridge, 1/3 of a freezer<br><br>Storage of ambient tissue:<br>- 10 years, 2011-2021<br>- Assumption: tissue samples occupy $1m^2$	Storage in fridge/-20 freezer:<br>- 298.9 $kgCO_2e$ per year<br>- Multiply by number of years stored<br>Storage in an ultra-low/-80 freezer:<br>- 2192.2 $kgCO_2e$ per year<br>- Multiply by number of years stored<br><br>NB: this is for a whole freezer; you will need to make an assumption about the amount of space in the freezer that the trial samples take up.<br><br>Fridge/-20:<br>298.9 $kgCO_2e$ x 10 years = 2989 $kgCO_2e$<br>2989 $kgCO_2e$ x 0.20 = 597.8 $kgCO_2e$<br><br>Storage in an ultra-low/-80 freezer:<br>2192.2 $kgCO_2e$ per year x 10 = 21922 $kgCO_2e$<br>21922 $kgCO_2e$ x 0.333 = 7307.3 $kgCO_2e$<br><br>Ambient storage of tissue:<br>Electricity:<br>- Health building = 22.1 $kgCO_2e/m^2$<br>- 22.1 $kgCO_2e/m^2$ x 10 = 221 $kgCO_2e$	8541.1 $kgCO_2e$

Griffiths J, *et al. BMJ Open* 2024; 14:e075755. doi: 10.1136/bmjopen-2023-075755

FUNDED BY NIHR | National Institute for Health and Care Research · LIVERPOOL CLINICAL TRIALS CENTRE LCTC · ICR

			Heating:<br>41.5 $kgCO_2e$ x 10 = 415 $kgCO_2e$	
**10. Trial close out**				
10.1. Storage and archiving of essential trial documentation and data	E.g. Hospital files, lab files, trial guidance documents etc	Duration of storage: 10 years<br><br>Amount of space required for storage (m^2): Assumption: archiving documents requires $4m^2$<br><br>Storage location: CTU (office)	Carbon footprint associated with $1m^2$ for 1 year:<br>- Office: 16.1 $kgCO_2e$<br>- Laboratory: 43.7 $kgCO_2e$<br>- Warehouse: 7.4 $kgCO_2e$<br>- Health building: 22.1 $kgCO_2e$<br><br>Choose the most suitable building type and multiply by number of years and m^2 necessary.<br><br>16.1 $kgC0_2e$ x 4 x 10 = 644 $kgCO_2e$<br><br>**Heating**<br>Carbon footprint associated with 1 m^2 for 1 year:<br><br>- Office: 35.69 $kgCO_2e$<br>- Laboratory: 34.4 $kgCO_2e$<br>- Warehouse: 13.3 $kgCO_2e$<br>- Health building: 41.5 $kgCO_2e$<br><br>Choose the most suitable building type and multiply by number of years and m^2 necessary.<br><br>35.69 $kgCO_2e$ x 4 x 10 = 1427.6 $kgCO_2e$	2085.3 $kgCO_2e$

Griffiths J, *et al. BMJ Open* 2024; 14:e075755. doi: 10.1136/bmjopen-2023-075755

			**For electronic storage,** estimate 1.35 $kgCO_2e$ per GB per year.<br><br>Size of CASPS on server = ~1GB<br>1.365 $kgCO_2e$ x 10 = 13.65 $kgCO_2e$	
10.2. Storage and destruction of biological samples	E.g. blood, tissue, urine etc	Duration of storage: 10 years<br><br>Amount of space required for storage (m^2): 1<br><br>Storage location: hospital/health building	See section 9.3. for storage of refrigerated or frozen samples.<br><br>See section 10.1 for storage of ambient samples.<br><br>Health building<br>Electricity: 22.1 $kgCO_2e$ x 10 = 221 $kgCO_2e$<br>Heating: 41.5 $kgCO_2e$ x 10 = 415 $kgCO_2e$	636 $kgCO_2e$
10.3. Return of equipment and supplies from participating sites to CTU	E.g. wearables, unused or expired equipment and supplies	Estimated weight and distance of delivery (t.km):	See section 1.2. (freight).	N/A

Griffiths J, *et al. BMJ Open* 2024; 14:e075755. doi: 10.1136/bmjopen-2023-075755

FUNDED BY NIHR | National Institute for Health and Care Research

LIVERPOOL CLINICAL TRIALS CENTRE LCTC

ICR

CASPS carbon footprint summary

**Module**	**$KgCO_2e$**
Trial set up	349.8
CTU emissions	23487.6
Trial staff meetings and travel	20465.3
Treatment intervention	1251.5
Data collection and exchange	558.3
Trial supplies and equipment	522.5
Trial specific patient assessments	11298.4
Samples	1930.5
Laboratory	9353.6
Analysis and trial close out	2721.3
**Total =**	71938.8 $kgCO_2e$ / 71.9 $tonnesCO_2e$

References

[1] What is an emission factor? [Internet]. Climfoot-project.eu. [cited 2023 May 11]. Available from: https://climfoot-project.eu/en/what-emission-factor

For all emission factor and benchmark data sources, please refer to the accompanying "Detailed Guidance and method to calculate the carbon footprint of a clinical trial."

Griffiths J, *et al. BMJ Open* 2024; 14:e075755. doi: 10.1136/bmjopen-2023-075755
