# Supplementary material for: Quantifying the carbon footprint of clinical trials: guidance development and case studies
Source: BMJ Open. 2024 Jan 24;14(1):e075755. doi: 10.1136/bmjopen-2023-075755 (PMC10823997; doi:10.1136/bmjopen-2023-075755)
Supplement: Supplementary data [file bmjopen-2023-075755supp004.pdf]

FUNDED BY NIHR | National Institute for Health and Care Research

Enabling lower carbon clinical trials: Development and prototype testing of a method to quantify the carbon footprint of clinical trials to inform future lower carbon clinical trial design

Guidance and method to calculate the carbon footprint of a clinical trial

Data collation quick guide and worksheet

This guidance provides information on how to carbon footprint a clinical trial for the purposes of the NIHR-funded project 'enabling lower carbon clinical trials.'

Within the guidance, clinical trial processes have been sub-divided into the following modules:

1. Trial set up
2. CTU emissions
3. Trial specific meetings and travel
4. Treatment intervention
5. Data collection and exchange
6. Trial supplies and equipment
7. Trial specific patient assessments
8. Samples
9. Laboratory
10. Trial close out

This list is not exhaustive, and it is expected that further activities and modules may need to be added to account for specialist processes in all clinical trial types.

NB: analysis of data does not need to be calculated separately, it is covered by the emissions attributed to trial staff FTE in "CTU emissions" and calculations included within "Data Collection and exchange".

Griffiths J, *et al. BMJ Open* 2024; 14:e075755. doi: 10.1136/bmjopen-2023-075755

FUNDED BY NIHR | National Institute for Health and Care Research
LIVERPOOL CLINICAL TRIALS CENTRE LCTC
ICR

In addition to this quick guide and worksheet, we have produced a detailed guidance and method document defining the project scope, limitations and assumptions. The detailed guidance contains a more in depth look and explanation of the calculations found in this document, including emission factor and benchmark data sources, and should be referred to when using this worksheet.

Introduction to calculating carbon footprint

A carbon footprint is a measure of greenhouse gases, usually quoted in kg or tonnes of carbon dioxide equivalent (CO_2e). To calculate the carbon footprint of a particular clinical trial process, both 'activity data' and 'emission factors' are required.

An emission factor, also known as a conversion factor, "is a coefficient which allows you to convert activity data into greenhouse gas emissions. It is the average **emission** rate of a given source, relative to units of activity or process/processes."[1]

The activity data is provided by the user and multiplied by the emission factors provided in this guidance document.

Data collation quick guide and worksheet

This data collation quick guide should be used in conjunction with the "Enabling lower carbon clinical trials: Development and prototype testing of a method to quantify the carbon footprint of clinical trials to inform future lower carbon clinical trial design - Detailed Guidance and method to calculate the carbon footprint of a clinical trial". The guidance document provides the detailed explanation of how calculations should be considered and calculated. This quick guide should be used to collate the trial-specific processes, necessary activity data and to record the subsequent calculations. It is important to avoid double-counting activities i.e., modules must not include activities already covered elsewhere in the clinical trial process map. Please complete this worksheet for each trial to be carbon footprinted.

NB: We are using the term 'CTU' to describe the organisation that manages all aspects of central trial management. For some institutions some of those tasks maybe done by groups outside the CTU team e.g., sponsor office/CRO etc.

FUNDED BY NIHR | National Institute for Health and Care Research — LIVERPOOL CLINICAL TRIALS CENTRE LCTC — ICR

PRIMETIME carbon footprint calculations

PRIMETIME is a post-operative avoidance of radiotherapy trial which recruited 1962 patients across 64 sites in the UK. The trial used electronic data capture for collection of data, data linkage with NHS routine data sources and involved shipment of tissue samples for immunohistochemistry testing and subsequent long-term sample storage.

Module	Examples	Trial activity data	Calculation	Results
**1. Trial set-up**				
1.1. Production of trial documentation to be sent to sites or participants	E.g. Site Investigator File and contents, Site Pharmacy File and contents, CRF Folder and contents, PIS/Cs, GP letters	67 sites initially set up (64 recruited patients)<br><br>3123 patients registered to pre-screening, 1962 patients consented and registered to main study<br><br>SIF: 260 pages, 67 sent (17420 total pages)<br>GP letter: 2 pages, 1962 (3924 total pages)<br>PIS/C form 1: 6 pages, 3123 patients (18738 total pages)<br>PIS/C form 2: 15 pages, 1962 (29430 total pages)<br><br>Number of folders used to send trial documentation = 67 large ring binders (0.5kg)	**Paper:**<br>[no. of page] x 0.005 = paper weight (kg)<br><br>b/w printing: Kg of paper x 0.22438 = $kgCO_2e$<br>Colour printing: Kg of paper x 0.31786 = $kgCO_2e$<br>Materials (paper): Kg of paper x 0.919 = $kgCO_2e$<br><br>Folders: Kg (of cardboard) x 0.821 = $kgCO_2e$<br>Assumption: Weight of lever arch = 0.5kg<br>Assumption: Weight of ring binder = 0.3kg<br><br>SIF:<br>17420 pages x 0.005= 87.1 kg<br>Printing: 87.1 kg x 0.22438 = 19.5 $KgCO_2e$<br>Materials (paper): 87.1 kg x 0.919 = 80 $kgCO_2e$<br><br>Materials (folder): 0.5 kg x 67= 33.5kg<br>33.5 kg x 0.821 = 27.5 $kgCO_2e$<br><br>PIS/C:<br>48168 x 0.005 kg = 240.8 kg<br>Printing: 240.8 kg x 0.31786 = 76.5 $kgCO_2e$<br>Materials: 240.8 kg x 0.919 = 221.3 $kgCO_2e$	447.2 kg CO_2e

Griffiths J, *et al. BMJ Open* 2024; 14:e075755. doi: 10.1136/bmjopen-2023-075755

FUNDED BY NIHR | National Institute for Health and Care Research
LIVERPOOL CLINICAL TRIALS CENTRE LCTC
ICR

			GP letter:<br>3924 x 0.005 kg = 19.6 kg<br>Printing: 19.6 kg x 0.22438 = 4.4 $kgCO_2e$<br>Materials: 19.6 kg x 0.919 = 18 $kgCO_2e$	
1.2. Provision/postage of trial documentation to sites<br><br>1.3. Provision/postage of documentation to participants by CTU or participating sites<br><br>1.4. Provision/postage of incentives to participant	E.g. Site Investigator File and contents, Site Pharmacy File and contents, CRF Folder and contents, PIS/Cs, GP letters	Estimated weight of SIF:<br>(260 pages x 0.005kg) + 0.5 kg = 1.8kg / 0.00177 tonnes<br><br>Estimated distance of deliveries:<br>Average site distance from ICR-CTSU is 301.8 km<br><br>Number of deliveries: SIF delivered to 67 sites	Delivery weight (tonnes) x distance (km) = t.km<br><br>For road freight: t.km x 0.19443 = $kgCO_2e$<br><br>For air freight: t.km x required emission factor below = $kgCO_2e$<br><br>- Domestic (to/from UK) = 4.98549<br>- Short-haul (to/from UK) = 2.55439<br>- Long-haul (to/from UK) = 1.13047<br>- International (to/from non-UK) = 1.13047<br><br>For delivery of trial supplies to patients or GP, if unknown, use 17.4km as distance from hospital to patient, or hospital to GP.<br><br>Calculations:<br>SIF delivery:<br>- 0.00177 x 301.8km = 0.53 t.km<br>- 0.53 x 0.19443 = 0.103<br>- x 67 sites = 6.9 $kgCO_2e$<br><br>GP letter:<br>Weight of letter = 2x5g + envelope (7g)= 17g	7.01 $kgCO_2e$

Griffiths J, *et al. BMJ Open* 2024; 14:e075755. doi: 10.1136/bmjopen-2023-075755

FUNDED BY NIHR | National Institute for Health and Care Research

LIVERPOOL CLINICAL TRIALS CENTRE LCTC

ICR

			x1962 patients = 33354g/0.033 tonnes<br>0.033 x 17.4km = 0.5742 t.km<br>0.5742 t.km x 0.19443 = 0.11 kgCO_2e	
**2. CTU emissions**				
2.1. Energy consumption at CTU according to trial staff FTE	E.g. energy consumption per square metre of air-conditioned office space	Trial duration: 10 years<br><br>Trial staff FTE: the total FTE over the 10-year trial period was 7.1	**Energy consumption for 1 FTE for 1 year** = 193.28 kgCO_2e<br><br>Multiply by the number of years and FTE applicable<br><br>7.1 X 193.28 kgCO_2e = 1372.3 kgCO_2e	1372.3 kgCO_2e
2.2. Heating	E.g. energy consumption at coordination centre attributed to heating (natural gas)	Trial duration: 10 years<br><br>Trial staff FTE: the total FTE over the 10-year trial period was 7.1	**Heating for 1 FTE for 1 year =** 431.56 kgCO_2e<br><br>Multiply by the number of years and FTE applicable<br><br>7.1 x 431.56 kgCO_2e = 3064 kgCO_2e	3064 kgCO_2e
2.3. Trial team commuting	E.g. Car, rail, bus, walking etc	Trial duration: 10 years<br><br>Trial staff FTE: the total FTE over the 10-year trial period was 7.1	For 1 FTE for 1 year, total average commuting emissions = 1027.8 kgCO_2e<br><br>Multiply by the number of years and FTE applicable<br><br>7.1 x 1027.8 kgCO_2e = 7297 kgCO_2e	7297 kgCO_2e
**3. Trial specific meetings and travel**				
3.1. Visits and travel to site<br>3.2. Travel to meetings	E.g. Feasibility, site initiation and monitoring visits,	No feasibility visits, monitoring, audits, or conferences.	Number of passengers x total distance (km) = p.km	579.3 kgCO_2e

Griffiths J, *et al. BMJ Open* 2024; 14:e075755. doi: 10.1136/bmjopen-2023-075755

FUNDED BY NIHR | National Institute for Health and Care Research
LIVERPOOL CLINICAL TRIALS CENTRE LCTC
ICR

	audits, inspections, Trial Management Group (TMG), Trial Steering Committee (TSC), Independent Data Monitoring Committee (IDMC), and investigator meetings, Patient and Public Involvement and Engagement (PPIE), conferences, scientific meetings etc	Site initiation visits<br>66 virtual (+6 refresher sessions), 1 in person<br><br>IDMC meetings<br>5 IDMC meetings, all virtual, total of 31 instances of attendance<br><br>TMG meetings<br>Total distances travelled by participants or number of virtual attendees:<br>- Meeting 1- 1760.6km<br>- Meeting 2- 1437.3km<br>- Meeting 3 – virtual, 12 attendees<br>- Meeting 4 – 1624.8km<br>- Meeting 5 – virtual, 25 attendees<br>- Meeting 6 – virtual, 18 attendees<br>- Meeting 7 – virtual, 23 attendees<br><br>TSC meetings<br>5 attendees to each TSC meeting, 1 hour duration, some teleconference and some in person. Distance staff travelled from CTU = 22.5km	For national rail: p.km x 0.04441= kgCO_2e<br><br>For flights: p.km x relevant emission factor below:<br>- Domestic (average): 0.27278<br>- Short-haul (average) to/from UK: 0.17034<br>- Long-haul (average) to/from UK: 0.21423<br>- International (average) to/from non-UK: 0.20373<br><br>NB: Distances may be calculated using google maps and calculated from CTU to destination<br><br>**Videoconferencing** = 157 grams CO_2e per hour.<br><br>Calculations:<br>Site Initiation Visits:<br>Virtual SIVs: 72 X 157 g CO2e/hr = 11304g/11.3 kgCO_2e<br><br>1 in person SIV: 1 attendee, 24.1KM.<br>1 x 2 x 24.1 km = 48.2 p.km<br>48.2 p.km x 0.04441= 2.1 kgCO_2e<br><br>IDMC<br>IDMC: 31 x 157 g CO2e/hr = 4867 g/4.9 kgCO_2e<br><br>TMG meetings - assumption: rail used to travel	

Griffiths J, *et al. BMJ Open* 2024; 14:e075755. doi: 10.1136/bmjopen-2023-075755

FUNDED BY NIHR | National Institute for Health and Care Research — LIVERPOOL CLINICAL TRIALS CENTRE LCTC — ICR

			TMG meeting 1: 1x2x1760.6km = 3521.2 p.km.<br>3521.2 p.km x 0.04441 = 156.4 $kgCO_2e$<br>TMG meeting 2: 1x2x1437.3km= 2874.6 p.km<br>2874.6 p.km x 0.04441 = 127.7 $kgCO_2e$<br>TMG meeting 3: teleconference, 2 hours, 12 external attendees. 157 g CO2e/hr x 2 x 12 = 3768g/3.77 $kgCO_2e$<br>TMG meeting 4: 1 x 2 x 1624.8km =<br>3249.6 p.km x 0.04441 = 144.3 $kgCO_2e$<br>TMG meeting 5: 13-14:30 via zoom, 25 attendees. 157 g CO2e/hr x 1.5 x 25 = 5887.5 g/5.89 $kgCO_2e$<br>TMG meeting 6: 157 g CO2e/hr x 1.5 x 18 = 4239 g/4.24 $kgCO_2e$<br>TMG meeting 7: 1hr, Teams meeting, 23 attendees - 157 g CO2e/hr x 23 = 3611g/3.6 $kgCO_2e$<br><br><u>TSC</u><br>2016, teleconference: 157 g CO2e/hr x 5 = 785g/0.8 $kgCO_2e$<br>2017: 5x(2x22.5km) = 225 p.km x 0.04441= 10 $kgCO_2e$<br>2018 = 10 $kgCO_2e$<br>2019 = 10 $kgCO_2e$<br>2021 = 10 $kgCO_2e$<br><br>Other:<br>➢ 2 virtual conferences: 157g CO2e/hr x 2 = 0.314 $kgCO_2e$	

Griffiths J, *et al. BMJ Open* 2024; 14:e075755. doi: 10.1136/bmjopen-2023-075755

FUNDED BY NIHR | National Institute for Health and Care Research

			➢ Public Health England (PHE) teleconference, 2 attendees: 2 x 157gCO_2e/hr = 0.314 kgCO_2e<br>➢ Sample management and logistics planning meeting, 5 attendees: 5 x 157 g CO2e/hr = 0.785 kgCO_2e<br>➢ Data management meetings, 20 instances of teleconferencing: 20 x 157 g CO2e/hr = 3140g/3.14 kgCO_2e<br>➢ LCRN Meeting re CPMS Uploads, 9 attendees - 9 x 157 g CO2e/hr = 1.413 kgCO_2e<br>➢ Primetime Team meetings, 132 instances of people teleconferencing for 1 hour. 132 x 157 = 20724 g/20.7 kgCO_2e<br>➢ CI_TC updates, 303 instances of people teleconferencing for 1 hour: 303 x 157 g CO2e/hr = 47571 g/47.6 kgCO_2e	
3.3. Hotel stays	E.g. TMG meetings, monitoring visits, audits, inspections etc	Number of rooms: 8 rooms (for one person for one night)	For UK: number of hotel rooms x number of nights x 13.9 = kgCO_2e<br><br>For UK (London) = number of rooms x number of nights x 13.8 = kgCO_2e.<br><br>For other countries use conversion factors from orange 'hotel stay' tab: conversion-factors-2021-full-set-advanced-users.xlsm (live.com)<br>Alternatively, you may use cost-based method:<br>£ spent x 0.388 = kgCO_2e	250.2 kgCO_2e

Griffiths J, *et al. BMJ Open* 2024; 14:e075755. doi: 10.1136/bmjopen-2023-075755

			Assumption: 8 stays (for TMG meetings)<br>8 x 13.9 = 250.2 $kgCO_2e$	
3.4. Sustenance	E.g. meeting lunches, hotel dinners	Number of lunches or dinners:<br>3 in-person TMG meetings, 52 total attendees<br>4 in person TSC meetings, 20 total attendees<br><br>Total = 72 lunches for TMG and TSC meeting participants	Meeting lunches or hotel dinners (vegetarian) = 2.6 $kgCO_2e$ per meal per person<br><br>Meeting lunches or hotel dinners (meat) = 5.92 $kgCO_2e$ per meal per person<br><br>Assumption: unable to determine if meat or vegetarian therefore assumed 50/50<br>(36 x 2.6) + (36 x 5.92) = 306.7 $kgCO_2e$	306.7 $kgCO_2e$
**4. Intervention*** **4.1. Physical (IMP)**<br>**4.2. Clinical (non-IMP)**<br>**4.3. Other (not captured above)**<br>**Please fill out the section most relevant to the intervention being investigated.**				
**4.1. Physical**				
4.1.1, 4.1.2. Movement of intervention, or materials required to deliver the intervention	E.g. movement of intervention from manufacturing site to distribution site, shipment of IMP to participating sites or direct to participant	Total weight of deliveries:<br><br>Total distance of deliveries:	Carry out freight calculation as described in section 1.2.<br><br>For refrigerated freight, increase the total $kgCO_2e$ associated with freight by 15%.<br><br>Frozen freight:	N/A – Avoidance of radiotherapy trial

* As per assumptions detailed in the guidance, manufacture of the intervention is considered out of scope. This section defines all processes relating to providing and delivering the trial intervention that are over and above routine care.

Griffiths J, *et al. BMJ Open* 2024; 14:e075755. doi: 10.1136/bmjopen-2023-075755

FUNDED BY NIHR | National Institute for Health and Care Research

LIVERPOOL CLINICAL TRIALS CENTRE LCTC

ICR

			Dry ice has a carbon footprint of **1.81kg CO_2e for 1 kg dry ice** produced/used.<br><br>When calculating the overall emissions of frozen freight, as well as the 1.81 kgCO_2e per 1kg attributed to manufacture, include the weight (kg) of dry ice used in the weight of the freight calculation in section 1.2.<br><br>In the absence of activity data, assume 1kg of dry ice is used per individual sample shipping box.	
4.1.3. Materials required for the packaging and shipment of IMP	E.g. cardboard, cold storage boxes, polystyrene	Number of cold storage boxes:<br><br>Kg of cardboard/ polystyrene:	Single use sample cold storage box = 25.2 kgCO_2e per box<br><br>Reusable sample cold storage box = 2.2 kgCO_2e per box<br><br>Kg (cardboard) x 0.821 = kgCO_2e<br>Kg (polystyrene) x 3.778 = kgCO_2e	N/A – avoidance of radiotherapy trial
4.1.4. Destruction of overage	E.g. incineration of IMP	Estimated weight of overage incinerated:	Kg of waste x 2.4252 = kgCO_2e	N/A – avoidance of radiotherapy trial
**4.2. Clinical** e.g., radiotherapy, device, surgical.<br>NB: not all calculations will be relevant to all interventions. **This section of the method will be further developed as we carbon footprint more trials, so please inform us if your protocol specifies an activity that has not been included, and we will help to determine the associated carbon footprint.**				
4.2.1 Movement of the intervention, or resources	E.g. movement of intervention from	Estimated total weight of delivery:	Please refer to section 1.2 and 4.1.1, 4.1.2.	N/A – avoidance of

Griffiths J, *et al. BMJ Open* 2024; 14:e075755. doi: 10.1136/bmjopen-2023-075755

required to deliver the intervention	manufacturing site to distribution site, shipment of intervention to participating sites.	Estimated total distance of deliveries:		radiotherapy trial
4.2.2 Materials required for the packaging and shipment of the intervention	E.g. cardboard, cold storage boxes, polystyrene	Number of cold storage boxes:<br><br>Kg of cardboard/ polystyrene:	Please refer to section 4.1.3.	N/A – avoidance of radiotherapy trial
4.2.3 Utilities required for delivery of the intervention	E.g. Hospital utilities if the intervention is delivered within a hospital	Hospital staff FTE required:	Please refer to section 7.3 to calculate the emissions attributed to hospital utilities if required to deliver the intervention.	N/A – avoidance of radiotherapy trial
4.2.4 Activities or resources required/relating to delivery of the intervention	E.g. consumables, surgical time, specialist equipment, incineration of surgical waste		Please refer to section 7.2 for consumables, surgery and other activities that may be relevant to the delivery of the intervention, but please take care to avoid double counting.<br><br>To calculate the emissions attributed to incineration, e.g. of surgical waste, please refer to section 4.1.4.	N/A – avoidance of radiotherapy trial
**4.3. Other**<br>NB: not all calculations will be relevant to all interventions. **This section of the method will be further developed as we carbon footprint more trials, so please inform us if your protocol specifies an activity that has not been included, and we will help to determine the associated carbon footprint.**				
4.3.1 Movement of the intervention to the participant or participating site	E.g. shipment of intervention to participating sites or direct to participant	Estimated weight of delivery:<br><br>Estimated distance of deliveries:	Please refer to section 1.2.	N/A – avoidance of radiotherapy trial
4.3.2 Materials required for packaging and	E.g. cardboard, cold storage boxes, polystyrene	Number of cold storage boxes:<br><br>Kg of cardboard/ polystyrene:	Please refer to section 4.1.3.	N/A – avoidance of

Griffiths J, et al. BMJ Open 2024; 14:e075755. doi: 10.1136/bmjopen-2023-075755

FUNDED BY NIHR | National Institute for Health and Care Research

LIVERPOOL CLINICAL TRIALS CENTRE LCTC

ICR

shipment of the intervention				radiotherapy trial
4.3.3 Materials or resources required for delivery of the intervention	E.g. software, booklets, specialist equipment		For printing and paper, please refer to section 1.1.	N/A – avoidance of radiotherapy trial
4.3.4 Travel required to facilitate delivery of the intervention	E.g. to deliver training, conduct interviews etc	Estimated distance travelled, and number of passengers (p.km):	Please refer to section 3.1, 3.2.	N/A – avoidance of radiotherapy trial
**5. Data collection and exchange**<br>NB: analysis of data does not need to be calculated separately, it is covered by the emissions attributed to trial staff FTE in "CTU emissions" and calculations included within "Data Collection and exchange".				
5.1. Data collection and query exchange between CTU and sites	E.g. CRFs, EDC completion and query resolution, scans copied to CDs	Postage from sites to ICR-CTSU:<br>Only the eligibility, pre-screening and registration checklists posted to CTU from sites.<br>-Total pages from all sites = 14094.<br>-Average site distance from ICR-CTSU: 301.8 km<br><br>Printing by sites for their SIF:<br>Estimate 419 pages printed by each site for their SIF, 63 sites = 26397 total pages<br><br>Paper CRFs:<br>28 folders/box files containing paper CRFs in ICR-CTSU.	Postage of documents from sites:<br>14094 x 0.005 = 70.47 kg / 0.07 tonnes<br>0.07 x 301.8 = 21.126 t.km<br>21.126 t.km x 0.19443 = 4.1 kgCO_2e<br><br>Printing by sites for their SIF:<br>26397 x 0.005 = 131.985 kg<br>Printing: 131.985 kg x 0.22438 = 29.6 kgCO_2e<br>Material: 131.985 kg x 0.919 = 121.3 kgCO_2e<br><br>Paper CRFs<br>14000 x 0.005 = 70kg<br>Printing: 70 kg x 0.22438 = 15.7 kgCO_2e<br>Materials: 70kg x 0.919 = 64.3 kgCO_2e<br><br>Site documents stored in ICR-CTSU:<br>6300 x 0.005= 31.5 kg<br>Printing: 31.5 kg x 0.22438 = 7.1 kgCO_2e	271.1 kgCO_2e

Griffiths J, *et al. BMJ Open* 2024; 14:e075755. doi: 10.1136/bmjopen-2023-075755

FUNDED BY NIHR | National Institute for Health and Care Research

LIVERPOOL CLINICAL TRIALS CENTRE LCTC

ICR

		Assumption: box file contains 500 pieces of paper<br>500 x 28 = 14000 total pages<br><br>Site documents stored in ICR-CTSU:<br>18 folders with TMFs and site documents stored in CTU.<br>Assumption: folder holds 350 pages.<br>18 x 350 = 6300 total pages	Materials: 31.5 kg x 0.919 = 29 $kgCO_2e$	
5.2. Data sent direct from participants to CTU or participating sites	E.g. Questionnaires, patient diaries, wearables	Estimated weight and distance of delivery (t.km):<br><br>Device used and time taken to complete electronic questionnaires:	For paper questionnaires, please refer to section 1.1. for the carbon footprint of producing the materials and section 1.2. for postage (freight).<br><br>For use of smart watches and other devices see section 6.3.<br><br>**Electronic questionnaires**<br>Add the emissions attributed to data storage and transmission to the emissions attributed to using a device to complete the questionnaire.<br><br>Web surfing (data storage and transmission) = 9.441 g CO2e/hr (10 mins = 1.57 g CO_2e)<br><br>Choose from the below:<br>- Desktop computer = 0.18079 kg CO_2e per hour<br>- Laptop = 0.028719 kg CO_2e per hour	N/A

Griffiths J, *et al. BMJ Open* 2024; 14:e075755. doi: 10.1136/bmjopen-2023-075755

			- Tablet = 0.027397 kg CO_2e per hour<br>- Smartphone = 0.015068 kg CO_2e per hour	
5.3. Data from labs to CTU<br>5.4. Data from other collaborators to CTU	E.g. Laboratory patient results, data linkage	GB required for data storage/transmission:<br>Duration of data storage:<br><br>Total £ spent on computer services: £49000	Data storage and transmission = estimate 1.365 kg CO_2e per GB per year.<br><br>For computer services such as data linkage:<br>£ x 0.149 = kgCO_2e<br><br>£49000 x 0.149 = 7301 kgC0_2e	7301 kgC0_2e
**6. Trial supplies and equipment**				
6.1. Equipment used by CTU	E.g. computers, laptops, printers, software	Total £ spent on office machinery and computers for trial: £4500	For any new office machinery and computers purchased specifically for trial:<br>£ x 0.387 = kgCO_2e<br>£4500 x 0.387 = 1741.5 kgCO_2e	1741.5 kgCO_2e
6.2. Equipment and supplies used by participating sites supplied by CTU	E.g. centrifuge, fridge, freezer	Estimated weight and distance of deliveries (t.km):	For the shipment of equipment to participating sites, please refer to section 1.2.<br><br>For the use of a centrifuge, please refer to section 9.2., for a fridge or freezer please refer to section 9.3.	N/A
6.3. Equipment and supplies provided to participants specifically for the trial	E.g. wearables, smartphone, tablet	Number of devices and duration of their usage:<br><br>Estimated weight and distance of deliveries (t.km):	**Smartphone** = 55 kgCO_2e from manufacture and add 5.5 kgCO_2e per year of usage.	N/A

Griffiths J, *et al. BMJ Open* 2024; 14:e075755. doi: 10.1136/bmjopen-2023-075755

FUNDED BY NIHR | National Institute for Health and Care Research — LIVERPOOL CLINICAL TRIALS CENTRE LCTC — ICR

			**Tablet =** 119 $kgCO_2e$ from manufacture and add 10 $kgCO_2e$ per year of usage.<br><br>**Wearables/smart watch** = 30.1 kg CO_2e for manufacture and add 1.633 kg CO_2e per year of usage.<br><br>**To calculate the carbon footprint associated with shipment of the devices, please refer to section 1.2.**	
**7. Trial specific patient assessments**				
7.1. Patient travel for study visits that are in addition to standard of care	E.g. Eligibility and screening assessments, trial-specific assessments and procedures	Number of times patient is required to travel (in addition to standard of care): 1623 patients had to travel for 5 additional above standard of care mammograms	Emissions associated with one patient visit to hospital (UK) = 5.8 $kgCO_2e$ (this includes both the out and back journeys)<br>Emissions associated with one patient visit to GP surgery (UK) = 1.12 $kgCO_2e$ (this includes both the out and back journeys)<br><br>5.8 $kgCO_2e$ x 5 x 1623 = 47067 $kgCO_2e$	47067 $kgCO_2e$
7.2. Materials and activities required for study assessments that are in addition to standard of care	E.g. Laboratory tests, imaging assessments, clinical activities relating to intervention for example administering of study drug, biopsy.	5 above standard of care mammograms for each patient in very low risk and no RT group (1623)<br><br>Assumption: 2 visits where consumables required	Consumables = 0.30 $kgCO_2e$ per patient per trial appointment where consumables (such as gloves) required<br><br>- 1 MRI = 24.7 kg CO_2e<br>- 1 CT scan = 9.2 $kgCO_2e$<br>- 1 CXR = 0.8 $kgCO_2e$/scan<br>- 1 hour in surgery = 53 kg CO_2e	7977.3 $kgCO_2e$

Griffiths J, *et al. BMJ Open* 2024; 14:e075755. doi: 10.1136/bmjopen-2023-075755

FUNDED BY NIHR | National Institute for Health and Care Research — LIVERPOOL CLINICAL TRIALS CENTRE LCTC — ICR

			- 1 low intensity (general ward) bed day = 37.9 kg CO_2e<br>- 1 high intensity (ICU) bed day = 103 kgCO_2e<br>- 15 sessions of breast radiotherapy = 5.7 kg CO_2e<br>- 20 sessions of prostate radiotherapy = 15.3 kg CO_2e<br><br>Blood tests:<br>- 82 g CO_2e for coagulation profile<br>- 116 g CO_2e for full blood examination<br>- 49 g CO_2e for arterial gas assessment<br>- 99 g CO_2e for urea and electrolyte assessment<br>- 0.5 g CO_2e for C-reactive protein<br><br>**Please note that the above figures for blood tests include the materials and consumables required for sample collection, phlebotomy and analysis, as well as power consumption by pathology analysers.**<br><br><u>Calculations:</u><br>Consumables:<br>- 0.30 kgCO_2e x 2989 (number of patients who received ki67 testing) = 896.7 kgCO_2e<br>- 0.30 x 1962 (for physical appointment for those registered to study) = 588.6 kgCO_2e	

Griffiths J, *et al. BMJ Open* 2024; 14:e075755. doi: 10.1136/bmjopen-2023-075755

FUNDED BY NIHR | National Institute for Health and Care Research — LIVERPOOL CLINICAL TRIALS CENTRE LCTC — ICR

			- Total = 1485.3 $kgCO_2e$<br><br>Scans: 5 x 0.8 $kgCO_2e$ x 1623 = 6,492 $kgCO_2e$	
7.3. Utilities required for study assessments that are in addition to standard of care	E.g. energy consumption per square metre of hospital space according to trial staff FTE, taking into account time required for CRF completion and study assessments, consent etc	Hospital trial staff FTE: total estimated FTE = 5.5 from 2017 - 2022†	Electricity: 1 FTE 1 year = 364.9 $kgCO_2e$<br>Multiply by the number of years and FTE applicable<br><br>364.9 $kgCO_2e$ x 5.5 = 2007 $kgCO_2e$<br><br>Heating: 1 FTE, 1 year = 685.7 $kgCO_2e$<br>Multiply by the number of years and FTE applicable<br><br>685.7 $kgCO_2e$ x 5.5 = 3771.4 $kgCO_2e$	5778.4 $kgCO_2e$
**8. Samples**				
8.1. Materials involved	E.g. sample collection kit and packaging for shipment	Kg of material:<br>- 30.6 kg slide container (pp)<br>- 243.8 kg jiffy bag<br>- 39 kg paper	The emissions attributed to sample collection consumables for common blood tests are included in the blood tests listed in section 7.2.<br><br>To calculate the carbon footprint of other common materials, multiply the weight in kg by the relevant emission factor below to produce $kgCO_2e$.<br>- Average plastics: 3.116<br>- Plastics (average film): 2.754<br>- Plastics (Average rigid): 3.277<br>- Plastics (PP): 3.105	397.9 $kgCO_2e$

† Hospital trial staff FTE was estimated over 6 years rather than the 10-year trial duration because the final 4 years are follow up via routine data sources.

Griffiths J, *et al. BMJ Open* 2024; 14:e075755. doi: 10.1136/bmjopen-2023-075755

FUNDED BY NIHR | National Institute for Health and Care Research — LIVERPOOL CLINICAL TRIALS CENTRE LCTC — ICR

			- Plastics (PET): 4.032<br>- Glass: 3.413<br>- Paper: 0.919<br>- Board: 0.821<br><br>Slide containers = 30.6 kg x 3.105 (pp) = 95 $kgCO_2e$<br><br>Paper (materials) = 39kg x 0.919 = 35.8 $kgCO_2e$<br>Paper (printing) = 39kg x 0.22438 = 8.8 $kgCO_2e$<br><br>Jiffy bag = 243.8 kg x 0.919= 224.1 $kgCO_2e$<br><br>Tracking slip: 2 copies of each tracking slip on top of one included in the kit– 2989 x 2 = 5978 pages<br>Printing: 5978 pages x 5g = 29890g/29.89kg<br>29.89 kg x 0.22438 = 6.7 $kgCO_2e$<br>26.9kg x 0.919 = 27.5 $kgCO_2e$	
8.2. Movement of sample kit materials from manufacturer to CTU<br>8.3. Movement of sample kits from CTU/distributor to participating Sites<br>8.4. Movement of samples from participating sites or	E.g. shipment of blood tubes for sample kits to CTU	8.2. Movement of sample kit materials from manufacturer to CTU<br>Envelopes:<br>- Weight = 0.004 tonnes, total<br>- Total distance transported = 849.6km (141.6km x 6 deliveries)<br><br>Slide mailing containers:<br>-Weight = 0.00204 tonnes	Please refer to section 1.2. for freight and 4.1. for refrigerated or frozen freight.<br><br>**8.2: Movement of sample kit materials from manufacturer to CTU**<br><br>Envelopes:<br>0.004 tonnes x 849.6km = 3.3984 t.km<br>3.3984 t.km x 0.19443 = 0.66 $kgCO_2e$	40.26 $kgCO_2e$

Griffiths J, *et al. BMJ Open* 2024; 14:e075755. doi: 10.1136/bmjopen-2023-075755

FUNDED BY NIHR | National Institute for Health and Care Research — LIVERPOOL CLINICAL TRIALS CENTRE LCTC — ICR

patients to central laboratory.		-Total distance transported = 6910.3 km (363 km x 19 deliveries)<br><br>8.3. Movement of sample kits from CTU to participating Sites:<br>-Average distance from CTU to participating site = 301.8km<br>- Shipment weight = 0.00053 tonnes<br>- 630 sample kit shipments<br><br>8.4. Movement of samples from participating sites to laboratory.<br>-Cambridge lab – average distance to participating site = 239.5km, 938 shipments<br>-RMH lab - average distance to participating site = 258km, 1094 shipments<br>-Glasgow lab - average distance to participating site = 467.8km, 957 shipments	Slide mailing containers:<br>0.00204 tonnes x 6910.3 km = 14.097 t.km<br>14.097 t.km x 0.19443 = 2.7 kgCO_2e<br><br>**8.3. Movement of sample kits from CTU/distributor to participating Sites**<br><br>0.00053 x (630 x 295.88) = 100.77 t.km x 0.19443 = 19.6 kgCO_2e<br><br>**8.4. Movement of samples from participating sites or patients to central laboratory.**<br><br>Cambridge laboratory: 0.0000935 x (239.5km x 938) x 0.19443 = 4.1 kgCO_2e<br>RMH laboratory: 0.0000935 X (258 km X 1094) X 0.19443 = 5.1 kgCO_2e<br>Glasgow laboratory: 0.0000935 x (467.8km x 957) x 0.19443 = 8.1 kgCO_2e	
**9. Laboratory**				
9.1. Emissions attributed to lab utilities according to staff FTE	E.g. energy consumption per square metre of laboratory space	Trial staff FTE and trial duration: 3 labs, 1 staff member in each. Trial is 5% of each of their FTE for 4 years and 2% for 1 year.	**Electricity:**<br>1747.2 kgCO_2e per FTE per year<br>Multiply 1747.2 kgCO_2e by the number of years and FTE applicable	2061.4 kgCO_2e

Griffiths J, *et al. BMJ Open* 2024; 14:e075755. doi: 10.1136/bmjopen-2023-075755

	according to trial staff FTE		1747.2 $kgCO_2e$ x 0.05 = 87.36 $kgCO_2e$<br>87.36 $kgCO_2e$ x 4 years = 349.44 $kgCO_2e$<br>1747. 2 $kgCO_2e$ x 0.02 = 34.944 $kgCO_2e$<br>349.44 + 34.944 = 384.4 $kgCO_2e$<br>384.4 x 3 lab staff = 1153.2 $kgCO_2e$<br><br>**Heating:**<br>1376 $kgCO_2e$ per FTE per year<br>Multiply 1376 $kgCO_2e$ by the number of years and FTE applicable<br><br>1376 $kgCO_2e$ x 0.05 = 68.8 $kgCO_2e$<br>68.8 $kgCO_2e$ x 4 = 275.2 $kgCO_2e$<br>1376 x 0.02 $kgCO_2e$ = 27.52 $kgCO_2e$<br>275.2 $kgCO_2e$ + 27.52 $kgCO_2e$ = 302.72 $kgCO_2e$<br>Total = 302.72 $kgCO_2e$ x 3 = 908.16 $kgCO_2e$	
9.2. Materials/equipment /consumables used in processing and analysis of samples	E.g. centrifuges, refrigerators	kWh usage of equipment, hours used:<br><br>Kg of material:	To avoid double counting, use of equipment will be included in lab staff FTE if calculated.<br><br>If the trial does not involve a central lab, but there is still sample processing on site, please see below. For storage of samples, please see section 9.3.<br><br>To calculate the emissions of a piece of equipment, multiply the power consumption by hours used to get a kWh value. Finally multiply kWh by the electricity emission factor (0.273).	N/A

Griffiths J, *et al. BMJ Open* 2024; 14:e075755. doi: 10.1136/bmjopen-2023-075755

FUNDED BY NIHR | National Institute for Health and Care Research — LIVERPOOL CLINICAL TRIALS CENTRE LCTC — ICR

			Example: use of a 310-Watt centrifuge for 15 minutes<br>- 310 Watts x 0.25 (hours) = 77.5 kWh<br>- 77.5 kWh x 0.273 = 21.2 $kgco_2e$<br><br>Consider the centrifuge capacity and multiply by the number of uses required.	
9.3. Storage of samples	E.g. utilities and ultra-low temperature freezer	Length of storage:<br><br>Amount of refrigerator/freezer space required:	Storage in fridge/-20 freezer:<br>- 298.9 $kgCO_2e$ per year<br>- Multiply by number of years stored<br><br>Storage in an ultra-low/-80 freezer:<br>- 2192.2 $kgCO_2e$ per year<br>- Multiply by number of years stored<br><br>NB: this is for a whole freezer; you will need to make an assumption about the amount of space in the freezer that the trial samples take up.	N/A
**10. Trial close out**				
10.1. Storage and archiving of essential trial documentation and data	E.g. Hospital files, lab files, trial guidance documents etc	Duration of storage: 10 years<br><br>Amount of space required for storage (m^2): $4m^2$<br><br>Storage location: CTU (office)	Carbon footprint associated with $1m^2$ for 1 year:<br>- Office: 16.1 $kgC0_2e$<br>- Laboratory: 43.7 $kgc0_2e$<br>- Warehouse: 7.4 $kgC0_2e$<br>- Health building: 22.1 $kgCO_2e$<br><br>Choose the most suitable building type and multiply by number of years and m^2 necessary.<br><br>**Heating**	2126.6 $kgCO_2e$

Griffiths J, *et al. BMJ Open* 2024; 14:e075755. doi: 10.1136/bmjopen-2023-075755

FUNDED BY NIHR | National Institute for Health and Care Research
LIVERPOOL CLINICAL TRIALS CENTRE LCTC
ICR

			Carbon footprint associated with 1 m^2 for 1 year:<br><br>- Office: 36 $kgCO_2e$<br>- Laboratory: 34.4 $kgCO_2e$<br>- Warehouse: 13.3 $kgCO_2e$<br>- Health building: 41.6 $kgCO_2e$<br><br>Choose the most suitable building type and multiply by number of years and m^2 necessary.<br><br>**For electronic storage,** estimate 1.365 kg CO_2e per GB per year.<br><br>Calculations:<br>Electricity: 16.1 $kgCO_2e$ x 4 x 10 = 644 $kgCO_2e$<br><br>Heating: 36 $kgCO_2e$ x 4 x 10 years = 1428 $kgCO_2e$<br><br>Electronic storage:<br>PRIMETIME trial database = ~ 4GB<br>Assumption: storage for 10 years<br>1.365 kg CO_2e x 4 x 10 = 54.6 $kgCO_2e$	
10.2. Storage and destruction of biological samples	E.g. blood, tissue, urine etc	Duration of storage: 10 years<br><br>Amount of space required for storage (m^2): 2m^2<br><br>Storage location: Health building	See section 9.3. for storage of refrigerated or frozen samples.<br><br>See section 10.1 for storage of ambient samples.	1274 $kgCO_2e$

Griffiths J, *et al. BMJ Open* 2024; 14:e075755. doi: 10.1136/bmjopen-2023-075755

FUNDED BY NIHR | National Institute for Health and Care Research

			Electricity:<br>22.1 $kgCO_2e$ x2 x 10 = 442 $kgCO_2e$<br><br>Heating:<br>41.6 $kgCO_2e$ x 2 x 10 = 832 $kgCO_2e$	
10.3. Return of equipment and supplies from participating sites to CTU	E.g. wearables, unused or expired equipment and supplies	Estimated weight and distance of delivery (t.km):	See section 1.2. (freight).	N/A

PRIMETIME carbon footprint summary

Module	$KgCO_2e$
Trial set up	454.2
CTU emissions	11733.3
Trial staff meetings and travel	1136.2
Treatment intervention	0
Data collection and exchange	7572.1
Trial supplies and equipment	1741.5
Trial specific patient assessments	60822.7
Samples	438.2
Laboratory	2061.4

Griffiths J, *et al. BMJ Open* 2024; 14:e075755. doi: 10.1136/bmjopen-2023-075755

FUNDED BY NIHR | National Institute for Health and Care Research

Analysis and trial close out	3400.6
**Total =**	89360.2 kgCO_2e/ 89.4 tonnes

References

[1] What is an emission factor? [Internet]. Climfoot-project.eu. [cited 2023 May 11]. Available from: https://climfoot-project.eu/en/what-emission-factor

For all emission factor and benchmark data sources, please refer to the accompanying "Detailed Guidance and method to calculate the carbon footprint of a clinical trial."

Griffiths J, *et al. BMJ Open* 2024; 14:e075755. doi: 10.1136/bmjopen-2023-075755
