# Supplementary material for: Quantifying the carbon footprint of clinical trials: guidance development and case studies
Source: BMJ Open. 2024 Jan 24;14(1):e075755. doi: 10.1136/bmjopen-2023-075755 (PMC10823997; doi:10.1136/bmjopen-2023-075755)
Supplement: Author's manuscript [file bmjopen-2023-075755.draft_revisions.pdf]

BMJ Open

BMJ Open is committed to open peer review. As part of this commitment we make the peer review history of every article we publish publicly available.

When an article is published we post the peer reviewers' comments and the authors' responses online. We also post the versions of the paper that were used during peer review. These are the versions that the peer review comments apply to.

The versions of the paper that follow are the versions that were submitted during the peer review process. They are not the versions of record or the final published versions. They should not be cited or distributed as the published version of this manuscript.

BMJ Open is an open access journal and the full, final, typeset and author-corrected version of record of the manuscript is available on our site with no access controls, subscription charges or pay-per-view fees (http://bmjopen.bmj.com).

If you have any questions on BMJ Open's open peer review process please email info.bmjopen@bmj.com

BMJ Open

Quantifying the carbon footprint of clinical trials: guidance development and pilot study

Journal:	*BMJ Open*
Manuscript ID	bmjopen-2023-075755
Article Type:	Original research
Date Submitted by the Author:	18-May-2023
Complete List of Authors:	Griffiths, Jessica; The Institute of Cancer Research,<br>Fox, Lisa; The Institute of Cancer Research<br>Williamson, Paula; The University of Liverpool<br>Low Carbon Clinical Trials Group, group authorship; Sustainable Healthcare Coalition
Keywords:	Clinical Trial, Decision Making, STATISTICS & RESEARCH METHODS

SCHOLARONE™
Manuscripts

I, the Submitting Author has the right to grant and does grant on behalf of all authors of the Work (as defined in the below author licence), an exclusive licence and/or a non-exclusive licence for contributions from authors who are: i) UK Crown employees; ii) where BMJ has agreed a CC-BY licence shall apply, and/or iii) in accordance with the terms applicable for US Federal Government officers or employees acting as part of their official duties; on a worldwide, perpetual, irrevocable, royalty-free basis to BMJ Publishing Group Ltd ("BMJ") its licensees and where the relevant Journal is co-owned by BMJ to the co-owners of the Journal, to publish the Work in this journal and any other BMJ products and to exploit all rights, as set out in our licence.

The Submitting Author accepts and understands that any supply made under these terms is made by BMJ to the Submitting Author unless you are acting as an employee on behalf of your employer or a postgraduate student of an affiliated institution which is paying any applicable article publishing charge ("APC") for Open Access articles. Where the Submitting Author wishes to make the Work available on an Open Access basis (and intends to pay the relevant APC), the terms of reuse of such Open Access shall be governed by a Creative Commons licence – details of these licences and which Creative Commons licence will apply to this Work are set out in our licence referred to above.

Other than as permitted in any relevant BMJ Author's Self Archiving Policies, I confirm this Work has not been accepted for publication elsewhere, is not being considered for publication elsewhere and does not duplicate material already published. I confirm all authors consent to publication of this Work and authorise the granting of this licence.

For peer review only

Quantifying the carbon footprint of clinical trials: guidance development and pilot study

Jessica Griffiths (Corresponding Author) *, Lisa Fox*, Paula Williamson§ on behalf of the Low Carbon Clinical Trials Group

*Clinical Trials and Statistics Unit at The Institute of Cancer Research, London, UK

§MRC/NIHR Trials Methodology Research Partnership, Department of Health Data Science, University of Liverpool, UK

Corresponding author email address: Jessica.Griffiths@icr.ac.uk

Low Carbon Clinical Trials Group: Fiona Adshead, Sustainable Healthcare Coalition, UK; Rustam Al-Shahi Salman, Edinburgh Clinical Trials Unit, University of Edinburgh, UK; Craig Anderson, The George Institute, Australia; Emma Bedson, Liverpool Clinical Trials Centre, UK; Judith Bliss, Clinical Trials and Statistics Unit, The Institute of Cancer Research London, UK; Ana Boshoff, Imperial Clinical Trials Unit, Imperial College London, UK; Xiaoying Chen, The George Institute, Australia; Denise Cranley, Edinburgh Clinical Trials Unit, University of Edinburgh, UK; Peter Doran, University of Galway, Ireland; Carrol Gamble, Liverpool Clinical Trials Centre, UK; Kerenza Hood, Centre for Trials Research, Cardiff University, UK; Naomi McGregor, Newcastle Clinical Trials Unit, Newcastle University, UK; Carolyn McNamara, Technology & Innovation, KCR, UK; Elis Midha, School of Medicine, Cardiff University, UK; Keith Moore, Sustainable Healthcare Coalition, UK; Alexis M Perkins, Imperial Clinical Trials Unit, Imperial College London, UK; Sarah Pett, MRC Clinical Trials Unit at UCL, Institute of Clinical Trials and Methodology, UCL, London, UK; Matthew R Sydes, MRC Clinical Trials Unit at UCL, Institute of Clinical Trials and Methodology, UCL, London, UK.

ABSTRACT

Background: The urgency of the climate crisis requires attention from biomedical research, not least clinical trials which can involve significant greenhouse gas emissions. The Low Carbon Clinical Trials

For peer review only

(LCCT) Working Group set out a strategy to reduce the emissions of clinical trials, starting with the development of a method to measure their carbon footprint (CO_2e).

Methods: As a first step, we developed a process map defining clinical trial core activities. Corresponding emission factors were sourced to convert activity data into greenhouse gas emissions. The subsequent method was piloted by application on two CRUK-funded trials (the international randomised sarcoma trial CASPS [ISRCTN63733470] and the UK cohort-based breast cancer trial PRIMETIME [ISRCTN41579286]). A guidance document defining the scope, method and assumptions was written to allow application to any clinical trial.

Results: Trial specific activities over and above routine care were grouped into 10 modules covering trial set up, conduct and closure. We identified emission factors for all trial activities within both trials and used them to estimate their total carbon footprint. The carbon footprint of CASPS, an international phase 2 clinical trial of an investigational medicinal product with 47 participants, was 72 tonnes CO_2e, largely attributable to clinical trials unit emissions and staff travel. PRIMETIME, a UK-based phase 3 non-investigational medicinal product trial with 1962 patients, produced 89 tonnes CO_2e, largely attributable to trial-specific in-person participant assessments.

Conclusion: We have developed a method and guidance that trialists can use to determine the carbon footprint of clinical trials. The guidance can be used to identify carbon hotspots where alternative approaches to trial design and conduct could reduce a trial footprint, and where methodology research is required to investigate the potential impact of interventions taken to reduce carbon emissions. We will continue to refine the guidance to increase the potential application and improve usability.

STRENGTHS AND LIMITATIONS

- This is the first published and publicly available method and guidance to carbon footprint a clinical trial that can be used to inform lower carbon trial design.

For peer review only

- The guidance has been developed by an extensive and broad reaching collaboration and is intended for use by trialists with no prior experience of carbon footprinting.
- The method and guidance accounts for the carbon footprint of a clinical trial but no other metrics that may be important to consider in a full life cycle analysis.
- The emission factors used are the most appropriate publicly available factors that we could identify. More suitable or up to date emission factors may be available in the future or via paid subscription.
- The validity of results generated by the guidance is dependent on the quality and completeness of the activity data collated and the emission factors used.

INTRODUCTION

The World Health Organization has called climate change "the single biggest health threat facing humanity today" [1]. Whilst clinical trials are critical to identifying effective and safe treatments, in line with all healthcare activities, they also have a significant environmental impact. This contribution was first recognised around 15 years ago, when Ian Roberts and other members of the Sustainable Trials Study Group concluded that *"clinical trials contribute substantially to greenhouse gas emissions…. Notably through energy use in research premises and air travel"* [2]. Another study conducted by Lyle et al in 2009 of 12 UK pragmatic randomised trials, involving an average of 402 participants, showed that the average carbon emissions generated by the trials was 78.4 tonnes [3]. Multiplying this total by the 350,000 national and international trials registered on ClinicalTrials.gov would estimate the emissions of all global trials to be about 27·5 million tonnes of carbon dioxide equivalent [4]. This total, which is likely a highly conservative estimate, is comparable to the 25 million tonne CO_2e total footprint of the UK National Health Service which accounts for 6% of the UKs total footprint [5, 6].

For peer review only

However, since these papers were published, little seems to have happened to quantify and consciously reduce the carbon consumption of clinical trials, but the urgency of the threat from climate change has increased exponentially.

It is not currently possible for trialists to easily estimate the environmental impact of running a clinical trial which limits their ability to contribute to climate change mitigation. However, there is recognition of the requirement to reduce the carbon footprint of clinical trials and practical guidance available encouraging this [7].

The Sustainable Healthcare Coalition (SHC) brought together the Low Carbon Clinical Trials (LCCT) Working Group including members of the MRC-NIHR Trials Methodology Research Partnership, UK and Ireland CRC trialists (led by the ICR-CTSU and the Liverpool Clinical Trials Centre), clinicians and others, to set out a strategy to reduce the carbon footprint of clinical trials [**Error! Bookmark not defined.**]. The first step to reducing the carbon footprint of a planned clinical trial is to reliably measure its potential footprint and identify the carbon hotspots. Subsequently, trialists will be able to interrogate alternative trial design approaches that may reduce the carbon footprint without impacting data quality, integrity, and validity.

Through an NIHR funded project [award ID: NIHR135419], we have developed a method and associated guidance that can be used by trialists to carbon footprint a clinical trial to inform future lower carbon trial design [8]. The aim is for the guidance to be applied prospectively during the design phase of a trial before trial funding is secured. In this paper we explain how the project team developed the guidance; illustrate initial pilot results from its application on two CRUK-funded trials managed by ICR-CTSU; and describe the ongoing work to refine and expand the guidance.

METHODS

The approach to estimating the carbon impact of a clinical trial comprised the following stages:

1) The development of a process map to capture all activities of a clinical trial;

For peer review only

2) The development of a method to quantify the carbon footprint of all trial activities conducted specifically for the trial and which are over and above the provision of routine care, as defined in the process map;

3) Testing of the methodology with selected clinical trials to identify activity carbon hotspots and opportunities for mitigation.

Process mapping

In order to calculate the carbon footprint of any product, process or service, the whole system must be understood and established. To do this, information was extracted from the NIHR Clinical Trials Toolkit and a series of information gathering meetings held with participating UKCRC registered CTUs to understand their trial portfolios and all possible activities across a wide variety of clinical trial designs. The output of this stage was a visual representation of a clinical trial and a list of activities considered to be core clinical trial activities, as shown in **Figure 1.**

Guidance development

The core trial activities were grouped into the following modules: Trial set up; CTU emissions (includes energy and heating used in research premises, trial staff commuting and statistical analysis); Trial specific meetings and travel; Treatment intervention; Data collection and exchange; Trial supplies and equipment; Trial specific patient assessments; Samples; Laboratory; and Trial close out.

The carbon footprint of a trial was calculated by multiplying activity data by emission factors. Only activities undertaken to answer the research question, over and above routine care, were included. To identify suitable emission factors for each of the activities within the system boundary, data sources including Ecoinvent version 2.2, GOV.UK GHG conversion factors, and the SHC care pathway carbon calculator were used [9, 10]. The most applicable and up to date factors that are freely available for public use were selected, and all sources referenced. It is important to note that in some

For peer review only

cases more up to date factors, or forecasted emission factors may be available, but they are subject to licensing requirements and as such are not publicly available.

Two documents were produced, a "Detailed Guidance and method to calculate the carbon footprint of a clinical trial" and a "Data collation quick guide and worksheet". The detailed guidance defines the project scope, limitations, assumptions, emission factors and benchmark data sources, as well as detailed breakdowns of the calculations required to calculate the carbon footprint of each activity. The "Data collation quick guide and worksheet" guides users in the application of the detailed guidance by summarising the calculations in their simplest form and displaying the activity data the user is required to collect in order to complete the required calculations. See **Appendices A and B** for the current version of the detailed guidance and worksheet respectively.

Pilot – applying the guidance

Although we intend for the guidance to be used prospectively at the trial design stage, in order to test the method and develop a full and comprehensive set of calculations in the pilot, this guidance was retrospectively and iteratively applied to two ICR-CTSU managed, CRUK-funded trials, CASPS and PRIMETIME. CASPS is an international phase II trial of cedirinib in the treatment of patients with alveolar soft part sarcoma. The trial enrolled 47 patients across twelve sites in the UK, Spain, and Australia and involved an internationally shipped IMP, completion and shipment of paper CRF, on site visits by the trial team for site initiation, monitoring visits and audit, additional hospital visits for patients for trial-specific assessments and provision of tissue samples. PRIMETIME is a post-operative avoidance of radiotherapy trial which recruited 1962 patients across 64 sites in the UK. The trial used electronic data capture for collection of data, data linkage with NHS routine data sources and involved shipment of tissue samples for immunohistochemistry testing and subsequent long-term sample storage.

For peer review only

These two trials were selected because of their contrasting designs (International CTIMP vs national non-CTIMP), the trials were complete (meaning we could test the method on the entirety of a trial), and activity information was readily available.

Having identified the trial activities using the process map and guidance, activity data was gathered from the trial protocol, Site Investigator File, funding application, trial specific databases, systems and trackers, and through discussion with the trial teams. Once collated, the trial activity data was multiplied by the emission factors identified in the guidance to calculate a carbon footprint. Application to real life, complete trials resulted in iteration of the guidance and the addition of activities not previously identified, as well as their correlating emission factors e.g. data linkage and copying images from trial-specific scans to CDs. The full calculations for CASPS and PRIMETIME can be found in appendices C and D respectively.

RESULTS

The guidance has been developed and used to calculate the carbon footprint of two clinical trials (results were presented at ICTMC in Oct 2022).

CASPS TRIAL

Applying the guidance, the carbon footprint of CASPS was estimated to be 72 tonnes (71,939 $kgCO_2e$). The carbon footprint of a typical UK citizen is 12.7 tonnes CO_2e per year, meaning CASPS has a footprint equivalent to around 6 UK citizens [11]. As shown below in **Figure 2**, the largest contribution to the footprint was CTU emissions (23,488 $kgCO_2e$), followed by trial specific meetings and travel (20,465 $kgCO_2e$), largely due to the sites in Australia which required internal flights for site visits. Despite the analysis of samples for a sub-study being out of scope, central laboratory activities still contributed significantly to the footprint due to storage of samples in -80 degree freezers for up to 10 years.

PRIMETIME TRIAL

For peer review only

The total carbon footprint of PRIMETIME was calculated to be 89 tonnes (89,360 kgCO_2e). This is equivalent to 7 times the annual carbon footprint of a UK citizen.

Figure 3 demonstrates that the largest contribution to the PRIMETIME carbon footprint was patient travel and assessments (60,823 kgCO_2e), followed by CTU emissions (11,733 kgCO_2e) and data collection and exchange (7572 kgCO_2e).

The PRIMETIME trial consisted of two treatment cohorts. In the standard of care cohort, the intervention comprised standard of care radiotherapy and annual follow up mammograms were undertaken for 5 years to assess disease outcome. In the other group, no radiotherapy was given and patients received annual follow up mammograms for 10 years to assess disease outcome. The additional 5 scans and associated patient travel were included in the PRIMETIME carbon footprint.

Further testing

The current version of the detailed guidance is undergoing further iterative testing on a selection of trials from 8 participating UK CRC CTUs as described below in **Table 1**. Through its wider application, we hope to capture all possible clinical trial activities and emission factors across a wide range of trial designs. Although the calculations required to carbon footprint the pilot trials in their entirety were extensive, through further testing and refinement of the guidance, we hope to identify hotspots so that future iterations of the guidance can be streamlined to enable data collection to be less resource-intensive.

The project team are also collecting qualitative data on the use and application of the guidance document. This will include how academic trialists managed this work alongside day-to-day trial management; the staff resource and time required to complete the calculations; any barriers to carrying out this work; how user friendly the guidance was and how much technical knowledge and support were required to complete the calculations.

Table 1. Participating CTUs testing the guidance and trials undergoing footprinting

For peer review only

CTU	Trial Name	Description
**Cardiff Centre for Trials Research**	The UK stand together trial	A two-arm pragmatic multicentre cluster randomised controlled trial which aims to evaluate the effectiveness and cost-effectiveness of KiVa, a school-based anti-bullying programme, in reducing bullying in schools compared to usual practice. 116 primary schools participated from four areas; North Wales, West Midlands, South East and South West England [12].
**Edinburgh Clinical Trials Unit**	RESTART	A prospective, open, blinded end point, parallel group randomized clinical trial that compared the effects of starting vs avoiding antiplatelet therapy after ICH. The trial recruited 537 participants at 122 hospitals in the UK [13].
**Imperial Clinical Trials Unit**	ON-PACE	On-PACE is a double-blind randomised trial investigating whether taking a nutritional supplement is beneficial for people with the most severe form of chronic obstructive pulmonary disease (COPD). It will recruit 96 people with COPD who use oxygen at home to take part in a 3 month long clinical trial [14].
**Liverpool Clinical Trials Centre**	HEAL-COVID	HElping Alleviate the Longer-term Consequences of COVID-19 (HEAL-COVID), an adaptive platform trial, aims to evaluate the impact of treatments on longer-term morbidity, mortality, re-hospitalisation, symptom burden and quality of life associated with COVID-19. The trial took place across 109 sites and randomised 1245 participants [15].
**MRC Clinical**	MAVMET	A multicentre, 48 week randomised controlled factorial trial of adding maraviroc and/or metformin for hepatic steatosis in HIV-1-

For peer review only

**Trials Unit at UCL**		infected adults on combination antiretroviral therapy. The trial took place at 6 sites across the UK and recruited 90 participants [16].
**Newcastle Clinical Trials Unit**	PREMISE	A multi-arm, multi-centre, non-inferiority randomised controlled trial comparing 3 minimally invasive treatments to the current gold standard operation for bladder obstruction due to enlarged prostate in the National Health Service. The planned sample size is 536 [17].
**The George Institute**	INTERACT3	An international, multicentre, prospective, stepped wedge, cluster randomised, blinded outcome assessed, controlled trial of a care bundle of physiological control strategies in acute intracerebral haemorrhage. The trial recruited 7064 patients from 122 hospitals in 10 countries (Chile, Brazil, China, India, Mexico, Nigeria, Pakistan, Peru, Sri Lanka, and Vietnam) [18].
**University of Galway**	EMERGE	A randomised placebo-controlled trial of the Effectiveness of MEtformin in addition to usual care in the Reduction of GEstational diabetes mellitus effects. Planned sample size is 550 [19].

For peer review only

DISCUSSION

We have developed guidance that trialists could use to carbon footprint clinical trials and subsequently inform lower carbon trial design and delivery.

The detailed guidance document, enclosed as an appendix, describes a method that can be used to calculate the carbon footprint of publicly funded, CTIMP and Non-CTIMP (inc complex intervention) trials conducted in the NHS. The guidance is currently undergoing testing on a wider selection of trials managed by collaborating UK CRC Registered Clinical Trial Units (UK CRC CTUs). The project team plan to publish the results of all additional trials footprinted as part of the subsequent testing phase and

updates to the guidance this will inevitably generate, on completion of the currently funded project. The collaboration is open to, and welcomes new partners.

It is important to note that this guidance accounts only for the carbon footprint of a clinical trial. It does not include other metrics that are important to consider in a full life cycle analysis when evaluating environmental impact and potential trade-offs, including water use, land use, waste and those relating to social and economic impacts. It is also necessary to note that the validity of the carbon footprinting results the guidance generates is highly dependent on the quality and completeness of the activity data collated and the specific emission factors used. We recognise that more suitable or up to date emission factors than those provided may be available or will become available as knowledge around the subject grows, but these could not be included as they are not publicly or freely available.

A number of assumptions are defined in the guidance to ensure consistency of application to different clinical trials and a degree of pragmatism applied, such as using average occupancies for members of hospital staff. All assumptions made are described in full in the guidance and can be interrogated in the real-life example calculations included in appendices C and D.

Previous research clearly demonstrated that clinical trials have an impact and where the carbon hotspots might be (trial team commuting, energy use in research premises, trial travel and freight). It is difficult to compare our pilot results to those of the CRASH-1 and CRASH-2 carbon audits as they were large international trials which enrolled 10,008 and 20,211 patients respectively, and emissions were calculated over a single, one-year period. We also grouped activities in different ways; in the CRASH audit the footprint of all deliveries was presented together, whereas here deliveries were calculated within their specific module, for example delivery of SIFs was included with 'trial set up' activities. Trial coordination centre emissions were the largest and second largest contributors in the CRASH trials, similarly in the pilot results presented here, 'CTU emissions' was the largest contributor to the CASPS footprint and the second largest in PRIMETIME. Trial team commuting and energy use in

For peer review only

research premises, which were calculated together in the current guidance under 'CTU emissions', also accounted for the most CO_2e emissions in the 2009 study conducted by Lyle et al. This study calculated the average emissions of 12 UK trials to be 78.4 tonnes, comparable to the 72 and 89 tonne carbon footprints of CASPS and PRIMETIME. Patient travel was not calculated in the CRASH trials and was only the 4th largest contributor to emissions in the Lyle study. Conversely, in the pilot results presented here 'trial specific patient assessments' was the largest contributor to PRIMETIME emissions and included both patient travel and procedures.

The results presented are in agreement with previously identified hotspots, however the pilot has also identified different and new hotspots, such as data collection and exchange and laboratory associated activities. The pilot results also show that the % contribution by activity varied across the two trials, demonstrating the need to collect data from more trial types and designs to capture all possible hotspots.

As well as refinement and enhancement of the guidance to ensure it is user-friendly and can be applied to as many clinical trial types as possible, more trials must be footprinted to understand how to define the scope of what is considered a clinical trial and how best to apply the guidance.

The carbon footprint associated with the manufacture and delivery of the trial intervention are out of scope in the current guidance. However, when implementing the results of a clinical trial it will also be important to consider the footprint of the recommended intervention. This latter point is exemplified by the PRIMETIME trial, where delivery of the trial has a notable carbon footprint. However, if the trial results recommend the avoidance of radiotherapy for some breast cancer patients, there would be a significant carbon saving because standard of care radiotherapy and associated patient travel would no longer be required (the carbon footprint associated with delivering 15 doses of standard of care radiotherapy is 94.19 kgCO_2e per patient, consisting of 7.19 kg CO_2e for the radiotherapy and 87 kgCO_2e for the travel required) [20, 9]. Further work is required to develop guidance on the interpretation of carbon footprinting results, so that the impact of conducting a

For peer review only

clinical trial can be balanced against the improvement an intervention may make both in terms of patient outcomes and the carbon footprint of the intervention once incorporated into standard of care.

The results of the future work with collaborating CTUs summarised in Table 1 will be published in due course and will include discussion and comparison of the hotspots, as well as evaluation of the inter-observer reliability and reproducibility. We will also look at the time commitment required to complete the calculations and how the guidance can be improved for use by those without specialist carbon footprinting knowledge. In this pilot work the guidance was applied retrospectively to completed trials, however, ultimately the aim is to use the guidance prospectively at the trial design stage. We anticipate application in this scenario will be less time and resource intensive, but this will be analysed and discussed in the next paper, as we plan to include trials which are currently in development. Our findings will contribute to ongoing work coordinated by the Sustainable Healthcare Coalition to develop an online, open-access carbon footprint calculator in collaboration with a group of pharmaceutical industry representatives conducting similar work. Through this collaboration, the generalisability of a common method and the scope of its application to academia and industry will be examined.

There remains much to do to help shape the future of sustainable clinical trials and support the move towards more responsible research. Investigation of carbon trade off decisions with patient and public involvement is necessary to enable recommendations to be made to reduce the carbon footprint of clinical trials, without reducing the clinical value of the trial and acceptability to participating patients and work with patient partners to shape the next steps of this important research is planned. Dissemination and engagement activities, including training and practical support for research teams incorporating carbon calculations into routine trial design, will be required to help drive the paradigm shift to responsible greener research.

For peer review only

As we gather more data on the estimated carbon footprint of planned clinical trials, further consideration should be given to how various stakeholders are going to use and consider the information generated. Funding panels often refer to cost per patient, a concept proposed for this area in terms of the carbon emission per patient. Care would be needed to interpret this statistic; as is the case with the cost per patient, carbon footprint per patient may discriminate against rare disease trials with a high health burden, as it does not include information on the nature and consequences of the condition under study. Further consideration is also required to determine a meaningful way to present the carbon footprint information and provide context to the public. A suggestion has been made that graphical representation in terms of the equivalent number of UK citizens' footprints could be more easily understood, but further research is needed.

Ultimately, a method and associated guidance describing how to carbon footprint a clinical trial will support various organisations including universities, health service providers and research funders to meet their carbon reduction commitments and allow stakeholders to incentivise a reduction of the carbon footprint of trials in their portfolios. We have produced this guidance as the first step of a strategy to help towards achieving this aim.

Figure legends

Figure 1. A process map displaying the core activities of a clinical trial considered within the system boundary of the carbon footprinting calculations.

Figure 2. A breakdown of the contributions to CASPS carbon footprint

Figure 3. A breakdown of the contributions to PRIMETIMEs carbon footprint

Acknowledgements

This project is funded by the National Institute for Health Research (NIHR) CTU Support Funding scheme [Award ID: NIHR135419]. The views expressed are those of the author(s) and not necessarily those of the NIHR or the Department of Health and Social Care.

For peer review only

Grateful thanks to Environmental Resources Management for their technical support and expertise in the development of the method.

Competing Interests

JG, LF, JB, PW and CG have received NIHR funding to undertake this study (support for the manuscript). FA is the paid chair of the Sustainable Healthcare Coalition. KM is a member of the Sustainable Healthcare Coalition. RA-SS reports an NIHR Health Technology Assessment trial grant (CARE pilot trial) and an NIHR Efficacy and Mechanism Evaluation (EME) Programme Application Acceleration Award – International platform studies in precision medicine (CARE PREP) and consulting fees from Recursion Pharmaceuticals, McMaster University Population Health Research Institute, and Bioxodes, all paid to The University of Edinburgh. MRS reports grants from Astellas, Clovis Oncology, Janssen, Novartis, Pfizer, and Sanofi-Aventis; consulting fees from Eli Lilly; speaker's fees from Lilly Oncology, Janssen and payment from Eisai, all unrelated to the topic of this Comment. SP reports grants/contracts from EDCTP; MRC; Gilead Sciences; Janssen-Cilag; ViiV Healthcare; NIHR; University of Minnesota and is a member of the TIPAL DSMB. PD reports grants from Health Research Board of Ireland and Enterprise Ireland for Peer Reviewed research funding. CSA is the Vice President of the World Stroke Organisation and is Editor-in-chief of Cerebrovascular Diseases, and holds Research grants from National Health and Medical Research Council (NHMRC) of Australia, Medical Research Council (MRC) of the UK, Takeda and Penumbra (paid to the George Institute). EB, EM, CM, AMP, AB, DC, XC, KH, NM report no competing interests.

References

[1] Climate change and health [Internet]. World Health Organization. 2021 October 30 [cited 2023 May 11]. Available from: https://www.who.int/news-room/fact-sheets/detail/climate-change-and-health
[2] Sustainable Trials Study Group. Towards sustainable clinical trials. *BMJ* 2007;334:671
[3] Lyle K, Dent L, Bailey S, et al. Carbon cost of pragmatic randomised controlled trials: retrospective analysis of sample of trials. *BMJ* 2009;339:b4187
[4] Adshead F, Al-Shahi Salman R, Aumonier S, Collins M, Hood K, McNamara C, Moore K, Smith R, Sydes MR, Williamson PR (2021) A strategy to reduce the carbon footprint of clinical trials. Lancet. 2021 Jul 24;398(10297):281-282. doi: 10.1016/S0140-6736(21)01384-2. 34171294.
[5] Tennison I, Roschnik S, Ashby B, Boyd R, Hamilton I, Oreszczyn T, et al. Health care's response to climate change: a carbon footprint assessment of the NHS in England. The Lancet Planetary Health [Internet]. 2021 Feb 1;5(2):e84–92. Available from: https://www.thelancet.com/journals/lanplh/article/PIIS2542-5196(20)30271-0/fulltext
[6] The NHS' "first climate friendly" operation [Internet]. BBC News. 2022 May 25 [cited 2023 May 12]. Available from: https://www.bbc.co.uk/news/uk-england-birmingham-61567187
[7] NIHR Carbon Reduction Guidelines [Internet]. NIHR. 2019 July 30 [cited 2023 May 11]. Available from: https://www.nihr.ac.uk/documents/the-nihr-carbon-reduction-guidelines/21685
[8] Development and prototype testing of a method to quantify the carbon footprint of current clinical trials to inform future lower carbon trial design [Internet]. NIHR funding and awards. [cited 2023 May 11]. Available from: https://fundingawards.nihr.ac.uk/award/NIHR135419

For peer review only

[9] Greenhouse gas reporting: conversion factors 2022 [Internet]. Gov.uk. 2022 [cited 2023 May 11]. Available from: https://www.gov.uk/government/publications/greenhouse-gas-reporting-conversion-factors-2022
[10] Care pathways carbon footprint calculator [Internet]. Sustainable Healthcare Coalition. [cited 2023 May 11]. Available from: https://shcpathways.org/full-calculator/
[11] Kayser B. What is the average carbon footprint per person in the UK? | Pawprint- Your Eco Companion [Internet]. 2020. [cited 2023 May 11]. Available from: https://www.pawprint.eco/eco-blog/average-carbon-footprint-uk
[12] Clarkson, S., Bowes, L., Coulman, E. *et al.* The UK stand together trial: protocol for a multicentre cluster randomised controlled trial to evaluate the effectiveness and cost-effectiveness of KiVa to reduce bullying in primary schools. *BMC Public Health* **22**, 608 (2022). https://doi.org/10.1186/s12889-022-12642-x
[13] REstart or STop Antithrombotics Randomised Trial (RESTART) [Internet]. The University of Edinburgh. 2021 December 1 [cited 2023 May 11]. Available from: https://www.ed.ac.uk/clinical-brain-sciences/research/completed-studies-trials/restart-or-stop-antithrombotics-randomised-trial
[14] Improving the experience of physical activity in people with severe lung disease using dietary nitrate supplementation with beetroot juice [Internet]. ISRCTN. [cited 2023 May 11]. Available from: https://www.isrctn.com/ISRCTN12474100
[15] HElping Alleviate the Longer-term Consequences of COVID-19 (HEAL-COVID) [Internet]. ClinicalTrials.gov. 2021 March 17 [cited 2023 May 11]. Available from: https://clinicaltrials.gov/ct2/show/NCT04801940
[16] MAVMET [Internet]. MRC Clinical Trials Unit at UCL. [cited 2023 May 12]. Available from: https://www.mrcctu.ucl.ac.uk/studies/all-studies/m/mavmet/
[17] PREMISE [Internet]. Newcastle Clinical Trials Unit. Newcastle University. [cited 2023 May 11]. Available from: https://www.ncl.ac.uk/nctu/trials/therapeutic-areas/renal/premise/
[18] The Third, Intensive Care Bundle With Blood Pressure Reduction in Acute Cerebral Hemorrhage Trial (INTERACT3) [Internet]. ClinicalTrials.gov. 2017 July 6 [cited 2023 May 11]. Available from: https://clinicaltrials.gov/ct2/show/NCT03209258
[19] Dunne F, Newman C, Devane D, Smyth A, Alvarez-Iglesias A, Gillespie P, Browne M, O'Donnell M. A randomised placebo-controlled trial of the effectiveness of early metformin in addition to usual care in the reduction of gestational diabetes mellitus effects (EMERGE): study protocol. Trials. 2022 Sep 21;23(1):795. doi: 10.1186/s13063-022-06694-y. PMID: 36131291; PMCID: PMC9494837.
[20] Shenker, RF., Johnson, TL., Ribeiro, M. *et al*. Estimating Carbon Dioxide Emissions and Direct Power Consumption of Linear Accelerator–Based External Beam Radiation Therapy. *Advances in Radiation Oncology* (2023) 8, 101170. https://doi.org/10.1016/j.adro.2022.101170

Appendices

Appendix A: Detailed guidance

Appendix B: Quick guide

Appendix C: CASPS calculations

Appendix D: PRIMETIME calculations

For peer review only

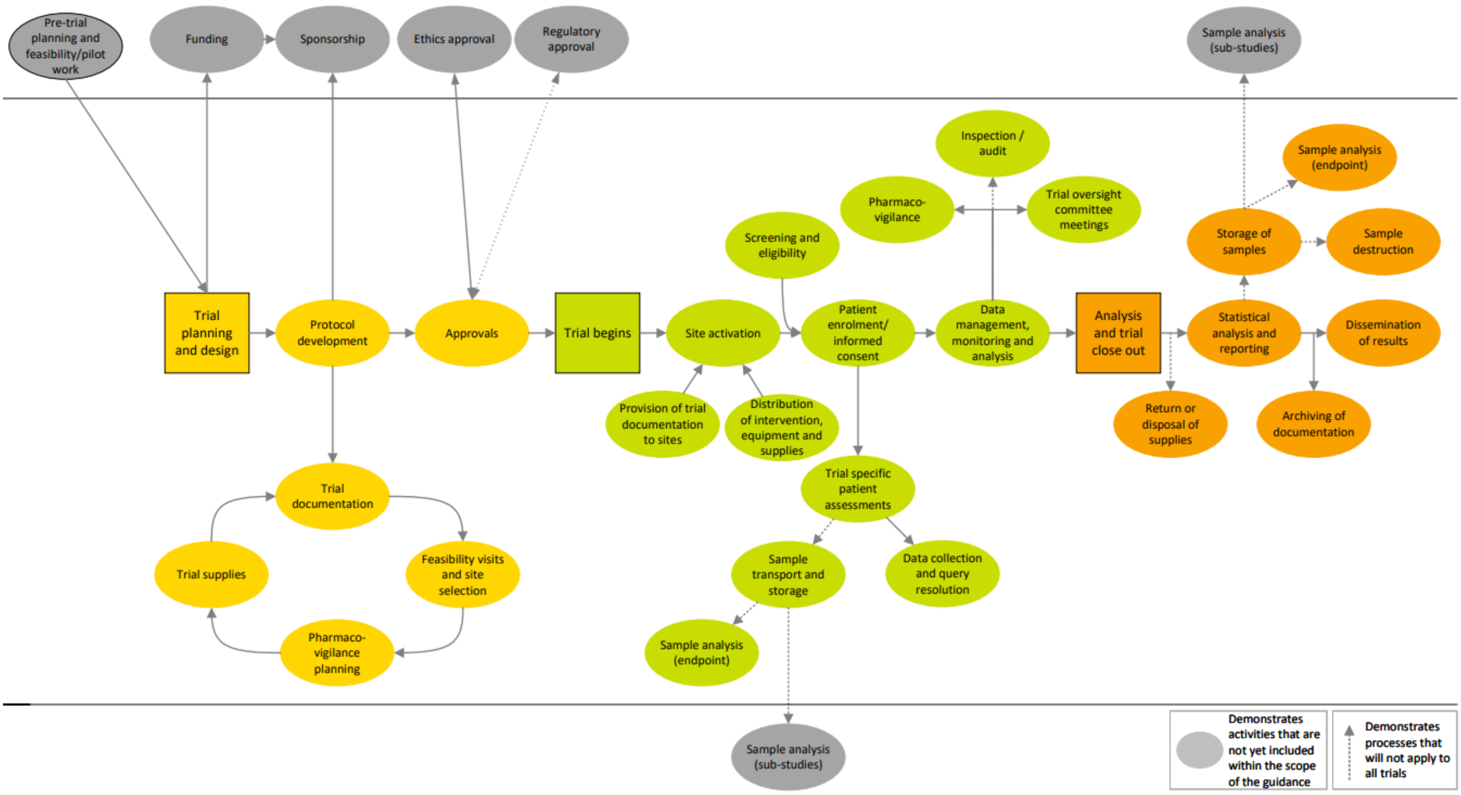
Pre-trial planning and feasibility/pilot work
Funding
Sponsorship
Ethics approval
Regulatory approval
Sample analysis (sub-studies)
Inspection / audit
Sample analysis (endpoint)
Pharmaco-vigilance
Trial oversight committee meetings
Screening and eligibility
Storage of samples
Sample destruction
Trial planning and design
Protocol development
Approvals
Trial begins
Site activation
Patient enrolment/ informed consent
Data management, monitoring and analysis
Analysis and trial close out
Statistical analysis and reporting
Dissemination of results
Provision of trial documentation to sites
Distribution of intervention, equipment and supplies
Return or disposal of supplies
Archiving of documentation
Trial documentation
Trial specific patient assessments
Trial supplies
Feasibility visits and site selection
Sample transport and storage
Data collection and query resolution
Pharmaco-vigilance planning
Sample analysis (endpoint)
Sample analysis (sub-studies)
Demonstrates activities that are not yet included within the scope of the guidance
Demonstrates processes that will not apply to all trials

review only

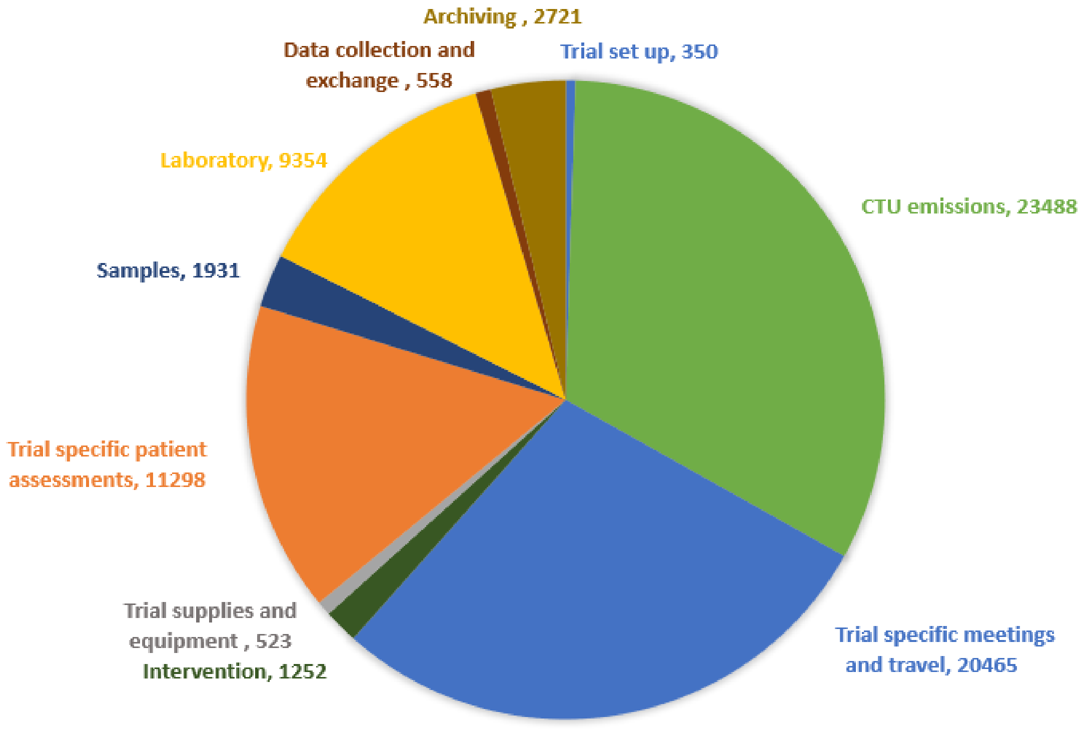

CONTRIBUTION TO CASPS CARBON FOOTPRINT (kg CO_2e)

For peer review only

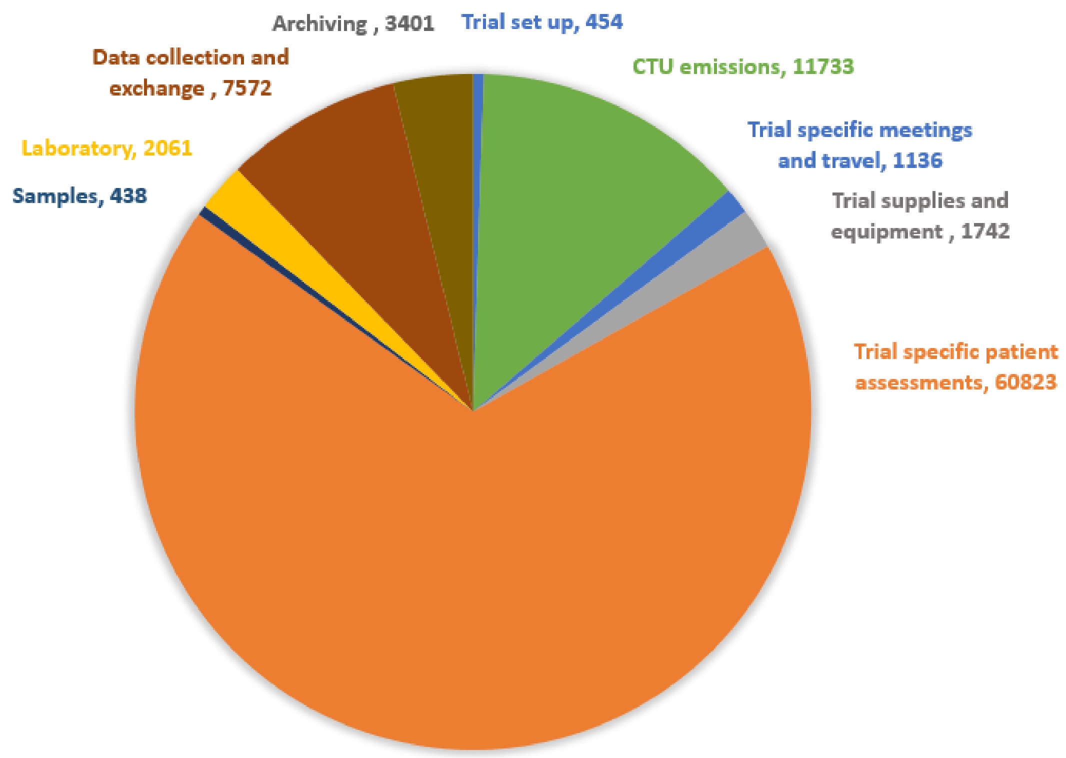

CONTRIBUTION TO PRIMETIME CARBON FOOTPRINT (kg CO_2e)

For peer review only

FUNDED BY NIHR | National Institute for Health and Care Research

Liverpool Clinical Trials Centre LCTC

ICR

Enabling lower carbon clinical trials: Development and prototype testing of a method to quantify the carbon footprint of clinical trials to inform future lower carbon clinical trial design

Detailed Guidance and method to calculate the carbon footprint of a clinical trial

Background

Around 15 years ago, the Sustainable Trials Study Group concluded that clinical trials contribute substantially to greenhouse gas emissions, notably through energy use in research premises and air travel[1].

In addition, a study conducted in 2009 of 12 UK pragmatic randomised trials involving an average of 402 participants showed that the average carbon emission generated by the trials was 78.4 tonnes[2]. Multiplying this total by the 350,000 national and international trials registered on ClinicalTrials.gov, this would estimate that emissions attributable to all global clinical trials to be about 27·5 million tonnes of carbon dioxide equivalent[3].

Since then, the urgency of the threat from the climate crisis has increased exponentially and the World Health Organization calls climate change the single biggest health threat facing humanity[4]. Planned climate action is not sufficient to prevent the current warming predictions; humanity must limit warming to 1.5 degrees and reach net-zero by 2040 to avoid the worst consequences of climate change.

As a first step to reduce the environmental impact of clinical trials, a method to quantify the carbon footprint of a clinical trial and associated processes is required.

Introduction

This guidance provides information on how to carbon footprint a clinical trial for the purposes of the NIHR-funded project 'enabling lower carbon clinical trials.'

Within the guidance, clinical trial processes have been sub-divided into the following modules:

1. Trial set up
2. CTU emissions
3. Trial specific meetings and travel
4. Treatment intervention
5. Data collection and exchange
6. Trial Supplies and equipment
7. Trials specific patient assessments
8. Samples
9. Laboratory
10. Trial close out

The above list is not exhaustive, and it is expected that further activities and modules may need to be added to account for specialist processes in all clinical trial types and as knowledge around life cycle analysis increases.

NB: analysis of data does not need to be calculated separately, it is covered by the emissions attributed to trial staff FTE in "CTU emissions" and calculations included within "Data Collection and exchange".

For peer review only - http://bmjopen.bmj.com/site/about/guidelines.xhtml

FUNDED BY NIHR | National Institute for Health and Care Research

LIVERPOOL CLINICAL TRIALS CENTRE LCTC

ICR

Calculating carbon footprint

A carbon footprint is a measure of greenhouse gases, usually quoted in kg or tonnes of carbon dioxide equivalent (CO_2e). To calculate the carbon footprint of a particular clinical trial process, both 'activity data' and 'emission factors' are required.

An 'activity' could be anything from electricity consumption to materials, travel and food. Activity data quantifies the amount of that activity e.g. distance travelled, kWh used etc.

An emission factor, also known as a conversion factor, "is a coefficient which allows you to convert the activity data into greenhouse gas emissions. It is the average emission rate of a given source, relative to units of activity or process/processes."[5]

To calculate a carbon footprint of a trial, the activity data will need to be provided by the trial management team and multiplied by the emission factors provided in this guidance document. Two types of activity data may be used:

- Primary data: data collected first-hand from specific activities within the studied clinical trial process i.e., the data collected where you can determine the amount of the activity taking place, for example electricity in kWh used by a building or the weight of an IMP shipment and the distance it travels.

- Secondary data: activity data that is not collected from specific activities within the studied clinical trial because you cannot determine or measure the exact quantity of the activity taking place, for example the number of m^2 occupied by an office worker or hospital worker. Secondary data may take the form of average, or typical, information about an activity from a published study or other source and will be provided in this guidance document e.g., average m^2 occupied by an office worker or average distance travelled.

Primary activity data are preferred for all activity data used in each module. However, secondary data may be used where primary activity data is unavailable or difficult to obtain.

It is important to avoid double-counting activities i.e., modules must not include activities already covered by other modules in the clinical trial process map. A data collection tool is provided alongside this guidance to aid in this process and help avoid double-counting.

Scope

This guidance describes a method to calculate the carbon footprint of a UK, academic clinical trial.

It can be applied to trials with international participation, however as emission factors vary between countries, and those provided within this guidance are UK-specific, country-specific emission factors may be required. Proxy emission factors which have been approved for use can be used where required and the source country of an emission factor will be stated where applicable.

The tool is intended to calculate the carbon footprint of the processes specific to the clinical trial, defined as the data required to analyse the trial endpoints and the research activities over and above standard of care.

The guidance is intended for use as a tool to inform sustainable decision-making in the design of clinical trials, rather than a tool to calculate the absolute footprint of a clinical trial or compare environmental performance of one trial over another.

For peer review only - http://bmjopen.bmj.com/site/about/guidelines.xhtml

Limitations

There are a number of emission factors that can be used for a particular process and activity data can be calculated in a number of ways. Therefore, life cycle analysis produces variation in its results, dependent on choices made by the individual performing the calculations.

We have endeavoured to include an explanation or justification for the choice of emission factors used. In addition, the emission factors have been selected as the most applicable and up to date factors that are freely available for public use. It is important to note that more up to date factors, or forecasted emission factors, may be available, but they are subject to licensing requirements and are not publicly available. The source of all factors used is included for reference.

This guidance accounts only for the greenhouse gas emissions. It does not include other metrics that are also important to consider when evaluating sustainability and the potential trade-offs, for example water use, land use, waste and those relating to social and economic impacts.

Assumptions

- The eventual aim of this tool is to be used prospectively during the design phase of a trial, before trial funding is secured. However, the tool will not capture the carbon footprint associated with work conducted during this period i.e., prior to confirmation of funding award. The tool can also be used retrospectively on clinical trials which are complete.
- The tool only calculates emissions of processes which have been funded and defined within the trial protocol i.e., future planned work which has not yet been funded or that will be defined outside of the protocol are not included.
- The tool only calculates the emissions of patient participation where it exceeds or is additional to routine care, where it is required to establish an endpoint, the patient population or eligibility. Use of the trial SOECAT or the costing included in the initial funding application (if the trial predates use of SOECAT) is encouraged, to consistently define the investigations considered in addition to routine care and/or part of the research question.
- For all translational/optional/research samples and sub studies, the tool does not calculate emissions associated with analysis performed by central laboratories or collaborators, but does calculate emissions for activities defined in the protocol, such as collecting the sample or data from patients, initial processing at participating sites and shipment of samples or data to the site of subsequent analysis.
- This guidance will only appraise trials with UK based trial management (sites may be international but the trial must be overseen by a UK Clinical Trials Unit/Sponsor/ Research team)
- The carbon footprint associated with the manufacture of a trial intervention e.g. IMPs and Medical Devices is not included in this guidance.
- The tool does not calculate the carbon footprint of waste associated with a particular clinical trial. Concerning clinical trial consumables, activity data is based on the quantities purchased. The only exception to this is the destruction of unused IMP at participating sites, as this an activity specifically undertaken for a clinical trial which has an associated carbon footprint.
- With respect to trial specific meetings (eg. IDMC/TMG/TSC groups), the carbon footprint associated with meetings/travel will be calculated but emissions associated with general information flow e.g. emailing, printing for these meetings is not included.
- The tool does not calculate the carbon footprint of hospital and laboratory staff commuting.
- Carbon emissions associated with ethics and regulatory approval are not within the scope of this guidance and will not be calculated.

For peer review only - http://bmjopen.bmj.com/site/about/guidelines.xhtml

FUNDED BY NIHR | National Institute for Health and Care Research

LIVERPOOL CLINICAL TRIALS CENTRE LCTC

ICR

NB: more module specific assumptions can be found throughout the document where applicable.

1. Trial set up

This module includes the following activities:

1.1 Production of trial documentation to be sent to sites or participants
1.2 Provision/postage of trial documentation to sites
1.3 Provision/postage of documentation to participants by CTU or participating sites
1.4 Provision/postage of incentives to participant

1.1. Production of trial documentation

For production of trial documentation, the carbon footprint of both the printing and materials must be calculated.

Printing: The number of pages must be multiplied by 0.005 to produce a weight in kilograms, and the weight multiplied by the relevant emission factor provided below.

- Black and white: kg (paper) x 0.22438 = kgCO_2e
- Colour: kg (paper) x 0.31786 = kgCO_2e

1292	use, printer, laser jet, b/w, per kg printed paper	electronics	services	kg	RER	0.22438
1293	use, printer, laser jet, b/w, printing per h	electronics	services	h	CH	0.37213
1294	use, printer, laser jet, b/w, printing per h	electronics	services	h	RER	0.39065
1295	use, printer, laser jet, colour, per kg printed paper	electronics	services	kg	CH	0.2027
1296	use, printer, laser jet, colour, per kg printed paper	electronics	services	kg	RER	0.31786

NB**:** If you are unable to calculate the number of pages, you may assume that there are 150 pages in a small ring binder and 500 pages in a large lever arch folder.

Assumption: 1 piece of paper weighs around 5g/0.005kg

Emission factor source: Ecoinvent, version 2.2, 2011 [6] (RER = European emission factor)

Materials (paper): For the carbon footprint of paper production**,** the number of pages must be multiplied by 0.005 to produce a weight, and the weight multiplied by the emission factor for paper manufacture provided below.

Kg (paper) x 0.919 = kg CO_2e

Paper and board: paper	Gov.UK	2021	kg	0.919

Emission factor source: Greenhouse gas reporting: conversion factors 2021, GOV.UK [7]

Materials (folders): As trial documentation is often sent to sites in folders, provided below are estimated weights of folders. The total weight in kg will then need be multiplied by the emission factor for cardboard (provided below).

53	Paper and board: board	Gov.UK	2021	kg	0.821

CF of the folder materials = Kg x 0.821 = kg CO_2e

Lever arch: Assumption: Weight of lever arch = 0.5kg

Ring binder: Assumption: Weight of ring binder = 0.3kg [Eastlight A4 Black Ring Binder - EA54121 (staples.co.uk)]

Emission factor source: Greenhouse gas reporting: conversion factors 2021, GOV.UK [7]

FUNDED BY NIHR | National Institute for Health and Care Research

LIVERPOOL CLINICAL TRIALS CENTRE LCTC

ICR

1.2, 1.3, 1.4 Provision of trial materials

For provision of trial materials to sites by post/courier you will need to calculate the total weight of the materials (in tonnes) and multiply this by the distance they travel (in km) to get tonne.km. The t.km is then multiplied by the emission factor for either road or air freight provided below.

Road freight:

Mass (tonnes) x distance (km) = t.km

t.km x emission factor = (kg CO_2e)

Emission factor for road freight = 0.19443

1942	2724 transport, lorry 20-28t, fleet average	transport systems	road	tkm	CH	0.19443

Emission factor source: Ecoinvent, version 2.2, 2011 [6]

Air freight:

Mass (tonnes) x distance (km) = t.km

t.km x most suitable emission factor from highlighted column = (kg CO_2e)

			With RF				Without RF			
Activity	Type	Unit	kg CO_2e	kg CO_2	kg CH_4	kg N_2O	kg CO_2e	kg CO_2	kg CH_4	kg N_2O
Freight flights	Domestic, to/from UK	tonne.km	4.49363	4.46949	0.00188	0.02226	2.3765	2.35236	0.00188	0.02226
	Short-haul, to/from UK	tonne.km	2.30229	2.2908	0.00008	0.01141	1.21717	1.20568	0.00008	0.01141
	Long-haul, to/from UK	tonne.km	1.0189	1.01381	0.00004	0.00505	0.53867	0.53358	0.00004	0.00505
	International, to/from non-UK	tonne.km	1.0189	1.01381	0.00004	0.00505	0.53867	0.53358	0.00004	0.00505

NB: ‘With RF’ values are recommended. RF (combustion and radiative forcing) includes the indirect and direct emissions.

NB: Always use values relating to ‘kgCO_2e’

For air freight you need to add the WTT (well-to-tank) value to get the final total for freight flight. WTT can be calculated by multiplying the t.km used in the first calculation by the correlating WTT conversion factor provided below.

Tonne.km x correlating WTT conversion factor

			With RF	Without RF
Activity	Type	Unit	kg CO_2e	kg CO_2e
WTT- freight flights	Domestic, to/from UK	tonne.km	0.49186	0.49186
	Short-haul, to/from UK	tonne.km	0.25210	0.25210
	Long-haul, to/from UK	tonne.km	0.11157	0.11157
	International, to/from non-UK	tonne.km	0.11157	0.11157

Emission factor source: Greenhouse gas reporting: conversion factors 2021, GOV.UK [7]

Assumption: If unknown, for delivery of trial supplies to patients or GP, use 17.4km as distance from hospital to patient, or hospital to GP. Source: BMJ 2009;339:b4187 [2]

FUNDED BY NIHR | National Institute for Health and Care Research

2. CTU Emissions

This module includes the following activities:

2.1. Energy consumption at CTU
2.2. Heating
2.3. Trial team commuting

2.1. Energy consumption at CTU (according to staff FTE)

Often, the carbon footprint associated with energy consumption by the CTU cannot be calculated directly because the space or building will be used for other trials not being appraised and non-trial activities. Emissions are therefore estimated based on average per person emissions and the FTE of the trial employees.

According to the UK Employment Destiny Guide, public sector office space is 12 m^2 per FTE. This is multiplied by 59 kWh/m^2, the median electricity intensity for offices, to produce a per person per year usage. The emissions attributed to CTU energy consumption can then be calculated by multiplying the per person usage by the electricity emission factor provided. The calculation is exemplified below.

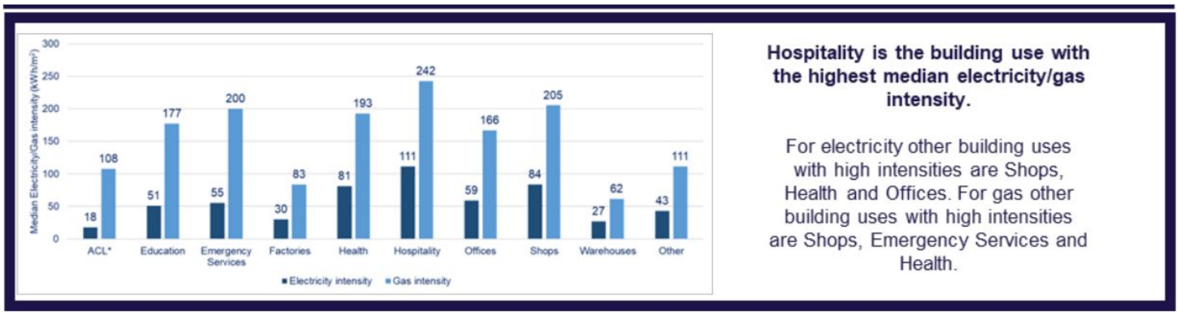

Calculation

$12m^2$ x 59 kWh/m^2 = 708 kWh per FTE per year

Electricity emission factor = 0.273

Name	Source	Year	Text	Unit	kg CO_2e	kg CO_2e	kg CO_2e	kg CO_2e	kg CO_2e/refrence flow unit
Electricity	Gov.UK	2021		kWh		0.21233	0.00489	0.05529	0.273

708 kWh x 0.273 = 193.3 kgCO_2e per FTE per year

Finally, multiply 193.3 kgCO_2e by the number of years and FTE applicable.

Assumption: According to the UK EMPLOYMENT DENSITY GUIDE, 3rd edition November 2015, office space per FTE is 12 m^2 (public sector) [8]

Office benchmark data source: The Non-Domestic National Energy Efficiency Data-Framework 2022 (England and Wales) (publishing.service.gov.uk)[9]

Electricity emission factor source: Greenhouse gas reporting: conversion factors 2021, GOV.UK [7]

FUNDED BY NIHR | National Institute for Health and Care Research

LIVERPOOL CLINICAL TRIALS CENTRE LCTC

ICR

2.2 Heating

For heating, the calculation follows the same method as above. The 12m^2 per person is multiplied by the office heating benchmark and then by the natural gas conversion factor provided below.

Office building heating benchmark: **167.27** (kWh/m^2)

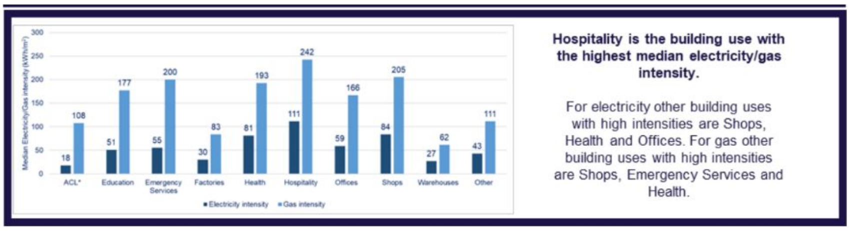

Natural gas conversion factor: **0.215**

Activity	Referen	EF	Comment	Refrence flow	Scope 1/Direc	Scope 2	Scope 3 T&D	Scope 3 WTT	Total Carbon intensity
Name	Source	Year	Text	Unit	kg CO_2e	kg CO_2e	kg CO_2e	kg CO_2e	kg CO_2e/refrence flow unit
Electricity	Gov.UK	2021		kWh		0.21233	0.00489	0.05529	0.273
Burning (hetaing) oil	Gov.UK	2021		litres	2.54014			0.52807	3.068
Natural gas	Gov.UK	2021		kWh (Gross CV)	0.18316			0.03135	0.215
LPG	Gov.UK	2021		litres	1.55709			0.18383	1.741

Calculation

- 12 m^2 x 167.27 kWh/m^2 = 2007.24 kWh per FTE per year
- 2007.24 kWh x 0.215 = 431.56 kg CO_2e per FTE per year

Multiply 431.56 kg CO_2e by the number of years and FTE applicable

Assumption: If the heating source is unknown, assume that the heating source is natural gas.

Assumption: According to the UK EMPLOYMENT DENSITY GUIDE, 3rd edition November 2015, office space per FTE is 12 m^2 public sector. [8]

Office benchmark data source: The Non-Domestic National Energy Efficiency Data-Framework 2022 (England and Wales) (publishing.service.gov.uk) [9]

Natural gas emission factor source: Greenhouse gas reporting: conversion factors 2021, GOV.UK [7]

Homeworking

Homeworking (office equipment + heating)	per FTE Working Hour	0.34075

Multiply the total number of FTE working hours by the conversion factor (0.34075) to calculate kgCO_2e (includes electricity use from office equipment and heating).

Emission factor source: Greenhouse gas reporting: conversion factors 2022, GOV.UK [10]

FUNDED BY NIHR | National Institute for Health and Care Research   LIVERPOOL CLINICAL TRIALS CENTRE LCTC   ICR

2.3. Trial team commuting

The emissions attributed to the trial team commuting is calculated using average commuting statistics. Using these statistics, the distance travelled by each mode of transport was calculated and multiplied by the relevant emission factor.

The average distance of a commuting journey was 8.8 miles (14km) in 2013/14. Multiplied by 220 commuting days, this results in a total distance of 6160km travelled per year.

Car (used for 68% of the 6160km distance) – 68% of 6160km = 4188.8km

4188.8 x 0.223 = 934.1 kgCO_2e

Large car petrol	Gov.UK	2021	km	0.27909	0.07833	0.357
Average car petrol	Gov.UK	2021	km	0.17431	0.04885	0.223
Dual purpose 4X4 petr[illegible]	Gov.UK	2021	km	0.21658	0.06074	0.277

Rail (used for 10% of the 6160km distance) = 616 km x 0.043 = 26.5 kgCO_2e

Coach	Gov.UK	2021	passenger.km	0.02684	0.00646	0.033
National rail	Gov.UK	2021	passenger.km	0.03549	0.00733	0.043
International rail	Gov.UK	2021	passenger.km	0.00446	0.00074	0.005
[illegible]	[illegible]	[illegible]	[illegible]	[illegible]	[illegible]	[illegible]

Bus (used for 6% of the 6160km distance): 369.6km x 0.127 = 46.9 kgCO_2e

11	Local bus (not London)	Gov.UK	2021	passenger.km	0.11774	0.02868	0.146
12	Local London bus	Gov.UK	2021	passenger.km	0.07718	0.01885	0.096
13	Average local bus	Gov.UK	2021	passenger.km	0.10227	0.02494	0.127
14	Coach	Gov.UK	2021	passenger.km	0.02684	0.00646	0.033
15	National rail	Gov.UK	2021	passenger.km	0.03549	0.00733	0.043

Other (used for 5% of the 6160km distance): Assume 3% cycling so zero emissions, 1% motorbike, 1% taxi

Motorbike: 61.6km x 0.145 = 8.9kgCO_2e

London Underground	Gov.UK	2021	passenger.km	0.02781	0.00461	0.032
Average motorbike	Gov.UK	2021	km	0.11355	0.03134	0.145
Small car diesel	Gov.UK	2021	km	0.13758	0.03344	0.171
Medium car diiesel	Gov.UK	2021	km	0.16496	0.04018	0.205

Taxi: 61.6 x 0.185 = 11.4

[illegible]	[illegible]	[illegible]	[illegible]	[illegible]	[illegible]	[illegible]	[illegible]
8	LPG	Gov.UK	2021	litres	1.55709	0.18383	1.741
9	Regular taxi	Gov.UK	2021	passenger.km	0.14876	0.03632	0.185
10	Black cab	Gov.UK	2021	passenger.km	0.20416	0.05	0.254
11	Local bus (not London)	Gov.UK	2021	passenger.km	0.11774	0.02868	0.146

For 1 FTE, emissions attributed to commuting for 1 year= 1027.8 kgCO_2e

Multiply by the number of years and FTE applicable

NB: The WTT (well-to-tank) has been included in the emission factors for all modes of transport and therefore does not need to be added manually in this case.

Assumptions:

- 220 days spent commuting. This was calculated by subtracting 10 days sick and 30 days paid leave from the 260 workdays in a year.
- 6160 km is the total distance an employee will commute per year. This was calculated by multiplying 28km (14km per commuting journey) by 220 days.

Sources:

- Transport Statistics Great Britain: 2021 - GOV.UK (www.gov.uk) [11]
- Commuting trends in England 1988 - 2015 (publishing.service.gov.uk) [12]
- Emission factor source: GOV.UK Greenhouse gas reporting, conversion factors 2021 [7]

For peer review only - http://bmjopen.bmj.com/site/about/guidelines.xhtml

FUNDED BY NIHR | National Institute for Health and Care Research — LIVERPOOL CLINICAL TRIALS CENTRE LCTC — ICR

3. Trial specific meetings and travel

This module includes the following activities:

3.1. Visits and travel to site
3.2. Travel to meetings
3.3. Hotel stays
3.4. Sustenance

3.1, 3.2. Visits and travel to site, travel to meetings

For business travel by bus, taxi and rail, the activity data is captured in passenger km (p.km). The number of passengers is multiplied by the distance travelled (km), then by the relevant emission factor.

Number of passengers x total distance (km) = p.km

p.km x suitable emission factor = (kg CO_2e)

Rail

Activity	Type	Unit	kg CO_2e	kg CO_2	kg CH_4	kg N_2O
Rail	National rail	passenger.km	0.03549	0.0351	0.00007	0.00032
	International rail	passenger.km	0.00446	0.00441	0.00002	0.00003
	Light rail and tram	passenger.km	0.02861	0.02832	0.00011	0.00018
	London Underground	passenger.km	0.02781	0.02753	0.0001	0.00018

For business travel you need to **add** the WTT (well-to-tank) value to get the final total. WTT can be calculated by multiplying the p.km used in the first calculation by the correlating WTT conversion factor provided below.

passenger.km x correlating WTT conversion factor

Activity	Type	Unit	kg CO_2e
WTT- rail	National rail	passenger.km	0.00892
	International rail	passenger.km	0.00116
	Light rail and tram	passenger.km	0.00745
	London Underground	passenger.km	0.00724

NB: Always use values relating to 'kgCO_2e'
NB: Distances can be calculated using google maps

Assumption: Distances calculated from CTU to destination

Emission factor source: Greenhouse gas reporting: conversion factors 2021, GOV.UK [7]

Emission factors for all modes can be found in the orange 'business travel' tabs of [conversion-factors-2021-full-set-advanced-users.xlsm (live.com)]

FUNDED BY NIHR | National Institute for Health and Care Research

LIVERPOOL CLINICAL TRIALS CENTRE LCTC

ICR

3.1, 3.2. Visits and travel to site, travel to meetings

For business travel by air, activity data is also captured in passenger km (p.km). The number of passengers is multiplied by the distance travelled (km), then by the relevant emission factor.

Number of passengers x total distance (km) = p.km

p.km x suitable emission factor = (kg CO_2e)

An emission factor must be chosen from the following categories:

- Domestic
- Short Haul International (≤3700 km) – Average/Economy/Business
- Long Haul International (>3700 km) – Average/Economy/Business
- International (travel between non-UK countries)

Activity	Haul	Class	Unit	kg CO_2e
Flights	Domestic, to/from UK	Average passenger	passenger.km	0.24587
	Short-haul, to/from UK	Average passenger	passenger.km	0.15353
		Economy class	passenger.km	0.15102
		Business class	passenger.km	0.22652
	Long-haul, to/from UK	Average passenger	passenger.km	0.19309
		Economy class	passenger.km	0.14787
		Premium economy class	passenger.km	0.23659
		Business class	passenger.km	0.42882
		First class	passenger.km	0.59147
	International, to/from non-UK	Average passenger	passenger.km	0.18362
		Economy class	passenger.km	0.140625
		Premium economy class	passenger.km	0.225
		Business class	passenger.km	0.40781
		First class	passenger.km	0.56251

For business travel you need to add the WTT (well-to-tank) value to get the final total. WTT can be calculated by multiplying the p.km used in the first calculation by the correlating WTT conversion factor provided below.

passenger.km x correlating WTT conversion factor

				With RF	Without RF
Activity	Haul	Class	Unit	kg CO_2e	kg CO_2e
WTT- flights	Domestic, to/from UK	Average passenger	passenger.km	0.02691	0.02691
	Short-haul, to/from UK	Average passenger	passenger.km	0.01681	0.01681
		Economy class	passenger.km	0.01654	0.01654
		Business class	passenger.km	0.0248	0.0248
	Long-haul, to/from UK	Average passenger	passenger.km	0.02114	0.02114
		Economy class	passenger.km	0.01619	0.01619
		Premium economy class	passenger.km	0.02591	0.02591
		Business class	passenger.km	0.04696	0.04696
		First class	passenger.km	0.06477	0.06477
	International, to/from non-UK	Average passenger	passenger.km	0.02011	0.02011
		Economy class	passenger.km	0.0154	0.0154
		Premium economy class	passenger.km	0.02464	0.02464
		Business class	passenger.km	0.04466	0.04466
		First class	passenger.km	0.06159	0.06159

NB: Always use values relating to 'kgCO_2e'
NB: Distances can be calculated using google maps
NB: 'With RF' values are recommended. RF (combustion and radiative forcing) includes the indirect and direct emissions

Assumption: travellers departed from the nearest airport to their place of work and flew directly to the airport of the city to which they were travelling.
Emission factor source: Greenhouse gas reporting: conversion factors 2021, GOV.UK [7]

Emission factors for all modes can be found in the orange 'business travel' tabs of conversion-factors-2021-full-set-advanced-users.xlsm (live.com)

For peer review only - http://bmjopen.bmj.com/site/about/guidelines.xhtml

FUNDED BY NIHR | National Institute for Health and Care Research

LIVERPOOL CLINICAL TRIALS CENTRE LCTC

ICR

3.2 Travel to meetings

Teleconferencing: for meetings which are conducted by teleconferencing, multiply the number of people and hours by the figure provided below.

A standard videoconferencing service has a carbon footprint of 157 g CO_2e/hr. When the video is switched off, videoconferencing uses 6.9 g CO_2e/hr.

Emission factor source: Turn off that camera during virtual meetings, environmental study says - Purdue University News [13]

3.3 Hotel Stays

To calculate the emissions attributed to hotel stays, the number of hotel rooms is multiplied by the length of stay (in number of nights) and by the conversion factor for the appropriate country to give the associated emissions.

Each country has a different emission factor. The emission factor for the UK is shown below, other countries can be found at conversion-factors-2021-full-set-advanced-users.xlsm (live.com)

	Country	Unit	kg CO_2e
Hotel stay	UK	Room per night	13.9
	UK (London)	Room per night	13.8

NB: A 'room per night' accounts for use of the room and does not differentiate for number of travellers staying in the room.

Source: Greenhouse gas reporting: conversion factors 2021, GOV.UK [7]

Alternatively, you may use a cost-based method by multiplying the total cost in the funding application by the emission factor below:

Cost (£) x 0.358 = kg CO_2e

Wholesale distribution	Gov.UK	2020	converted fron £	0.375
Retail distribution	Gov.UK	2020	converted fron £	0.277
Hotels, catering, pubs e	Gov.UK	2020	converted fron £	0.358
Railway transport[5]	Gov.UK	2020	converted fron £	0.678
Road transport[5]	Gov.UK	2020	converted fron £	0.690
Water transport[5]	Gov.UK	2020	converted fron £	1.428
Air transport[5]	Gov.UK	2020	converted fron £	2.089

Emission factor source: Gov.UK Government conversion factors for company reporting of greenhouse gas emissions 2012 - Annex 13 with consideration of 2020 inflation rates. [14]

3.4. Sustenance

For the carbon footprint associated with meeting lunches or hotel dinners, multiply the quantity by the relevant emission factor provided below.

Meeting lunches or hotel dinners (vegetarian) = 2.6 $kgCO_2e$ per meal per person

Meeting lunches or hotel dinners (meat) = 5.92 $kgCO_2e$ per meal per person

Emission factor source = WWF, 2018 Food_in_a_warming_world_report.PDF [15]

For peer review only

FUNDED BY NIHR | National Institute for Health and Care Research

LIVERPOOL CLINICAL TRIALS CENTRE LCTC

ICR

4. Intervention

This module includes guidance on the following different types of intervention. Pick the most applicable intervention type from:

4.1 Physical (IMP)
4.2 Clinical (Non-IMP)
4.3 Other (not captured above)

4.1 Physical (an IMP)

4.1.1 Movement of IMP from manufacturing site to distribution/packaging site

4.1.2 Movement of IMP from distribution/packaging site to participating sites or direct to participant

4.1.3 Materials required for the packaging and shipment of IMP

4.1.4 Destruction of overage

Assumptions: Calculations do not include manufacture of IMP.

4.2 Clinical (non-IMP)

NB: not all calculations will be relevant to all interventions.

4.2.1 Movement (shipment) of the intervention, or resources required to deliver the intervention
4.2.2 Materials required for the shipment of the intervention
4.2.3 Utilities required for delivery of the intervention
4.2.4 Activities or resources required/relating to delivery of the intervention

Assumptions: Calculations do not include manufacture of device/machinery/equipment delivering the intervention.

4.3 Other (Not captured above)

NB: not all calculations will be relevant to all interventions, depending on the specific intervention.

4.3.1 Movement of the intervention to the participant or participating site
4.3.2 Materials required for packaging and shipment of the intervention
4.3.3 Materials or resources required for delivery of the intervention
4.3.4 Travel required to facilitate delivery of the intervention

For peer review only - http://bmjopen.bmj.com/site/about/guidelines.xhtml

FUNDED BY NIHR | National Institute for Health and Care Research

LIVERPOOL CLINICAL TRIALS CENTRE LCTC

ICR

4.1. Physical (an IMP)

4.1.1.,4.1.2. Movement of intervention to participating site or direct to participant

For freight, please refer to section 1.2, 1.3, 1.4 for the calculation and emission factors. If the delivery is not ambient, follow the guidance provided below.

Refrigerated freight

Increase the total kgCO_2e associated with freight by 15% for samples transported at temperatures of 2-8 degrees.

Frozen (dry ice) freight

When calculating the carbon footprint of frozen shipments, make sure to consider the emissions attributable to the dry ice, both in terms of:

1. Weight - If the total weight of the posted package is not available, when estimating the weight of the sample and box, make sure to include the additional weight due to the dry ice (add/include in normal calculation of weight x distance x emission factor).
2. Emissions of dry ice manufacture: for 1 kg of dry ice, you need to account for 2.22 kg of liquid CO_2 using the Ecoinvent 2.2 data below. E.g., 2.22kg x 0.81605 = **1.81kg CO_2e per 1 kg dry ice** produced/used.

261	443	carbon black, at plant	chemicals	inorganics	kg	GLO	2.3658
262	444	carbon dioxide liquid, at plant	chemicals	inorganics	kg	RER	0.81605
263	445	carbon monoxide, CO, at plant	chemicals	inorganics	kg	RER	1.5539
6949	446	cerium concentrate, 60% cerium oxide, at plant	chemicals	inorganics	kg	CN	8.309

If the amount of dry ice used in frozen shipments is unknown, use an estimated 1kg of dry ice per sample box. Ensure this additional weight is included in the freight calculation and 1.81KgCO_2e in the total carbon footprint of the freight.

Assumption: use 1 kg of dry ice per sample box in the absence of established activity data.

Emission factor source: Consultation /estimation by Environmental Resource Management

4.1.3. Materials involved in the packaging and shipment of the intervention

Shipping boxes

For single use (SU) cold storage boxes multiply the number required by 25.2 kgCO_2e

For reusable cold storage boxes, multiply the number required by 2.2 kgCO_2e

Sample Average Shipping Box (85% ambient / 15% frozen)	1.34	kg CO_2e	per cold storage box	The International Journal of Life Cycle Assessment, Goellner et al. Vol 19, pp 611–619 (2014)
Sample Cold Storage Box Manufacture (SU)	25.2	kg CO_2e	per cold storage box	The International Journal of Life Cycle Assessment, Goellner et al. Vol 19, pp 611–619 (2014)
Sample Cold Storage Box Manufacture (Reuse)	2.2	kg CO_2e	per cold storage box	The International Journal of Life Cycle Assessment, Goellner et al. Vol 19, pp 611–619 (2014)

Emission factor source: The International Journal of Life Cycle Assessment, Goellner et al. Vol 19, pp 611-619 (2014) [16]

For cardboard: Kg (cardboard) x 0.821 = kgCO_2e

For polystyrene: kg (polystyrene) x 3.778 = kgCO_2e

Greenhouse gas reporting: conversion factors 2021, GOV.UK [7]

For peer review only - http://bmjopen.bmj.com/site/about/guidelines.xhtml

FUNDED BY NIHR | National Institute for Health and Care Research

LIVERPOOL CLINICAL TRIALS CENTRE LCTC

ICR

4.1.4. Destruction of overage

For the destruction of overage, such as the incineration of IMP, multiply the weight in kg of the material being destroyed by the emission factor provided below.

Kg of waste x 2.4252 = kgCO_2e

2958	disposal, hazardous waste, 25% water, to hazardous waste incineration	waste management	hazardous waste incineration	kg	CH	2.4252

Emission factor source: Ecoinvent, version 2.2, 2011 (CH = SWITZERLAND) [6]

4.2. Clinical (e.g. radiotherapy, device, surgery)

NB: not all calculations will be relevant to all interventions. **This section of the method will be further developed as we carbon footprint more trials, so please inform us if your protocol specifies an activity that has not been included, and we will help to determine the associated carbon footprint.**

4.2.1 Movement of intervention, or materials required to deliver the intervention

Please refer to section 1.2 and 4.1.1, 4.1.2.

4.2.2 Materials involved in the shipment of the intervention

Please refer to section 4.1.3.

4.2.3 Utilities required for delivery of the intervention

Please refer to section 7.3 to calculate the emissions attributed to hospital utilities if required to deliver the intervention.

4.2.4 Activities or resources required/relating to delivery of the intervention

Please refer to section 7.2 for consumables, surgery and other activities that may be relevant to the delivery of the intervention, but please take care to avoid double counting.

To calculate the emissions attributed to incineration, please refer to section 4.1.4.

4.3. Other

NB: not all calculations will be relevant to all interventions. **This section of the method will be further developed as we carbon footprint more trials, so please inform us if your protocol specifies an activity that has not been included, and we will help to determine the associated carbon footprint.**

4.3.1 Movement of intervention, or materials required to deliver the intervention

Please refer to section 1.2 and 4.1.1, 4.1.2.

For peer review only - http://bmjopen.bmj.com/site/about/guidelines.xhtml

FUNDED BY NIHR | National Institute for Health and Care Research

LIVERPOOL CLINICAL TRIALS CENTRE LCTC

ICR

4.3.2 Materials required for packaging and shipment of the intervention

Please refer to section 4.1.3

4.3.3 Materials or resources required for delivery of the intervention

For printing and paper, please refer to section 1.1.

4.3.4 Travel required to facilitate delivery of the intervention

Please refer to section 3.1, 3.2 for travel.

For peer review only

FUNDED BY NIHR | National Institute for Health and Care Research

LIVERPOOL CLINICAL TRIALS CENTRE LCTC

ICR

5 Data collection and exchange

This module includes the following activities:

- **5.1. Data collection and query exchange between CTU and sites**
- **5.2. Data sent direct from participants to CTU or participating sites**
- **5.3. Data from labs to CTU**
- **5.4. Data from other collaborators to CTU**

NB: Analysis of data does not need to be calculated separately, it is covered by the emissions attributed to trial staff FTE in “CTU emissions” and by calculations within “Data Collection and exchange”.

5.1. Data collection and query exchange between CTU and sites

CRFs

For postage of paper CRFs, please refer to section 1.2 (freight).

Web-based data entry at sites, e.g. CRF completion, will be accounted for in the time a hospital worker spends on the trial and the carbon footprint of the trial databases (See 5.2).

Scans copied to CD

To estimate the emissions attributed to copying patient scans to a CD, add the carbon footprint of CD manufacture to the carbon footprint of computer use.

The carbon footprint of manufacturing a CD = 0.83 kg $C0_2e$ per CD (833 g $C0_2e$)

Source: Journal of Industrial Ecology, “The Energy and Climate Change Impacts Of Different Music Delivery Methods”. Weber et al. Vol 14, Issue 5, pg. 754-769 (2010) [17]

The carbon footprint of copying the scans on to a CD using a computer = 0.18079 kg $C0_2e$ per hour

1269	use, computer, desktop with LCD monitor, active mode	electronics	services	h	RER	0.18079

Emission factor source: Ecoinvent, version 2.2, 2011 [6]

Email traffic

An email without an attachment = 10g CO_2e. Double this for an email with a one-megabyte attachment.

NB: this is an estimate of all emails exchanged between CTU and participating sites throughout the study lifetime, including data query resolution emails.

Source: Carbon footprint of your emails | mail.com blog [18]

For peer review only - http://bmjopen.bmj.com/site/about/guidelines.xhtml

5.1. (continued)

Data collection via electronic trial databases/systems

The combination of transmitting data and storing it in a data centre requires between 3 to 7 kWh per gigabyte[19]. Therefore multiply 5 kWh by the electricity emission factor (0.273) to calculate the kg CO_2e per GB per year.

5 kwh x 0.273 = 1.365 kg CO_2e per GB per year

Assumption: data storage requires 5 kWh.

Source: Carbon and the Cloud. Hard facts about data storage. | by Stanford Magazine | Stanford Magazine | Medium[19]

5.3. Data sent direct from participants to CTU/participating sites

For paper questionnaires, please refer to section 1.1. for the carbon footprint of producing the materials and section 1.2. for postage (freight).

Electronic questionnaire

For completion of an electronic questionnaire, you must account for both the use of a device to complete the questionnaire and the carbon footprint of data storage and transmission associated with web surfing.

For completion using a **desktop computer**: 0.18079 kg CO_2e per hour

1269	use, computer, desktop with LCD monitor, active mode	electronics	services	h	RER	0.18079

For completion using a **laptop**: 0.028719 kg CO_2e per hour

1284	use, computer, laptop, active mode	electronics	services	h	RER	0.028719

Computer and laptop emission factor source: Ecoinvent, version 2.2, 2011 [6]

For completion using a **tablet**: 0.027397 kg CO_2e per hour

For completion using a **smartphone**: 0.015068 kg CO_2e per hour

Tablet and smartphone emission factor source: Examining the Carbon Footprint of Devices - Sustainable Software (microsoft.com) [20]

Web surfing = 9.441 g CO2e/hr (10 mins = 1.57 g CO_2e)

Source: Resources, Conservation and Recycling, "The overlooked environmental footprint of increasing Internet use". Obringer et al. Vol 167 (2021) [21]

5.4. Data from labs to CTU

For data collection via electronic trial database systems estimate 1.365 kgCO_2e per GB per year.

Source: Carbon and the Cloud. Hard facts about data storage. | by Stanford Magazine | Stanford Magazine | Medium[19]

FUNDED BY NIHR | National Institute for Health and Care Research

LIVERPOOL CLINICAL TRIALS CENTRE LCTC

ICR

5.5. Data from other collaborators to CTU

Data linkage

For data linkage, multiple the cost spent by the emission factor provided below.

Computer services – 0.149 x unit (£) = $kgCO_2e$

42	Real estate activities	Gov.UK	2020	converted from £					0.085
43	Renting of machinery e	Gov.UK	2020	converted from £					0.232
44	Computer services	Gov.UK	2020	converted from £					0.149
45	Research and developn	Gov.UK	2020	converted from £					0.216
46	Legal, consultancy and	Gov.UK	2020	converted from £					0.121

Emission factor source: Gov.UK Government conversion factors for company reporting of greenhouse gas emissions 2012 - Annex 13 with consideration of 2020 inflation rates. [13]

For peer review only

FUNDED BY NIHR | National Institute for Health and Care Research

LIVERPOOL CLINICAL TRIALS CENTRE LCTC

ICR

6 Trial supplies and equipment

This module includes the following activities:

- **6.1. Equipment used by CTU**
- **6.2. Equipment and supplies used by participating sites supplied by CTU**
- **6.3. Equipment and supplies provided to participants specifically for the trial**

6.1. Equipment used by CTU

For office machinery and computers purchased for the trial, multiply the cost by the emission factor provided below.

£ x 0.387 = $kgCO_2e$

17	Machinery and equipme	Gov.UK	2020 converted from £	0.512
18	Office machinery and co	Gov.UK	2020 converted from £	0.387
19	Electrical machinery	Gov.UK	2020 converted from £	0.452

Emission factor source: Gov.UK Government conversion factors for company reporting of greenhouse gas emissions 2012 - Annex 13 with consideration of 2020 inflation rates. [13]

6.2. Equipment and supplies used by participating sites supplied by CTU

For the shipment of equipment to participating sites, please refer to section 1.2.

For the use of a centrifuge, please refer to section 9.2., for a fridge or freezer please refer to section 9.3.

Please inform us if your protocol specifies any equipment or supplies that have not been included and we will help to determine the associated carbon footprint.

6.3. Equipment and supplies provided to participants specifically for the trial

Smartphone: For a smartphone, account for 55 $kgCO_2e$ from manufacture and add 5.5 $kgCO_2e$ per year of usage.

Source: Examining the Carbon Footprint of Devices - Sustainable Software (microsoft.com) [19]

Tablet: For a tablet, account for 119 $kgCO_2e$ from manufacture and add 10kg CO_2e per year of usage. Assume a maximum lifetime of 3 years, therefore 30 kg CO_2e is the total possible carbon footprint that can be attributed to use.

Source: Examining the Carbon Footprint of Devices - Sustainable Software (microsoft.com) [19]

Wearables/smart watch: For a smart watch, account for 30.1 kg CO_2e for manufacture and add 1.633 kg CO_2e per year of usage. Assume a maximum lifetime of 3 years, therefore 4.9 kg CO_2e is the total possible carbon footprint that can be attributed to use.

Source: Apple Watch SE Product Environmental Report [22]

To calculate the carbon footprint associated with shipment of the devices, please refer to section 1.2.

For peer review only - http://bmjopen.bmj.com/site/about/guidelines.xhtml

FUNDED BY NIHR | National Institute for Health and Care Research

LIVERPOOL CLINICAL TRIALS CENTRE LCTC

ICR

7 Trial specific patient assessments

This module includes the following activities:

7.1. Travel of patients for study in visits in addition to standard of care (eligibility/screening assessments, trial-specific assessments and procedures)
7.2. Materials and activities required for study assessments in addition to standard of care
7.3. Utilities required for study assessments according to trial staff FTE

7.1. Travel of patients for study in visits that are in addition to standard of care (eligibility/screening assessments, trial-specific assessments and procedures)

Patient travel to elective care (e.g. Hospital)

1 journey by patient associated with attendance at elective care = 2.9 kg CO_2e

Therefore 1 visit to elective care (2 journeys – out and back) = 5.8 kgCO_2e

Patient travel to primary care (e.g. GP)

Emissions associated with attendance at GP surgery (journey out and back) = 1.12 kgCO_2e

Emission factors source: SHC care pathway calculator [23]

7.2. Materials and activities required for study assessments that are in addition to standard of care

This includes everything that happens to the patient in the protocol schedule of assessments which is over and above routine care. This is not an exhaustive list; please inform us if your protocol specifies an activity that has not been included, and we will help to determine the associated carbon footprint.

Surgery

A 1-hour surgery = 53 kg CO_2e

A 30-minute surgery = 26.5 kg CO_2e

Emission factor source: SHC care pathway calculator [23]

Bed days

Low intensity (general ward) = 37.9 kg CO_2e

High intensity (ICU) = 103 kg CO_2e

Emission factor source: SHC care pathway calculator [23]

Scans

1 MRI = 24.7 kg CO_2e

Emission factor source: SHC care pathway calculator [23]

1 CT scan = 9.2 kg CO_2e, 1 CXR = 0.8 kgCO_2e

Emission factor source: The carbon footprint of hospital diagnostic imaging in Australia (thelancet.com) [24]

For peer review only - http://bmjopen.bmj.com/site/about/guidelines.xhtml

FUNDED BY NIHR | National Institute for Health and Care Research

7.2. Materials and activities required for study assessments (continued)

Radiotherapy

To calculate the carbon footprint of radiotherapy, multiply the total treatment power in kWh by the electricity emission factor.

Full course of breast radiotherapy (15 sessions) **= 5.7 kgCO_2e**

Calculation: 1.4 kWh x 0.273 x 15 = 5.7 kgCO_2e

Full course prostate radiotherapy (20 sessions) = **15.3 kgCO_2e**

Calculation: 2.8 kWh x 0.273 x 20 = 15.3 kgCO_2e

Assumption: 15 sessions required for breast radiotherapy. Source: Radiotherapy for breast cancer - Macmillan Cancer Support [25]

Assumption: 20 sessions required for prostate radiotherapy. Source: Radiotherapy for advanced prostate cancer | Prostate Cancer UK [26]

Benchmark data source: PO-1058 Estimating the carbon footprint of the radiotherapy pathway and changes in response to COVID-19 main.pdf (nih.gov) [27]

Emission factor source: GOV.UK Greenhouse gas reporting, conversion factors 2021 [7]

Consumables

For a trial appointment where consumables such as gloves are required, account for **0.30 kgCO_2e per patient per appointment.**

Emission factor source: SHC care pathway calculator guidance GP consultation module, 2015 [28]

Blood investigations

CO_2 e emissions for haematology tests:
- 82 g/test (95% CI, 73-91 g/test) for coagulation profile
- 116 g/test (95% CI, 101-135 g/test) for full blood examination.

CO_2 e emissions for biochemical tests:
- 0.5 g/test CO_2 e (95% CI, 0.4-0.6 g/test) for C-reactive protein (low because typically ordered with urea and electrolyte assessment)
- 49 g/test (95% CI, 45-53 g/test) for arterial blood gas assessment
- 99 g/test (95% CI, 84-113 g/test) for urea and electrolyte assessment.

NB: These emissions include the materials and consumables required for sample collection, phlebotomy and analysis, as well as power consumption by pathology analysers.

Emission factor sourced from: The carbon footprint of pathology testing. Scott McAlister, Alexandra L Barratt, Katy JL Bell and Forbes McGain. Med J Aust 2020; 212 (8): 377-382. Published online: 4 May 2020 **The carbon footprint of pathology testing - McAlister - 2020 - Medical Journal of Australia - Wiley Online Library** [29]

For peer review only - http://bmjopen.bmj.com/site/about/guidelines.xhtml

FUNDED BY NIHR | National Institute for Health and Care Research

LIVERPOOL CLINICAL TRIALS CENTRE LCTC

ICR

7.3. Utilities required for study assessments that are in addition to standard of care

The carbon footprint associated with energy consumption by hospital staff cannot be calculated directly because a hospital and the equipment and staff within in it are used for many other non-trial activities. Emissions are therefore estimated based on average per person emissions and the FTE of the trial hospital staff.

This is calculated by multiplying the average space occupied by a hospital staff member (16.5 m^2) by the kWh used per m^2 of a hospital (81 kWh/m^2). The kWh per FTE per year is then multiplied by the electricity emission factor provided below to calculate the carbon footprint attributed per hospital staff FTE. Finally, multiply by the number of years and FTE applicable**.** The calculation is exemplified below.

Calculation

16.5 m^2 x 81 kWh/m^2 = 1336.5 kWh per FTE per year

- Electricity emission factor = 0.273

1336.5 kWh x 0.273 = **364.9 kgCO_2e per FTE per year**

Multiply 364.9 kgCO_2e by the number of years and FTE applicable - Use the trial SOECAT or the costing included in the initial funding application (if predates use of SOECAT) to establish the FTE or total number of hours required by hospital staff for the trial. If using the number of hours, please follow the below method to establish the FTE required for the calculation.

Number of hours in SOECAT / 1762.5 = FTE required for trial.

e.g. if 449.25 total hospital staff hours required for trial.

449.25 / 1762.5 = 0.25. Therefore 25% of 1 hospital staff FTE required

364.9 kgCO_2e x 0.25 = 91.2 kgCO_2e

Assumption: The FTE of a nurse/hospital staff is 1762.5 hours. The standard full-time working week for NHS staff is 37.5 hours (1950 hours - 35 days a year off = 1762.5 hours).

Assumption: each health care professional occupies 16.5m^2 room, source HBN 12 (england.nhs.uk) page 32 [30]

Hospital benchmark data source: The Non-Domestic National Energy Efficiency Data-Framework 2022 (England and Wales) (publishing.service.gov.uk). [9]

Electricity emission factor source: Greenhouse gas reporting: conversion factors 2021, GOV.UK [7]

FUNDED BY NIHR | National Institute for Health and Care Research

LIVERPOOL CLINICAL TRIALS CENTRE LCTC

ICR

7.3. Utilities required for study assessments that are in addition to standard of care (continued)

Heating

For heating, the calculation follows the same method as above. The 16.5m^2 per person is multiplied by the hospital heating benchmark and then by the natural gas conversion factor provided below.

Hospital building heating benchmark: **193.28** kWh per year per sqm floor area (kWh/m^2)

Natural gas conversion factor: **0.215**

Activity	Referen	EF	Comment	Refrence flow	Scope 1/Direc	Scope 2	Scope 3 T&D	Scope 3 WTT	Total Carbon intensity
Name	Source	Year	Text	Unit	kg CO_2e	kg CO_2e	kg CO_2e	kg CO_2e	kg CO_2e/refrence flow unit
Electricity	Gov.UK	2021		kWh		0.21233	0.00489	0.05529	0.273
Burning (hetaing) oil	Gov.UK	2021		litres	2.54014			0.52807	3.068
Natural gas	Gov.UK	2021		kWh (Gross CV)	0.18316			0.03135	0.215
LPG	Gov.UK	2021		litres	1.55709			0.18383	1.741

16.5 x 193.28 = 3189.1 kWh per FTE per year
3189.1 x 0.215 = 685.7 kgCO_2e per FTE per year

Multiply 685.7 kgCO_2e by the number of years and FTE applicable

Assumption: each health care professional occupies 16.5m^2 room, source HBN 12 (england.nhs.uk) page 32 [30]

Assumption: If the heating source is unknown, assume that the heating source is natural gas.

Hospital benchmark data source: The Non-Domestic National Energy Efficiency Data-Framework 2022 (England and Wales) (publishing.service.gov.uk) [9]

Natural gas emission factor source: Greenhouse gas reporting: conversion factors 2021, GOV.UK [7]

For peer review only

FUNDED BY NIHR | National Institute for Health and Care Research

8 Samples

This module includes the following activities:

8.1. Materials involved
8.2. Movement of sample kits from manufacturer to CTU
8.3. Movement of sample kits from CTU/distributor to participating sites
8.4. Movement of sample from participating sites to central laboratory

8.1. Material involved

The emissions attributed to sample collection consumables for common blood tests are included in the blood investigations section of 7.2.

Below is a list of commonly used materials used in sample collection and distribution and their equivalent emission factor. Multiply the kg of the material by the yellow highlighted number (the emission factor) to produce a kgCO_2e.

Paper and board: board	Gov.UK	2021	kg	0.821
Paper and board: mixed	Gov.UK	2021	kg	0.881
Paper and board: paper	Gov.UK	2021	kg	0.919
Plastics: average plastics	Gov.UK	2021	kg	3.116
Plastics: average plastic film	Gov.UK	2021	kg	2.574
Plastics: average plastic rigid	Gov.UK	2021	kg	3.277
Plastics: HDPE (incl. forming)	Gov.UK	2021	kg	3.270
Plastics: LDPE and LLDPE (incl. forming)	Gov.UK	2021	kg	2.601
Plastics: PET (incl. forming)	Gov.UK	2021	kg	4.032
Plastics: PP (incl. forming)	Gov.UK	2021	kg	3.105
Plastics: PS (incl. forming)	Gov.UK	2021	kg	3.778
Plastics: PVC (incl. forming)	Gov.UK	2021	kg	3.413
Glass	Gov.UK	2021	kg	1.403

Example: 100 x 10ml PET blood tubes (such as Streck), weight 5kg.

5kg x 4.032 (emission factor for PET) = 20.2 kgCO_2e

Example: 100 slide mailing containers made of polypropene, weight 1.02 kg

1.02kg x 3.105 = 3.2 kgCO_2e

Emission factor source: Greenhouse gas reporting: conversion factors 2021, GOV.UK [7]

For 8.2., 8.3., and 8.4, please refer to section 1.2. for freight and 4.1.1 for refrigerated or frozen freight.

For peer review only - http://bmjopen.bmj.com/site/about/guidelines.xhtml

FUNDED BY NIHR | National Institute for Health and Care Research

LIVERPOOL CLINICAL TRIALS CENTRE LCTC

ICR

9 Laboratory

This module includes the following activities:

- **9.1. Emissions attributed to lab utilities according to staff FTE**
- **9.2. Materials/equipment/consumables used in processing and analysis of samples**
- **9.3. Storage of samples e.g., utilities and ultra-low temperature freezer**

9.1. Laboratory utilities according to staff FTE

The carbon footprint associated with energy consumption by laboratory staff cannot be calculated directly because a laboratory, and the equipment and staff within in it, are used for many other non-trial activities. Emissions are therefore estimated based on average per person emissions and the FTE of the trial laboratory staff.

This is calculated by multiplying the average space occupied by a laboratory staff member (40 m^2) by the kWh used per m^2 of a laboratory (160 kWh/m^2). The kWh per FTE per year is then multiplied by the electricity emission factor provided below to calculate the carbon footprint attributed to 1 FTE for 1 year. Finally, multiply by the number of years and FTE applicable**.** The calculation is exemplified below. Use the trial SOECAT or the costing included in the initial funding application (if predates use of SOECAT) to establish the FTE or total number of hours required by laboratory staff.

Calculation

40m^2 x 160kWh = 6400 kWh per FTE per year

- Electricity emission factor = 0.273

Name	Source	Year	Text	Unit	kg CO_2e	kg CO_2e	kg CO_2e	kg CO_2e	kg CO_2e/refrence flow unit
Electricity	Gov.UK	2021		kWh		0.21233	0.00489	0.05529	0.273

6400 kWh x 0.273 = 1747.2 kgCO_2e per FTE per year

Multiply 1747.2 kgCO_2e by the number of years and FTE applicable

Heating

For heating, the calculation follows the same method as above. The 40m^2 per person is multiplied by the laboratory heating benchmark and then by the natural gas conversion factor provided below.

Laboratory fossil thermal typical benchmark: **160** kWh per year per sqm floor area (kWh/m^2)

Natural gas conversion factor**: 0.215**

Activity	Referen	EF	Comment	Refrence flow	Scope 1/Direc	Scope 2	Scope 3 T&D	Scope 3 WTT	Total Carbon intensity
Name	Source	Year	Text	Unit	kg CO_2e	kg CO_2e	kg CO_2e	kg CO_2e	kg CO_2e/refrence flow unit
Electricity	Gov.UK	2021		kWh		0.21233	0.00489	0.05529	0.273
Burning (hetaing) oil	Gov.UK	2021		litres	2.54014			0.52807	3.068
Natural gas	Gov.UK	2021		kWh (Gross CV)	0.18316			0.03135	0.215
LPG	Gov.UK	2021		litres	1.55709			0.18383	1.741

40m^2 x 160kWh = 6400 kWh per FTE per year

6400 kWh x 0.215 = 1376 kgCO_2e per FTE per year

Multiply 1376 kgCO_2e by the number of years and FTE applicable

Assumption: For R&D, 40m^2 required per FTE according to UK EMPLOYMENT DENSITY GUIDE, 3rd edition November 2015 [8]

Assumption: If the heating source is unknown, assume that the heating source is natural gas.

Laboratory benchmark data source: Health Technical Memorandum 07-02: EnCO2de 2015 – making energy work in healthcare (england.nhs.uk) [31]

Emission factor source: Greenhouse gas reporting: conversion factors 2021, GOV.UK [7]

For peer review only - http://bmjopen.bmj.com/site/about/guidelines.xhtml

FUNDED BY NIHR | National Institute for Health and Care Research

LIVERPOOL CLINICAL TRIALS CENTRE LCTC

ICR

9.2. Materials/equipment/consumables used in processing and analysis of samples

To avoid double counting, use of equipment will be included in lab staff FTE if calculated.

However, if the trial does not involve a central lab, but there is still sample processing on site, please see below. For storage of samples, please see section 9.3.

To calculate the emissions of a piece of equipment, multiply the power consumption in Watts by hours used to get a kWh value. Depending on the equipment, this can often be found in the specifications of a listed product. Finally multiply kWh by the electricity emission factor (0.273).

Example: use of a 310-Watt centrifuge for 15 minutes.

310 Watts x 0.25 (hours) = 77.5 kWh

- 77.5 kWh x 0.273 = 21.2 kg CO_2e

Consider the centrifuge capacity and multiply by the number of uses required.

Emission factor source: Greenhouse gas reporting: conversion factors 2021, GOV.UK [7]

9.3. Storage and destruction of biological samples

For storage of samples in a fridge/-20 freezer or an ultra-low temperature freezer, the kWh usage per day is multiplied by 365 to calculate the kWh per year. This is then multiplied by the electricity emission factor and the number of years the samples are stored. However, you will need to make an assumption about the amount of space in the freezer that the trial samples take up.

Fridge/-20 freezer

- 3kWh/day x 365 days = 1095 kWh per year
- 1095 x 0.273 = 298.9 kgCO_2e per year
- Multiply by number of years stored
- This total is for the whole fridge/-20 freezer for 1 year.

-80 freezer

- 22kWh/day x 365 days = 8030 kWh per year
- 8030 x 0.273 = 2192.2 kgCO_2e per year
- Multiply by number of years stored

This total is for the whole freezer for 1 year. You will need to make an assumption about the amount of space in the freezer that the trial samples take up.

Example: if the samples take up a third of the freezer space - 2192.2 kg CO_2e x 0.333 = 723.4 kg CO_2e

Assumptions: A -80°C freezer uses 22 kWh/day, a -20°C freezer uses 3 kWh/day **(**Source: Did You Know? - International Laboratory Freezer Challenge) [32]

Emission factor source: Greenhouse gas reporting: conversion factors 2021, GOV.UK [7]

For peer review only - http://bmjopen.bmj.com/site/about/guidelines.xhtml

10 Trial close out

This module includes the following activities:

10.1. Storage and archiving of essential trial documentation and data
10.2. Storage and destruction of biological samples
10.3. Return of equipment and supplies from participating sites to CTU

For peer review only

FUNDED BY NIHR | National Institute for Health and Care Research · LIVERPOOL CLINICAL TRIALS CENTRE LCTC · ICR

10.1. Storage and archiving of essential trial documentation and data

For the storage of archived documentation, estimate the m^2 required for archiving. This estimate will then be multiplied by the most suitable energy benchmark from the list provided below; choose the type of building most similar to the one the documents are stored in e.g. office/lab/warehouse/health building. Finally multiply by the electricity emission factor.

Energy benchmarks:

- office = 59 kWh/m^2
- laboratory = 160 kWh/m^2
- warehouse = 27 kWh/m^2
- health building = 81 kWh/m^2

Calculation
m^2 required x benchmark = kWh
- Electricity conversion factor = 0.273

kWh x 0.273 = kg CO_2e for 1 year of storage

Multiply by number of years necessary.

Heating
For heating, use the same method as above. Estimate the m^2 used and multiply by the corresponding heating benchmark (i.e., if 'laboratory' was used above then select the same for this calculation). Multiply by the emission factor for natural gas provided below.
Benchmarks:

- office = 167.27 kWh/m^2
- laboratory = 160 kWh/m^2
- warehouse = 62 kWh/m^2
- health building = 193.28 kWh/m^2

Calculation
m^2 required x benchmark = kWh
- Natural gas conversion factor: **0.215**

kWh x 0.215 = kgCO_2e for 1 year of storage

Multiply by number of years necessary.

Assumption: If the heating source is unknown, make assumption the heating source is natural gas.

Benchmark data source: The Non-Domestic National Energy Efficiency Data-Framework 2022 (England and Wales) (publishing.service.gov.uk) [9]

Emission factor source: Greenhouse gas reporting: conversion factors 2021, GOV.UK [7]

For peer review only - http://bmjopen.bmj.com/site/about/guidelines.xhtml

FUNDED BY NIHR | National Institute for Health and Care Research

LIVERPOOL CLINICAL TRIALS CENTRE LCTC

ICR

10.1. Storage and archiving of essential trial documentation and data (continued)

For electronic data or documentation storage, estimate 1.365 kgCO_2e per GB per year.

Source: Carbon and the Cloud. Hard facts about data storage. | by Stanford Magazine | Stanford Magazine | Medium[19]

10.2. Storage and destruction of biological samples

See section 9.3. for storage of refrigerated or frozen samples.

See section 10.1 for storage of ambient samples.

10.3. Return of equipment and supplies from participating sites to CTU

See section 1.2. for freight.

For peer review only

FUNDED BY NIHR | National Institute for Health and Care Research

LIVERPOOL CLINICAL TRIALS CENTRE LCTC

ICR

References

[1] Sustainable Trials Study Group. Towards sustainable clinical trials. *BMJ* 2007;334:671

[2] Lyle K, Dent L, Bailey S, et al. Carbon cost of pragmatic randomised controlled trials: retrospective analysis of sample of trials. *BMJ* 2009;339:b4187

[3] Making clinical trials sustainable [Internet]. The Sustainable Healthcare Coalition. [cited 2023 May 11]. Available from: https://shcoalition.org/clinical-trials/

[4] Climate change and health [Internet]. World Health Organization. 2021 October 30 [cited 2023 May 11]. Available from: https://www.who.int/news-room/fact-sheets/detail/climate-change-and-health

[5] What is an emission factor? [Internet]. Climfoot-project.eu. [cited 2023 May 11]. Available from: https://climfoot-project.eu/en/what-emission-factor

[6] Ecoinvent, version 2.2. 2011. Available from: https://ecoinvent.org/the-ecoinvent-database/data-releases/ecoinvent-version-2/

[7] Greenhouse gas reporting: conversion factors [Internet]. GOV.UK 2021. [cited 2023 May 11]. Available from: https://www.gov.uk/government/publications/greenhouse-gas-reporting-conversion-factors-2021

[8] EMPLOYMT DENSITY GUIDE (3rd Edition) [Internet]. Homes & Communities Agency. November 2015 [cited 2023 May 11]. Available from: https://www.kirklees.gov.uk/beta/planning-policy/pdf/examination/national-evidence/NE48_employment_density_guide_3rd_edition.pdf

[9] The Non-Domestic National Energy Efficiency Data-Framework (England and Wales) [Internet]. Department for Business, Energy & Industrial Strategy. 2022 [cited 2023 May 11]. Available from: https://assets.publishing.service.gov.uk/government/uploads/system/uploads/attachment_data/file/1086903/non_domestic_need_data_framework_2022.pdf

[10] Greenhouse gas reporting: conversion factors 2022 [Internet]. Gov.uk. 2022 [cited 2023 May 11]. Available from: https://www.gov.uk/government/publications/greenhouse-gas-reporting-conversion-factors-2022

[11] Transport Statistics Great Britain: 2021 [Internet]. GOV.UK (www.gov.uk). 2021 December 16 [cited May 11 2023]. Available from: https://www.gov.uk/government/statistics/transport-statistics-great-britain-2021/transport-statistics-great-britain-2021

[12] Commuting trends in England 1988-2015 [Internet]. GOV.UK. 2017 November 7 [cited 2023 May 11]. Available from: https://www.gov.uk/government/publications/commuting-trends-in-england-1988-to-2015

[13] Kayla Wiles. Turn off that camera during virtual meetings, environmental study says [Internet]. Purdue University News. 2021 January 14 [cited 2023 May 11]. Available from: https://www.purdue.edu/newsroom/releases/2021/Q1/turn-off-that-camera-during-virtual-meetings,-environmental-study-says.html

[14] Gov.UK Government conversion factors for company reporting of greenhouse gas emissions 2012 – Annex 13 (with consideration of 2020 inflation rates)

[15] WWF. 2018. Food in a warming world [Internet]. [cited 2023 May 11]. Available from: https://www.wwf.org.uk/sites/default/files/2018-03/Food_in_a_warming_world_report.PDF?source=post_page---------------------------

[16] Goellner et al. The International Journal of Life Cycle Assessment. Vol 19, pp 611-619 (2014)

[17] Weber et al. Journal of Industrial Ecology, "The Energy and Climate Change Impacts Of Different Music Delivery Methods". Vol 14, Issue 5, pg. 754-769 (2010)

[18] What's the carbon footprint of an email? [Internet]. Mail.com blog. 202 April 21 [cited 2023 May 11]. Available from: https://www.mail.com/blog/posts/email-carbon-footprint/9/

[19] Justin Adamson. Carbon and the Cloud. Hard facts about data storage [Internet]. Stanford Magazine. 2017 May 15 [cited 2023 May 11]. Available from: https://medium.com/stanford-magazine/carbon-and-the-cloud-d6f481b79dfe

[20] Srilatha Manne. Examining the Carbon Footprint of Devices [Internet]. Microsoft.com. 2020 November 23 [cited 2023 May 11. Available from: https://devblogs.microsoft.com/sustainable-software/examining-the-carbon-footprint-of-devices/

[21] Obringer, R., Rachunok, B., Maia-Silva, D., Arbabzadeh, M., Nateghi, R., & Madani, K. (2021). The overlooked environmental footprint of increasing Internet use. *Resources, Conservation and Recycling*, *167*, [105389]. https://doi.org/10.1016/j.resconrec.2020.105389

[22] Apple watch SE. Product Environmental Report – Apple [Internet]. [cited 2023 May 11]. Available from: https://www.apple.com/by/environment/pdf/products/watch/Apple_Watch_SE_PER_sept2020.pdf

[23] Care pathways carbon footprint calculator [Internet]. Sustainable Healthcare Coalition. [cited 2023 May 11]. Available from: https://shcpathways.org/full-calculator/

[24] McAlister S, McGain F, Petersen M, Story D, Charlesworth K, Ison G, Barratt A. The carbon footprint of hospital diagnostic imaging in Australia. Lancet Reg Health West Pac. 2022 May 3;24:100459. doi: 10.1016/j.lanwpc.2022.100459. PMID: 35538935; PMCID: PMC9079346.

[25] Radiotherapy for breast cancer [Internet]. Macmillan.org. [cited 2023 May 11]. Available from: https://www.macmillan.org.uk/cancer-information-and-support/treatments-and-drugs/radiotherapy-for-breast-cancer

[26] Radiotherapy for advanced prostate cancer [Internet]. Prostate Cancer UK. [cited 2023 May 11]. Available from: https://prostatecanceruk.org/prostate-information-and-support/treatments/radiotherapy-for-advanced-prostate-cancer

[27] J. Cummings, C. Taylor, R. Chuter1. PO-1058 Estimating the carbon footprint of the radiotherapy pathway and changes in response to COVID-19. Radiotherapy and oncology. 2022. DOI:10.1016/S0167-8140(22)03022-5

For peer review only - http://bmjopen.bmj.com/site/about/guidelines.xhtml

FUNDED BY NIHR | National Institute for Health and Care Research

LIVERPOOL CLINICAL TRIALS CENTRE LCTC

ICR

[28] SHC care pathway calculator guidance, GP consultation module, page 18, 2015. Available from: https://shcoalition.org/sustainable-care-pathways-guidance/

[29] McAlister, S., Barratt, A.L., Bell, K.J. and McGain, F. (2020), The carbon footprint of pathology testing. Med. J. Aust., 212: 377-382. https://doi.org/10.5694/mja2.50583

[30] Health Building Note (HBN) 12, page 32. NHS Estates. 2004 [cited 2023 May 11]. Available from: https://www.england.nhs.uk/wp-content/uploads/2021/05/HBN_12.pdf

[31] Health Technical Memorandum 07-02: EnCO2de 2015 – making energy work in healthcare. Department of Health. 2015 [cited 2023 May 11]. Available from: https://www.england.nhs.uk/wp-content/uploads/2021/05/HTM_07-02_Part_A_FINAL.pdf

[32] Freezer challenge blog. Did you know? [Internet]. Freezerchallenge.org. 2020 June 19 [cited 2023 May 11]. Available from: https://www.freezerchallenge.org/fc-blog/did-you-know

Acknowledgements

This work was produced through funding from the NIHR entitled **NIHR Clinical Trials Unit Support Funding Opportunity – Supporting efficient / innovative delivery of NIHR research**

Authors: Jessica Griffiths, Lisa Fox, Paula Williamson

On behalf of the Low Carbon Clinical Trials working group: Fiona Adshead, Rustam Al-Shahi Salman, Craig Anderson, Emma Bedson, Judith Bliss, Ana Boshoff, Xiaoying Chen, Denise Cranley, Peter Doran, Carrol Gamble, Kerenza Hood, Naomi McGregor, Carolyn McNamara, Elis Midha, Keith Moore, Alexis M Perkins, Sarah Pett, Matthew R Sydes.

Grateful thanks to Environmental Resources Management for their technical support and expertise in the development of the method.

For peer review only

FUNDED BY NIHR | National Institute for Health and Care Research

Enabling lower carbon clinical trials: Development and prototype testing of a method to quantify the carbon footprint of clinical trials to inform future lower carbon clinical trial design

Guidance and method to calculate the carbon footprint of a clinical trial

For peer review only

Data collation quick guide and worksheet

This guidance provides information on how to carbon footprint a clinical trial for the purposes of the NIHR-funded project ‘enabling lower carbon clinical trials.’

Within the guidance, clinical trial processes have been sub-divided into the following modules:

1. Trial set up
2. CTU emissions
3. Trial specific meetings and travel
4. Treatment intervention
5. Data collection and exchange
6. Trial supplies and equipment
7. Trial specific patient assessments
8. Samples
9. Laboratory
10. Trial close out

This list is not exhaustive, and it is expected that further activities and modules may need to be added to account for specialist processes in all clinical trial types.

NB: analysis of data does not need to be calculated separately, it is covered by the emissions attributed to trial staff FTE in “CTU emissions” and calculations included within “Data Collection and exchange”.

For peer review only - http://bmjopen.bmj.com/site/about/guidelines.xhtml

FUNDED BY NIHR | National Institute for Health and Care Research
LIVERPOOL CLINICAL TRIALS CENTRE LCTC
ICR

In addition to this quick guide and worksheet, we have produced a detailed guidance and method document defining the project scope, limitations and assumptions. The detailed guidance contains a more in depth look and explanation of the calculations found in this document, including emission factor and benchmark data sources, and should be referred to when using this worksheet.

Introduction to calculating carbon footprint

A carbon footprint is a measure of greenhouse gases, usually quoted in kg or tonnes of carbon dioxide equivalent (CO_2e). To calculate the carbon footprint of a particular clinical trial process, both 'activity data' and 'emission factors' are required.

An emission factor, also known as a conversion factor, "is a coefficient which allows you to convert activity data into greenhouse gas emissions. It is the average **emission** rate of a given source, relative to units of activity or process/processes."[1]

The activity data is provided by the user and multiplied by the emission factors provided in this guidance document.

Data collation quick guide and worksheet

This data collation quick guide should be used in conjunction with the "Enabling lower carbon clinical trials: Development and prototype testing of a method to quantify the carbon footprint of clinical trials to inform future lower carbon clinical trial design - Detailed Guidance and method to calculate the carbon footprint of a clinical trial". The guidance document provides the detailed explanation of how calculations should be considered and calculated. This quick guide should be used to collate the trial-specific processes, necessary activity data and to record the subsequent calculations. It is important to avoid double-counting activities i.e., modules must not include activities already covered elsewhere in the clinical trial process map. Please complete this worksheet for each trial to be carbon footprinted.

NB: We are using the term 'CTU' to describe the organisation that manages all aspects of central trial management. For some institutions some of those tasks maybe done by groups outside the CTU team e.g., sponsor office/CRO etc.

For peer review only - http://bmjopen.bmj.com/site/about/guidelines.xhtml

FUNDED BY NIHR | National Institute for Health and Care Research

LIVERPOOL CLINICAL TRIALS CENTRE LCTC

ICR

Module	Examples	Trial activity data	Calculation	Total
**1. Trial set-up**				
1.1. Production of trial documentation to be sent to sites or participants	E.g. Site Investigator File and contents, Site Pharmacy File and contents, CRF Folder and contents, PIS/Cs, GP letters	Number of pages:<br><br>Number of folders used to send trial documentation:<br><br>**For eTMF:**<br>GB required for data storage and transmission:<br>Duration of data storage:	**Paper:**<br>[no. of page] x 0.005 = paper weight (kg)<br><br>b/w printing: Kg of paper x 0.22438 = $kgCO_2e$<br>Colour printing: Kg of paper x 0.31786 = $kgCO_2e$<br>Materials (paper): Kg of paper x 0.919 = $kgCO_2e$<br><br>**Folders**:<br>Kg (of cardboard) x 0.821 = $kgCO_2e$<br>Assumption: Weight of lever arch = 0.5kg<br>Assumption: Weight of ring binder = 0.3kg<br><br>**eTMF:**<br>Estimate 1.365 kg CO_2e per GB per year.	

For peer review only

For peer review only - http://bmjopen.bmj.com/site/about/guidelines.xhtml

FUNDED BY NIHR | National Institute for Health and Care Research

LIVERPOOL CLINICAL TRIALS CENTRE LCTC

ICR

1.2. Provision/postage of trial documentation to sites<br><br>1.3. Provision/postage of documentation to participants by CTU or participating sites<br><br>1.4. Provision/postage of incentives to participant	E.g. Site Investigator File and contents, Site Pharmacy File and contents, CRF Folder and contents, PIS/Cs, GP letters	Estimated weight of delivery (road):<br><br>Estimated distance of delivery (road):<br><br>Estimated weight of delivery (air):<br><br>Estimated distance of delivery (air):	Delivery weight (tonnes) x distance (km) = t.km<br><br>For road freight: t.km x 0.19443 = kgCO_2e<br><br>For air freight: t.km x required emission factor below = kgCO_2e<br><br>▪ Domestic (to/from UK) = 4.98549<br>▪ Short-haul (to/from UK) = 2.55439<br>▪ Long-haul (to/from UK) = 1.13047<br>▪ International (to/from non-UK) = 1.13047<br><br>For delivery of trial supplies to patients or GP, if unknown, use 17.4km as distance from hospital to patient, or hospital to GP.	
**2. CTU emissions**				
2.1. Energy consumption at CTU according to trial staff FTE	E.g. energy consumption per square metre of air-conditioned office space	Trial duration:<br>Trial staff FTE:	**Energy consumption for 1 FTE for 1 year** = 193.3 kgCO_2e<br><br>Multiply by the number of years and FTE applicable	

For peer review only

2.2. Heating	E.g. energy consumption at coordination centre attributed to heating (natural gas), homeworking	Trial duration:<br>Trial staff FTE:	**Heating for 1 FTE for 1 year =** 431.56 $kgCO_2e$<br><br>Multiply by the number of years and FTE applicable<br><br>**Homeworking =** 0.34075 $kgCO_2e$ per FTE working hour (includes electricity for office equipment and heating).<br><br>Total FTE working hours x 0.34075 = $kgCO_2e$	
2.3. Trial team commuting	E.g. Car, rail, bus, walking etc	Trial duration:<br>Trial staff FTE:	For 1 FTE for 1 year, total average commuting emissions = 1027.8 $kgCO_2e$<br><br>**Multiply by the number of years and FTE applicable**<br><br>If you can avoid using averages, refer to section 2.3 of the detailed guidance for the emission factors of individual modes of transport.	
**3. Trial specific meetings and travel**				
3.1. Visits and travel to site<br>3.2. Travel to meetings	E.g. Feasibility, site initiation and monitoring visits, audits, inspections, TMG, TSC, IDMC, and	Estimated distance travelled:<br><br>Number of passengers:	Number of passengers x total distance (km) = p.km	

For peer review only - http://bmjopen.bmj.com/site/about/guidelines.xhtml

FUNDED BY NIHR | National Institute for Health and Care Research

	investigator meetings, PPIE, conferences, scientific meetings etc		For national rail: p.km x 0.04441= $kgCO_2e$<br><br>For flights: p.km x relevant emission factor below<br><br>- Domestic (average) to/from UK: 0.27278<br>- Short-haul (average) to/from UK: 0.17034<br>- Long-haul (average) to/from UK: 0.21423<br>- International (average) to/from non-UK: 0.20373<br><br>NB: Distances may be calculated using google maps and calculated from CTU to destination<br><br>**Videoconferencing** = 157 grams CO_2e per hour.	
3.3. Hotel stays	E.g. monitoring visits, audits, inspections etc	Number of rooms:<br>Number of nights:	For UK: number of hotel rooms x number of nights x 13.9 = $kgCO_2e$<br><br>For UK (London) = number of rooms x number of nights x 13.8 = $kgCO_2e$.	

For peer review only

			For other countries use conversion factors from orange 'hotel stay' tab: conversion-factors-2021-full-set-advanced-users.xlsm (live.com)<br><br>Alternatively, you may use cost-based method:<br>£ spent x 0.358 = $kgCO_2e$	
3.4. Sustenance	E.g. meeting lunches, hotel dinners	Number of lunches or dinners:	Meeting lunches or hotel dinners (vegetarian) = 2.6 $kgCO_2e$ per meal per person<br><br>Meeting lunches or hotel dinners (meat) = 5.92 $kgCO_2e$ per meal per person	
**4. Intervention*** **4.1. Physical (IMP)**<br>**4.2. Clinical (non-IMP)**<br>**4.3. Other (not captured above)**<br>**Please fill out the section most relevant to the intervention being investigated.**				
**4.1. Physical**				
4.1.1, 4.1.2. Movement of intervention, or materials	E.g. movement of intervention from manufacturing site to	Estimated weight and distance of delivery (t.km):	Carry out freight calculation as described in section 1.2.	

* As per assumptions detailed in the guidance, manufacture of the intervention is considered out of scope. This section defines all processes relating to providing and delivering the trial intervention that are over and above routine care.

For peer review only - http://bmjopen.bmj.com/site/about/guidelines.xhtml

required to deliver the intervention	distribution site, shipment of IMP to participating sites or direct to participant		For refrigerated freight, increase the total $kgCO_2e$ associated with freight by 15%.<br><br>Frozen freight:<br>Dry ice has a carbon footprint of **1.81kg CO_2e for 1 kg dry ice** produced/used.<br><br>When calculating the overall emissions of frozen freight, as well as the 1.81 $kgCO_2e$ per 1kg attributed to manufacture, include the weight (kg) of dry ice used in the weight of the freight calculation in section 1.2.<br><br>In the absence of activity data, assume 1kg of dry ice is used per individual sample shipping box.	
4.1.3. Materials required for the packaging and shipment of IMP	E.g. cardboard, cold storage boxes, polystyrene	Number of cold storage boxes:<br><br>Kg of cardboard/ polystyrene:	Single use sample cold storage box = 25.2 $kgCO_2e$ per box<br><br>Reusable sample cold storage box = 2.2 $kgCO_2e$ per box<br><br>Kg (cardboard) x 0.821 = $kgCO_2e$	

For peer review only

For peer review only - http://bmjopen.bmj.com/site/about/guidelines.xhtml

FUNDED BY NIHR | National Institute for Health and Care Research

LIVERPOOL CLINICAL TRIALS CENTRE LCTC

ICR

			Kg (polystyrene) x 3.778 = kgCO_2e	
4.1.4. Destruction of overage	E.g. incineration of IMP	Estimated weight of overage incinerated:	Kg of waste x 2.4252 = kgCO_2e	
**4.2. Clinical** e.g., radiotherapy, device, surgical.<br>NB: not all calculations will be relevant to all interventions. **This section of the method will be further developed as we carbon footprint more trials, so please inform us if your protocol specifies an activity that has not been included, and we will help to determine the associated carbon footprint.**				
4.2.1 Movement of the intervention, or resources required to deliver the intervention	E.g. movement of intervention from manufacturing site to distribution site, shipment of intervention to participating sites.	Estimated weight and distance of delivery (t.km):	Please refer to section 1.2 and 4.1.1, 4.1.2.	
4.2.2 Materials required for the packaging and shipment of the intervention	E.g. cardboard, cold storage boxes, polystyrene	Number of cold storage boxes:<br><br>Kg of cardboard/ polystyrene:	Please refer to section 4.1.3.	
4.2.3 Utilities required for delivery of the intervention	E.g. Hospital utilities if the intervention is delivered within a hospital	Hospital staff FTE required:	Please refer to section 7.3 to calculate the emissions attributed to hospital utilities if required to deliver the intervention.	

For peer review only - http://bmjopen.bmj.com/site/about/guidelines.xhtml

FUNDED BY NIHR | National Institute for Health and Care Research
LIVERPOOL CLINICAL TRIALS CENTRE LCTC
ICR

4.2.4 Activities or resources required/relating to delivery of the intervention	E.g. consumables, surgical time, specialist equipment, incineration of surgical waste		Please refer to section 7.2 for consumables, surgery and other activities that may be relevant to the delivery of the intervention, but please take care to avoid double counting.<br><br>To calculate the emissions attributed to incineration, e.g. of surgical waste, please refer to section 4.1.4.	
**4.3. Other**<br>NB: not all calculations will be relevant to all interventions. **This section of the method will be further developed as we carbon footprint more trials, so please inform us if your protocol specifies an activity that has not been included, and we will help to determine the associated carbon footprint.**				
4.3.1 Movement of the intervention to the participant or participating site	E.g. shipment of intervention to participating sites or direct to participant	Estimated weight and distance of delivery (t.km):	Please refer to section 1.2.	
4.3.2 Materials required for packaging and shipment of the intervention	E.g. cardboard, cold storage boxes, polystyrene	Number of cold storage boxes:<br><br>Kg of cardboard/ polystyrene:	Please refer to section 4.1.3.	
4.3.3 Materials or resources required for delivery of the intervention	E.g. software, booklets, specialist equipment		For printing and paper, please refer to section 1.1.	
4.3.4 Travel required to facilitate delivery of the intervention	E.g. to deliver training, conduct interviews etc	Estimated distance travelled, number of passengers (p.km):	Please refer to section 3.1, 3.2.	
**5. Data collection and exchange**				

For peer review only - http://bmjopen.bmj.com/site/about/guidelines.xhtml

NB: analysis of data does not need to be calculated separately, it is covered by the emissions attributed to trial staff FTE in "CTU emissions" and calculations included within "Data Collection and exchange".				
5.1. Data collection and query exchange between CTU and sites	E.g. CRFs, EDC completion and query resolution, scans copied to CDs	Estimated weight and distance of deliveries (t.km):<br><br>Number of CDs and time taken to copy scans:<br><br>Number of emails:<br>NB: this is an estimate of all emails exchanged between CTU and participating sites throughout the study lifetime, including data query resolution emails.<br><br>GB required for data storage and transmission:<br>Duration of data storage:<br><br>NB: Web-based data entry at sites, e.g. CRF completion, will be accounted for in the time a hospital worker spends on the trial and the carbon footprint of the trial databases.	For postage of materials, please refer to section 1.2 (freight).<br><br>The carbon footprint of manufacturing a CD = 0.83 kg CO_2e per CD (833 g CO_2e)<br><br>The carbon footprint of copying the scans on to a CD using a computer = 0.18079 kg CO_2e per hour<br><br>An email without an attachment = 10g CO_2e. Double this for an email with a one-megabyte attachment.<br><br>Data storage and transmission: estimate 1.365 kg CO_2e per GB per year.	
5.2. Data sent direct from participants to CTU or participating sites	E.g. Questionnaires, patient diaries, wearables	Estimated weight, and distance of delivery (t.km):<br><br>Device used and time taken to complete electronic questionnaires:	For paper questionnaires, please refer to section 1.1. for the carbon footprint of producing the materials and section 1.2. for postage (freight).	

For peer review only - http://bmjopen.bmj.com/site/about/guidelines.xhtml

FUNDED BY NIHR | National Institute for Health and Care Research

			For use of smart watches and other devices see section 6.3.<br><br>**Electronic questionnaires**<br>Add the emissions attributed to data storage and transmission to the emissions attributed to using a device to complete the questionnaire.<br><br>Web surfing (data storage and transmission) = 9.441 g CO2e/hr (10 mins = 1.57 g CO_2e)<br><br>Choose from the below:<br>- Desktop computer = 0.18079 kg CO_2e per hour<br>- Laptop = 0.028719 kg CO_2e per hour<br>- Tablet = 0.027397 kg CO_2e per hour<br>- Smartphone = 0.015068 kg CO_2e per hour	

For peer review only

FUNDED BY NIHR | National Institute for Health and Care Research

LIVERPOOL CLINICAL TRIALS CENTRE LCTC

ICR

5.3. Data from labs to CTU<br>5.4. Data from other collaborators to CTU	E.g. Laboratory patient results, data linkage	GB required for data storage and transmission:<br>Duration of data storage:<br><br>Total £ spent on computer services:	For electronic storage**,** estimate 1.365 $kgCO_2e$ per GB per year.<br><br>For computer services such as data linkage:<br>£ x 0.149 = $kgCO_2e$	
**6. Trial supplies and equipment**				
6.1. Equipment used by CTU	E.g. computers, laptops, printers, software	Total £ spent on office machinery and computers for trial:	For any new office machinery and computers purchased specifically for trial:<br>£ x 0.387 = $kgCO_2e$	
6.2. Equipment and supplies used by participating sites supplied by CTU	E.g. centrifuge, fridge, freezer	Estimated weight and distance of delivery(t.km):	For the shipment of equipment to participating sites, please refer to section 1.2.<br><br>For the use of a centrifuge, please refer to section 9.2., for a fridge or freezer please refer to section 9.3.	
6.3. Equipment and supplies provided to participants specifically for the trial	E.g. wearables, smartphone, tablet	Number of devices and duration of their usage:<br><br>Estimated weight and distance of deliveries (t.km):	**Smartphone =** 55 $kgCO_2e$ from manufacture and add 5.5 $kgCO_2e$ per year of usage.<br><br>**Tablet =** 119 $kgCO_2e$ from manufacture and add 10 $kgCO_2e$ per year of usage.	

For peer review only - http://bmjopen.bmj.com/site/about/guidelines.xhtml

FUNDED BY NIHR | National Institute for Health and Care Research

LIVERPOOL CLINICAL TRIALS CENTRE LCTC

ICR

			**Wearables/smart watch** = 30.1 kg CO_2e for manufacture and add 1.633 kg CO_2e per year of usage.<br><br>**To calculate the carbon footprint associated with shipment of the devices, please refer to section 1.2.**	
**7. Trial specific patient assessments**				
7.1. Patient travel for study visits that are in addition to standard of care	E.g. Eligibility and screening assessments, trial-specific assessments and procedures	Number of times patient is required to travel (in addition to standard of care):<br><br>Number of patients:	Emissions associated with one patient visit to hospital (UK) = 5.8 kgCO_2e (this includes both the out and back journeys)<br><br>Emissions associated with one patient visit to GP surgery (UK) = 1.12 kgCO_2e (this includes both the out and back journeys)	
7.2. Materials and activities required for study assessments that are in addition to standard of care	E.g. Laboratory tests, imaging assessments, clinical activities relating to intervention for example administering of study drug, biopsy.	Patient schedule of assessments:	Consumables = 0.30 kgCO_2e per patient per trial appointment where consumables (such as gloves) required<br><br>▪ 1 MRI = 24.7 kg CO_2e<br>▪ 1 CT scan = 9.2 kgCO_2e<br>▪ 1 Chest X-Ray = 0.8 kgCO_2e	

For peer review only

FUNDED BY NIHR | National Institute for Health and Care Research
LIVERPOOL CLINICAL TRIALS CENTRE LCTC
ICR

			1 hour in surgery = 53 kg CO_2e 1 low intensity (general ward) bed day = 37.9 kg CO_2e 1 high intensity (ICU) bed day = 103 kgCO_2e 15 sessions of breast radiotherapy = 5.7 kg CO_2e 20 sessions of prostate radiotherapy = 15.3 kg CO_2e Blood tests: 82 g CO_2e for coagulation profile 116 g CO_2e for full blood examination 49 g CO_2e for arterial gas assessment 99 g CO_2e for urea and electrolyte assessment 0.5 g CO_2e for C-reactive protein **Please note that the above figures for blood tests include the materials and consumables required for sample collection, phlebotomy and analysis, as**	

For peer review only

FUNDED BY NIHR | National Institute for Health and Care Research

			**well as power consumption by pathology analysers.**	
7.3. Utilities required for study assessments that are in addition to standard of care	E.g. energy consumption per square metre of hospital space according to trial staff FTE, taking into account time required for CRF completion and study assessments, consent etc	Trial duration:<br>Trial staff FTE:	1 FTE 1 year = 364.9 $kgCO_2e$<br><br>**Multiply by the number of years and FTE applicable**<br><br>Heating: 1 FTE, 1 year = 685.7 $kgCO_2e$<br>**Multiply by the number of years and FTE applicable**	
**8. Samples**				
8.1. Materials involved	E.g. sample collection kit and packaging for shipment	Kg of material:	The emissions attributed to sample collection consumables for common blood tests are included in the blood tests listed in section 7.2.<br><br>To calculate the carbon footprint of other common materials, multiply the weight in kg by the relevant emission factor below to produce $kgCO_2e$.<br>- Average plastics: 3.116<br>- Plastics (average film): 2.754<br>- Plastics (Average rigid): 3.277	

For peer review only - http://bmjopen.bmj.com/site/about/guidelines.xhtml

FUNDED BY NIHR | National Institute for Health and Care Research

LIVERPOOL CLINICAL TRIALS CENTRE LCTC

ICR

			- Plastics (PP): 3.105<br>- Plastics (PET): 4.032<br>- Glass: 1.403<br>- Paper: 0.919<br>- Board: 0.821<br><br>Example: 100 slide mailing containers made of polypropene = 1.02 kg<br>1.02kg x 3.105 = 3.2 kgCO_2e	
8.2. Movement of sample kit materials from manufacturer to CTU<br>8.3. Movement of sample kits from CTU/distributor to participating Sites<br>8.4. Movement of samples from participating sites or patients to central laboratory.	E.g. shipment of blood tubes for sample kits to CTU	Estimated weight of and distance of delivery (t.km):	Please refer to section 1.2. for freight and 4.1. for refrigerated or frozen freight.	
**9. Laboratory**				
9.1. Emissions attributed to lab utilities according to staff FTE	E.g. energy consumption per square metre of laboratory space according to trial staff FTE	Trial duration:<br>Trial staff FTE:	**Electricity:**<br>1747.2 kgCO_2e per FTE per year<br>Multiply 1747.2 kgCO_2e by the number of years and FTE applicable<br><br>**Heating:**	

For peer review only - http://bmjopen.bmj.com/site/about/guidelines.xhtml

FUNDED BY NIHR | National Institute for Health and Care Research

LIVERPOOL CLINICAL TRIALS CENTRE LCTC

ICR

			1376 $kgCO_2e$ per FTE per year<br>Multiply 1376 $kgCO_2e$ by the number of years and FTE applicable	
9.2. Materials/equipment/consumables used in processing and analysis of samples	E.g. centrifuges, refrigerators	kWh usage of equipment:<br><br>Kg of material:	To avoid double counting, use of equipment will be included in lab staff FTE if calculated.<br><br>If the trial does not involve a central lab, but there is still sample processing on site, please see below. For storage of samples, please see section 9.3.<br><br>To calculate the emissions of a piece of equipment, multiply the power consumption by hours used to get a kWh value. Finally multiply kWh by the electricity emission factor (0.273).<br><br>Example: use of a 310-Watt centrifuge for 15 minutes<br>- 310 Watts x 0.25 (hours) = 77.5 kWh<br>- 77.5 kWh x 0.273 = 21.2 $kgco_2e$	

For peer review only

FUNDED BY NIHR | National Institute for Health and Care Research

LIVERPOOL CLINICAL TRIALS CENTRE LCTC

ICR

			Consider the centrifuge capacity and multiply by the number of uses required.	
9.3. Storage of samples	E.g. utilities and ultra-low temperature freezer	Duration of storage:<br><br>Amount of refrigerator/freezer space required:	Storage in fridge/-20 freezer:<br>- 298.9 $kgCO_2e$ per year<br>- Multiply by number of years stored<br><br>Storage in an ultra-low/-80 freezer:<br>- 2192.2 $kgCO_2e$ per year<br>- Multiply by number of years stored<br><br>NB: this is for a whole freezer; you will need to make an assumption about the amount of space in the freezer that the trial samples take up.	
**10. Trial close out**				
10.1. Storage and archiving of essential trial documentation and data	E.g. Hospital files, lab files, trial guidance documents etc	Duration of storage:<br><br>Amount of space required for storage:	Carbon footprint associated with $1m^2$ for 1 year:<br>- Office: 16.1 $kgC0_2e$<br>- Laboratory: 43.7 $kgc0_2e$<br>- Warehouse: 7.4 $kgC0_2e$<br>- Health building: 22.1 $kgCO_2e$	

For peer review only - http://bmjopen.bmj.com/site/about/guidelines.xhtml

FUNDED BY NIHR | National Institute for Health and Care Research

LIVERPOOL CLINICAL TRIALS CENTRE LCTC

ICR

			Choose the most suitable building type and multiply by number of years and m^2 necessary.<br><br>**Heating**<br>Carbon footprint associated with 1 m^2 for 1 year:<br>- Office: 35.69 $kgCO_2e$<br>- Laboratory: 34.4 $kgCO_2e$<br>- Warehouse: 13.3 $kgCO_2e$<br>- Health building: 41.5 $kgCO_2e$<br><br>Choose the most suitable building type and multiply by number of years and m^2 necessary.<br><br>**Electronic data and information storage:**<br>estimate 1.365 kg CO_2e per GB per year.	
10.2. Storage and destruction of biological samples	E.g. blood, tissue, urine etc	M^2 required for storage:<br><br>Duration of storage:	See section 9.3. for storage of refrigerated or frozen samples.<br><br>See section 10.1 for storage of ambient samples.	

For peer review only

FUNDED BY NIHR | National Institute for Health and Care Research

LIVERPOOL CLINICAL TRIALS CENTRE LCTC

ICR

10.3. Return of equipment and supplies from participating sites to CTU	E.g. wearables, unused or expired equipment and supplies	Estimated weight and distance of delivery (t.km):	See section 1.2. (freight).	

Carbon footprint summary

**Module**	**KgCO_2e**
Trial set up	
CTU emissions	
Trial staff meetings and travel	
Treatment intervention	
Data collection and exchange	
Trial supplies and equipment	
Trial specific patient assessments	
Samples	
Laboratory	
Analysis and trial close out	
**Total =**	

For peer review only - http://bmjopen.bmj.com/site/about/guidelines.xhtml

FUNDED BY NIHR | National Institute for Health and Care Research

References

[1]What is an emission factor? [Internet]. Climfoot-project.eu. [cited 2023 May 11]. Available from: https://climfoot-project.eu/en/what-emission-factor

For all emission factor and benchmark data sources, please refer to the accompanying “Detailed Guidance and method to calculate the carbon footprint of a clinical trial.”

For peer review only

For peer review only - http://bmjopen.bmj.com/site/about/guidelines.xhtml

FUNDED BY NIHR | National Institute for Health and Care Research

Enabling lower carbon clinical trials: Development and prototype testing of a method to quantify the carbon footprint of clinical trials to inform future lower carbon clinical trial design

Guidance and method to calculate the carbon footprint of a clinical trial

For peer review only

Data collation quick guide and worksheet

This guidance provides information on how to carbon footprint a clinical trial for the purposes of the NIHR-funded project 'enabling lower carbon clinical trials.'

Within the guidance, clinical trial processes have been sub-divided into the following modules:

1. Trial set up
2. CTU emissions
3. Trial specific meetings and travel
4. Treatment intervention
5. Data collection and exchange
6. Trial supplies and equipment
7. Trial specific patient assessments
8. Samples
9. Laboratory
10. Trial close out

This list is not exhaustive, and it is expected that further activities and modules may need to be added to account for specialist processes in all clinical trial types.

NB: analysis of data does not need to be calculated separately, it is covered by the emissions attributed to trial staff FTE in "CTU emissions" and calculations included within "Data Collection and exchange".

For peer review only - http://bmjopen.bmj.com/site/about/guidelines.xhtml

FUNDED BY NIHR | National Institute for Health and Care Research

LIVERPOOL CLINICAL TRIALS CENTRE LCTC

ICR

In addition to this quick guide and worksheet, we have produced a detailed guidance and method document defining the project scope, limitations and assumptions. The detailed guidance contains a more in depth look and explanation of the calculations found in this document, including emission factor and benchmark data sources, and should be referred to when using this worksheet.

Introduction to calculating carbon footprint

A carbon footprint is a measure of greenhouse gases, usually quoted in kg or tonnes of carbon dioxide equivalent (CO_2e). To calculate the carbon footprint of a particular clinical trial process, both 'activity data' and 'emission factors' are required.

An emission factor, also known as a conversion factor, "is a coefficient which allows you to convert activity data into greenhouse gas emissions. It is the average **emission** rate of a given source, relative to units of activity or process/processes."[1]

The activity data is provided by the user and multiplied by the emission factors provided in this guidance document.

Data collation quick guide and worksheet

This data collation quick guide should be used in conjunction with the "Enabling lower carbon clinical trials: Development and prototype testing of a method to quantify the carbon footprint of clinical trials to inform future lower carbon clinical trial design - Detailed Guidance and method to calculate the carbon footprint of a clinical trial". The guidance document provides the detailed explanation of how calculations should be considered and calculated. This quick guide should be used to collate the trial-specific processes, necessary activity data and to record the subsequent calculations. It is important to avoid double-counting activities i.e., modules must not include activities already covered elsewhere in the clinical trial process map. Please complete this worksheet for each trial to be carbon footprinted.

NB: We are using the term 'CTU' to describe the organisation that manages all aspects of central trial management. For some institutions some of those tasks maybe done by groups outside the CTU team e.g., sponsor office/CRO etc.

For peer review only - http://bmjopen.bmj.com/site/about/guidelines.xhtml

FUNDED BY NIHR | National Institute for Health and Care Research

LIVERPOOL CLINICAL TRIALS CENTRE LCTC

ICR

CASPS carbon footprint calculations

CASPS is an international phase II trial of CEDIRANIB in the treatment of patients with alveolar soft part sarcoma. The trial enrolled 47 patients across twelve sites in the UK, Spain, and Australia and involved an internationally shipped IMP, completion and shipment of paper CRF, on site visits for site initiation, monitoring visits and audits, additional hospital visits for patients for trial-specific patient assessments and provision of samples.

Module	Examples	Trial activity data	Calculation	Results
**1. Trial set-up**				
1.1. Production of trial documentation to be sent to sites or participants	E.g. Site Investigator File and contents, Site Pharmacy File and contents, CRF Folder and contents, PIS/Cs, GP letters	12 sites, 47 patients<br><br>SIF: 401 pages, 12 sent (4812 pages)<br>Site pharmacy file: 301 pages, 11 sent (3311 pages)<br>CRF folder: 76 pages in set up pack, 17 sent (1292 pages)<br>PIS/C 1 = 17 pages, 27 patients (799 pages)<br>PIS/C 2 = 16 pages, 47 patients (762)<br>Letters inc. GP = 8 pages, 47 patients (376)<br><br>Total = 11352 pages<br><br>Number of folders used to send trial documentation: estimate 23 large ring binders (0.5kg) and 18 small (0.3kg)	**Paper:**<br>[no. of page] x 0.005 = paper weight (kg)<br><br>b/w printing: Kg of paper x 0.22438 = $kgCO_2e$<br>Colour printing: Kg of paper x 0.31786 = $kgCO_2e$<br>Materials (paper): Kg of paper x 0.919 = $kgCO_2e$<br><br>Folders: Kg (of cardboard) x 0.821 = $kgCO_2e$<br>Assumption: Weight of lever arch = 0.5kg<br>Assumption: Weight of ring binder = 0.3kg<br><br>Paper:<br>11352 pages x 0.005 = 56.76kg<br>Printing: 56.76 kg x 0.22438 = 12.7 $kgCO_2e$<br>Materials: 56.76 kg x 0.919 = 52.2 $kgCO_2e$<br><br>Folders:<br>(23 x 0.5) + (18 x 0.3) = 16.9kg<br>16.9kg x 0.821 = 13.9 $kgCO_2e$	78.8 $kgCO_2e$

For peer review only - http://bmjopen.bmj.com/site/about/guidelines.xhtml

FUNDED BY NIHR | National Institute for Health and Care Research

1.2. Provision/postage of trial documentation to sites<br><br>1.3. Provision/postage of documentation to participants by CTU or participating sites<br><br>1.4. Provision/postage of incentives to participant	E.g. Site Investigator File and contents, Site Pharmacy File and contents, CRF Folder and contents, PIS/Cs, GP letters	Weight and distance of deliveries (t.km):<br>UK sites<br>- RMH - 0.00519 tonnes x 24.1km = 0.125079 t.km<br>- Christie - 0.0092 x 355.7km = 3.275997 t.km<br>- Bristol - 0.0063 tonnes x 196.3 km= 1.23669 t.km<br>- UCLH - 0.00519 tonnes x 25.7km = 0.133383 t.km<br>- Newcastle - 0.008725 tonnes x 506.9km = 4.4227 t.km<br>- Nottingham - 0.00519 tonnes x 259.1km = 1.344729 t.km<br>Australia<br>0.00587 tonnes x (17018.5km+16544km) = 197 t.km<br><br>Spain<br>0.00519 x (1147km + 1244km + 1091km) = 18.07 t.km<br><br>GP letter<br>Weight of GP letter:<br>2x5g + envelope (7g) = 17g /0.000017tonnes	Delivery weight (tonnes) x distance (km) = t.km<br><br>For road freight: t.km x 0.19443 = $kgCO_2e$<br><br>For air freight: t.km x required emission factor below = $kgCO_2e$<br>▪ Domestic (to/from UK) = 4.98549<br>▪ Short haul (to/from UK) = 2.55439<br>▪ Long-haul (to/from UK) = 1.13047<br>▪ International (to/from non-UK) = 1.13047<br><br>UK<br>Total t.km = 10.539289 x 0.19443 = 2.05 $kgCO_2e$<br><br>Australia<br>197 t.km x 1.13047 = 222.7 $kgCO_2e$<br><br>Spain<br>18.07 t.km x 2.55439 = 46.2 $kgCO_2e$<br><br>GP letter postage:<br>For delivery of trial supplies to patients or GP, if unknown, use 17.4km as distance from hospital to patient, or hospital to GP.<br><br>0.000799 tonnes x 17.4km = 0.014 t.km<br>0.014 t.km x 0.19443 = 0.002722 $kgCO_2e$	271 $kgCO_2e$

For peer review only

FUNDED BY NIHR | National Institute for Health and Care Research — Liverpool Clinical Trials Centre LCTC — ICR

		0.000017 tonnes x 47 patients = 0.000799 tonnes	6 letters sent to each patient - 0.002722 x 6 = 0.016 $kgCO_2e$[*]	
**2. CTU emissions**				
2.1. Energy consumption at CTU according to trial staff FTE	E.g. energy consumption per square metre of air-conditioned office space	Trial duration: 6 years<br><br>Trial staff FTE: UK - 1, Spain - 0.3, Australia - 0.5	**Energy consumption for 1 FTE for 1 year** = 193.28 $kgCO_2e$<br>Multiply by the number of years and FTE applicable<br><br>UK CTU emissions = 193.28 $kgCO_2e$ x 6 = 1159.7 $kgCO_2e$<br><br>Australia CTU<br>- location: Victoria<br>- Office building energy consumption = 231.39 kwh/m^2 source: baseline-energy-consumption-part_1-report-2012.pdf. energy split is 90% electricity, 10% gas. Therefore electricity = 208.251 kwh/m^2<br>- Energy usage per staff (per 12 m^2) = 12 m^2 x 208.251 kWh =2499.012 kwh<br>- Victoria electricity emission factor = 0.98 kg CO2-e/kWh (Source: EERS release 2020–21 (cleanenergyregulator.gov.au))<br>- 2499.012 kWh x 0.98 = 2449$kgCO_2e$ per FTE per year	8854.6 $kgCO_2e$

[*] Assumption: as the international sites are also located in cities, the same patient to GP distance was used for Australia and Spain

For peer review only - http://bmjopen.bmj.com/site/about/guidelines.xhtml

FUNDED BY NIHR | National Institute for Health and Care Research
LIVERPOOL CLINICAL TRIALS CENTRE LCTC
ICR

			- 2449 $kgCO_2e$ x 0.5 (FTE) x 6 years = 7347$kgCO_2e$<br><br>Spain trial staff<br>- UK benchmark data used as a proxy for average office building energy consumption because like the UK, Spain has a reasonably contribution from renewables.<br>- 193.284 $kgCO_2e$ x 6 x 0.3 = 347.9 $kgCO_2e$	
2.2. Heating	E.g. energy consumption at coordination centre attributed to heating (natural gas)	Trial duration: 6 years<br><br>Trial staff FTE: UK - 1, Spain - 0.3, Australia - 0.5	**Heating for 1 FTE for 1 year =** 431.56 $kgCO_2e$<br>Multiply by the number of years and FTE applicable<br>UK = 431.56 $kgCO_2e$ x 6 = 2589.4$kgCO_2e$<br><br>Australia<br>- In office buildings energy split is 90% electricity, 10% gas. Office gas intensity = 23.139 kwh/m^2<br>- Energy usage per staff (per 12 m^2) = 12m^2 x 23.139 kWh = 277.668 kWh<br>- 277.668 kWh = 1GJ<br>- Australian National Greenhouse Accounts Factors (dcceew.gov.au) Emission factor for natural gas: Scope 1 = 51.3 $kgCO_2e$/GJ, Scope 3 = 4.0 $kgCO_2e$/GJ<br>- (1x 51.53 $kgCO_2e$/GJ) + (1x4.0 $kgCO_2e$/GJ)	3532.8 $kgCO_2e$

For peer review only - http://bmjopen.bmj.com/site/about/guidelines.xhtml

			- = 55.53 $kgCO_2e$<br>- 55.53 $kgCO_2e$ x 0.5 (FTE) x 6 years = Total = 166.59 $kgCO_2e$<br>Spain<br>- UK benchmark and emission factor data used as a proxy for average office building energy consumption.<br>- 431.56 $kgCO_2e$ x 0.3 x 6 = 776.8 $kgCO_2e$	
2.3. Trial team commuting	E.g. Car, rail, bus, walking etc	Trial duration: 6 years<br><br>Trial staff FTE: UK - 1, Spain - 0.3, Australia - 0.5	For 1 FTE for 1 year, total average commuting emissions = 1027.8 $kgCO_2e$[†]<br>Multiply by the number of years and FTE applicable<br><br>UK = 1027.8 x 6 = 6166.8 $kgCO_2e$<br>Australia = 1027.8 $kgCO_2e$ x 0.5 x 6 years = 3083.4 $kgCO_2e$<br>Spain = 1027.8 $kgCO_2e$ x 0.3 x 6 years = 1850 $kgCO_2e$	11100.2 $kgCO_2e$
**3. Trial specific meetings and travel**				
3.1. Visits and travel to site<br>3.2. Travel to meetings	E.g. Feasibility, site initiation and monitoring visits, audits, inspections, Trial Management Group (TMG), Trial Steering Committee (TSC), Independent Data Monitoring	Australia feasibility visit:<br>- Flight from Melbourne to Brisbane<br>- Estimated distance travelled = 1375 km<br>- Number of passengers = 2<br><br>Site Initiation visits:	Number of passengers x total distance (km) = p.km<br><br>For national rail: p.km x 0.04441= $kgCO_2e$<br><br>For flights: p.km x relevant emission factor below:<br>- Domestic (average): 0.27278	19433 $kgCO_2e$

[†] Assumption: the average commuting statistics for the UK were found to be similar to those for Spain and Australia and therefore also applied to the international CTUs.

For peer review only - http://bmjopen.bmj.com/site/about/guidelines.xhtml

FUNDED BY NIHR | National Institute for Health and Care Research

LIVERPOOL CLINICAL TRIALS CENTRE LCTC

ICR

	Committee (IDMC), and investigator meetings, Patient and Public Involvement and Engagement (PPIE), conferences, scientific meetings etc	Assumption: Rail used for all travel<br>UK site visits<br>UK total p.km = 3868.6 p.km<br>Australia total p.km = 9736 p.km<br>Spain: 6 teleconferences, 34 total attendees<br><br><u>Monitoring visits:</u><br>UK total p.km = 12957.4 p.km<br>Australia total p.km = 15310 p.km<br>Spain total p.km = 3280 p.km<br><br><u>Audits</u><br>Long haul to/from UK flights = 29442 p.km<br>Short haul = 13464 p.km<br>International = 11444 p.km<br><br><u>Other</u><br>- In person visit to Spanish CTU, 2 ICR-CTSU travelled to Barcelona: 2x2x(1309km) = 5236 p.km<br>- RMH lab visit, 115.8 p.km<br><br><u>Meetings</u><br>Joint IDMC/TSC: all teleconferences, 5 meetings, 3 external attendees<br>TMG: 5 meetings, all teleconferences, 16 attendees	- Short-haul (average) to/from UK: 0.17034<br>- Long-haul (average) to/from UK: 0.21423<br>- International (average) to/from non-UK: 0.20373<br><br>NB: Distances may be calculated using google maps and calculated from CTU to destination<br><br>**Videoconferencing** = 157 grams CO_2e per hour.<br><br><u>Feasibility visits</u><br>Australia:<br>- 2 x (2 x 1375) = 5500 p.km<br>- 5500 p.km x 0.20373 = 1120.5 $kgCO_2e$<br><br><u>Site initiation and pharmacy visits</u><br>UK: 3868.6 p.km x 0.0441 = 171.8 $kgCO_2e$<br>1 teleconference , 4 attendees – 0.157 $kgCO_2e$/hr x 4 = 0.628 $kgCO_2e$<br>Australia: 9736 p.km x 0.20373 = 1983.5 $kgCO_2e$<br>Spain (all teleconference) = 34 x 0.157 $kgCO_2e$/hr = 5.3 $kgCO_2e$<br><br><u>Monitoring visits</u><br>UK: 12957.4 p.km x 0.04441 = 575.4 $kgCO_2e$<br>Australia: 11074 p.km x 0.20373 = 3119.1 $kgCO_2e$<br>Spain: 3280 p.km x 0.17034 = 558.7 $kgCO_2e$	

For peer review only - http://bmjopen.bmj.com/site/about/guidelines.xhtml

FUNDED BY NIHR | National Institute for Health and Care Research

		Conferences:<br>- 2 attendees to a conference in Bristol, p.km = 785.2<br>- 1 attendee to conference in Nottingham, p.km = 518.2	Audits<br>Long haul to/from UK flights = 29422 p.km x 0.21423 = 6303.1 $kgCO_2e$<br>Short-haul = 13464 p.km x 0.17034 = 2293.5 $kgCO_2e$<br>International = 11444 p.km x 0.20373 = 2331.5 $kgCO_2e$<br><br>Other<br>- ICR-CTSU trial staff visit to Barcelona: 5236 p.km x 0.17034 = 891.9 $kgCO_2e$<br>- Lab visit: 115.8p.km x 0.04441 = 5.14 $kgCO_2e$<br><br>Meetings<br>IDMC/TSC: 5x3x 0.157 $kgCO_2e$/hr = 2.4 $kgCO_2e$<br>TMG: 5 x 18 x 0.157 $kgCO_2e$/hr = 12.6 $kgCO_2e$<br><br>Conferences<br>- Bristol: 785.2 p.km x0.04441 = 34.9 $kgCO_2e$<br>- Nottingham: 518.2 p.km = 23 $kgCO_2e$	
3.3. Hotel stays	E.g. monitoring visits, audits, inspections etc	UK hotel stays: 30 stays<br>Assumption: Hotel stays for all visits to Christie, Nottingham and Newcastle (same day visits to RMH, Bristol and UCLH)<br><br>Visit to Spain CTU: 1 room, 7 nights<br><br>Australia: 1 room, 3 nights	For UK: number of hotel rooms x number of nights x 13.9 = $kgCO_2e$<br><br>For UK (London) = number of rooms x number of nights x 13.8 = $kgCO_2e$.<br><br>For other countries use conversion factors from orange 'hotel stay' tab: conversion-factors-2021-full-set-advanced-users.xlsm (live.com)	675.7 $kgCO_2e$

For peer review only - http://bmjopen.bmj.com/site/about/guidelines.xhtml

FUNDED BY NIHR | National Institute for Health and Care Research

LIVERPOOL CLINICAL TRIALS CENTRE LCTC

ICR

			Alternatively, you may use cost-based method:<br>£ spent x 0.358 = $kgCO_2e$<br><br>Trial calculations:<br>UK hotel stays for monitoring visits:<br>13.9 x 30 = 417 $kgCO_2e$<br><br>Visits to Spain CTU:<br>7 x 18.7 = 130.9 $kgCO_2e$<br><br>Australia hotel stays:<br>42.6 x 3 = 127.8 $kgCO_2e$	
3.4. Sustenance	E.g. meeting lunches, hotel dinners	Number of lunches or dinners:<br><br>Or<br><br>£ spent: £503.65	Meeting lunches or hotel dinners (vegetarian) = 2.6 $kgCO_2e$ per meal per person<br><br>Meeting lunches or hotel dinners (meat) = 5.92 $kgCO_2e$ per meal per person<br><br>Or cost-based approach = £ x 0.708 = $kgCO_2e$<br>£503.65 x 0.708 = 356.6 $kgCO_2e$	356.6 $kgCO_2e$
**4. Intervention‡**<br>**4.1. Physical (IMP)**<br>**4.2. Clinical (non-IMP)**<br>**4.3. Other (not captured above)**<br>**Please fill out the section most relevant to the intervention being investigated.**				

‡As per assumptions detailed in the guidance, manufacture of the intervention is considered out of scope. This section defines all processes relating to providing and delivering the trial intervention that are over and above routine care.

For peer review only - http://bmjopen.bmj.com/site/about/guidelines.xhtml

FUNDED BY NIHR | National Institute for Health and Care Research

4.1. Physical				
4.1.1, 4.1.2. Movement of intervention, or materials required to deliver the intervention	E.g. movement of intervention from manufacturing site to distribution site, shipment of IMP to participating sites or direct to participant	NB: all shipping of IMP ambient<br><br>Movement of IMP from manufacturing site to QP site for QP declaration:<br>Assumption: 1 shipment<br>Assumption: 1 pill bottle contains 35 tablets - weight ~30g<br>Assumption: ~4000 IMP bottles shipped<br>Flight distance from Puerto Rico to QP site = 6697 km<br>Shipment weight = 4000x 30g = 120000g = 0.118 tonnes<br><br>Movement of IMP from QP site to distribution/packaging site:<br>Distance from QP site to distribution/packaging site = 352.4 km<br>Shipment weight = 0.118 tonnes<br>Movement of IMP from distribution/packaging site to participating sites:<br>UK deliveries: 7.7 t.km<br>Spain deliveries: 13 t.km<br>Australia deliveries: 184.6 t.km	Carry out freight calculation as described in section 1.2.<br><br>Movement of IMP from manufacturing site to Macclesfield for QP declaration:<br>▪ Long-haul (to/from UK) = 1.13047<br>▪ 0.118 tonnes x 6697km = 790.246 t.km<br>▪ 790.246 t.km x 1.13047 = 893.4 $kgCO_2e$<br><br>Movement of IMP from manufacturing site to distribution/packaging site (Fisher Clinical services):<br>▪ 0.118 tonnes x 352.4km = 41.5832 t.km<br>▪ 41.5832 t.km x 0.19443 = 8.1 $kgCO_2e$<br><br>Movement of IMP from distribution/packaging site to participating sites:<br>UK deliveries: 7.7 t.km x 0.19443 = 1.5 $kgCO_2e$<br>Spain deliveries:<br>▪ short - haul (to/from UK) = 2.55439<br>▪ 13 t.km x 2.55439 = 33.3 $kgCO_2e$<br>Australia deliveries:<br>▪ Long-haul (to/from UK) = 1.13047<br>▪ 184.6 t.km x 1.13047 = 208.7 $kgCO_2e$	1145 $kgCO_2e$

For peer review only - http://bmjopen.bmj.com/site/about/guidelines.xhtml

FUNDED BY NIHR | National Institute for Health and Care Research

LIVERPOOL CLINICAL TRIALS CENTRE LCTC

ICR

4.1.3. Materials required for the packaging and shipment of IMP	E.g. cardboard, cold storage boxes, polystyrene	Kg of cardboard/ polystyrene:	E.g. Single use sample cold storage box = 25.2 $kgCO_2e$ per box<br><br>E.g. Reusable sample cold storage box = 2.2 $kgCO_2e$ per box<br><br>E.g. Kg (cardboard) x 0.821 = $kgCO_2e$<br>E.g. Kg (polystyrene) x 3.778 = $kgCO_2e$	Data for IMP shipment packaging unobtainable from trial records. Item not included for CASPS.
4.1.4. Destruction of overage	E.g. incineration of IMP	Estimated weight of overage incinerated:<br>- Total bottles destroyed = 1464<br>- 1464 x 30g = 43.92 kg	Kg of waste x 2.4252 = $kgCO_2e$<br>43.92 kg x 2.4252 =106.5 $kgCO_2e$	106.5 $kgCO_2e$
**5. Data collection and exchange**<br>NB: analysis of data does not need to be calculated separately, it is covered by the emissions attributed to trial staff FTE in "CTU emissions" and calculations included within "Data Collection and exchange".				
5.1. Data collection and query exchange between CTU and sites	E.g. CRFs, EDC completion and query resolution, scans copied to CDs	CASPS hospital files<br>35 x large folders (500 pages)<br>- 500 x 35 = 17500<br>104 x small (150 pages)<br>- 104 x 150 = 15600<br>Total pages = 17500 + 15600 = 33100 pages<br><br>Scans<br>3 scans copied onto a CD per patient, 47 patients total<br>5 mins laptop time to burn CD per patient	CASPS hospital files<br>33100 x 0.005kg = 165.5 kg<br>Paper (printing) = 165.5kg x 0.22438= 37 $kgCO_2e$<br>Paper (materials) = 165.5kg x 0.919 = 152.1 $kgCO_2e$<br><br>Scans<br>The carbon footprint of manufacturing a CD = 0.83 $kgCO_2e$ per CD<br>0.83 $kgCO_2e$ x 47 = 39.01 $kgCO_2e$	558.3 $kgCO_2e$

For peer review only - http://bmjopen.bmj.com/site/about/guidelines.xhtml

		Postage of scans:<br>UK: 0.278671 t.km<br>Australia: 3.25 t.km<br>Spain: 0.28 t.km<br><br><u>CRFs</u><br>Postage of CRF booklets to sites:<br>UK sites, total t.km = 4.41 t.km<br>Australia sites, total t.km = 102.2 t.km<br>Spain sites, total t.km= 8.58963 t.km<br><br>Postage of CRFs from sites to CTU:<br>UK sites, total t.km = 6.37<br>Australia sites, total t.km = 132.9<br>Spain sites, total t.km = 13.8	The carbon footprint of copying the scans on to a CD using a computer = 0.18079 kgCO$_2$e per hour<br>5mins x 47 = 235 minutes = 3.91666667 hours<br>0.18079 kgCO$_2$e x 3.91666667 hours = 0.71 kgCO$_2$e<br><br>Postage of scans:<br>UK: 0.26115 t.km x 0.19443 = 0.05 kgCO$_2$e<br>Australia: 3.25 t.km x 1.13047 = 3.7 kgCO$_2$e<br>Spain: 0.28 t.km x 2.55439 = 0.72 kgCO$_2$e<br><br><u>CRFs</u><br>Postage of CRF booklets to sites:<br>UK: 4.41 t.km x 0.19443 = 0.86 kgCO$_2$e<br>Australia: 102.2 t.km x 1.13047 = 115.5 kgCO$_2$e<br>Spain: 8.58963 t.km x 2.55439 = 21.9 kgCO$_2$e<br><br>Postage of CRFs from sites to CTU:<br>UK: 6.37 t.km x 0.19443 = 1.2 kgCO$_2$e<br>Australia: 132.9 t.km x 1.13047 = 150.2 kgCO$_2$e<br>Spain: 13.8 t.km x 2.55439 = 35.3 kgCO$_2$e	
5.2. Data sent direct from participants to CTU or participating sites	E.g. Questionnaires, patient diaries, wearables	Estimated weight and distance of delivery (t.km):<br><br>Device used and time taken to complete electronic questionnaires:	For paper questionnaires, please refer to section 1.1. for the carbon footprint of producing the materials and section 1.2. for postage (freight).<br><br>For use of smart watches and other devices see section 6.3.	N/A

For peer review only

FUNDED BY NIHR | National Institute for Health and Care Research — LIVERPOOL CLINICAL TRIALS CENTRE LCTC — ICR

			**Electronic questionnaires**<br>Add the emissions attributed to data storage and transmission to the emissions attributed to using a device to complete the questionnaire.<br><br>Web surfing (data storage and transmission) = 9.441 g CO2e/hr (10 mins = 1.57 g CO_2e)<br><br>Choose from the below:<br>- Desktop computer = 0.18079 kg CO_2e per hour<br>- Laptop = 0.028719 kg CO_2e per hour<br>- Tablet = 0.027397 kg CO_2e per hour<br>- Smartphone = 0.015068 kg CO_2e per hour	
5.3. Data from labs to CTU<br>5.4. Data from other collaborators to CTU	E.g. Laboratory patient results, data linkage	GB required for data storage/transmission:<br>Duration of data storage:<br><br>Total £ spent on computer services:	Data storage and transmission = estimate 1.365 kg CO_2e per GB per year.<br><br>For computer services such as data linkage:<br>£ x 0.149 = kgCO_2e	N/A
**6. Trial supplies and equipment**				
6.1. Equipment used by CTU	E.g. computers, laptops, printers, software	Total £ spent on office machinery and computers for trial: £1350	For any new office machinery and computers purchased specifically for trial:<br>£ x 0.387 = kgCO_2e<br><br>£1350 x 0.387 = 522.45 kgCO_2e	522.45 kgCO_2e
6.2. Equipment and supplies used by	E.g. centrifuge, fridge, freezer	Estimated weight and distance of deliveries (t.km):	For the shipment of equipment to participating sites, please refer to section 1.2.	N/A

For peer review only - http://bmjopen.bmj.com/site/about/guidelines.xhtml

participating sites supplied by CTU			For the use of a centrifuge, please refer to section 9.2., for a fridge or freezer please refer to section 9.3.	
6.3. Equipment and supplies provided to participants specifically for the trial	E.g. wearables, smartphone, tablet	Number of devices and duration of their usage:<br>Estimated weight and distance of deliveries (t.km):	**Smartphone =** 55 kgCO_2e from manufacture and add 5.5 kgCO_2e per year of usage.<br>**Tablet =** 119 kgCO_2e from manufacture and add 10 kgCO_2e per year of usage.<br><br>**Wearables/smart watch** = 30.1 kg CO_2e for manufacture and add 1.633 kg CO_2e per year of usage.<br><br>**To calculate the carbon footprint associated with shipment of the devices, please refer to section 1.2.**	N/A
**7. Trial specific patient assessments**				
7.1. Patient travel for study visits that are in addition to standard of care	E.g. Eligibility and screening assessments, trial-specific assessments and procedures	Number of patients: 47<br>On average patients were on trial 72 weeks.<br><br>Number of times patient were required to travel (in addition to standard of care): 880 total patient visits (496 total visits by patients receiving IMP, 384 total visits by patients receiving placebo)	Emissions associated with one patient visit to hospital (UK) = 5.8 kgCO_2e (this includes both the out and back journeys)<br><br>Emissions associated with one patient visit to GP surgery (UK) = 1.12 kgCO_2e (this includes both the out and back journeys)<br><br>5.8 kgCO_2e x 880 = 5104 kgCO_2e	5104 kgCO_2e

For peer review only - http://bmjopen.bmj.com/site/about/guidelines.xhtml

FUNDED BY NIHR | National Institute for Health and Care Research

LIVERPOOL CLINICAL TRIALS CENTRE LCTC

ICR

7.2. Materials and activities required for study assessments that are in addition to standard of care	E.g. Laboratory tests, imaging assessments, clinical activities relating to intervention for example administering of study drug, biopsy.	On average patients were on trial 72 weeks. Within that time:<br>- CT scans per patient<br>- 754 total visits requiring consumables	Consumables = 0.30 kgCO_2e per patient per trial appointment where consumables (such as gloves) required<br><br>▪ 1 MRI = 24.7 kg CO_2e<br>▪ 1 CT scan = 9.2 kgCO_2e<br>▪ 1 hour in surgery = 53 kg CO_2e<br>▪ 1 low intensity (general ward) bed day = 37.9 kg CO_2e<br>▪ 1 high intensity (ICU) bed day = 103 kgCO_2e<br>▪ 15 sessions of breast radiotherapy = 5.7 kg CO_2e<br>▪ 20 sessions of prostate radiotherapy = 15.3 kg CO_2e<br><br>Blood tests:<br>▪ 82 g CO_2e for coagulation profile<br>▪ 116 g CO_2e for full blood examination<br>▪ 49 g CO_2e for arterial gas assessment<br>▪ 99 g CO_2e for urea and electrolyte assessment<br>▪ 0.5 g CO_2e for C-reactive protein<br><br>**Please note that the above figures for blood tests include the materials and consumables required for sample collection, phlebotomy and analysis, as well as power consumption by pathology analysers.**<br><br>**Trial calculations:**	4117.8 kgCO_2e

For peer review only

For peer review only - http://bmjopen.bmj.com/site/about/guidelines.xhtml

FUNDED BY NIHR | National Institute for Health and Care Research

LIVERPOOL CLINICAL TRIALS CENTRE LCTC

ICR

			CT scans: 47 x 9 x 9.2 $kgCO_2e$ = 3891.6 $kgCO_2e$<br>Consumables: 0.30 $kgCO_2e$ x 754 = 226.2 $kgCO_2e$	
7.3. Utilities required for study assessments that are in addition to standard of care	E.g. energy consumption per square metre of hospital space according to trial staff FTE, taking into account time required for CRF completion and study assessments, consent etc	Trial staff FTE (FTEs added up over whole trial duration):<br>UK – 0.85<br>Spain – 0.25<br>Australia – 0.23<br>Australia benchmark and emission factor data:<br>- Australian average hospital energy intensity = 1676 MJ = 465.55 kWh/m2.<br>- Energy intensity per employee 16.5m² x 465.55 = 7681.6 kWh<br>-50:50 Electricity/gas split, therefore 3840.8 kWh electricity, 3840.8 kWh natural gas<br>- Australia hospital sites located in NSW and QLD where electricity emission factor is 0.81 $kgCO_2e$/kWh.<br>- Australian National Greenhouse Accounts Factors (dcceew.gov.au)<br>Emission factor for natural gas: Scope 1 = 51.3 $kgCO_2e$/GJ, scope 3 = 13.1 $kgCO_2e$/GJ	1 FTE 1 year = 364.9 $kgCO_2e$<br>Multiply by the number of years and FTE applicable<br><br>UK: 364.9 $kgCO_2e$ x 0.85 = 310.2 $kgCO_2e$<br>Spain: 364.9 $kgCO_2e$ x 0.25 = 91.2 $kgCO_2e$<br>Australia: 3840.8 kWh x 0.81 x 0.23 (FTE) = 715.5 $kgCO_2e$<br><br>Heating: 1 FTE, 1 year = 685.7 $kgCO_2e$<br>Multiply by the number of years and FTE applicable<br>UK: 685.7 $kgCO_2e$ x 0.85 = 582.8 $kgCO_2e$<br>Spain: 685.7 $kgCO_2e$ x 0.25 = 171.4$kgCO_2e$<br>Australia: 3840.8 kWh = 13.82688 GJ<br>- (13.82688 GJ x 51.53 $kgCO_2e$/GJ) + (13.82688 GJ x 13.1 $kgCO_2e$/GJ)<br>- = 893.6 $kgCO_2e$<br>- x 0.23 (FTE) = 205.5 $kgCO_2e$	2076.6 $kgCO_2e$
**8. Samples**				

For peer review only - http://bmjopen.bmj.com/site/about/guidelines.xhtml

FUNDED BY NIHR | National Institute for Health and Care Research

LIVERPOOL CLINICAL TRIALS CENTRE LCTC

ICR

8.1. Materials involved	E.g. sample collection kit and packaging for shipment	Kg of material:<br>- Safety kit (average plastic): 3kg<br>- Cryotubes (PP): 6.88kg<br>- Falcon tubes (PP): 6.88kg<br>- PAXgene tube (PET): 6kg<br>- Sea airbags (PET): 1.31kg<br>- Labels (PET): 1.6kg<br>- CTAD tubes (glass): 3.6335 kg<br>- CPT (glass): 3.2kg<br>- Jiffy bags (paper): 6kg<br>- Paper: 1.53kg<br>- Dry ice: 85kg<br>- Sample cold storage box: 29 single use boxes	The emissions attributed to sample collection consumables for common blood tests are included in the blood tests listed in section 7.2.<br><br>To calculate the carbon footprint of other common materials, multiply the weight in kg by the relevant emission factor below to produce $kgCO_2e$.<br>- Average plastics: 3.116<br>- Plastics (average film): 2.754<br>- Plastics (Average rigid): 3.277<br>- Plastics (PP): 3.105<br>- Plastics (PET): 4.032<br>- Glass: 1.403<br>- Paper: 0.919<br>- Board: 0.821<br>- Dry ice: **1.81kg CO_2e for 1 kg dry ice** produced/used<br>- Sample cold storage box (SU): 25.2 $kgCO_2e$ per cold storage box<br><br>Safety kit: 3kg x 3.116 = 9.35 $kgCO_2e$<br>Cryotubes + falcon tubes: 13.76kg x 3.105 = 42.7 $kgCO_2e$<br>PAXgene, sea airbags and labels: 8.9 1kg x 4.032 = 35.9 $kgCO_2e$<br>CTAD+CPT: 6.8225 kg x 3.413 = 9.57 $kgCO_2e$<br>Jiffy bags: 6kg x 0.881 (paper and board mixed) = 5.29 $kgCO_2e$<br>Paper: 1.53kg x 0.919 = 1.4 $kgCO_2e$	989.2 $kgCO_2e$

For peer review only

FUNDED BY NIHR | National Institute for Health and Care Research

			Paper (printing): 1.53kg x 0.22438 = 0.34 $kgCO_2e$<br>Dry ice: 85kg x 1.81 = 153.85 $kgCO_2e$<br>Sample boxes: 29 boxes x 25.2 $kgCO_2e$ = 730.8 $kgCO_2e$	
8.2. Movement of sample kit materials from manufacturer to CTU<br>8.3. Movement of sample kits from CTU/distributor to participating Sites<br>8.4. Movement of samples from participating sites or patients to central laboratory.	E.g. shipment of blood tubes for sample kits to CTU	8.2. Movement of sample kit materials from manufacturer to CTU<br>- T.km = 11.9<br>8.3. Movement of sample kits from CTU/distributor to participating Sites:<br>▪ UK sites, total t.km = 11.9 t.km<br>▪ Australia, total t.km = 19.8528 t.km<br>▪ Spain, total t.km = 10.2 t.km<br>8.4. Movement of samples from participating sites to laboratory.<br>▪ UK sites, total t.km = 32.7 t.km<br>▪ Australia, total t.km = 663.65 t.km<br>▪ Spain, total t.km = 51.486 t.km<br>Movement of samples between laboratories:<br>- 0.8676 t.km	Please refer to section 1.2. for freight and 4.1. for refrigerated or frozen freight.<br><br>Movement of sample kit materials from manufacturer to CTU:<br>- 11.9 t.km X 0.19443 = 2.31 $kgCO_2e$<br><br>Movement of sample kits from CTU/distributor to participating Sites:<br>▪ UK sites:11.9 t.km x 0.19443 = 2.3 $kgCO_2e$<br>▪ Australia: 19.8528 t.km x 1.13047 = 22.4 $kgCO_2e$<br>▪ Spain: 10.2 t.km x 2.55439 = 26 $kgCO_2e$<br><br>8.4. Movement of samples from participating sites to laboratory.<br>- UK sites: 32.7 t.km x 0.19443 = 6.4 $kgCO_2e$<br>- Australia: 663.65 t.km x 1.13047 = 750.2 $kgCO_2e$<br>- Spain: 51.486 t.km x 2.55439 = 131.5 $kgCO_2e$<br><br>Movement of samples between laboratories:	941.3 $kgCO_2e$

For peer review only

For peer review only - http://bmjopen.bmj.com/site/about/guidelines.xhtml

FUNDED BY NIHR | National Institute for Health and Care Research

			- 0.8676 t.km x 0.19443 = 0.17 $kgCO_2e$	
**9. Laboratory**				
9.1. Emissions attributed to lab utilities according to staff FTE	E.g. energy consumption per square metre of laboratory space according to trial staff FTE	Trial duration:<br><br>Trial staff FTE:	**Electricity:**<br>1747.2 $kgCO_2e$ per FTE per year<br>Multiply 1747.2 $kgCO_2e$ by the number of years and FTE applicable<br><br>**Heating:**<br>1376 $kgCO_2e$ per FTE per year<br>Multiply 1376 $kgCO_2e$ by the number of years and FTE applicable	N/A - No central labs within the scope of this trial. This module refers to the site laboratories where initial processing of samples and sample storage occurs.
9.2. Materials/equipment /consumables used in processing and analysis of samples	E.g. centrifuges, refrigerators	Centrifuges required for processing the following samples:<br><br>CTAD samples<br>- Samples centrifuged for 15 mins (0.25 hours)<br>- 310 watts<br>- 310 x 0.25 = 77.5 kWh<br>- 313 samples<br><br>CPT samples<br>- Samples centrifuged for 45 mins (0.583 hours)<br>- 310 Watts	To avoid double counting, use of equipment will be included in lab staff FTE if calculated.<br><br>If the trial does not involve a central lab, but there is still sample processing on site, please see below. For storage of samples, please see section 9.3.<br><br>To calculate the emissions of a piece of equipment, multiply the power consumption by hours used to get a kWh value. Finally multiply kWh by the electricity emission factor (0.273).	812.5 $kgCO_2e$

For peer review only - http://bmjopen.bmj.com/site/about/guidelines.xhtml

FUNDED BY NIHR | National Institute for Health and Care Research

LIVERPOOL CLINICAL TRIALS CENTRE LCTC

ICR

		- 310 x 0.583 = 180.73 kWh<br>- 261 samples	Consider the centrifuge capacity and multiply by the number of uses required.<br><br>Centrifuge – CTAD Samples:<br>- 77.5 kWh x 0.273 = 21.2 $kgCO_2e$ / 24 = 0.881$kgCO_2e$ per sample (Assumption: centrifuge capacity is 24 samples)<br>- 0.881 x 313 = 275.9 $kgCO_2e$<br><br>Centrifuge – CPT samples:<br>- 180.73 kWh x 0.273 = 49.33929 $kgCO_2e$ / 24 = 2.1 $kgCO_2e$ per sample (Assumption: centrifuge capacity is 24 samples)<br>- 2.1 x 261 samples = 536.6 $kgCO_2e$<br><br>Total = 275.9 kgCO2e + 536.6 $kgCO_2e$ = 812.5 $kgCO_2e$	
9.3. Storage of samples	E.g. utilities and ultra-low temperature freezer	Storage of blood samples in fridge/freezer:<br>Length of storage = 10 years<br>Amount of refrigerator/freezer space required = 1/5 of a fridge, 1/3 of a freezer<br><br>Storage of ambient tissue:<br>- 10 years, 2011-2021<br>- Assumption: tissue samples occupy $1m^2$	Storage in fridge/-20 freezer:<br>- 298.9 $kgCO_2e$ per year<br>- Multiply by number of years stored<br>Storage in an ultra-low/-80 freezer:<br>- 2192.2 $kgCO_2e$ per year<br>- Multiply by number of years stored<br><br>NB: this is for a whole freezer; you will need to make an assumption about the amount of space in the freezer that the trial samples take up.<br><br>Fridge/-20:<br>298.9 $kgCO_2e$ x 10 years = 2989 $kgCO_2e$	8541.1 $kgCO_2e$

For peer review only

			2989 kgCO_2e x 0.20 = 597.8 kgCO_2e<br><br>Storage in an ultra-low/-80 freezer:<br>2192.2 kgCO_2e per year x 10 = 21922 kgCO_2e<br>21922 kgCO_2e x 0.333 = 7307.3 kgCO_2e<br><br>Ambient storage of tissue:<br>Electricity:<br>- Health building = 22.1 kgCO_2e/ m^2<br>- 22.1 kgCO_2e/ m^2 x 10 = 221 kgCO_2e<br>Heating:<br>41.5 kgCO_2e x 10 = 415 kgCO_2e	
**10. Trial close out**				
10.1. Storage and archiving of essential trial documentation and data	E.g. Hospital files, lab files, trial guidance documents etc	Duration of storage: 10 years<br><br>Amount of space required for storage (m^2): Assumption: archiving documents requires 4m^2<br><br>Storage location: CTU (office)	Carbon footprint associated with 1m^2 for 1 year:<br>- Office: 16.1 kgCO_2e<br>- Laboratory: 43.7 kgCO_2e<br>- Warehouse: 7.4 kgCO_2e<br>- Health building: 22.1 kgCO_2e<br><br>Choose the most suitable building type and multiply by number of years and m^2 necessary.<br><br>16.1 kgCO_2e x 4 x 10 = 644 kgCO_2e<br><br>**Heating**<br>Carbon footprint associated with 1 m^2 for 1 year:<br><br>- Office: 35.69 kgCO_2e	2085.3 kgCO_2e

For peer review only

For peer review only - http://bmjopen.bmj.com/site/about/guidelines.xhtml

FUNDED BY NIHR | National Institute for Health and Care Research | LIVERPOOL CLINICAL TRIALS CENTRE LCTC | ICR

			- Laboratory: 34.4 $kgCO_2e$<br>- Warehouse: 13.3 $kgCO_2e$<br>- Health building: 41.5 $kgCO_2e$<br><br>Choose the most suitable building type and multiply by number of years and m^2 necessary.<br><br>35.69 $kgCO_2e$ x 4 x 10 = 1427.6 $kgCO_2e$<br><br>**For electronic storage,** estimate 1.35 $kgCO_2e$ per GB per year.<br><br>Size of CASPS on server = ~1GB<br>1.365 $kgCO_2e$ x 10 = 13.65 $kgCO_2e$	
10.2. Storage and destruction of biological samples	E.g. blood, tissue, urine etc	Duration of storage: 10 years<br><br>Amount of space required for storage (m^2): 1<br><br>Storage location: hospital/health building	See section 9.3. for storage of refrigerated or frozen samples.<br><br>See section 10.1 for storage of ambient samples.<br><br>Health building<br>Electricity: 22.1 $kgCO_2e$ x 10 = 221 $kgCO_2e$<br>Heating: 41.5 $kgCO_2e$ x 10 = 415 $kgCO_2e$	636 $kgCO_2e$
10.3. Return of equipment and supplies from participating sites to CTU	E.g. wearables, unused or expired equipment and supplies	Estimated weight and distance of delivery (t.km):	See section 1.2. (freight).	N/A

For peer review only - http://bmjopen.bmj.com/site/about/guidelines.xhtml

FUNDED BY NIHR | National Institute for Health and Care Research

LIVERPOOL CLINICAL TRIALS CENTRE LCTC

ICR

For peer review only

CASPS carbon footprint summary

**Module**	**$KgCO_2e$**
Trial set up	349.8
CTU emissions	23487.6
Trial staff meetings and travel	20465.3
Treatment intervention	1251.5
Data collection and exchange	558.3
Trial supplies and equipment	522.5
Trial specific patient assessments	11298.4
Samples	1930.5
Laboratory	9353.6
Analysis and trial close out	2721.3
**Total =**	71938.8 $kgCO_2e$ / 71.9 $tonnesCO_2e$

For peer review only - http://bmjopen.bmj.com/site/about/guidelines.xhtml

FUNDED BY NIHR | National Institute for Health and Care Research

References

[1] What is an emission factor? [Internet]. Climfoot-project.eu. [cited 2023 May 11]. Available from: https://climfoot-project.eu/en/what-emission-factor

For all emission factor and benchmark data sources, please refer to the accompanying "Detailed Guidance and method to calculate the carbon footprint of a clinical trial."

For peer review only

FUNDED BY NIHR | National Institute for Health and Care Research

LIVERPOOL CLINICAL TRIALS CENTRE LCTC

ICR

Enabling lower carbon clinical trials: Development and prototype testing of a method to quantify the carbon footprint of clinical trials to inform future lower carbon clinical trial design

Guidance and method to calculate the carbon footprint of a clinical trial

For peer review only

Data collation quick guide and worksheet

This guidance provides information on how to carbon footprint a clinical trial for the purposes of the NIHR-funded project 'enabling lower carbon clinical trials.'

Within the guidance, clinical trial processes have been sub-divided into the following modules:

1. Trial set up
2. CTU emissions
3. Trial specific meetings and travel
4. Treatment intervention
5. Data collection and exchange
6. Trial supplies and equipment
7. Trial specific patient assessments
8. Samples
9. Laboratory
10. Trial close out

This list is not exhaustive, and it is expected that further activities and modules may need to be added to account for specialist processes in all clinical trial types.

NB: analysis of data does not need to be calculated separately, it is covered by the emissions attributed to trial staff FTE in "CTU emissions" and calculations included within "Data Collection and exchange".

For peer review only - http://bmjopen.bmj.com/site/about/guidelines.xhtml

In addition to this quick guide and worksheet, we have produced a detailed guidance and method document defining the project scope, limitations and assumptions. The detailed guidance contains a more in depth look and explanation of the calculations found in this document, including emission factor and benchmark data sources, and should be referred to when using this worksheet.

Introduction to calculating carbon footprint

A carbon footprint is a measure of greenhouse gases, usually quoted in kg or tonnes of carbon dioxide equivalent (CO_2e). To calculate the carbon footprint of a particular clinical trial process, both 'activity data' and 'emission factors' are required.

An emission factor, also known as a conversion factor, "is a coefficient which allows you to convert activity data into greenhouse gas emissions. It is the average **emission** rate of a given source, relative to units of activity or process/processes."[1]

The activity data is provided by the user and multiplied by the emission factors provided in this guidance document.

Data collation quick guide and worksheet

This data collation quick guide should be used in conjunction with the "Enabling lower carbon clinical trials: Development and prototype testing of a method to quantify the carbon footprint of clinical trials to inform future lower carbon clinical trial design - Detailed Guidance and method to calculate the carbon footprint of a clinical trial". The guidance document provides the detailed explanation of how calculations should be considered and calculated. This quick guide should be used to collate the trial-specific processes, necessary activity data and to record the subsequent calculations. It is important to avoid double-counting activities i.e., modules must not include activities already covered elsewhere in the clinical trial process map. Please complete this worksheet for each trial to be carbon footprinted.

NB: We are using the term 'CTU' to describe the organisation that manages all aspects of central trial management. For some institutions some of those tasks maybe done by groups outside the CTU team e.g., sponsor office/CRO etc.

For peer review only - http://bmjopen.bmj.com/site/about/guidelines.xhtml

FUNDED BY NIHR | National Institute for Health and Care Research

LIVERPOOL CLINICAL TRIALS CENTRE LCTC

ICR

PRIMETIME carbon footprint calculations

PRIMETIME is a post-operative avoidance of radiotherapy trial which recruited 1962 patients across 64 sites in the UK. The trial used electronic data capture for collection of data, data linkage with NHS routine data sources and involved shipment of tissue samples for immunohistochemistry testing and subsequent long-term sample storage.

Module	Examples	Trial activity data	Calculation	Results
**1. Trial set-up**				
1.1. Production of trial documentation to be sent to sites or participants	E.g. Site Investigator File and contents, Site Pharmacy File and contents, CRF Folder and contents, PIS/Cs, GP letters	67 sites initially set up (64 recruited patients)<br><br>3123 patients registered to pre-screening, 1962 patients consented and registered to main study<br><br>SIF: 260 pages, 67 sent (17420 total pages)<br>GP letter: 2 pages, 1962 (3924 total pages)<br>PIS/C form 1: 6 pages, 3123 patients (18738 total pages)<br>PIS/C form 2: 15 pages, 1962 (29430 total pages)<br><br>Number of folders used to send trial documentation = 67 large ring binders (0.5kg)	**Paper:**<br>[no. of page] x 0.005 = paper weight (kg)<br><br>b/w printing: Kg of paper x 0.22438 = $kgCO_2e$<br>Colour printing: Kg of paper x 0.31786 = $kgCO_2e$<br>Materials (paper): Kg of paper x 0.919 = $kgCO_2e$<br><br>Folders: Kg (of cardboard) x 0.821 = $kgCO_2e$<br>Assumption: Weight of lever arch = 0.5kg<br>Assumption: Weight of ring binder = 0.3kg<br><br>SIF:<br>17420 pages x 0.005= 87.1 kg<br>Printing: 87.1 kg x 0.22438 = 19.5 $KgCO_2e$<br>Materials (paper): 87.1 kg x 0.919 = 80 $kgCO_2e$<br><br>Materials (folder): 0.5 kg x 67= 33.5kg<br>33.5 kg x 0.821 = 27.5 $kgCO_2e$<br><br>PIS/C:<br>48168 x 0.005 kg = 240.8 kg<br>Printing: 240.8 kg x 0.31786 = 76.5 $kgCO_2e$<br>Materials: 240.8 kg x 0.919 = 221.3 $kgCO_2e$	447.2 kg CO_2e

For peer review only - http://bmjopen.bmj.com/site/about/guidelines.xhtml

FUNDED BY NIHR | National Institute for Health and Care Research

LIVERPOOL CLINICAL TRIALS CENTRE LCTC

ICR

			<u>GP letter:</u><br>3924 x 0.005 kg = 19.6 kg<br>Printing: 19.6 kg x 0.22438 = 4.4 $kgCO_2e$<br>Materials: 19.6 kg x 0.919 = 18 $kgCO_2e$	
1.2. Provision/postage of trial documentation to sites<br><br>1.3. Provision/postage of documentation to participants by CTU or participating sites<br><br>1.4. Provision/postage of incentives to participant	E.g. Site Investigator File and contents, Site Pharmacy File and contents, CRF Folder and contents, PIS/Cs, GP letters	Estimated weight of SIF:<br>(260 pages x 0.005kg) + 0.5 kg = 1.8kg / 0.00177 tonnes<br><br>Estimated distance of deliveries:<br>Average site distance from ICR-CTSU is 301.8 km<br><br>Number of deliveries: SIF delivered to 67 sites	Delivery weight (tonnes) x distance (km) = t.km<br><br>For road freight: t.km x 0.19443 = $kgCO_2e$<br><br>For air freight: t.km x required emission factor below = $kgCO_2e$<br><br>- Domestic (to/from UK) = 4.98549<br>- Short-haul (to/from UK) = 2.55439<br>- Long-haul (to/from UK) = 1.13047<br>- International (to/from non-UK) = 1.13047<br><br>For delivery of trial supplies to patients or GP, if unknown, use 17.4km as distance from hospital to patient, or hospital to GP.<br><br><u>Calculations:</u><br>SIF delivery:<br>- 0.00177 x 301.8km = 0.53 t.km<br>- 0.53 x 0.19443 = 0.103<br>- x 67 sites = 6.9 $kgCO_2e$<br><br>GP letter:<br>Weight of letter = 2x5g + envelope (7g)= 17g	7.01 $kgCO_2e$

For peer review only

For peer review only - http://bmjopen.bmj.com/site/about/guidelines.xhtml

FUNDED BY NIHR | National Institute for Health and Care Research

LIVERPOOL CLINICAL TRIALS CENTRE LCTC

ICR

			x1962 patients = 33354g/0.033 tonnes<br>0.033 x 17.4km = 0.5742 t.km<br>0.5742 t.km x 0.19443 = 0.11 kgCO_2e	
**2. CTU emissions**				
2.1. Energy consumption at CTU according to trial staff FTE	E.g. energy consumption per square metre of air-conditioned office space	Trial duration: 10 years<br><br>Trial staff FTE: the total FTE over the 10-year trial period was 7.1	**Energy consumption for 1 FTE for 1 year** = 193.28 kgCO_2e<br><br>Multiply by the number of years and FTE applicable<br><br>7.1 X 193.28 kgCO_2e = 1372.3 kgCO_2e	1372.3 kgCO_2e
2.2. Heating	E.g. energy consumption at coordination centre attributed to heating (natural gas)	Trial duration: 10 years<br><br>Trial staff FTE: the total FTE over the 10-year trial period was 7.1	**Heating for 1 FTE for 1 year =** 431.56 kgCO_2e<br><br>Multiply by the number of years and FTE applicable<br><br>7.1 x 431.56 kgCO_2e = 3064 kgCO_2e	3064 kgCO_2e
2.3. Trial team commuting	E.g. Car, rail, bus, walking etc	Trial duration: 10 years<br><br>Trial staff FTE: the total FTE over the 10-year trial period was 7.1	For 1 FTE for 1 year, total average commuting emissions = 1027.8 kgCO_2e<br><br>Multiply by the number of years and FTE applicable<br><br>7.1 x 1027.8 kgCO_2e = 7297 kgCO_2e	7297 kgCO_2e
**3. Trial specific meetings and travel**				
3.1. Visits and travel to site<br>3.2. Travel to meetings	E.g. Feasibility, site initiation and monitoring visits,	No feasibility visits, monitoring, audits, or conferences.	Number of passengers x total distance (km) = p.km	579.3 kgCO_2e

For peer review only

FUNDED BY NIHR | National Institute for Health and Care Research

	audits, inspections, Trial Management Group (TMG), Trial Steering Committee (TSC), Independent Data Monitoring Committee (IDMC), and investigator meetings, Patient and Public Involvement and Engagement (PPIE), conferences, scientific meetings etc	<u>Site initiation visits</u><br>66 virtual (+6 refresher sessions), 1 in person<br><br><u>IDMC meetings</u><br>5 IDMC meetings, all virtual, total of 31 instances of attendance<br><br><u>TMG meetings</u><br>Total distances travelled by participants or number of virtual attendees:<br>- Meeting 1- 1760.6km<br>- Meeting 2- 1437.3km<br>- Meeting 3 – virtual, 12 attendees<br>- Meeting 4 – 1624.8km<br>- Meeting 5 – virtual, 25 attendees<br>- Meeting 6 – virtual, 18 attendees<br>- Meeting 7 – virtual, 23 attendees<br><br><u>TSC meetings</u><br>5 attendees to each TSC meeting, 1 hour duration, some teleconference and some in person. Distance staff travelled from CTU = 22.5km	For national rail: p.km x 0.04441= $kgCO_2e$<br><br>For flights: p.km x relevant emission factor below:<br>- Domestic (average): 0.27278<br>- Short-haul (average) to/from UK: 0.17034<br>- Long-haul (average) to/from UK: 0.21423<br>- International (average) to/from non-UK: 0.20373<br><br>NB: Distances may be calculated using google maps and calculated from CTU to destination<br><br>**Videoconferencing** = 157 grams CO_2e per hour.<br><br>Calculations:<br><u>Site Initiation Visits:</u><br>Virtual SIVs: 72 X 157 g CO2e/hr = 11304g/11.3 $kgCO_2e$<br><br>1 in person SIV: 1 attendee, 24.1KM.<br>1 x 2 x 24.1 km = 48.2 p.km<br>48.2 p.km x 0.04441= 2.1 $kgCO_2e$<br><br><u>IDMC</u><br>IDMC: 31 x 157 g CO2e/hr = 4867 g/4.9 $kgCO_2e$<br><br><u>TMG meetings</u> - assumption: rail used to travel	

For peer review only - http://bmjopen.bmj.com/site/about/guidelines.xhtml

FUNDED BY NIHR | National Institute for Health and Care Research

LIVERPOOL CLINICAL TRIALS CENTRE LCTC

ICR

			TMG meeting 1: 1x2x1760.6km = 3521.2 p.km.<br>3521.2 p.km x 0.04441 = 156.4 $kgCO_2e$<br>TMG meeting 2: 1x2x1437.3km= 2874.6 p.km<br>2874.6 p.km x 0.04441 = 127.7 $kgCO_2e$<br>TMG meeting 3: teleconference, 2 hours, 12 external attendees. 157 g CO2e/hr x 2 x 12 = 3768g/3.77 $kgCO_2e$<br>TMG meeting 4: 1 x 2 x 1624.8km = 3249.6 p.km x 0.04441 = 144.3 $kgCO_2e$<br>TMG meeting 5: 13-14:30 via zoom, 25 attendees. 157 g CO2e/hr x 1.5 x 25 = 5887.5 g/5.89 $kgCO_2e$<br>TMG meeting 6: 157 g CO2e/hr x 1.5 x 18 = 4239 g/4.24 $kgCO_2e$<br>TMG meeting 7: 1hr, Teams meeting, 23 attendees - 157 g CO2e/hr x 23 = 3611g/3.6 $kgCO_2e$<br><br>TSC<br>2016, teleconference: 157 g CO2e/hr x 5 = 785g/0.8 $kgCO_2e$<br>2017: 5x(2x22.5km) = 225 p.km x 0.04441= 10 $kgCO_2e$<br>2018 = 10 $kgCO_2e$<br>2019 = 10 $kgCO_2e$<br>2021 = 10 $kgCO_2e$<br><br>Other:<br>➢ 2 virtual conferences: 157g CO2e/hr x 2 = 0.314 $kgCO_2e$	

For peer review only

FUNDED BY NIHR National Institute for Health and Care Research

			➢ Public Health England (PHE) teleconference, 2 attendees: 2 x 157gCO_2e/hr = 0.314 kgCO_2e<br>➢ Sample management and logistics planning meeting, 5 attendees: 5 x 157 g CO2e/hr = 0.785 kgCO_2e<br>➢ Data management meetings, 20 instances of teleconferencing: 20 x 157 g CO2e/hr = 3140g/3.14 kgCO_2e<br>➢ LCRN Meeting re CPMS Uploads, 9 attendees - 9 x 157 g CO2e/hr = 1.413 kgCO_2e<br>➢ Primetime Team meetings, 132 instances of people teleconferencing for 1 hour. 132 x 157 = 20724 g/20.7 kgCO_2e<br>➢ CI_TC updates, 303 instances of people teleconferencing for 1 hour: 303 x 157 g CO2e/hr = 47571 g/47.6 kgCO_2e	
3.3. Hotel stays	E.g. TMG meetings, monitoring visits, audits, inspections etc	Number of rooms: 8 rooms (for one person for one night)	For UK: number of hotel rooms x number of nights x 13.9 = kgCO_2e<br><br>For UK (London) = number of rooms x number of nights x 13.8 = kgCO_2e.<br><br>For other countries use conversion factors from orange 'hotel stay' tab: conversion-factors-2021-full-set-advanced-users.xlsm (live.com)<br>Alternatively, you may use cost-based method: £ spent x 0.388 = kgCO_2e	250.2 kgCO_2e

For peer review only - http://bmjopen.bmj.com/site/about/guidelines.xhtml

			Assumption: 8 stays (for TMG meetings)<br>8 x 13.9 = 250.2 $kgCO_2e$	
3.4. Sustenance	E.g. meeting lunches, hotel dinners	Number of lunches or dinners:<br>3 in-person TMG meetings, 52 total attendees<br>4 in person TSC meetings, 20 total attendees<br><br>Total = 72 lunches for TMG and TSC meeting participants	Meeting lunches or hotel dinners (vegetarian) = 2.6 $kgCO_2e$ per meal per person<br><br>Meeting lunches or hotel dinners (meat) = 5.92 $kgCO_2e$ per meal per person<br><br>Assumption: unable to determine if meat or vegetarian therefore assumed 50/50<br>(36 x 2.6) + (36 x 5.92) = 306.7 $kgCO_2e$	306.7 $kgCO_2e$
**4. Intervention*** **4.1. Physical (IMP)**<br>**4.2. Clinical (non-IMP)**<br>**4.3. Other (not captured above)**<br>**Please fill out the section most relevant to the intervention being investigated.**				
**4.1. Physical**				
4.1.1, 4.1.2. Movement of intervention, or materials required to deliver the intervention	E.g. movement of intervention from manufacturing site to distribution site, shipment of IMP to participating sites or direct to participant	Total weight of deliveries:<br><br>Total distance of deliveries:	Carry out freight calculation as described in section 1.2.<br><br>For refrigerated freight, increase the total $kgCO_2e$ associated with freight by 15%.<br><br>Frozen freight:	N/A – Avoidance of radiotherapy trial

* As per assumptions detailed in the guidance, manufacture of the intervention is considered out of scope. This section defines all processes relating to providing and delivering the trial intervention that are over and above routine care.

For peer review only - http://bmjopen.bmj.com/site/about/guidelines.xhtml

FUNDED BY NIHR | National Institute for Health and Care Research LIVERPOOL CLINICAL TRIALS CENTRE LCTC ICR

			Dry ice has a carbon footprint of **1.81kg CO_2e for 1 kg dry ice** produced/used.<br><br>When calculating the overall emissions of frozen freight, as well as the 1.81 kgCO_2e per 1kg attributed to manufacture, include the weight (kg) of dry ice used in the weight of the freight calculation in section 1.2.<br><br>In the absence of activity data, assume 1kg of dry ice is used per individual sample shipping box.	
4.1.3. Materials required for the packaging and shipment of IMP	E.g. cardboard, cold storage boxes, polystyrene	Number of cold storage boxes:<br><br>Kg of cardboard/ polystyrene:	Single use sample cold storage box = 25.2 kgCO_2e per box<br><br>Reusable sample cold storage box = 2.2 kgCO_2e per box<br><br>Kg (cardboard) x 0.821 = kgCO_2e<br>Kg (polystyrene) x 3.778 = kgCO_2e	N/A – avoidance of radiotherapy trial
4.1.4. Destruction of overage	E.g. incineration of IMP	Estimated weight of overage incinerated:	Kg of waste x 2.4252 = kgCO_2e	N/A – avoidance of radiotherapy trial
**4.2. Clinical** e.g., radiotherapy, device, surgical.<br>NB: not all calculations will be relevant to all interventions. **This section of the method will be further developed as we carbon footprint more trials, so please inform us if your protocol specifies an activity that has not been included, and we will help to determine the associated carbon footprint.**				
4.2.1 Movement of the intervention, or resources	E.g. movement of intervention from	Estimated total weight of delivery:	Please refer to section 1.2 and 4.1.1, 4.1.2.	N/A – avoidance of

For peer review only

FUNDED BY NIHR | National Institute for Health and Care Research

LIVERPOOL CLINICAL TRIALS CENTRE LCTC

ICR

required to deliver the intervention	manufacturing site to distribution site, shipment of intervention to participating sites.	Estimated total distance of deliveries:		radiotherapy trial
4.2.2 Materials required for the packaging and shipment of the intervention	E.g. cardboard, cold storage boxes, polystyrene	Number of cold storage boxes:<br><br>Kg of cardboard/ polystyrene:	Please refer to section 4.1.3.	N/A – avoidance of radiotherapy trial
4.2.3 Utilities required for delivery of the intervention	E.g. Hospital utilities if the intervention is delivered within a hospital	Hospital staff FTE required:	Please refer to section 7.3 to calculate the emissions attributed to hospital utilities if required to deliver the intervention.	N/A – avoidance of radiotherapy trial
4.2.4 Activities or resources required/relating to delivery of the intervention	E.g. consumables, surgical time, specialist equipment, incineration of surgical waste		Please refer to section 7.2 for consumables, surgery and other activities that may be relevant to the delivery of the intervention, but please take care to avoid double counting.<br><br>To calculate the emissions attributed to incineration, e.g. of surgical waste, please refer to section 4.1.4.	N/A – avoidance of radiotherapy trial
**4.3. Other**<br>NB: not all calculations will be relevant to all interventions. **This section of the method will be further developed as we carbon footprint more trials, so please inform us if your protocol specifies an activity that has not been included, and we will help to determine the associated carbon footprint.**				
4.3.1 Movement of the intervention to the participant or participating site	E.g. shipment of intervention to participating sites or direct to participant	Estimated weight of delivery:<br><br>Estimated distance of deliveries:	Please refer to section 1.2.	N/A – avoidance of radiotherapy trial
4.3.2 Materials required for packaging and	E.g. cardboard, cold storage boxes, polystyrene	Number of cold storage boxes:<br><br>Kg of cardboard/ polystyrene:	Please refer to section 4.1.3.	N/A – avoidance of

For peer review only - http://bmjopen.bmj.com/site/about/guidelines.xhtml

shipment of the intervention				radiotherapy trial
4.3.3 Materials or resources required for delivery of the intervention	E.g. software, booklets, specialist equipment		For printing and paper, please refer to section 1.1.	N/A – avoidance of radiotherapy trial
4.3.4 Travel required to facilitate delivery of the intervention	E.g. to deliver training, conduct interviews etc	Estimated distance travelled, and number of passengers (p.km):	Please refer to section 3.1, 3.2.	N/A – avoidance of radiotherapy trial
**5. Data collection and exchange**<br>NB: analysis of data does not need to be calculated separately, it is covered by the emissions attributed to trial staff FTE in "CTU emissions" and calculations included within "Data Collection and exchange".				
5.1. Data collection and query exchange between CTU and sites	E.g. CRFs, EDC completion and query resolution, scans copied to CDs	Postage from sites to ICR-CTSU:<br>Only the eligibility, pre-screening and registration checklists posted to CTU from sites.<br>-Total pages from all sites = 14094.<br>-Average site distance from ICR-CTSU: 301.8 km<br><br>Printing by sites for their SIF:<br>Estimate 419 pages printed by each site for their SIF, 63 sites = 26397 total pages<br><br>Paper CRFs:<br>28 folders/box files containing paper CRFs in ICR-CTSU.	Postage of documents from sites:<br>14094 x 0.005 = 70.47 kg / 0.07 tonnes<br>0.07 x 301.8 = 21.126 t.km<br>21.126 t.km x 0.19443 = 4.1 $kgCO_2e$<br><br>Printing by sites for their SIF:<br>26397 x 0.005 = 131.985 kg<br>Printing: 131.985 kg x 0.22438 = 29.6 $kgCO_2e$<br>Material: 131.985 kg x 0.919 = 121.3 $kgCO_2e$<br><br>Paper CRFs<br>14000 x 0.005 = 70kg<br>Printing: 70 kg x 0.22438 = 15.7 $kgCO_2e$<br>Materials: 70kg x 0.919 = 64.3 $kgCO_2e$<br><br>Site documents stored in ICR-CTSU:<br>6300 x 0.005= 31.5 kg<br>Printing: 31.5 kg x 0.22438 = 7.1 $kgCO_2e$	271.1 $kgCO_2e$

For peer review only - http://bmjopen.bmj.com/site/about/guidelines.xhtml

		Assumption: box file contains 500 pieces of paper<br>500 x 28 = 14000 total pages<br><br>Site documents stored in ICR-CTSU:<br>18 folders with TMFs and site documents stored in CTU.<br>Assumption: folder holds 350 pages.<br>18 x 350 = 6300 total pages	Materials: 31.5 kg x 0.919 = 29 $kgCO_2e$	
5.2. Data sent direct from participants to CTU or participating sites	E.g. Questionnaires, patient diaries, wearables	Estimated weight and distance of delivery (t.km):<br><br>Device used and time taken to complete electronic questionnaires:	For paper questionnaires, please refer to section 1.1. for the carbon footprint of producing the materials and section 1.2. for postage (freight).<br><br>For use of smart watches and other devices see section 6.3.<br><br>**Electronic questionnaires**<br>Add the emissions attributed to data storage and transmission to the emissions attributed to using a device to complete the questionnaire.<br><br>Web surfing (data storage and transmission) = 9.441 g CO2e/hr (10 mins = 1.57 g CO_2e)<br><br>Choose from the below:<br>- Desktop computer = 0.18079 kg CO_2e per hour<br>- Laptop = 0.028719 kg CO_2e per hour	N/A

For peer review only

FUNDED BY NIHR | National Institute for Health and Care Research · LIVERPOOL CLINICAL TRIALS CENTRE LCTC · ICR

			- Tablet = 0.027397 kg CO_2e per hour<br>- Smartphone = 0.015068 kg CO_2e per hour	
5.3. Data from labs to CTU<br>5.4. Data from other collaborators to CTU	E.g. Laboratory patient results, data linkage	GB required for data storage/transmission:<br>Duration of data storage:<br><br>Total £ spent on computer services: £49000	Data storage and transmission = estimate 1.365 kg CO_2e per GB per year.<br><br>For computer services such as data linkage:<br>£ x 0.149 = kgCO_2e<br><br>£49000 x 0.149 = 7301 kgC0_2e	7301 kgC0_2e
**6. Trial supplies and equipment**				
6.1. Equipment used by CTU	E.g. computers, laptops, printers, software	Total £ spent on office machinery and computers for trial: £4500	For any new office machinery and computers purchased specifically for trial:<br>£ x 0.387 = kgCO_2e<br>£4500 x 0.387 = 1741.5 kgCO_2e	1741.5 kgCO_2e
6.2. Equipment and supplies used by participating sites supplied by CTU	E.g. centrifuge, fridge, freezer	Estimated weight and distance of deliveries (t.km):	For the shipment of equipment to participating sites, please refer to section 1.2.<br><br>For the use of a centrifuge, please refer to section 9.2., for a fridge or freezer please refer to section 9.3.	N/A
6.3. Equipment and supplies provided to participants specifically for the trial	E.g. wearables, smartphone, tablet	Number of devices and duration of their usage:<br><br>Estimated weight and distance of deliveries (t.km):	**Smartphone =** 55 kgCO_2e from manufacture and add 5.5 kgCO_2e per year of usage.	N/A

For peer review only - http://bmjopen.bmj.com/site/about/guidelines.xhtml

FUNDED BY NIHR | National Institute for Health and Care Research
LIVERPOOL CLINICAL TRIALS CENTRE LCTC
ICR

			**Tablet =** 119 $kgCO_2e$ from manufacture and add 10 $kgCO_2e$ per year of usage.<br><br>**Wearables/smart watch** = 30.1 kg CO_2e for manufacture and add 1.633 kg CO_2e per year of usage.<br><br>**To calculate the carbon footprint associated with shipment of the devices, please refer to section 1.2.**	
**7. Trial specific patient assessments**				
7.1. Patient travel for study visits that are in addition to standard of care	E.g. Eligibility and screening assessments, trial-specific assessments and procedures	Number of times patient is required to travel (in addition to standard of care): 1623 patients had to travel for 5 additional above standard of care mammograms	Emissions associated with one patient visit to hospital (UK) = 5.8 $kgCO_2e$ (this includes both the out and back journeys)<br>Emissions associated with one patient visit to GP surgery (UK) = 1.12 $kgCO_2e$ (this includes both the out and back journeys)<br><br>5.8 $kgCO_2e$ x 5 x 1623 = 47067 $kgCO_2e$	47067 $kgCO_2e$
7.2. Materials and activities required for study assessments that are in addition to standard of care	E.g. Laboratory tests, imaging assessments, clinical activities relating to intervention for example administering of study drug, biopsy.	5 above standard of care mammograms for each patient in very low risk and no RT group (1623)<br><br>Assumption: 2 visits where consumables required	Consumables = 0.30 $kgCO_2e$ per patient per trial appointment where consumables (such as gloves) required<br><br>- 1 MRI = 24.7 kg CO_2e<br>- 1 CT scan = 9.2 $kgCO_2e$<br>- 1 CXR = 0.8 $kgCO_2e$/scan<br>- 1 hour in surgery = 53 kg CO_2e	7977.3 $kgCO_2e$

For peer review only - http://bmjopen.bmj.com/site/about/guidelines.xhtml

			- 1 low intensity (general ward) bed day = 37.9 kg CO_2e<br>- 1 high intensity (ICU) bed day = 103 kgCO_2e<br>- 15 sessions of breast radiotherapy = 5.7 kg CO_2e<br>- 20 sessions of prostate radiotherapy = 15.3 kg CO_2e<br><br>Blood tests:<br>- 82 g CO_2e for coagulation profile<br>- 116 g CO_2e for full blood examination<br>- 49 g CO_2e for arterial gas assessment<br>- 99 g CO_2e for urea and electrolyte assessment<br>- 0.5 g CO_2e for C-reactive protein<br><br>**Please note that the above figures for blood tests include the materials and consumables required for sample collection, phlebotomy and analysis, as well as power consumption by pathology analysers.**<br><br><u>Calculations:</u><br>Consumables:<br>- 0.30 kgCO_2e x 2989 (number of patients who received ki67 testing) = 896.7 kgCO_2e<br>- 0.30 x 1962 (for physical appointment for those registered to study) = 588.6 kgCO_2e	

For peer review only - http://bmjopen.bmj.com/site/about/guidelines.xhtml

FUNDED BY NIHR | National Institute for Health and Care Research

			- Total = 1485.3 $kgCO_2e$<br><br>Scans: 5 x 0.8 $kgCO_2e$ x 1623 = 6,492 $kgCO_2e$	
7.3. Utilities required for study assessments that are in addition to standard of care	E.g. energy consumption per square metre of hospital space according to trial staff FTE, taking into account time required for CRF completion and study assessments, consent etc	Hospital trial staff FTE: total estimated FTE = 5.5 from 2017 - 2022†	Electricity: 1 FTE 1 year = 364.9 $kgCO_2e$ Multiply by the number of years and FTE applicable<br><br>364.9 $kgCO_2e$ x 5.5 = 2007 $kgCO_2e$<br><br>Heating: 1 FTE, 1 year = 685.7 $kgCO_2e$ Multiply by the number of years and FTE applicable<br><br>685.7 $kgCO_2e$ x 5.5 = 3771.4 $kgCO_2e$	5778.4 $kgCO_2e$
**8. Samples**				
8.1. Materials involved	E.g. sample collection kit and packaging for shipment	Kg of material:<br>- 30.6 kg slide container (pp)<br>- 243.8 kg jiffy bag<br>- 39 kg paper	The emissions attributed to sample collection consumables for common blood tests are included in the blood tests listed in section 7.2.<br><br>To calculate the carbon footprint of other common materials, multiply the weight in kg by the relevant emission factor below to produce $kgCO_2e$.<br>- Average plastics: 3.116<br>- Plastics (average film): 2.754<br>- Plastics (Average rigid): 3.277<br>- Plastics (PP): 3.105	397.9 $kgCO_2e$

† Hospital trial staff FTE was estimated over 6 years rather than the 10-year trial duration because the final 4 years are follow up via routine data sources.

For peer review only - http://bmjopen.bmj.com/site/about/guidelines.xhtml

FUNDED BY NIHR | National Institute for Health and Care Research

			- Plastics (PET): 4.032<br>- Glass: 3.413<br>- Paper: 0.919<br>- Board: 0.821<br><br>Slide containers = 30.6 kg x 3.105 (pp) = 95 $kgCO_2e$<br><br>Paper (materials) = 39kg x 0.919 = 35.8 $kgCO_2e$<br>Paper (printing) = 39kg x 0.22438 = 8.8 $kgCO_2e$<br><br>Jiffy bag = 243.8 kg x 0.919= 224.1 $kgCO_2e$<br><br>Tracking slip: 2 copies of each tracking slip on top of one included in the kit– 2989 x 2 = 5978 pages<br>Printing: 5978 pages x 5g = 29890g/29.89kg<br>29.89 kg x 0.22438 = 6.7 $kgCO_2e$<br>26.9kg x 0.919 = 27.5 $kgCO_2e$	
8.2. Movement of sample kit materials from manufacturer to CTU<br>8.3. Movement of sample kits from CTU/distributor to participating Sites<br>8.4. Movement of samples from participating sites or	E.g. shipment of blood tubes for sample kits to CTU	8.2. Movement of sample kit materials from manufacturer to CTU<br>Envelopes:<br>- Weight = 0.004 tonnes, total<br>- Total distance transported = 849.6km (141.6km x 6 deliveries)<br><br>Slide mailing containers:<br>-Weight = 0.00204 tonnes	Please refer to section 1.2. for freight and 4.1. for refrigerated or frozen freight.<br><br>**8.2: Movement of sample kit materials from manufacturer to CTU**<br><br>Envelopes:<br>0.004 tonnes x 849.6km = 3.3984 t.km<br>3.3984 t.km x 0.19443 = 0.66 $kgCO_2e$	40.26 $kgCO_2e$

For peer review only - http://bmjopen.bmj.com/site/about/guidelines.xhtml

FUNDED BY NIHR | National Institute for Health and Care Research

patients to central laboratory.		-Total distance transported = 6910.3 km (363 km x 19 deliveries)<br><br>8.3. Movement of sample kits from CTU to participating Sites:<br>-Average distance from CTU to participating site = 301.8km<br>- Shipment weight = 0.00053 tonnes<br>- 630 sample kit shipments<br><br>8.4. Movement of samples from participating sites to laboratory.<br>-Cambridge lab – average distance to participating site = 239.5km, 938 shipments<br>-RMH lab - average distance to participating site = 258km, 1094 shipments<br>-Glasgow lab - average distance to participating site = 467.8km, 957 shipments	Slide mailing containers:<br>0.00204 tonnes x 6910.3 km = 14.097 t.km<br>14.097 t.km x 0.19443 = 2.7 $kgCO_2e$<br><br>**8.3. Movement of sample kits from CTU/distributor to participating Sites**<br><br>0.00053 x (630 x 295.88) = 100.77 t.km x 0.19443 = 19.6 $kgCO_2e$<br><br>**8.4. Movement of samples from participating sites or patients to central laboratory.**<br><br>Cambridge laboratory: 0.0000935 x (239.5km x 938) x 0.19443 = 4.1 $kgCO_2e$<br>RMH laboratory: 0.0000935 X (258 km X 1094) X 0.19443 = 5.1 $kgCO_2e$<br>Glasgow laboratory: 0.0000935 x (467.8km x 957) x 0.19443 = 8.1 $kgCO_2e$	
**9. Laboratory**				
9.1. Emissions attributed to lab utilities according to staff FTE	E.g. energy consumption per square metre of laboratory space	Trial staff FTE and trial duration: 3 labs, 1 staff member in each. Trial is 5% of each of their FTE for 4 years and 2% for 1 year.	**Electricity:**<br>1747.2 $kgCO_2e$ per FTE per year<br>Multiply 1747.2 $kgCO_2e$ by the number of years and FTE applicable	2061.4 $kgCO_2e$

For peer review only - http://bmjopen.bmj.com/site/about/guidelines.xhtml

FUNDED BY NIHR | National Institute for Health and Care Research

	according to trial staff FTE		1747.2 $kgCO_2e$ x 0.05 = 87.36 $kgCO_2e$<br>87.36 $kgCO_2e$ x 4 years = 349.44 $kgCO_2e$<br>1747. 2 $kgCO_2e$ x 0.02 = 34.944 $kgCO_2e$<br>349.44 + 34.944 = 384.4 $kgCO_2e$<br>384.4 x 3 lab staff = 1153.2 $kgCO_2e$<br><br>**Heating:**<br>1376 $kgCO_2e$ per FTE per year<br>Multiply 1376 $kgCO_2e$ by the number of years and FTE applicable<br><br>1376 $kgCO_2e$ x 0.05 = 68.8 $kgCO_2e$<br>68.8 $kgCO_2e$ x 4 = 275.2 $kgCO_2e$<br>1376 x 0.02 $kgCO_2e$ = 27.52 $kgCO_2e$<br>275.2 $kgCO_2e$ + 27.52 $kgCO_2e$ = 302.72 $kgCO_2e$<br>Total = 302.72 $kgCO_2e$ x 3 = 908.16 $kgCO_2e$	
9.2. Materials/equipment /consumables used in processing and analysis of samples	E.g. centrifuges, refrigerators	kWh usage of equipment, hours used:<br><br>Kg of material:	To avoid double counting, use of equipment will be included in lab staff FTE if calculated.<br><br>If the trial does not involve a central lab, but there is still sample processing on site, please see below. For storage of samples, please see section 9.3.<br><br>To calculate the emissions of a piece of equipment, multiply the power consumption by hours used to get a kWh value. Finally multiply kWh by the electricity emission factor (0.273).	N/A

For peer review only

FUNDED BY NIHR | National Institute for Health and Care Research

LIVERPOOL CLINICAL TRIALS CENTRE LCTC

ICR

			Example: use of a 310-Watt centrifuge for 15 minutes<br>- 310 Watts x 0.25 (hours) = 77.5 kWh<br>- 77.5 kWh x 0.273 = 21.2 kgco$_2$e<br><br>Consider the centrifuge capacity and multiply by the number of uses required.	
9.3. Storage of samples	E.g. utilities and ultra-low temperature freezer	Length of storage:<br><br>Amount of refrigerator/freezer space required:	Storage in fridge/-20 freezer:<br>- 298.9 kgCO_2e per year<br>- Multiply by number of years stored<br><br>Storage in an ultra-low/-80 freezer:<br>- 2192.2 kgCO_2e per year<br>- Multiply by number of years stored<br><br>NB: this is for a whole freezer; you will need to make an assumption about the amount of space in the freezer that the trial samples take up.	N/A
**10. Trial close out**				
10.1. Storage and archiving of essential trial documentation and data	E.g. Hospital files, lab files, trial guidance documents etc	Duration of storage: 10 years<br><br>Amount of space required for storage (m^2): 4m^2<br><br>Storage location: CTU (office)	Carbon footprint associated with 1m^2 for 1 year:<br>- Office: 16.1 kgC0$_2$e<br>- Laboratory: 43.7 kgc0$_2$e<br>- Warehouse: 7.4 kgC0$_2$e<br>- Health building: 22.1 kgCO_2e<br><br>Choose the most suitable building type and multiply by number of years and m^2 necessary.<br><br>**Heating**	2126.6 kgCO_2e

For peer review only

FUNDED BY NIHR | National Institute for Health and Care Research
LIVERPOOL CLINICAL TRIALS CENTRE LCTC
ICR

			Carbon footprint associated with 1 m^2 for 1 year:<br><br>- Office: 36 $kgCO_2e$<br>- Laboratory: 34.4 $kgCO_2e$<br>- Warehouse: 13.3 $kgCO_2e$<br>- Health building: 41.6 $kgCO_2e$<br><br>Choose the most suitable building type and multiply by number of years and m^2 necessary.<br><br>**For electronic storage,** estimate 1.365 kg CO_2e per GB per year.<br><br>Calculations:<br>Electricity: 16.1 $kgCO_2e$ x 4 x 10 = 644 $kgCO_2e$<br><br>Heating: 36 $kgCO_2e$ x 4 x 10 years = 1428 $kgCO_2e$<br><br>Electronic storage:<br>PRIMETIME trial database = ~ 4GB<br>Assumption: storage for 10 years<br>1.365 kg CO_2e x 4 x 10 = 54.6 $kgCO_2e$	
10.2. Storage and destruction of biological samples	E.g. blood, tissue, urine etc	Duration of storage: 10 years<br><br>Amount of space required for storage (m^2): 2m^2<br><br>Storage location: Health building	See section 9.3. for storage of refrigerated or frozen samples.<br><br>See section 10.1 for storage of ambient samples.	1274 $kgCO_2e$

For peer review only - http://bmjopen.bmj.com/site/about/guidelines.xhtml

			Electricity:<br>22.1 $kgCO_2e$ x2 x 10 = 442 $kgCO_2e$<br><br>Heating:<br>41.6 $kgCO_2e$ x 2 x 10 = 832 $kgCO_2e$	
10.3. Return of equipment and supplies from participating sites to CTU	E.g. wearables, unused or expired equipment and supplies	Estimated weight and distance of delivery (t.km):	See section 1.2. (freight).	N/A

PRIMETIME carbon footprint summary

Module	$KgCO_2e$
Trial set up	454.2
CTU emissions	11733.3
Trial staff meetings and travel	1136.2
Treatment intervention	0
Data collection and exchange	7572.1
Trial supplies and equipment	1741.5
Trial specific patient assessments	60822.7
Samples	438.2
Laboratory	2061.4

For peer review only - http://bmjopen.bmj.com/site/about/guidelines.xhtml

Analysis and trial close out	3400.6
**Total =**	89360.2 kgCO_2e/ 89.4 tonnes

References

[1] What is an emission factor? [Internet]. Climfoot-project.eu. [cited 2023 May 11]. Available from: https://climfoot-project.eu/en/what-emission-factor

For all emission factor and benchmark data sources, please refer to the accompanying "Detailed Guidance and method to calculate the carbon footprint of a clinical trial."

For peer review only - http://bmjopen.bmj.com/site/about/guidelines.xhtml

BMJ Open

Quantifying the carbon footprint of clinical trials: guidance development and case studies

Journal:	*BMJ Open*
Manuscript ID	bmjopen-2023-075755.R1
Article Type:	Communication
Date Submitted by the Author:	06-Dec-2023
Complete List of Authors:	Griffiths, Jessica; The Institute of Cancer Research,<br>Fox, Lisa; The Institute of Cancer Research<br>Williamson, Paula; The University of Liverpool<br>Low Carbon Clinical Trials Group, group authorship; various
**Primary Subject Heading**:	Research methods
Secondary Subject Heading:	Communication
Keywords:	Clinical Trial, Decision Making, STATISTICS & RESEARCH METHODS

SCHOLARONE™
Manuscripts

I, the Submitting Author has the right to grant and does grant on behalf of all authors of the Work (as defined in the below author licence), an exclusive licence and/or a non-exclusive licence for contributions from authors who are: i) UK Crown employees; ii) where BMJ has agreed a CC-BY licence shall apply, and/or iii) in accordance with the terms applicable for US Federal Government officers or employees acting as part of their official duties; on a worldwide, perpetual, irrevocable, royalty-free basis to BMJ Publishing Group Ltd ("BMJ") its licensees and where the relevant Journal is co-owned by BMJ to the co-owners of the Journal, to publish the Work in BMJ Open and any other BMJ products and to exploit all rights, as set out in our licence.

The Submitting Author accepts and understands that any supply made under these terms is made by BMJ to the Submitting Author unless you are acting as an employee on behalf of your employer or a postgraduate student of an affiliated institution which is paying any applicable article publishing charge ("APC") for Open Access articles. Where the Submitting Author wishes to make the Work available on an Open Access basis (and intends to pay the relevant APC), the terms of reuse of such Open Access shall be governed by a Creative Commons licence – details of these licences and which Creative Commons licence will apply to this Work are set out in our licence referred to above.

Other than as permitted in any relevant BMJ Author's Self Archiving Policies, I confirm this Work has not been accepted for publication elsewhere, is not being considered for publication elsewhere and does not duplicate material already published. I confirm all authors consent to publication of this Work and authorise the granting of this licence.

For peer review only

Quantifying the carbon footprint of clinical trials: guidance development and case studies

Jessica Griffiths (Corresponding Author) *, Lisa Fox*, Paula Williamson§ on behalf of the Low Carbon Clinical Trials Group

*Clinical Trials and Statistics Unit at The Institute of Cancer Research, London, UK

§MRC/NIHR Trials Methodology Research Partnership, Department of Health Data Science, University of Liverpool, UK

Corresponding author email address: Jessica.Griffiths@icr.ac.uk

Low Carbon Clinical Trials Group Co-authors: Fiona Adshead, Sustainable Healthcare Coalition, UK; Rustam Al-Shahi Salman, Edinburgh Clinical Trials Unit, University of Edinburgh, UK; Craig Anderson, The George Institute, Australia; Emma Bedson, Liverpool Clinical Trials Centre, UK; Judith Bliss, Clinical Trials and Statistics Unit, The Institute of Cancer Research London, UK; Ana Boshoff, Imperial Clinical Trials Unit, Imperial College London, UK; Xiaoying Chen, The George Institute, Australia; Denise Cranley, Edinburgh Clinical Trials Unit, University of Edinburgh, UK; Peter Doran, University of Galway, Ireland; Carrol Gamble, Liverpool Clinical Trials Centre, UK; Kerenza Hood, Centre for Trials Research, Cardiff University, UK; Naomi McGregor, Newcastle Clinical Trials Unit, Newcastle University, UK; Carolyn McNamara, Technology & Innovation, KCR, UK; Elis Midha, School of Medicine, Cardiff University, UK; Keith Moore, Sustainable Healthcare Coalition, UK; Alexis M Perkins, Imperial Clinical Trials Unit, Imperial College London, UK; Sarah Pett, MRC Clinical Trials Unit at UCL, Institute of Clinical Trials and Methodology, UCL, London, UK; Matthew R Sydes, MRC Clinical Trials Unit at UCL, Institute of Clinical Trials and Methodology, UCL, London, UK.

ABSTRACT

Background: The urgency of the climate crisis requires attention from biomedical research, not least clinical trials which can involve significant greenhouse gas emissions. The Low Carbon Clinical Trials

For peer review only

(LCCT) Working Group set out a strategy to reduce the emissions of clinical trials, starting with the development of a method to measure their carbon footprint (CO_2e).

Methods: As a first step, we developed a process map defining clinical trial core activities. Corresponding emission factors were sourced to convert activity data into greenhouse gas emissions. The subsequent method was applied to two CRUK-funded trials (the international randomised sarcoma trial CASPS [ISRCTN63733470] and the UK cohort-based breast cancer trial PRIMETIME [ISRCTN41579286]). A guidance document defining the scope, method and assumptions was written to allow application to any publicly funded/investigator initiated clinical trial.

Results: Trial specific activities over and above routine care were grouped into 10 modules covering trial set up, conduct and closure. We identified emission factors for all trial activities within both trials and used them to estimate their total carbon footprint. The carbon footprint of CASPS, an international phase 2 trial of an investigational medicinal product with 47 participants, was 72 tonnes CO_2e, largely attributable to clinical trials unit emissions and staff travel. PRIMETIME, a UK-based phase 3 non-investigational medicinal product trial with 1962 patients, produced 89 tonnes CO_2e, largely attributable to trial-specific in-person participant assessments.

Conclusion: We have developed a method and guidance that trialists can use to determine the carbon footprint of clinical trials. The guidance can be used to identify carbon hotspots where alternative approaches to trial design and conduct could reduce a trial footprint, and where methodology research is required to investigate the potential impact of interventions taken to reduce carbon emissions. We will continue to refine the guidance to increase the potential application and improve usability.

STRENGTHS AND LIMITATIONS

- This is the first published and publicly available method and guidance to carbon footprint a clinical trial that can be used to inform lower carbon trial design.

For peer review only

- The guidance has been developed by an extensive and broad reaching collaboration and is intended for use by trialists with no prior experience of carbon footprinting.
- The method and guidance accounts for the carbon footprint of a clinical trial but no other metrics that may be important to consider in a full life cycle analysis.
- The emission factors used are the most appropriate publicly available factors that we could identify. More suitable or up to date emission factors may be available in the future or via paid subscription.
- The validity of results generated by the guidance is dependent on the quality and completeness of the activity data collated and the emission factors used.

INTRODUCTION

The World Health Organization has called climate change "the single biggest health threat facing humanity today" [1]. Whilst clinical trials are critical to identifying effective and safe treatments, in line with all healthcare activities, they also have a significant environmental impact. This contribution was first recognised around 16 years ago, when Ian Roberts and other members of the Sustainable Trials Study Group concluded that *"clinical trials contribute substantially to greenhouse gas emissions…. Notably through energy use in research premises and air travel"* [2]. Another study conducted by Lyle et al in 2009 of 12 UK pragmatic randomised trials, involving an average of 402 participants, showed that the average carbon emissions generated by the trials was 78.4 tonnes [3]. Multiplying this total by the 350,000 national and international trials registered on ClinicalTrials.gov would estimate the emissions of all global trials to be about 27·5 million tonnes of carbon dioxide equivalent [4]. This total, which is likely a highly conservative estimate, is comparable to the 25 million tonne CO_2e total footprint of the UK National Health Service which accounts for 6% of the UKs total footprint [5, 6]. More recently, Mackillop et al published a study quantifying the carbon footprint of three industry-sponsored late-stage cardiovascular, oncology and respiratory clinical trials [7]. The

For peer review only

total carbon footprint of the trials was 2498 tonnes CO_2e, 1632 tonnes CO_2e and 1437 tonnes CO_2e respectively.

However, with the exception of the aforementioned industry-sponsored study by Mackillop et al, little seems to have happened to quantify and consciously reduce the carbon consumption of clinical trials, but the urgency of the threat from climate change has increased exponentially.

It is not currently possible for trialists to easily estimate the environmental impact of running a clinical trial which limits their ability to contribute to climate change mitigation. However, there is recognition of the requirement to reduce the carbon footprint of clinical trials and practical guidance available encouraging this [8].

The Sustainable Healthcare Coalition (SHC) brought together the Low Carbon Clinical Trials (LCCT) Working Group including members of the MRC-NIHR Trials Methodology Research Partnership, UK and Ireland CRC trialists (led by the ICR-CTSU and the Liverpool Clinical Trials Centre), clinicians and others, to set out a strategy to reduce the carbon footprint of clinical trials [**Error! Bookmark not defined.**]. The first step to reducing the carbon footprint of a planned clinical trial is to reliably measure its potential footprint and identify the carbon hotspots. Subsequently, trialists will be able to interrogate alternative trial design approaches that may reduce the carbon footprint without impacting data quality, integrity, and validity.

Through an NIHR funded project [award ID: NIHR135419], we have developed a method and associated guidance that can be used by trialists to carbon footprint a clinical trial to inform future lower carbon trial design [9]. The aim is for the guidance to be applied prospectively during the design phase of a trial before trial funding is secured. In this paper we explain how the project team developed the guidance; illustrate initial results from its application on two CRUK-funded trials managed by ICR-CTSU; and describe the ongoing work to refine and expand the guidance.

METHODS

For peer review only

The approach to estimating the carbon impact of a publicly funded and/or investigator initiated clinical trial comprised the following stages:

1) The development of a process map to capture all activities of a clinical trial;
2) The development of a method to quantify the carbon footprint of all trial activities conducted specifically for the trial and which are over and above the provision of routine care, as defined in the process map;
3) Testing of the methodology with selected clinical trials to identify activity carbon hotspots and opportunities for mitigation.

Process mapping

In order to calculate the carbon footprint of any product, process or service, the whole system must be understood and established. To do this, information was extracted from available sources such as the ICR-CTSU Quality Management System and the NIHR Clinical Trials Toolkit. In addition, a series of information gathering meetings were held with participating Clinical Trials Units (CTUs) (see Table 1) to understand their trial portfolios and identify clinical trials and activities not yet represented in the process map. Discussions resulted in refinement of the process map and identification of a trial within each CTU to footprint which would result in the inclusion of the widest variety of trial types and activities in the test phase. The output of this stage was a visual representation of a clinical trial and a list of activities considered to be core clinical trial activities, as shown in **Figure 1.**

Guidance development

The core trial activities were grouped into the following modules: Trial set up; CTU emissions (includes energy and heating used in research premises, trial staff commuting and statistical analysis); Trial specific meetings and travel; Treatment intervention; Data collection and exchange; Trial supplies and equipment; Trial specific patient assessments; Samples; Laboratory; and Trial close out.

For peer review only

The carbon footprint of a trial was calculated by multiplying activity data by emission factors. Only activities undertaken to answer the research question, over and above routine care, were included. The main emission factor sources used were Ecoinvent version 2.2, GOV.UK GHG conversion factors, and the SHC care pathway carbon calculator [10, 11]. Where factors were not available in these publicly available sources, peer-reviewed, published papers, articles and product specifications (e.g. for pathology tests and electronic devices) were used. The most applicable and up to date factors that are freely available for public use were selected, and all sources cited and referenced. It is important to note that in some cases more up to date factors, or forecasted emission factors may be available, however we wanted to ensure that the guidance developed could be used without the need for purchasing licenses for those various emission factors, which would be a barrier for publicly funded trialists.

Two documents were produced, a "Detailed Guidance and method to calculate the carbon footprint of a clinical trial" and a "Data collation quick guide and worksheet". The detailed guidance defines the project scope, limitations, assumptions, emission factors and benchmark data sources, as well as detailed breakdowns of the calculations required to calculate the carbon footprint of each activity. The "Data collation quick guide and worksheet" guides users in the application of the detailed guidance by summarising the calculations in their simplest form and displaying the activity data the user is required to collect in order to complete the required calculations. See **Appendices A and B** for the current version of the detailed guidance and worksheet respectively.

Applying the guidance – case studies

Although we intend for the guidance to be used prospectively at the trial design stage, in order to test the method and develop a full and comprehensive set of calculations, this guidance was retrospectively and iteratively applied to two ICR-CTSU managed, CRUK-funded trials, CASPS and PRIMETIME. CASPS is an international phase II trial of cedirinib in the treatment of patients with alveolar soft part sarcoma. The trial enrolled 47 patients across twelve sites in the UK, Spain, and

For peer review only

Australia and involved an internationally shipped IMP, completion and shipment of paper CRF, on site visits by the trial team for site initiation, monitoring visits and audit, additional hospital visits for patients for trial-specific assessments and provision of tissue samples. PRIMETIME is a post-operative avoidance of radiotherapy trial which recruited 1962 patients across 64 sites in the UK. The trial used electronic data capture for collection of data, data linkage with NHS routine data sources and involved shipment of tissue samples for immunohistochemistry testing and subsequent long-term sample storage.

These two trials were selected because of their contrasting designs (International CTIMP vs national non-CTIMP), the trials were complete (meaning we could test the method on the entirety of a trial), and activity information was readily available.

Having identified the trial activities using the process map and guidance, activity data was gathered from the trial protocol, Site Investigator File, funding application, trial specific databases, systems and trackers, and through discussion with the trial teams. Once collated, the trial activity data was multiplied by the emission factors identified in the guidance to calculate a carbon footprint. Application to real life, complete trials resulted in iteration of the guidance and the addition of activities not previously identified, as well as their correlating emission factors e.g. data linkage and copying images from trial-specific scans to CDs. The full calculations for CASPS and PRIMETIME can be found in appendices C and D respectively.

Patient and Public Involvement

Patients and the public were not involved in this stage of the research to develop a method and guidance to carbon footprint a clinical trial. However, it is critical that patient partners are involved in this work now that a method is available, to seek their views on suggestions and recommendations to reduce the carbon footprint of clinical trials without reducing acceptability to participants.

RESULTS

For peer review only

The guidance has been developed and used to calculate the carbon footprint of two clinical trials (results were presented at ICTMC in Oct 2022).

CASPS TRIAL

Applying the guidance, the carbon footprint of CASPS was estimated to be 72 tonnes (71,939 kgCO_2e). The carbon footprint of a typical UK citizen is 12.7 tonnes CO_2e per year, meaning CASPS has a footprint equivalent to around 6 UK citizens [12]. As shown below in **Figure 2**, the largest contribution to the footprint was CTU emissions (23,488 kgCO_2e), followed by trial specific meetings and travel (20,465 kgCO_2e), largely due to the sites in Australia which required internal flights for site visits. Despite the analysis of samples for a sub-study being out of scope, central laboratory activities still contributed significantly to the footprint due to storage of samples in -80 degree freezers for up to 10 years.

PRIMETIME TRIAL

The total carbon footprint of PRIMETIME was calculated to be 89 tonnes (89,360 kgCO_2e). This is equivalent to 7 times the annual carbon footprint of a UK citizen.

Figure 3 demonstrates that the largest contribution to the PRIMETIME carbon footprint was patient travel and assessments (60,823 kgCO_2e), followed by CTU emissions (11,733 kgCO_2e) and data collection and exchange (7572 kgCO_2e).

The PRIMETIME trial consisted of two treatment cohorts. In the standard of care cohort, the intervention comprised standard of care radiotherapy and annual follow up mammograms were undertaken for 5 years to assess disease outcome. In the other group, no radiotherapy was given and patients received annual follow up mammograms for 10 years to assess disease outcome. The additional 5 scans and associated patient travel were included in the PRIMETIME carbon footprint.

Further testing

For peer review only

The current version of the detailed guidance is undergoing further iterative testing on a selection of trials from 8 participating CTUs as described below in **Table 1**. Through its wider application, we hope to capture all possible clinical trial activities and emission factors across a wide range of trial designs. Although the calculations required to carbon footprint the case studies in their entirety were extensive, through further testing and refinement of the guidance, we hope to identify hotspots so that future iterations of the guidance can be streamlined to enable data collection to be less resource-intensive.

The project team are also collecting qualitative data on the use and application of the guidance document. This will include how academic trialists managed this work alongside day-to-day trial management; the staff resource and time required to complete the calculations; any barriers to carrying out this work; how user friendly the guidance was and how much technical knowledge and support were required to complete the calculations.

Table 1. Participating CTUs testing the guidance and trials undergoing footprinting

CTU	Trial Name	Description
**Cardiff Centre for Trials Research**	The UK stand together trial	A two-arm pragmatic multicentre cluster randomised controlled trial which aims to evaluate the effectiveness and cost-effectiveness of KiVa, a school-based anti-bullying programme, in reducing bullying in schools compared to usual practice. 116 primary schools participated from four areas; North Wales, West Midlands, South East and South West England [13].
**Edinburgh Clinical Trials Unit**	RESTART	A prospective, open, blinded end point, parallel group randomized clinical trial that compared the effects of starting vs avoiding antiplatelet therapy after ICH. The trial recruited 537 participants at 122 hospitals in the UK [14].

For peer review only

**Imperial Clinical Trials Unit**	ON-PACE	On-PACE is a double-blind randomised trial investigating whether taking a nutritional supplement is beneficial for people with the most severe form of chronic obstructive pulmonary disease (COPD). It will recruit 96 people with COPD who use oxygen at home to take part in a 3 month long clinical trial [15].
**Liverpool Clinical Trials Centre**	HEAL-COVID	HElping Alleviate the Longer-term Consequences of COVID-19 (HEAL-COVID), an adaptive platform trial, aims to evaluate the impact of treatments on longer-term morbidity, mortality, re-hospitalisation, symptom burden and quality of life associated with COVID-19. The trial took place across 109 sites and randomised 1245 participants [16].
**MRC Clinical Trials Unit at UCL**	MAVMET	A multicentre, 48 week randomised controlled factorial trial of adding maraviroc and/or metformin for hepatic steatosis in HIV-1-infected adults on combination antiretroviral therapy. The trial took place at 6 sites across the UK and recruited 90 participants [17].
**Newcastle Clinical Trials Unit**	PREMISE	A multi-arm, multi-centre, non-inferiority randomised controlled trial comparing 3 minimally invasive treatments to the current gold standard operation for bladder obstruction due to enlarged prostate in the National Health Service. The planned sample size is 536 [18].
**The George Institute**	INTERACT3	An international, multicentre, prospective, stepped wedge, cluster randomised, blinded outcome assessed, controlled trial of a care bundle of physiological control strategies in acute intracerebral haemorrhage. The trial recruited 7064 patients from 122 hospitals in

For peer review only

		10 countries (Chile, Brazil, China, India, Mexico, Nigeria, Pakistan, Peru, Sri Lanka, and Vietnam) [19].
**University of Galway**	EMERGE	A randomised placebo-controlled trial of the Effectiveness of MEtformin in addition to usual care in the Reduction of GEstational diabetes mellitus effects. Planned sample size is 550 [20].

DISCUSSION

We have developed guidance that trialists could use to carbon footprint clinical trials and subsequently inform lower carbon trial design and delivery.

The detailed guidance document, enclosed as an appendix, describes a method that can be used to calculate the carbon footprint of publicly funded/investigator initiated, CTIMP and Non-CTIMP (inc complex intervention) trials conducted in the NHS. Although the governance, funding and intent of commercially sponsored clinical trials can be different, the individual trial activities are comparable with publicly funded/investigator initiated research and as such this method could be used to footprint the activities within any clinical trial. However, the scope and assumptions defined in the guidance are tailored to academic, publicly funded clinical trials. The guidance is currently undergoing testing on a wider selection of trials managed by collaborating UK CRC Registered and international CTUs. The project team plan to publish the results of all additional trials footprinted as part of the subsequent testing phase and updates to the guidance this will inevitably generate, on completion of the currently funded project. The collaboration is open to, and welcomes new partners. Collaborators can receive support in using and applying the guidance as well as help sourcing new emission factors through webinars and drop in clinics facilitated by the MRC-NIHR TMRP Greener Trials Group. The Group aims to provide a forum for the development and dissemination of greener research practice.

For peer review only

It is important to note that this guidance accounts only for the carbon footprint of a clinical trial. It does not include other metrics that are important to consider in a full life cycle analysis when evaluating environmental impact and potential trade-offs, including water use, land use, waste and those relating to social and economic impacts. In addition, activities performed by the manufacturers of interventions used in the trial and activities performed by regulators to review and approve clinical trials have been excluded from the scope of the guidance, as these are not activities that trialists working on publicly funded and/or investigator initiated clinical trials can influence or control.

It is necessary to note that the validity of the carbon footprinting results the guidance generates is highly dependent on the quality and completeness of the activity data collated and the specific emission factors used. We recognise that more suitable or up to date emission factors than those provided may be available or will become available as knowledge around the subject grows, but these could not be included as they are not publicly or freely available.

A number of assumptions are defined in the guidance to ensure consistency of application to different clinical trials and a degree of pragmatism applied, such as using average occupancies for members of hospital staff. All assumptions made are described in full in the guidance and can be interrogated in the real-life example calculations included in appendices C and D.

Previous research clearly demonstrated that clinical trials have an impact and where the carbon hotspots might be (trial team commuting, energy use in research premises, trial travel and freight). It is difficult to compare the results of the case studies to those of the CRASH-1 and CRASH-2 carbon audits as they were large international trials which enrolled 10,008 and 20,211 patients respectively, and emissions were calculated over a single, one-year period. We also grouped activities in different ways; in the CRASH audit the footprint of all deliveries was presented together, whereas here deliveries were calculated within their specific module, for example delivery of SIFs was included with 'trial set up' activities. Trial coordination centre emissions were the largest and second largest contributors in the CRASH trials, similarly in the case study results presented here, 'CTU emissions'

For peer review only

was the largest contributor to the CASPS footprint and the second largest in PRIMETIME. Trial team commuting and energy use in research premises, which were calculated together in the current guidance under ‘CTU emissions’, also accounted for the most CO_2e emissions in the 2009 study conducted by Lyle et al. This study calculated the average emissions of 12 UK trials to be 78.4 tonnes, comparable to the 72 and 89 tonne carbon footprints of CASPS and PRIMETIME. Patient travel was not calculated in the CRASH trials and was only the 4th largest contributor to emissions in the Lyle study. Conversely, in the case study results presented here ‘trial specific patient assessments’ was the largest contributor to PRIMETIME emissions and included both patient travel and procedures.

The results presented are in agreement with previously identified hotspots, however the case studies have also identified different and new hotspots, such as data collection and exchange and laboratory associated activities. The results also show that the % contribution by activity varied across the two case studies, demonstrating the need to collect data from more trial types and designs to capture all possible hotspots.

As well as refinement and enhancement of the guidance to ensure it is user-friendly and can be applied to as many clinical trial types as possible, more trials must be footprinted to understand how to define the scope of what is considered a clinical trial and how best to apply the guidance.

The carbon footprint associated with the manufacture and delivery of the trial intervention are out of scope in the current guidance. However, when implementing the results of a clinical trial it will also be important to consider the footprint of the recommended intervention. This latter point is exemplified by the PRIMETIME trial, where delivery of the trial has a notable carbon footprint. However, if the trial results recommend the avoidance of radiotherapy for some breast cancer patients, there would be a significant carbon saving because standard of care radiotherapy and associated patient travel would no longer be required (the carbon footprint associated with delivering 15 doses of standard of care radiotherapy is 94.19 kgCO_2e per patient, consisting of 7.19 kg CO_2e for the radiotherapy and 87 kgCO_2e for the travel required) [21, 9]. Further work is required to develop

For peer review only

guidance on the interpretation of carbon footprinting results, so that the impact of conducting a clinical trial can be balanced against the improvement an intervention may make both in terms of patient outcomes and the carbon footprint of the intervention once incorporated into standard of care.

The results of the future work with collaborating CTUs summarised in Table 1 will be published in due course and will include discussion and comparison of the hotspots, as well as evaluation of the inter-observer reliability and reproducibility. We will also look at the time commitment required to complete the calculations and how the guidance can be improved for use by those without specialist carbon footprinting knowledge. In the case studies the guidance was applied retrospectively to completed trials, however, ultimately the aim is to use the guidance prospectively at the trial design stage. We anticipate application in this scenario will be less time and resource intensive, but this will be analysed and discussed in the next paper, as we plan to include trials which are currently in development. Our findings will contribute to ongoing work coordinated by the Sustainable Healthcare Coalition to develop an online, open-access carbon footprint calculator in collaboration with a group of pharmaceutical industry representatives conducting similar work. Through this collaboration, the generalisability of a common method and the scope of its application to academia and industry will be examined.

There remains much to do to help shape the future of sustainable clinical trials and support the move towards more responsible research. Investigation of carbon trade off decisions with patient and public involvement is necessary to enable recommendations to be made to reduce the carbon footprint of clinical trials, without reducing the clinical value of the trial and acceptability to participating patients and work with patient partners to shape the next steps of this important research is planned. Dissemination and engagement activities, including training and practical support for research teams incorporating carbon calculations into routine trial design, will be required to help drive the paradigm shift to responsible greener research.

For peer review only

As we gather more data on the estimated carbon footprint of planned clinical trials, further consideration should be given to how various stakeholders are going to use and consider the information generated. Funding panels often refer to cost per patient, a concept proposed for this area in terms of the carbon emission per patient. Care would be needed to interpret this statistic; as is the case with the cost per patient, carbon footprint per patient may discriminate against rare disease trials with a high health burden, as it does not include information on the nature and consequences of the condition under study. Further consideration is also required to determine a meaningful way to present the carbon footprint information and provide context to the public. A suggestion has been made that graphical representation in terms of the equivalent number of UK citizens' footprints could be more easily understood, but further research is needed.

Ultimately, a method and associated guidance describing how to carbon footprint a clinical trial will support various organisations including universities, health service providers and research funders to meet their carbon reduction commitments and allow stakeholders to incentivise a reduction of the carbon footprint of trials in their portfolios. We have produced this guidance as the first step of a strategy to help towards achieving this aim.

Figure legends

Figure 1. A process map displaying the core activities of a clinical trial considered within the system boundary of the carbon footprinting calculations.

Figure 2. A breakdown of the contributions to CASPS carbon footprint

Figure 3. A breakdown of the contributions to PRIMETIMEs carbon footprint

Acknowledgements

This project is funded by the National Institute for Health Research (NIHR) CTU Support Funding scheme [Award ID: NIHR135419]. The views expressed are those of the author(s) and not necessarily those of the NIHR or the Department of Health and Social Care.

For peer review only

Grateful thanks to Environmental Resources Management for their technical support and expertise in the development of the method and sourcing some emission factors.

Competing Interests

JG, LF, JB, PW and CG have received NIHR funding to undertake this study (support for the manuscript). FA is the paid chair of the Sustainable Healthcare Coalition. KM is a member of the Sustainable Healthcare Coalition. RA-SS reports an NIHR Health Technology Assessment trial grant (CARE pilot trial) and an NIHR Efficacy and Mechanism Evaluation (EME) Programme Application Acceleration Award – International platform studies in precision medicine (CARE PREP) and consulting fees from Recursion Pharmaceuticals, McMaster University Population Health Research Institute, and Bioxodes, all paid to The University of Edinburgh. MRS reports grants from Astellas, Clovis Oncology, Janssen, Novartis, Pfizer, and Sanofi-Aventis; consulting fees from Eli Lilly; speaker's fees from Lilly Oncology, Janssen and payment from Eisai, all unrelated to the topic of this Comment. SP reports grants/contracts from EDCTP; MRC; Gilead Sciences; Janssen-Cilag; ViiV Healthcare; NIHR; University of Minnesota and is a member of the TIPAL DSMB. PD reports grants from Health Research Board of Ireland and Enterprise Ireland for Peer Reviewed research funding. CSA is the Vice President of the World Stroke Organisation and is Editor-in-chief of Cerebrovascular Diseases, and holds Research grants from National Health and Medical Research Council (NHMRC) of Australia, Medical Research Council (MRC) of the UK, Takeda and Penumbra (paid to the George Institute). EB, EM, CM, AMP, AB, DC, XC, KH, NM report no competing interests.

Contributorship statement

JG made substantial contributions to the conception and design of the work, acquired, analysed and interpreted date for the work, drafted the work, reviewed it critically for important intellectual content, gave final approval of the version to be published, ensured questions related to the accuracy or integrity of any part of the work were appropriately investigated and resolved.

LF made substantial contributions to the conception and design of the work, acquired, analysed and interpreted date for the work, drafted the work, reviewed it critically for important intellectual content, gave final approval of the version to be published, ensured questions related to the accuracy or integrity of any part of the work were appropriately investigated and resolved.

PW made substantial contributions to the conception and design of the work, acquired, analysed and interpreted date for the work, drafted the work, reviewed it critically for important intellectual content, gave final approval of the version to be published, ensured questions related to the accuracy or integrity of any part of the work were appropriately investigated and resolved.

The Low Carbon Clinical Trials group made substantial contributions to the conception and design of the work and/or acquired, analysed and interpreted date for the work, reviewed the work critically for important intellectual content, gave final approval of the version to be published and ensured questions related to the accuracy or integrity of any part of the work were appropriately investigated and resolved.

Funding

This project is funded by the National Institute for Health Research (NIHR) CTU Support Funding scheme [Award ID: NIHR135419].

For peer review only

Data sharing statement

All data relevant to the study are included in the article or uploaded as supplementary information.

Ethics approval statement

This work did not require ethics approval.

References

[1] Climate change and health [Internet]. World Health Organization. 2021 October 30 [cited 2023 May 11]. Available from: https://www.who.int/news-room/fact-sheets/detail/climate-change-and-health
[2] Sustainable Trials Study Group. Towards sustainable clinical trials. *BMJ* 2007;334:671
[3] Lyle K, Dent L, Bailey S, et al. Carbon cost of pragmatic randomised controlled trials: retrospective analysis of sample of trials. *BMJ* 2009;339:b4187
[4] Adshead F, Al-Shahi Salman R, Aumonier S, Collins M, Hood K, McNamara C, Moore K, Smith R, Sydes MR, Williamson PR (2021) A strategy to reduce the carbon footprint of clinical trials. Lancet. 2021 Jul 24;398(10297):281-282. doi: 10.1016/S0140-6736(21)01384-2. 34171294.
[5] Tennison I, Roschnik S, Ashby B, Boyd R, Hamilton I, Oreszczyn T, et al. Health care's response to climate change: a carbon footprint assessment of the NHS in England. The Lancet Planetary Health [Internet]. 2021 Feb 1;5(2):e84–92. Available from: https://www.thelancet.com/journals/lanplh/article/PIIS2542-5196(20)30271-0/fulltext
[6] The NHS' "first climate friendly" operation [Internet]. BBC News. 2022 May 25 [cited 2023 May 12]. Available from: https://www.bbc.co.uk/news/uk-england-birmingham-61567187
[7] Mackillop N, Shah J, Collins M*, et al* Carbon footprint of industry-sponsored late-stage clinical trials *BMJ Open* 2023;**13:**e072491. doi: 10.1136/bmjopen-2023-072491
[8] NIHR Carbon Reduction Guidelines [Internet]. NIHR. 2019 July 30 [cited 2023 May 11]. Available from: https://www.nihr.ac.uk/documents/the-nihr-carbon-reduction-guidelines/21685
[9] Development and prototype testing of a method to quantify the carbon footprint of current clinical trials to inform future lower carbon trial design [Internet]. NIHR funding and awards. [cited 2023 May 11]. Available from: https://fundingawards.nihr.ac.uk/award/NIHR135419
[10] Greenhouse gas reporting: conversion factors 2022 [Internet]. Gov.uk. 2022 [cited 2023 May 11]. Available from: https://www.gov.uk/government/publications/greenhouse-gas-reporting-conversion-factors-2022
[11] Care pathways carbon footprint calculator [Internet]. Sustainable Healthcare Coalition. [cited 2023 May 11]. Available from: https://shcpathways.org/full-calculator/
[12] Kayser B. What is the average carbon footprint per person in the UK? | Pawprint- Your Eco Companion [Internet]. 2020. [cited 2023 May 11]. Available from: https://www.pawprint.eco/eco-blog/average-carbon-footprint-uk
[13] Clarkson, S., Bowes, L., Coulman, E. *et al.* The UK stand together trial: protocol for a multicentre cluster randomised controlled trial to evaluate the effectiveness and cost-effectiveness of KiVa to reduce bullying in primary schools. *BMC Public Health* **22**, 608 (2022). https://doi.org/10.1186/s12889-022-12642-x
[14] REstart or STop Antithrombotics Randomised Trial (RESTART) [Internet]. The University of Edinburgh. 2021 December 1 [cited 2023 May 11]. Available from: https://www.ed.ac.uk/clinical-brain-sciences/research/completed-studies-trials/restart-or-stop-antithrombotics-randomised-trial
[15] Improving the experience of physical activity in people with severe lung disease using dietary nitrate supplementation with beetroot juice [Internet]. ISRCTN. [cited 2023 May 11]. Available from: https://www.isrctn.com/ISRCTN12474100
[16] HElping Alleviate the Longer-term Consequences of COVID-19 (HEAL-COVID) [Internet]. ClinicalTrials.gov. 2021 March 17 [cited 2023 May 11]. Available from: https://clinicaltrials.gov/ct2/show/NCT04801940
[17] MAVMET [Internet]. MRC Clinical Trials Unit at UCL. [cited 2023 May 12]. Available from: https://www.mrcctu.ucl.ac.uk/studies/all-studies/m/mavmet/
[18] PREMISE [Internet]. Newcastle Clinical Trials Unit. Newcastle University. [cited 2023 May 11]. Available from: https://www.ncl.ac.uk/nctu/trials/therapeutic-areas/renal/premise/

For peer review only

[19] The Third, Intensive Care Bundle With Blood Pressure Reduction in Acute Cerebral Hemorrhage Trial (INTERACT3) [Internet]. ClinicalTrials.gov. 2017 July 6 [cited 2023 May 11]. Available from: https://clinicaltrials.gov/ct2/show/NCT03209258
[20] Dunne F, Newman C, Devane D, Smyth A, Alvarez-Iglesias A, Gillespie P, Browne M, O'Donnell M. A randomised placebo-controlled trial of the effectiveness of early metformin in addition to usual care in the reduction of gestational diabetes mellitus effects (EMERGE): study protocol. Trials. 2022 Sep 21;23(1):795. doi: 10.1186/s13063-022-06694-y. PMID: 36131291; PMCID: PMC9494837.
[21] Shenker, RF., Johnson, TL., Ribeiro, M. *et al*. Estimating Carbon Dioxide Emissions and Direct Power Consumption of Linear Accelerator–Based External Beam Radiation Therapy. *Advances in Radiation Oncology* (2023) 8, 101170. https://doi.org/10.1016/j.adro.2022.101170

Appendices

Appendix A: Detailed guidance

Appendix B: Quick guide

Appendix C: CASPS calculations

Appendix D: PRIMETIME calculations

For peer review only

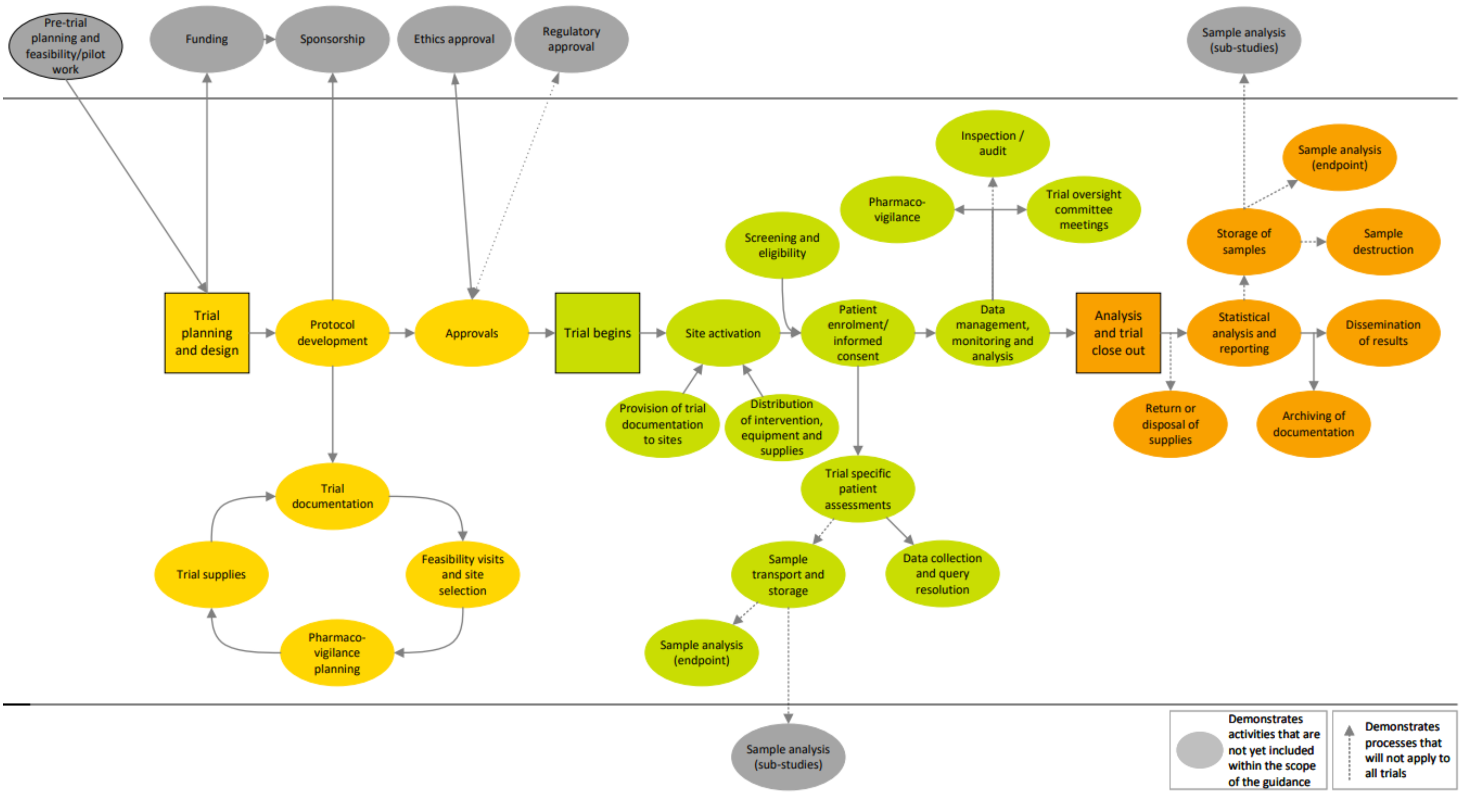

For peer review only

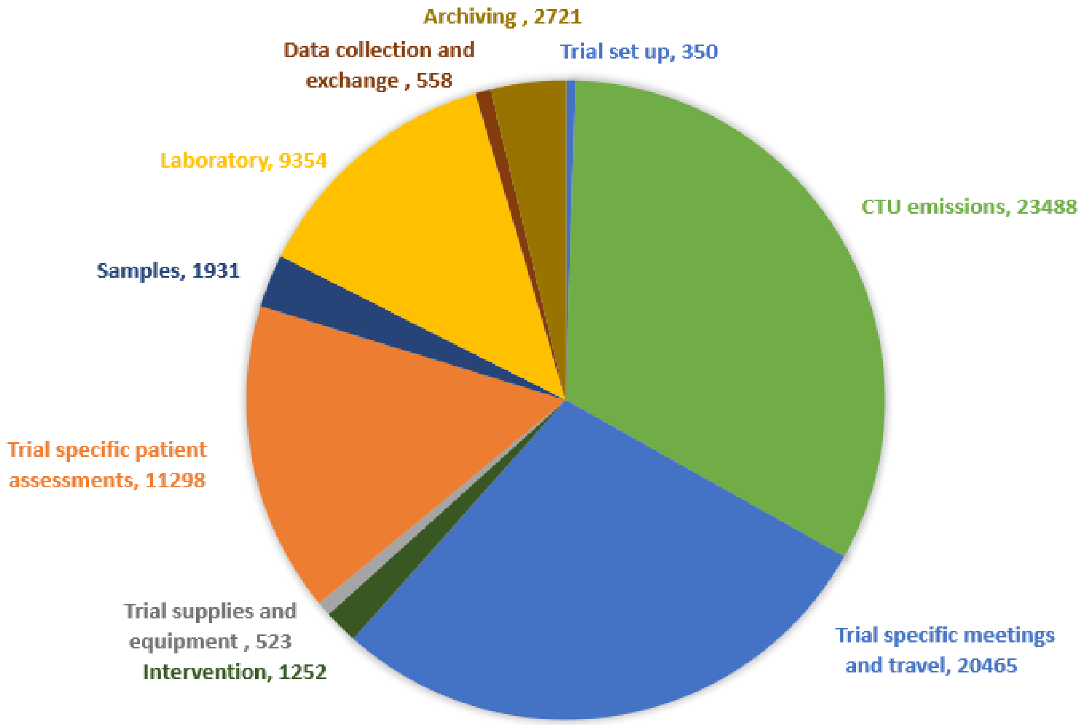

CONTRIBUTION TO CASPS CARBON FOOTPRINT (kg CO_2e)

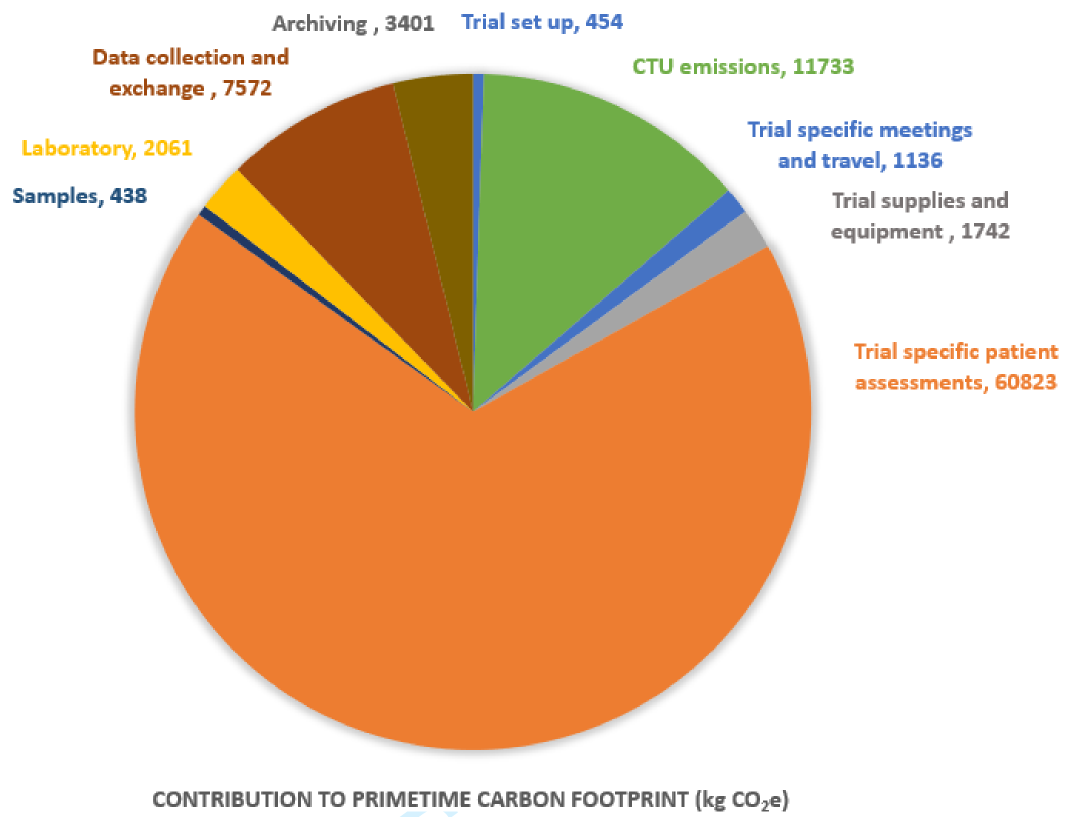
Archiving , 3401
Trial set up, 454
Data collection and exchange , 7572
CTU emissions, 11733
Trial specific meetings and travel, 1136
Laboratory, 2061
Samples, 438
Trial supplies and equipment , 1742
Trial specific patient assessments, 60823
CONTRIBUTION TO PRIMETIME CARBON FOOTPRINT (kg CO_2e)

FUNDED BY NIHR | National Institute for Health and Care Research

Enabling lower carbon clinical trials: Development and prototype testing of a method to quantify the carbon footprint of clinical trials to inform future lower carbon clinical trial design

Detailed Guidance and method to calculate the carbon footprint of a clinical trial

Background

Around 15 years ago, the Sustainable Trials Study Group concluded that clinical trials contribute substantially to greenhouse gas emissions, notably through energy use in research premises and air travel[1].

In addition, a study conducted in 2009 of 12 UK pragmatic randomised trials involving an average of 402 participants showed that the average carbon emission generated by the trials was 78.4 tonnes[2]. Multiplying this total by the 350,000 national and international trials registered on ClinicalTrials.gov, this would estimate that emissions attributable to all global clinical trials to be about 27·5 million tonnes of carbon dioxide equivalent[3].

Since then, the urgency of the threat from the climate crisis has increased exponentially and the World Health Organization calls climate change the single biggest health threat facing humanity[4]. Planned climate action is not sufficient to prevent the current warming predictions; humanity must limit warming to 1.5 degrees and reach net-zero by 2040 to avoid the worst consequences of climate change.

As a first step to reduce the environmental impact of clinical trials, a method to quantify the carbon footprint of a clinical trial and associated processes is required.

Introduction

This guidance provides information on how to carbon footprint a clinical trial for the purposes of the NIHR-funded project ‘enabling lower carbon clinical trials.’

Within the guidance, clinical trial processes have been sub-divided into the following modules:

1. Trial set up
2. CTU emissions
3. Trial specific meetings and travel
4. Treatment intervention
5. Data collection and exchange
6. Trial Supplies and equipment
7. Trials specific patient assessments
8. Samples
9. Laboratory
10. Trial close out

The above list is not exhaustive, and it is expected that further activities and modules may need to be added to account for specialist processes in all clinical trial types and as knowledge around life cycle analysis increases.

NB: analysis of data does not need to be calculated separately, it is covered by the emissions attributed to trial staff FTE in “CTU emissions” and calculations included within “Data Collection and exchange”.

For peer review only - http://bmjopen.bmj.com/site/about/guidelines.xhtml

Calculating carbon footprint

A carbon footprint is a measure of greenhouse gases, usually quoted in kg or tonnes of carbon dioxide equivalent (CO_2e). To calculate the carbon footprint of a particular clinical trial process, both 'activity data' and 'emission factors' are required.

An 'activity' could be anything from electricity consumption to materials, travel and food. Activity data quantifies the amount of that activity e.g. distance travelled, kWh used etc.

An emission factor, also known as a conversion factor, "is a coefficient which allows you to convert the activity data into greenhouse gas emissions. It is the average emission rate of a given source, relative to units of activity or process/processes."[5]

To calculate a carbon footprint of a trial, the activity data will need to be provided by the trial management team and multiplied by the emission factors provided in this guidance document. Two types of activity data may be used:

- Primary data: data collected first-hand from specific activities within the studied clinical trial process i.e., the data collected where you can determine the amount of the activity taking place, for example electricity in kWh used by a building or the weight of an IMP shipment and the distance it travels.

- Secondary data: activity data that is not collected from specific activities within the studied clinical trial because you cannot determine or measure the exact quantity of the activity taking place, for example the number of m^2 occupied by an office worker or hospital worker. Secondary data may take the form of average, or typical, information about an activity from a published study or other source and will be provided in this guidance document e.g., average m^2 occupied by an office worker or average distance travelled.

Primary activity data are preferred for all activity data used in each module. However, secondary data may be used where primary activity data is unavailable or difficult to obtain.

It is important to avoid double-counting activities i.e., modules must not include activities already covered by other modules in the clinical trial process map. A data collection tool is provided alongside this guidance to aid in this process and help avoid double-counting.

Scope

This guidance describes a method to calculate the carbon footprint of a UK, academic clinical trial.

It can be applied to trials with international participation, however as emission factors vary between countries, and those provided within this guidance are UK-specific, country-specific emission factors may be required. Proxy emission factors which have been approved for use can be used where required and the source country of an emission factor will be stated where applicable.

The tool is intended to calculate the carbon footprint of the processes specific to the clinical trial, defined as the data required to analyse the trial endpoints and the research activities over and above standard of care.

The guidance is intended for use as a tool to inform sustainable decision-making in the design of clinical trials, rather than a tool to calculate the absolute footprint of a clinical trial or compare environmental performance of one trial over another.

For peer review only - http://bmjopen.bmj.com/site/about/guidelines.xhtml

FUNDED BY NIHR | National Institute for Health and Care Research

LIVERPOOL CLINICAL TRIALS CENTRE LCTC

ICR

Limitations

There are a number of emission factors that can be used for a particular process and activity data can be calculated in a number of ways. Therefore, life cycle analysis produces variation in its results, dependent on choices made by the individual performing the calculations.

We have endeavoured to include an explanation or justification for the choice of emission factors used. In addition, the emission factors have been selected as the most applicable and up to date factors that are freely available for public use. It is important to note that more up to date factors, or forecasted emission factors, may be available, but they are subject to licensing requirements and are not publicly available. The source of all factors used is included for reference.

This guidance accounts only for the greenhouse gas emissions. It does not include other metrics that are also important to consider when evaluating sustainability and the potential trade-offs, for example water use, land use, waste and those relating to social and economic impacts.

Assumptions

- The eventual aim of this tool is to be used prospectively during the design phase of a trial, before trial funding is secured. However, the tool will not capture the carbon footprint associated with work conducted during this period i.e., prior to confirmation of funding award. The tool can also be used retrospectively on clinical trials which are complete.
- The tool only calculates emissions of processes which have been funded and defined within the trial protocol i.e., future planned work which has not yet been funded or that will be defined outside of the protocol are not included.
- The tool only calculates the emissions of patient participation where it exceeds or is additional to routine care, where it is required to establish an endpoint, the patient population or eligibility. Use of the trial SOECAT or the costing included in the initial funding application (if the trial predates use of SOECAT) is encouraged, to consistently define the investigations considered in addition to routine care and/or part of the research question.
- For all translational/optional/research samples and sub studies, the tool does not calculate emissions associated with analysis performed by central laboratories or collaborators, but does calculate emissions for activities defined in the protocol, such as collecting the sample or data from patients, initial processing at participating sites and shipment of samples or data to the site of subsequent analysis.
- This guidance will only appraise trials with UK based trial management (sites may be international but the trial must be overseen by a UK Clinical Trials Unit/Sponsor/ Research team)
- The carbon footprint associated with the manufacture of a trial intervention e.g. IMPs and Medical Devices is not included in this guidance.
- The tool does not calculate the carbon footprint of waste associated with a particular clinical trial. Concerning clinical trial consumables, activity data is based on the quantities purchased. The only exception to this is the destruction of unused IMP at participating sites, as this an activity specifically undertaken for a clinical trial which has an associated carbon footprint.
- The tool does not calculate the carbon footprint of hospital and laboratory staff commuting.
- Carbon emissions generated by ethics and regulatory approval bodies are not within the scope of this guidance and will not be calculated.

NB: more module specific assumptions can be found throughout the document where applicable.

For peer review only - http://bmjopen.bmj.com/site/about/guidelines.xhtml

FUNDED BY NIHR | National Institute for Health and Care Research

LIVERPOOL CLINICAL TRIALS CENTRE LCTC

ICR

1. Trial set up

This module includes the following activities:

1.1 Production of trial documentation to be sent to sites or participants
1.2 Provision/postage of trial documentation to sites
1.3 Provision/postage of documentation to participants by CTU or participating sites
1.4 Provision/postage of incentives to participant

1.1. Production of trial documentation

For production of trial documentation, the carbon footprint of both the printing and materials must be calculated.

Printing: The number of pages must be multiplied by 0.005 to produce a weight in kilograms, and the weight multiplied by the relevant emission factor provided below.

- Black and white: kg (paper) x 0.22438 = kgCO_2e
- Colour: kg (paper) x 0.31786 = kgCO_2e

1292	use, printer, laser jet, b/w, per kg printed paper	electronics	services	kg	RER	0.22438
1293	use, printer, laser jet, b/w, printing per h	electronics	services	h	CH	0.37213
1294	use, printer, laser jet, b/w, printing per h	electronics	services	h	RER	0.39065
1295	use, printer, laser jet, colour, per kg printed paper	electronics	services	kg	CH	0.2027
1296	use, printer, laser jet, colour, per kg printed paper	electronics	services	kg	RER	0.31786

NB**:** If you are unable to calculate the number of pages, you may assume that there are 150 pages in a small ring binder and 500 pages in a large lever arch folder.

Assumption: 1 piece of paper weighs around 5g/0.005kg

Emission factor source: Ecoinvent, version 2.2, 2011 [6] (RER = European emission factor)

Materials (paper): For the carbon footprint of paper production**,** the number of pages must be multiplied by 0.005 to produce a weight, and the weight multiplied by the emission factor for paper manufacture provided below.

Kg (paper) x 0.919 = kg CO_2e

Paper and board: paper	Gov.UK	2021	kg						0.919

Emission factor source: Greenhouse gas reporting: conversion factors 2021, GOV.UK [7]

Materials (folders): As trial documentation is often sent to sites in folders, provided below are estimated weights of folders. The total weight in kg will then need be multiplied by the emission factor for cardboard (provided below).

53	Paper and board: board	Gov.UK	2021	kg						0.821

CF of the folder materials = Kg x 0.821 = kg CO_2e

Lever arch: Assumption: Weight of lever arch = 0.5kg

Ring binder: Assumption: Weight of ring binder = 0.3kg Eastlight A4 Black Ring Binder - EA54121 (staples.co.uk)

Emission factor source: Greenhouse gas reporting: conversion factors 2021, GOV.UK [7]

For peer review only - http://bmjopen.bmj.com/site/about/guidelines.xhtml

FUNDED BY NIHR | National Institute for Health and Care Research

LIVERPOOL CLINICAL TRIALS CENTRE LCTC

ICR

1.2, 1.3, 1.4 Provision of trial materials

For provision of trial materials to sites by post/courier you will need to calculate the total weight of the materials (in tonnes) and multiply this by the distance they travel (in km) to get tonne.km. The t.km is then multiplied by the emission factor for either road or air freight provided below.

Road freight:

Mass (tonnes) x distance (km) = t.km

t.km x emission factor = (kg CO_2e)

Emission factor for road freight = 0.19443

1942	2724	transport, lorry 20-28t, fleet average	transport systems	road	tkm	CH	0.19443

Emission factor source: Ecoinvent, version 2.2, 2011 [6]

Air freight:

Mass (tonnes) x distance (km) = t.km

t.km x most suitable emission factor from highlighted column = (kg CO_2e)

			With RF				Without RF			
Activity	Type	Unit	kg CO_2e	kg CO_2	kg CH_4	kg N_2O	kg CO_2e	kg CO_2	kg CH_4	kg N_2O
Freight flights	Domestic, to/from UK	tonne.km	4.49363	4.46949	0.00188	0.02226	2.3765	2.35236	0.00188	0.02226
	Short-haul, to/from UK	tonne.km	2.30229	2.2908	0.00008	0.01141	1.21717	1.20568	0.00008	0.01141
	Long-haul, to/from UK	tonne.km	1.0189	1.01381	0.00004	0.00505	0.53867	0.53358	0.00004	0.00505
	International, to/from non-UK	tonne.km	1.0189	1.01381	0.00004	0.00505	0.53867	0.53358	0.00004	0.00505

NB: 'With RF' values are recommended. RF (combustion and radiative forcing) includes the indirect and direct emissions.

NB: Always use values relating to 'kgCO_2e'

For air freight you need to add the WTT (well-to-tank) value to get the final total for freight flight. WTT can be calculated by multiplying the t.km used in the first calculation by the correlating WTT conversion factor provided below.

Tonne.km x correlating WTT conversion factor

			With RF	Without RF
Activity	Type	Unit	kg CO_2e	kg CO_2e
WTT- freight flights	Domestic, to/from UK	tonne.km	0.49186	0.49186
	Short-haul, to/from UK	tonne.km	0.25210	0.25210
	Long-haul, to/from UK	tonne.km	0.11157	0.11157
	International, to/from non-UK	tonne.km	0.11157	0.11157

Emission factor source: Greenhouse gas reporting: conversion factors 2021, GOV.UK [7]

Assumption: If unknown, for delivery of trial supplies to patients or GP, use 17.4km as distance from hospital to patient, or hospital to GP. Source: BMJ 2009;339:b4187 [2]

For peer review only - http://bmjopen.bmj.com/site/about/guidelines.xhtml

FUNDED BY NIHR | National Institute for Health and Care Research

LIVERPOOL CLINICAL TRIALS CENTRE LCTC

ICR

2. CTU Emissions

This module includes the following activities:

2.1. Energy consumption at CTU
2.2. Heating
2.3. Trial team commuting

2.1. Energy consumption at CTU (according to staff FTE)

Often, the carbon footprint associated with energy consumption by the CTU cannot be calculated directly because the space or building will be used for other trials not being appraised and non-trial activities. Emissions are therefore estimated based on average per person emissions and the FTE of the trial employees.

According to the UK Employment Destiny Guide, public sector office space is 12 m^2 per FTE. This is multiplied by 59 kWh/m^2, the median electricity intensity for offices, to produce a per person per year usage. The emissions attributed to CTU energy consumption can then be calculated by multiplying the per person usage by the electricity emission factor provided. The calculation is exemplified below.

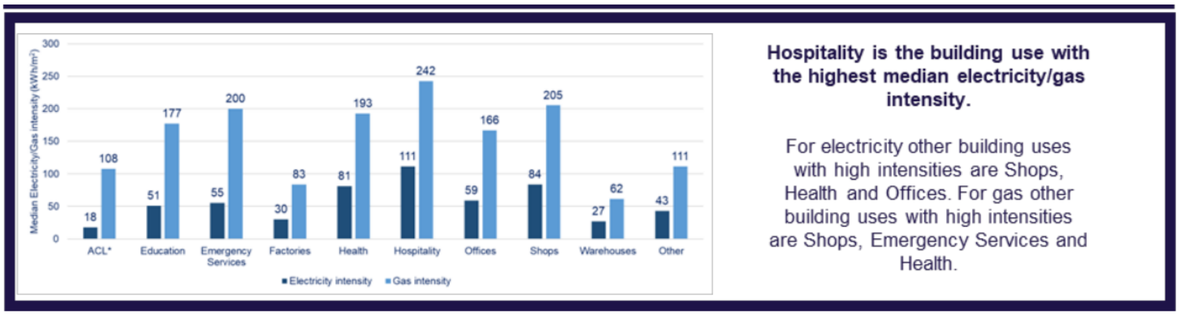

Calculation

12m^2 x 59 kWh/m^2 = 708 kWh per FTE per year

Electricity emission factor = 0.273

Name	Source	Year	Text	Unit	kg CO_2e	kg CO_2e	kg CO_2e	kg CO_2e	kg CO_2e/refrence flow unit
Electricity	Gov.UK	2021		kWh		0.21233	0.00489	0.05529	0.273

708 kWh x 0.273 = 193.3 kgCO_2e per FTE per year

Finally, multiply 193.3 kgCO_2e by the number of years and FTE applicable.

Assumption: According to the UK EMPLOYMENT DENSITY GUIDE, 3rd edition November 2015, office space per FTE is 12 m^2 (public sector) [8]

Office benchmark data source: The Non-Domestic National Energy Efficiency Data-Framework 2022 (England and Wales) (publishing.service.gov.uk)[9]

Electricity emission factor source: Greenhouse gas reporting: conversion factors 2021, GOV.UK [7]

FUNDED BY NIHR | National Institute for Health and Care Research

LIVERPOOL CLINICAL TRIALS CENTRE LCTC

ICR

2.2 Heating

For heating, the calculation follows the same method as above. The $12m^2$ per person is multiplied by the office heating benchmark and then by the natural gas conversion factor provided below.

Office building heating benchmark: **167.27** (kWh/m^2)

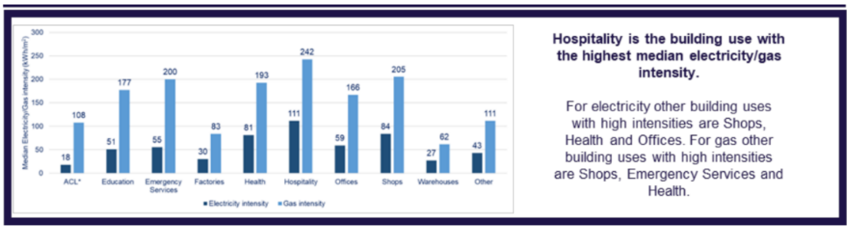

Natural gas conversion factor: **0.215**

Activity	Referen	EF	Comment	Refrence flow	Scope 1/Direc	Scope 2	Scope 3 T&D	Scope 3 WTT	Total Carbon intensity
Name	Source	Year	Text	Unit	kg CO_2e	kg CO_2e	kg CO_2e	kg CO_2e	kg CO_2e/refrence flow unit
Electricity	Gov.UK	2021		kWh		0.21233	0.00489	0.05529	0.273
Burning (hetaing) oil	Gov.UK	2021		litres	2.54014			0.52807	3.068
Natural gas	Gov.UK	2021		kWh (Gross CV)	0.18316			0.03135	0.215
LPG	Gov.UK	2021		litres	1.55709			0.18383	1.741

Calculation

- $12\ m^2 \times 167.27\ kWh/m^2 = 2007.24$ kWh per FTE per year
- $2007.24\ kWh \times 0.215 = 431.56$ kg CO_2e per FTE per year

Multiply 431.56 kg CO_2e by the number of years and FTE applicable

Assumption: If the heating source is unknown, assume that the heating source is natural gas.

Assumption: According to the UK EMPLOYMENT DENSITY GUIDE, 3rd edition November 2015, office space per FTE is 12 m^2 public sector. [8]

Office benchmark data source: The Non-Domestic National Energy Efficiency Data-Framework 2022 (England and Wales) (publishing.service.gov.uk) [9]

Natural gas emission factor source: Greenhouse gas reporting: conversion factors 2021, GOV.UK [7]

Homeworking

Homeworking (office equipment + heating)	per FTE Working Hour	0.34075

Multiply the total number of FTE working hours by the conversion factor (0.34075) to calculate $kgCO_2e$ (includes electricity use from office equipment and heating).

Emission factor source: Greenhouse gas reporting: conversion factors 2022, GOV.UK [10]

FUNDED BY NIHR | National Institute for Health and Care Research

LIVERPOOL CLINICAL TRIALS CENTRE LCTC

ICR

2.3. Trial team commuting

The emissions attributed to the trial team commuting is calculated using average commuting statistics. Using these statistics, the distance travelled by each mode of transport was calculated and multiplied by the relevant emission factor.

The average distance of a commuting journey was 8.8 miles (14km) in 2013/14. Multiplied by 220 commuting days, this results in a total distance of 6160km travelled per year.

Car (used for 68% of the 6160km distance) – 68% of 6160km = 4188.8km

4188.8 x 0.223 = 934.1 kgCO_2e

Large car petrol	Gov.UK	2021	km	0.27909	0.07833	0.357
Average car petrol	Gov.UK	2021	km	0.17431	0.04885	0.223

Rail (used for 10% of the 6160km distance) = 616 km x 0.043 = 26.5 kgCO_2e

Coach	Gov.UK	2021	passenger.km	0.02684	0.00646	0.033
National rail	Gov.UK	2021	passenger.km	0.03549	0.00733	0.043
International rail	Gov.UK	2021	passenger.km	0.00446	0.00074	0.005

Bus (used for 6% of the 6160km distance): 369.6km x 0.127 = 46.9 kgCO_2e

11	Local bus (not London)	Gov.UK	2021	passenger.km	0.11774	0.02868	0.146
12	Local London bus	Gov.UK	2021	passenger.km	0.07718	0.01885	0.096
13	Average local bus	Gov.UK	2021	passenger.km	0.10227	0.02494	0.127
14	Coach	Gov.UK	2021	passenger.km	0.02684	0.00646	0.033

Other (used for 5% of the 6160km distance): Assume 3% cycling so zero emissions, 1% motorbike, 1% taxi

Motorbike: 61.6km x 0.145 = 8.9kgCO_2e

London Underground	Gov.UK	2021	passenger.km	0.02781	0.00461	0.032
Average motorbike	Gov.UK	2021	km	0.11355	0.03134	0.145
Small car diesel	Gov.UK	2021	km	0.13758	0.03344	0.171
Medium car diiesel	Gov.UK	2021	km	0.16496	0.04018	0.205

Taxi: 61.6 x 0.185 = 11.4

8	LPG	Gov.UK	2021	litres	1.55709	0.18383	1.741
9	Regular taxi	Gov.UK	2021	passenger.km	0.14876	0.03632	0.185
10	Black cab	Gov.UK	2021	passenger.km	0.20416	0.05	0.254

For 1 FTE, emissions attributed to commuting for 1 year= 1027.8 kgCO_2e

Multiply by the number of years and FTE applicable

NB: The WTT (well-to-tank) has been included in the emission factors for all modes of transport and therefore does not need to be added manually in this case.

Assumptions:

- 220 days spent commuting. This was calculated by subtracting 10 days sick and 30 days paid leave from the 260 workdays in a year.
- 6160 km is the total distance an employee will commute per year. This was calculated by multiplying 28km (14km per commuting journey) by 220 days.

Sources:

- Transport Statistics Great Britain: 2021 - GOV.UK (www.gov.uk) [11]
- Commuting trends in England 1988 - 2015 (publishing.service.gov.uk) [12]
- Emission factor source: GOV.UK Greenhouse gas reporting, conversion factors 2021 [7]

For peer review only - http://bmjopen.bmj.com/site/about/guidelines.xhtml

FUNDED BY NIHR | National Institute for Health and Care Research

LIVERPOOL CLINICAL TRIALS CENTRE LCTC

ICR

3. Trial specific meetings and travel

This module includes the following activities:

3.1. Visits and travel to site
3.2. Travel to meetings
3.3. Hotel stays
3.4. Sustenance

3.1, 3.2. Visits and travel to site, travel to meetings

For business travel by bus, taxi and rail, the activity data is captured in passenger km (p.km). The number of passengers is multiplied by the distance travelled (km), then by the relevant emission factor.

Number of passengers x total distance (km) = p.km

p.km x suitable emission factor = (kg CO_2e)

Rail

Activity	Type	Unit	kg CO_2e	kg CO_2	kg CH_4	kg N_2O
Rail	National rail	passenger.km	0.03549	0.0351	0.00007	0.00032
	International rail	passenger.km	0.00446	0.00441	0.00002	0.00003
	Light rail and tram	passenger.km	0.02861	0.02832	0.00011	0.00018
	London Underground	passenger.km	0.02781	0.02753	0.0001	0.00018

For business travel you need to **add** the WTT (well-to-tank) value to get the final total. WTT can be calculated by multiplying the p.km used in the first calculation by the correlating WTT conversion factor provided below.

passenger.km x correlating WTT conversion factor

Activity	Type	Unit	kg CO_2e
WTT- rail	National rail	passenger.km	0.00892
	International rail	passenger.km	0.00116
	Light rail and tram	passenger.km	0.00745
	London Underground	passenger.km	0.00724

NB: Always use values relating to 'kgCO_2e'
NB: Distances can be calculated using google maps

Assumption: Distances calculated from CTU to destination

Emission factor source: Greenhouse gas reporting: conversion factors 2021, GOV.UK [7]

Emission factors for all modes can be found in the orange 'business travel' tabs of conversion-factors-2021-full-set-advanced-users.xlsm (live.com)

FUNDED BY NIHR | National Institute for Health and Care Research

3.1, 3.2. Visits and travel to site, travel to meetings

For business travel by air, activity data is also captured in passenger km (p.km). The number of passengers is multiplied by the distance travelled (km), then by the relevant emission factor.

Number of passengers x total distance (km) = p.km

p.km x suitable emission factor = (kg CO_2e)

An emission factor must be chosen from the following categories:

- Domestic
- Short Haul International (≤3700 km) – Average/Economy/Business
- Long Haul International (>3700 km) – Average/Economy/Business
- International (travel between non-UK countries)

Activity	Haul	Class	Unit	kg CO_2e
Flights	Domestic, to/from UK	Average passenger	passenger.km	0.24587
	Short-haul, to/from UK	Average passenger	passenger.km	0.15353
		Economy class	passenger.km	0.15102
		Business class	passenger.km	0.22652
	Long-haul, to/from UK	Average passenger	passenger.km	0.19309
		Economy class	passenger.km	0.14787
		Premium economy class	passenger.km	0.23659
		Business class	passenger.km	0.42882
		First class	passenger.km	0.59147
	International, to/from non-UK	Average passenger	passenger.km	0.18362
		Economy class	passenger.km	0.140625
		Premium economy class	passenger.km	0.225
		Business class	passenger.km	0.40781
		First class	passenger.km	0.56251

For business travel you need to add the WTT (well-to-tank) value to get the final total. WTT can be calculated by multiplying the p.km used in the first calculation by the correlating WTT conversion factor provided below.

passenger.km x correlating WTT conversion factor

				With RF	Without RF
Activity	Haul	Class	Unit	kg CO_2e	kg CO_2e
WTT- flights	Domestic, to/from UK	Average passenger	passenger.km	0.02691	0.02691
	Short-haul, to/from UK	Average passenger	passenger.km	0.01681	0.01681
		Economy class	passenger.km	0.01654	0.01654
		Business class	passenger.km	0.0248	0.0248
	Long-haul, to/from UK	Average passenger	passenger.km	0.02114	0.02114
		Economy class	passenger.km	0.01619	0.01619
		Premium economy class	passenger.km	0.02591	0.02591
		Business class	passenger.km	0.04696	0.04696
		First class	passenger.km	0.06477	0.06477
	International, to/from non-UK	Average passenger	passenger.km	0.02011	0.02011
		Economy class	passenger.km	0.0154	0.0154
		Premium economy class	passenger.km	0.02464	0.02464
		Business class	passenger.km	0.04466	0.04466
		First class	passenger.km	0.06159	0.06159

NB: Always use values relating to 'kgCO_2e'
NB: Distances can be calculated using google maps
NB: 'With RF' values are recommended. RF (combustion and radiative forcing) includes the indirect and direct emissions

Assumption: travellers departed from the nearest airport to their place of work and flew directly to the airport of the city to which they were travelling.
Emission factor source: Greenhouse gas reporting: conversion factors 2021, GOV.UK [7]

Emission factors for all modes can be found in the orange 'business travel' tabs of conversion-factors-2021-full-set-advanced-users.xlsm (live.com)

For peer review only - http://bmjopen.bmj.com/site/about/guidelines.xhtml

FUNDED BY NIHR | National Institute for Health and Care Research

LIVERPOOL CLINICAL TRIALS CENTRE LCTC

ICR

3.2 Travel to meetings

Teleconferencing: for meetings which are conducted by teleconferencing, multiply the number of people and hours by the figure provided below.

A standard videoconferencing service has a carbon footprint of 157 g CO_2e/hr. When the video is switched off, videoconferencing uses 6.9 g CO_2e/hr.

Emission factor source: Turn off that camera during virtual meetings, environmental study says - Purdue University News [13]

3.3 Hotel Stays

To calculate the emissions attributed to hotel stays, the number of hotel rooms is multiplied by the length of stay (in number of nights) and by the conversion factor for the appropriate country to give the associated emissions.

Each country has a different emission factor. The emission factor for the UK is shown below, other countries can be found at conversion-factors-2021-full-set-advanced-users.xlsm (live.com)

	Country	Unit	kg CO_2e
Hotel stay	UK	Room per night	13.9
	UK (London)	Room per night	13.8

NB: A ‘room per night’ accounts for use of the room and does not differentiate for number of travellers staying in the room.

Source: Greenhouse gas reporting: conversion factors 2021, GOV.UK [7]

Alternatively, you may use a cost-based method by multiplying the total cost in the funding application by the emission factor below:

Cost (£) x 0.358 = kg CO_2e

Wholesale distribution	Gov.UK	2020	converted fron	£	0.375
Retail distribution	Gov.UK	2020	converted fron	£	0.277
Hotels, catering, pubs e	Gov.UK	2020	converted fron	£	0.358
Railway transport[5]	Gov.UK	2020	converted fron	£	0.678
Road transport[5]	Gov.UK	2020	converted fron	£	0.690
Water transport[5]	Gov.UK	2020	converted fron	£	1.428
Air transport[5]	Gov.UK	2020	converted fron	£	2.089

Emission factor source: Gov.UK Government conversion factors for company reporting of greenhouse gas emissions 2012 - Annex 13 with consideration of 2020 inflation rates. [14]

For peer review only - http://bmjopen.bmj.com/site/about/guidelines.xhtml

3.4. Sustenance

For the carbon footprint associated with meeting lunches or hotel dinners, multiply the quantity by the relevant emission factor provided below.

Meeting lunches or hotel dinners (vegetarian) = 2.6 $kgCO_2e$ per meal per person

Meeting lunches or hotel dinners (meat) = 5.92 $kgCO_2e$ per meal per person

Emission factor source = WWF, 2018 Food_in_a_warming_world_report.PDF [15]

For peer review only

FUNDED BY NIHR | National Institute for Health and Care Research

LIVERPOOL CLINICAL TRIALS CENTRE LCTC

ICR

4. Intervention

This module includes guidance on the following different types of intervention. Pick the most applicable intervention type from:

4.1 Physical (IMP)
4.2 Clinical (Non-IMP)
4.3 Other (not captured above)

4.1 Physical (an IMP)

4.1.1 Movement of IMP from manufacturing site to distribution/packaging site

4.1.2 Movement of IMP from distribution/packaging site to participating sites or direct to participant

4.1.3 Materials required for the packaging and shipment of IMP

4.1.4 Destruction of overage

Assumptions: Calculations do not include manufacture of IMP.

4.2 Clinical (non-IMP)

NB: not all calculations will be relevant to all interventions.

4.2.1 Movement (shipment) of the intervention, or resources required to deliver the intervention
4.2.2 Materials required for the shipment of the intervention
4.2.3 Utilities required for delivery of the intervention
4.2.4 Activities or resources required/relating to delivery of the intervention

Assumptions: Calculations do not include manufacture of device/machinery/equipment delivering the intervention.

4.3 Other (Not captured above)

NB: not all calculations will be relevant to all interventions, depending on the specific intervention.

4.3.1 Movement of the intervention to the participant or participating site
4.3.2 Materials required for packaging and shipment of the intervention
4.3.3 Materials or resources required for delivery of the intervention
4.3.4 Travel required to facilitate delivery of the intervention

For peer review only

FUNDED BY NIHR | National Institute for Health and Care Research

LIVERPOOL CLINICAL TRIALS CENTRE LCTC

ICR

4.1. Physical (an IMP)

4.1.1.,4.1.2. Movement of intervention to participating site or direct to participant

For freight, please refer to section 1.2, 1.3, 1.4 for the calculation and emission factors. If the delivery is not ambient, follow the guidance provided below.

Refrigerated freight

Increase the total $kgCO_2e$ associated with freight by 15% for samples transported at temperatures of 2-8 degrees.

Frozen (dry ice) freight

When calculating the carbon footprint of frozen shipments, make sure to consider the emissions attributable to the dry ice, both in terms of:

1. Weight - If the total weight of the posted package is not available, when estimating the weight of the sample and box, make sure to include the additional weight due to the dry ice (add/include in normal calculation of weight x distance x emission factor).
2. Emissions of dry ice manufacture: for 1 kg of dry ice, you need to account for 2.22 kg of liquid CO_2 using the Ecoinvent 2.2 data below. E.g., 2.22kg x 0.81605 = **1.81kg CO_2e per 1 kg dry ice** produced/used.

261	443	carbon black, at plant	chemicals	inorganics	kg	GLO	2.3658
262	444	carbon dioxide liquid, at plant	chemicals	inorganics	kg	RER	0.81605
263	445	carbon monoxide, CO, at plant	chemicals	inorganics	kg	RER	1.5539
6949	446	cerium concentrate, 60% cerium oxide, at plant	chemicals	inorganics	kg	CN	8.309

If the amount of dry ice used in frozen shipments is unknown, use an estimated 1kg of dry ice per sample box. Ensure this additional weight is included in the freight calculation and $1.81KgCO_2e$ in the total carbon footprint of the freight.

Assumption: use 1 kg of dry ice per sample box in the absence of established activity data.

Emission factor source: Consultation /estimation by Environmental Resource Management

4.1.3. Materials involved in the packaging and shipment of the intervention

Shipping boxes

For single use (SU) cold storage boxes multiply the number required by 25.2 $kgCO_2e$

For reusable cold storage boxes, multiply the number required by 2.2 $kgCO_2e$

Sample Average Shipping Box (85% ambient / 15% frozen)	1.34	kg CO_2e	per cold storage box	The International Journal of Life Cycle Assessment, Goellner et al. Vol 19, pp 611–619 (2014)
Sample Cold Storage Box Manufacture (SU)	25.2	kg CO_2e	per cold storage box	The International Journal of Life Cycle Assessment, Goellner et al. Vol 19, pp 611–619 (2014)
Sample Cold Storage Box Manufacture (Reuse)	2.2	kg CO_2e	per cold storage box	The International Journal of Life Cycle Assessment, Goellner et al. Vol 19, pp 611–619 (2014)

Emission factor source: The International Journal of Life Cycle Assessment, Goellner et al. Vol 19, pp 611-619 (2014) [16]

For cardboard: Kg (cardboard) x 0.821 = $kgCO_2e$

For polystyrene: kg (polystyrene) x 3.778 = $kgCO_2e$

Greenhouse gas reporting: conversion factors 2021, GOV.UK [7]

For peer review only - http://bmjopen.bmj.com/site/about/guidelines.xhtml

FUNDED BY NIHR | National Institute for Health and Care Research

4.1.4. Destruction of overage

For the destruction of overage, such as the incineration of IMP, multiply the weight in kg of the material being destroyed by the emission factor provided below.

Kg of waste x 2.4252 = kgCO_2e

2958	disposal, hazardous waste, 25% water, to hazardous waste incineration	waste management	hazardous waste incineration	kg	CH	2.4252

Emission factor source: Ecoinvent, version 2.2, 2011 (CH = SWITZERLAND) [6]

4.2. Clinical (e.g. radiotherapy, device, surgery)

NB: not all calculations will be relevant to all interventions. **This section of the method will be further developed as we carbon footprint more trials, so please inform us if your protocol specifies an activity that has not been included, and we will help to determine the associated carbon footprint.**

4.2.1 Movement of intervention, or materials required to deliver the intervention

Please refer to section 1.2 and 4.1.1, 4.1.2.

4.2.2 Materials involved in the shipment of the intervention

Please refer to section 4.1.3.

4.2.3 Utilities required for delivery of the intervention

Please refer to section 7.3 to calculate the emissions attributed to hospital utilities if required to deliver the intervention.

4.2.4 Activities or resources required/relating to delivery of the intervention

Please refer to section 7.2 for consumables, surgery and other activities that may be relevant to the delivery of the intervention, but please take care to avoid double counting.

To calculate the emissions attributed to incineration, please refer to section 4.1.4.

4.3. Other

NB: not all calculations will be relevant to all interventions. **This section of the method will be further developed as we carbon footprint more trials, so please inform us if your protocol specifies an activity that has not been included, and we will help to determine the associated carbon footprint.**

4.3.1 Movement of intervention, or materials required to deliver the intervention

Please refer to section 1.2 and 4.1.1, 4.1.2.

For peer review only - http://bmjopen.bmj.com/site/about/guidelines.xhtml

FUNDED BY NIHR | National Institute for Health and Care Research

4.3.2 Materials required for packaging and shipment of the intervention

Please refer to section 4.1.3

4.3.3 Materials or resources required for delivery of the intervention

For printing and paper, please refer to section 1.1.

4.3.4 Travel required to facilitate delivery of the intervention

Please refer to section 3.1, 3.2 for travel.

For peer review only

FUNDED BY NIHR | National Institute for Health and Care Research
LIVERPOOL CLINICAL TRIALS CENTRE LCTC
ICR

5 Data collection and exchange

This module includes the following activities:

- **5.1. Data collection and query exchange between CTU and sites**
- **5.2. Data sent direct from participants to CTU or participating sites**
- **5.3. Data from labs to CTU**
- **5.4. Data from other collaborators to CTU**

NB: Analysis of data does not need to be calculated separately, it is covered by the emissions attributed to trial staff FTE in "CTU emissions" and by calculations within "Data Collection and exchange".

5.1. Data collection and query exchange between CTU and sites

CRFs

For postage of paper CRFs, please refer to section 1.2 (freight).

Web-based data entry at sites, e.g. CRF completion, will be accounted for in the time a hospital worker spends on the trial and the carbon footprint of the trial databases (See 5.2).

Scans copied to CD

To estimate the emissions attributed to copying patient scans to a CD, add the carbon footprint of CD manufacture to the carbon footprint of computer use.

The carbon footprint of manufacturing a CD = 0.83 kg CO_2e per CD (833 g CO_2e)

Source: Journal of Industrial Ecology, "The Energy and Climate Change Impacts Of Different Music Delivery Methods". Weber et al. Vol 14, Issue 5, pg. 754-769 (2010) [17]

The carbon footprint of copying the scans on to a CD using a computer = 0.18079 kg CO_2e per hour

1269 use, computer, desktop with LCD monitor, active mode	electronics	services	h	RER	0.18079

Emission factor source: Ecoinvent, version 2.2, 2011 [6]

Email traffic

An email without an attachment = 10g CO_2e. Double this for an email with a one-megabyte attachment.

NB: this is an estimate of all emails exchanged between CTU and participating sites throughout the study lifetime, including data query resolution emails.

Source: Carbon footprint of your emails | mail.com blog [18]

For peer review only - http://bmjopen.bmj.com/site/about/guidelines.xhtml

FUNDED BY NIHR | National Institute for Health and Care Research

LIVERPOOL CLINICAL TRIALS CENTRE LCTC

ICR

5.1. (continued)

Data collection via electronic trial databases/systems

The combination of transmitting data and storing it in a data centre requires between 3 to 7 kWh per gigabyte[19]. Therefore multiply 5 kWh by the electricity emission factor (0.273) to calculate the kg CO_2e per GB per year.

5 kwh x 0.273 = 1.365 kg CO_2e per GB per year

Assumption: data storage requires 5 kWh.

Source: Carbon and the Cloud. Hard facts about data storage. | by Stanford Magazine | Stanford Magazine | Medium[19]

5.3. Data sent direct from participants to CTU/participating sites

For paper questionnaires, please refer to section 1.1. for the carbon footprint of producing the materials and section 1.2. for postage (freight).

Electronic questionnaire

For completion of an electronic questionnaire, you must account for both the use of a device to complete the questionnaire and the carbon footprint of data storage and transmission associated with web surfing.

For completion using a **desktop computer**: 0.18079 kg CO_2e per hour

1269	use, computer, desktop with LCD monitor, active mode	electronics	services	h	RER	0.18079

For completion using a **laptop**: 0.028719 kg CO_2e per hour

1284	use, computer, laptop, active mode	electronics	services	h	RER	0.028719

Computer and laptop emission factor source: Ecoinvent, version 2.2, 2011 [6]

For completion using a **tablet**: 0.027397 kg CO_2e per hour

For completion using a **smartphone**: 0.015068 kg CO_2e per hour

Tablet and smartphone emission factor source: Examining the Carbon Footprint of Devices - Sustainable Software (microsoft.com) [20]

Web surfing = 9.441 g CO2e/hr (10 mins = 1.57 g CO_2e)

Source: Resources, Conservation and Recycling, "The overlooked environmental footprint of increasing Internet use". Obringer et al. Vol 167 (2021) [21]

5.4. Data from labs to CTU

For data collection via electronic trial database systems estimate 1.365 kgCO_2e per GB per year.

Source: Carbon and the Cloud. Hard facts about data storage. | by Stanford Magazine | Stanford Magazine | Medium[19]

FUNDED BY NIHR | National Institute for Health and Care Research

5.5. Data from other collaborators to CTU

Data linkage

For data linkage, multiple the cost spent by the emission factor provided below.

Computer services – 0.149 x unit (£) = $kgCO_2e$

42	Real estate activities	Gov.UK	2020	converted fron	£	0.085
43	Renting of machinery e	Gov.UK	2020	converted fron	£	0.232
44	Computer services	Gov.UK	2020	converted fron	£	0.149
45	Research and developn	Gov.UK	2020	converted fron	£	0.216
46	Legal, consultancy and	Gov.UK	2020	converted fron	£	0.121

Emission factor source: Gov.UK Government conversion factors for company reporting of greenhouse gas emissions 2012 - Annex 13 with consideration of 2020 inflation rates. [13]

For peer review only

For peer review only - http://bmjopen.bmj.com/site/about/guidelines.xhtml

FUNDED BY NIHR | National Institute for Health and Care Research

LIVERPOOL CLINICAL TRIALS CENTRE LCTC

ICR

6 Trial supplies and equipment

This module includes the following activities:

6.1. Equipment used by CTU
6.2. Equipment and supplies used by participating sites supplied by CTU
6.3. Equipment and supplies provided to participants specifically for the trial

6.1. Equipment used by CTU

For office machinery and computers purchased for the trial, multiply the cost by the emission factor provided below.

£ x 0.387 = $kgCO_2e$

17	Machinery and equipme	Gov.UK	2020 converted from £	0.512
18	Office machinery and co	Gov.UK	2020 converted from £	0.387
19	Electrical machinery	Gov.UK	2020 converted from £	0.452

Emission factor source: Gov.UK Government conversion factors for company reporting of greenhouse gas emissions 2012 - Annex 13 with consideration of 2020 inflation rates. [13]

6.2. Equipment and supplies used by participating sites supplied by CTU

For the shipment of equipment to participating sites, please refer to section 1.2.

For the use of a centrifuge, please refer to section 9.2., for a fridge or freezer please refer to section 9.3.

Please inform us if your protocol specifies any equipment or supplies that have not been included and we will help to determine the associated carbon footprint.

6.3. Equipment and supplies provided to participants specifically for the trial

Smartphone: For a smartphone, account for 55 $kgCO_2e$ from manufacture and add 5.5 $kgCO_2e$ per year of usage.

Source: Examining the Carbon Footprint of Devices - Sustainable Software (microsoft.com) [19]

Tablet: For a tablet, account for 119 $kgCO_2e$ from manufacture and add 10kg CO_2e per year of usage. Assume a maximum lifetime of 3 years, therefore 30 kg CO_2e is the total possible carbon footprint that can be attributed to use.

Source: Examining the Carbon Footprint of Devices - Sustainable Software (microsoft.com) [19]

Wearables/smart watch: For a smart watch, account for 30.1 kg CO_2e for manufacture and add 1.633 kg CO_2e per year of usage. Assume a maximum lifetime of 3 years, therefore 4.9 kg CO_2e is the total possible carbon footprint that can be attributed to use.

Source: Apple Watch SE Product Environmental Report [22]

To calculate the carbon footprint associated with shipment of the devices, please refer to section 1.2.

For peer review only - http://bmjopen.bmj.com/site/about/guidelines.xhtml

FUNDED BY NIHR | National Institute for Health and Care Research
LIVERPOOL CLINICAL TRIALS CENTRE LCTC
ICR

7 Trial specific patient assessments

This module includes the following activities:

7.1. Travel of patients for study in visits in addition to standard of care (eligibility/screening assessments, trial-specific assessments and procedures)
7.2. Materials and activities required for study assessments in addition to standard of care
7.3. Utilities required for study assessments according to trial staff FTE

7.1. Travel of patients for study in visits that are in addition to standard of care (eligibility/screening assessments, trial-specific assessments and procedures)

Patient travel to elective care (e.g. Hospital)

1 journey by patient associated with attendance at elective care = 2.9 kg CO_2e

Therefore 1 visit to elective care (2 journeys – out and back) = 5.8 kgCO_2e

Patient travel to primary care (e.g. GP)

Emissions associated with attendance at GP surgery (journey out and back) = 1.12 kgCO_2e

Emission factors source: SHC care pathway calculator [23]

7.2. Materials and activities required for study assessments that are in addition to standard of care

This includes everything that happens to the patient in the protocol schedule of assessments which is over and above routine care. This is not an exhaustive list; please inform us if your protocol specifies an activity that has not been included, and we will help to determine the associated carbon footprint.

Surgery

A 1-hour surgery = 53 kg CO_2e

A 30-minute surgery = 26.5 kg CO_2e

Emission factor source: SHC care pathway calculator [23]

Bed days

Low intensity (general ward) = 37.9 kg CO_2e

High intensity (ICU) = 103 kg CO_2e

Emission factor source: SHC care pathway calculator [23]

Scans

1 MRI = 24.7 kg CO_2e

Emission factor source: SHC care pathway calculator [23]

1 CT scan = 9.2 kg CO_2e, 1 CXR = 0.8 kgCO_2e

Emission factor source: The carbon footprint of hospital diagnostic imaging in Australia (thelancet.com) [24]

For peer review only - http://bmjopen.bmj.com/site/about/guidelines.xhtml

FUNDED BY NIHR | National Institute for Health and Care Research

LIVERPOOL CLINICAL TRIALS CENTRE LCTC

ICR

7.2. Materials and activities required for study assessments (continued)

Radiotherapy

To calculate the carbon footprint of radiotherapy, multiply the total treatment power in kWh by the electricity emission factor.

Full course of breast radiotherapy (15 sessions) **= 5.7 kgCO_2e**

Calculation: 1.4 kWh x 0.273 x 15 = 5.7 kgCO_2e

Full course prostate radiotherapy (20 sessions) = **15.3 kgCO_2e**

Calculation: 2.8 kWh x 0.273 x 20 = 15.3 kgCO_2e

Assumption: 15 sessions required for breast radiotherapy. Source: Radiotherapy for breast cancer - Macmillan Cancer Support [25]

Assumption: 20 sessions required for prostate radiotherapy. Source: Radiotherapy for advanced prostate cancer | Prostate Cancer UK [26]

Benchmark data source: PO-1058 Estimating the carbon footprint of the radiotherapy pathway and changes in response to COVID-19 main.pdf (nih.gov) [27]

Emission factor source: GOV.UK Greenhouse gas reporting, conversion factors 2021 [7]

Consumables

For a trial appointment where consumables such as gloves are required, account for **0.30 kgCO_2e per patient per appointment.**

Emission factor source: SHC care pathway calculator guidance GP consultation module, 2015 [28]

Blood investigations

CO_2 e emissions for haematology tests:

- 82 g/test (95% CI, 73-91 g/test) for coagulation profile
- 116 g/test (95% CI, 101-135 g/test) for full blood examination.

CO_2 e emissions for biochemical tests:

- 0.5 g/test CO_2 e (95% CI, 0.4-0.6 g/test) for C-reactive protein (low because typically ordered with urea and electrolyte assessment)
- 49 g/test (95% CI, 45-53 g/test) for arterial blood gas assessment
- 99 g/test (95% CI, 84-113 g/test) for urea and electrolyte assessment.

NB: These emissions include the materials and consumables required for sample collection, phlebotomy and analysis, as well as power consumption by pathology analysers.

Emission factor sourced from: The carbon footprint of pathology testing. Scott McAlister, Alexandra L Barratt, Katy JL Bell and Forbes McGain. Med J Aust 2020; 212 (8): 377-382. Published online: 4 May 2020 **The carbon footprint of pathology testing - McAlister - 2020 - Medical Journal of Australia - Wiley Online Library** [29]

FUNDED BY NIHR | National Institute for Health and Care Research

LIVERPOOL CLINICAL TRIALS CENTRE LCTC

ICR

7.3. Utilities required for study assessments that are in addition to standard of care

The carbon footprint associated with energy consumption by hospital staff cannot be calculated directly because a hospital and the equipment and staff within in it are used for many other non-trial activities. Emissions are therefore estimated based on average per person emissions and the FTE of the trial hospital staff.

This is calculated by multiplying the average space occupied by a hospital staff member (16.5 m^2) by the kWh used per m^2 of a hospital (81 kWh/m^2). The kWh per FTE per year is then multiplied by the electricity emission factor provided below to calculate the carbon footprint attributed per hospital staff FTE. Finally, multiply by the number of years and FTE applicable**.** The calculation is exemplified below.

Calculation

16.5 m^2 x 81 kWh/m^2 = 1336.5 kWh per FTE per year

- Electricity emission factor = 0.273

1336.5 kWh x 0.273 = **364.9 kgCO_2e per FTE per year**

Multiply 364.9 kgCO_2e by the number of years and FTE applicable - Use the trial SOECAT or the costing included in the initial funding application (if predates use of SOECAT) to establish the FTE or total number of hours required by hospital staff for the trial. If using the number of hours, please follow the below method to establish the FTE required for the calculation.

Number of hours in SOECAT / 1762.5 = FTE required for trial.

e.g. if 449.25 total hospital staff hours required for trial.

449.25 / 1762.5 = 0.25. Therefore 25% of 1 hospital staff FTE required

364.9 kgCO_2e x 0.25 = 91.2 kgCO_2e

Assumption: The FTE of a nurse/hospital staff is 1762.5 hours. The standard full-time working week for NHS staff is 37.5 hours (1950 hours - 35 days a year off = 1762.5 hours).

Assumption: each health care professional occupies 16.5m^2 room, source HBN 12 (england.nhs.uk) page 32 [30]

Hospital benchmark data source: The Non-Domestic National Energy Efficiency Data-Framework 2022 (England and Wales) (publishing.service.gov.uk). [9]

Electricity emission factor source: Greenhouse gas reporting: conversion factors 2021, GOV.UK [7]

For peer review only - http://bmjopen.bmj.com/site/about/guidelines.xhtml

FUNDED BY NIHR | National Institute for Health and Care Research

LIVERPOOL CLINICAL TRIALS CENTRE LCTC

ICR

7.3. Utilities required for study assessments that are in addition to standard of care (continued)

Heating

For heating, the calculation follows the same method as above. The 16.5m^2 per person is multiplied by the hospital heating benchmark and then by the natural gas conversion factor provided below.

Hospital building heating benchmark: **193.28** kWh per year per sqm floor area (kWh/m^2)

Natural gas conversion factor: **0.215**

Activity	Referen	EF	Comment	Refrence flow	Scope 1/Direc	Scope 2	Scope 3 T&D	Scope 3 WTT	Total Carbon intensity
Name	Source	Year	Text	Unit	kg CO_2e	kg CO_2e	kg CO_2e	kg CO_2e	kg CO_2e/refrence flow unit
Electricity	Gov.UK	2021		kWh		0.21233	0.00489	0.05529	0.273
Burning (hetaing) oil	Gov.UK	2021		litres	2.54014			0.52807	3.068
Natural gas	Gov.UK	2021		kWh (Gross CV)	0.18316			0.03135	0.215
LPG	Gov.UK	2021		litres	1.55709			0.18383	1.741

16.5 x 193.28 = 3189.1 kWh per FTE per year
3189.1 x 0.215 = 685.7 kgCO_2e per FTE per year

Multiply 685.7 kgCO_2e by the number of years and FTE applicable

Assumption: each health care professional occupies 16.5m^2 room, source HBN 12 (england.nhs.uk) page 32 [30]

Assumption: If the heating source is unknown, assume that the heating source is natural gas.

Hospital benchmark data source: The Non-Domestic National Energy Efficiency Data-Framework 2022 (England and Wales) (publishing.service.gov.uk) [9]

Natural gas emission factor source: Greenhouse gas reporting: conversion factors 2021, GOV.UK [7]

For peer review only - http://bmjopen.bmj.com/site/about/guidelines.xhtml

FUNDED BY NIHR | National Institute for Health and Care Research — Liverpool Clinical Trials Centre LCTC — ICR

8 Samples

This module includes the following activities:

8.1. Materials involved
8.2. Movement of sample kits from manufacturer to CTU
8.3. Movement of sample kits from CTU/distributor to participating sites
8.4. Movement of sample from participating sites to central laboratory

8.1. Material involved

The emissions attributed to sample collection consumables for common blood tests are included in the blood investigations section of 7.2.

Below is a list of commonly used materials used in sample collection and distribution and their equivalent emission factor. Multiply the kg of the material by the yellow highlighted number (the emission factor) to produce a $kgCO_2e$.

Paper and board: board	Gov.UK	2021	kg	0.821
Paper and board: mixed	Gov.UK	2021	kg	0.881
Paper and board: paper	Gov.UK	2021	kg	0.919
Plastics: average plastics	Gov.UK	2021	kg	3.116
Plastics: average plastic film	Gov.UK	2021	kg	2.574
Plastics: average plastic rigid	Gov.UK	2021	kg	3.277
Plastics: HDPE (incl. forming)	Gov.UK	2021	kg	3.270
Plastics: LDPE and LLDPE (incl. forming)	Gov.UK	2021	kg	2.601
Plastics: PET (incl. forming)	Gov.UK	2021	kg	4.032
Plastics: PP (incl. forming)	Gov.UK	2021	kg	3.105
Plastics: PS (incl. forming)	Gov.UK	2021	kg	3.778
Plastics: PVC (incl. forming)	Gov.UK	2021	kg	3.413

Glass	Gov.UK	2021	kg	1.403

Example: 100 x 10ml PET blood tubes (such as Streck), weight 5kg.

5kg x 4.032 (emission factor for PET) = 20.2 $kgCO_2e$

Example: 100 slide mailing containers made of polypropene, weight 1.02 kg

1.02kg x 3.105 = 3.2 $kgCO_2e$

Emission factor source: Greenhouse gas reporting: conversion factors 2021, GOV.UK [7]

For 8.2., 8.3., and 8.4, please refer to section 1.2. for freight and 4.1.1 for refrigerated or frozen freight.

For peer review only - http://bmjopen.bmj.com/site/about/guidelines.xhtml

FUNDED BY NIHR | National Institute for Health and Care Research

LIVERPOOL CLINICAL TRIALS CENTRE LCTC

ICR

9 Laboratory

This module includes the following activities:

9.1. Emissions attributed to lab utilities according to staff FTE

9.2. Materials/equipment/consumables used in processing and analysis of samples

9.3. Storage of samples e.g., utilities and ultra-low temperature freezer

9.1. Laboratory utilities according to staff FTE

The carbon footprint associated with energy consumption by laboratory staff cannot be calculated directly because a laboratory, and the equipment and staff within in it, are used for many other non-trial activities. Emissions are therefore estimated based on average per person emissions and the FTE of the trial laboratory staff.

This is calculated by multiplying the average space occupied by a laboratory staff member (40 m^2) by the kWh used per m^2 of a laboratory (160 kWh/m^2). The kWh per FTE per year is then multiplied by the electricity emission factor provided below to calculate the carbon footprint attributed to 1 FTE for 1 year. Finally, multiply by the number of years and FTE applicable**.** The calculation is exemplified below. Use the trial SOECAT or the costing included in the initial funding application (if predates use of SOECAT) to establish the FTE or total number of hours required by laboratory staff.

Calculation

40m^2 x 160kWh = 6400 kWh per FTE per year

- Electricity emission factor = 0.273

Name	Source	Year	Text	Unit	kg CO_2e	kg CO_2e	kg CO_2e	kg CO_2e	kg CO_2e/refrence flow unit
Electricity	Gov.UK	2021		kWh		0.21233	0.00489	0.05529	0.273

6400 kWh x 0.273 = 1747.2 kgCO_2e per FTE per year

Multiply 1747.2 kgCO_2e by the number of years and FTE applicable

Heating

For heating, the calculation follows the same method as above. The 40m^2 per person is multiplied by the laboratory heating benchmark and then by the natural gas conversion factor provided below.

Laboratory fossil thermal typical benchmark: **160** kWh per year per sqm floor area (kWh/m^2)

Natural gas conversion factor**: 0.215**

Activity	Referen EF		Comment	Refrence flow	Scope 1/Direc	Scope 2	Scope 3 T&D	Scope 3 WTT	Total Carbon intensity
Name	Source	Year	Text	Unit	kg CO_2e	kg CO_2e	kg CO_2e	kg CO_2e	kg CO_2e/refrence flow unit
Electricity	Gov.UK	2021		kWh		0.21233	0.00489	0.05529	0.273
Burning (hetaing) oil	Gov.UK	2021		litres	2.54014			0.52807	3.068
Natural gas	Gov.UK	2021		kWh (Gross CV)	0.18316			0.03135	0.215
LPG	Gov.UK	2021		litres	1.55709			0.18383	1.741

40m^2 x 160kWh = 6400 kWh per FTE per year

6400 kWh x 0.215 = 1376 kgCO_2e per FTE per year

Multiply 1376 kgCO_2e by the number of years and FTE applicable

Assumption: For R&D, 40m^2 required per FTE according to UK EMPLOYMENT DENSITY GUIDE, 3rd edition November 2015 [8]

Assumption: If the heating source is unknown, assume that the heating source is natural gas.

Laboratory benchmark data source: Health Technical Memorandum 07-02: EnCO2de 2015 – making energy work in healthcare (england.nhs.uk) [31]

Emission factor source: Greenhouse gas reporting: conversion factors 2021, GOV.UK [7]

For peer review only - http://bmjopen.bmj.com/site/about/guidelines.xhtml

FUNDED BY NIHR | National Institute for Health and Care Research

LIVERPOOL CLINICAL TRIALS CENTRE LCTC

ICR

9.2. Materials/equipment/consumables used in processing and analysis of samples

To avoid double counting, use of equipment will be included in lab staff FTE if calculated.

However, if the trial does not involve a central lab, but there is still sample processing on site, please see below. For storage of samples, please see section 9.3.

To calculate the emissions of a piece of equipment, multiply the power consumption in Watts by hours used to get a kWh value. Depending on the equipment, this can often be found in the specifications of a listed product. Finally multiply kWh by the electricity emission factor (0.273).

Example: use of a 310-Watt centrifuge for 15 minutes.

310 Watts x 0.25 (hours) = 77.5 kWh

- 77.5 kWh x 0.273 = 21.2 kg CO_2e

Consider the centrifuge capacity and multiply by the number of uses required.

Emission factor source: Greenhouse gas reporting: conversion factors 2021, GOV.UK [7]

9.3. Storage and destruction of biological samples

For storage of samples in a fridge/-20 freezer or an ultra-low temperature freezer, the kWh usage per day is multiplied by 365 to calculate the kWh per year. This is then multiplied by the electricity emission factor and the number of years the samples are stored. However, you will need to make an assumption about the amount of space in the freezer that the trial samples take up.

Fridge/-20 freezer

- 3kWh/day x 365 days = 1095 kWh per year
- 1095 x 0.273 = 298.9 kgCO_2e per year
- Multiply by number of years stored
- This total is for the whole fridge/-20 freezer for 1 year.

-80 freezer

- 22kWh/day x 365 days = 8030 kWh per year
- 8030 x 0.273 = 2192.2 kgCO_2e per year
- Multiply by number of years stored

This total is for the whole freezer for 1 year. You will need to make an assumption about the amount of space in the freezer that the trial samples take up.

Example: if the samples take up a third of the freezer space - 2192.2 kg CO_2e x 0.333 = 723.4 kg CO_2e

Assumptions: A -80°C freezer uses 22 kWh/day, a -20°C freezer uses 3 kWh/day **(**Source: Did You Know? - International Laboratory Freezer Challenge) [32]

Emission factor source: Greenhouse gas reporting: conversion factors 2021, GOV.UK [7]

FUNDED BY NIHR | National Institute for Health and Care Research

10 Trial close out

This module includes the following activities:

10.1. Storage and archiving of essential trial documentation and data
10.2. Storage and destruction of biological samples
10.3. Return of equipment and supplies from participating sites to CTU

For peer review only

For peer review only - http://bmjopen.bmj.com/site/about/guidelines.xhtml

10.1. Storage and archiving of essential trial documentation and data

For the storage of archived documentation, estimate the m^2 required for archiving. This estimate will then be multiplied by the most suitable energy benchmark from the list provided below; choose the type of building most similar to the one the documents are stored in e.g. office/lab/warehouse/health building. Finally multiply by the electricity emission factor.

Energy benchmarks:

- office = 59 kWh/m^2
- laboratory = 160 kWh/m^2
- warehouse = 27 kWh/m^2
- health building = 81 kWh/m^2

Calculation

m^2 required x benchmark = kWh

- Electricity conversion factor = 0.273

kWh x 0.273 = kg CO_2e for 1 year of storage

Multiply by number of years necessary.

Heating

For heating, use the same method as above. Estimate the m^2 used and multiply by the corresponding heating benchmark (i.e., if 'laboratory' was used above then select the same for this calculation). Multiply by the emission factor for natural gas provided below.

Benchmarks:

- office = 167.27 kWh/m^2
- laboratory = 160 kWh/m^2
- warehouse = 62 kWh/m^2
- health building = 193.28 kWh/m^2

Calculation

m^2 required x benchmark = kWh

- Natural gas conversion factor: **0.215**

kWh x 0.215 = kgCO_2e for 1 year of storage

Multiply by number of years necessary.

Assumption: If the heating source is unknown, make assumption the heating source is natural gas.

Benchmark data source: The Non-Domestic National Energy Efficiency Data-Framework 2022 (England and Wales) (publishing.service.gov.uk) [9]

Emission factor source: Greenhouse gas reporting: conversion factors 2021, GOV.UK [7]

FUNDED BY NIHR | National Institute for Health and Care Research

LIVERPOOL CLINICAL TRIALS CENTRE LCTC

ICR

10.1. Storage and archiving of essential trial documentation and data (continued)

For electronic data or documentation storage, estimate 1.365 kgCO_2e per GB per year.

Source: Carbon and the Cloud. Hard facts about data storage. | by Stanford Magazine | Stanford Magazine | Medium[19]

10.2. Storage and destruction of biological samples

See section 9.3. for storage of refrigerated or frozen samples.

See section 10.1 for storage of ambient samples.

10.3. Return of equipment and supplies from participating sites to CTU

See section 1.2. for freight.

For peer review only

FUNDED BY NIHR | National Institute for Health and Care Research

LIVERPOOL CLINICAL TRIALS CENTRE LCTC

ICR

References

[1] Sustainable Trials Study Group. Towards sustainable clinical trials. *BMJ* 2007;334:671

[2] Lyle K, Dent L, Bailey S, et al. Carbon cost of pragmatic randomised controlled trials: retrospective analysis of sample of trials. *BMJ* 2009;339:b4187

[3] Making clinical trials sustainable [Internet]. The Sustainable Healthcare Coalition. [cited 2023 May 11]. Available from: https://shcoalition.org/clinical-trials/

[4] Climate change and health [Internet]. World Health Organization. 2021 October 30 [cited 2023 May 11]. Available from: https://www.who.int/news-room/fact-sheets/detail/climate-change-and-health

[5] What is an emission factor? [Internet]. Climfoot-project.eu. [cited 2023 May 11]. Available from: https://climfoot-project.eu/en/what-emission-factor

[6] Ecoinvent, version 2.2. 2011. Available from: https://ecoinvent.org/the-ecoinvent-database/data-releases/ecoinvent-version-2/

[7] Greenhouse gas reporting: conversion factors [Internet]. GOV.UK 2021. [cited 2023 May 11]. Available from: https://www.gov.uk/government/publications/greenhouse-gas-reporting-conversion-factors-2021

[8] EMPLOYMT DENSITY GUIDE (3rd Edition) [Internet]. Homes & Communities Agency. November 2015 [cited 2023 May 11]. Available from: https://www.kirklees.gov.uk/beta/planning-policy/pdf/examination/national-evidence/NE48_employment_density_guide_3rd_edition.pdf

[9] The Non-Domestic National Energy Efficiency Data-Framework (England and Wales) [Internet]. Department for Business, Energy & Industrial Strategy. 2022 [cited 2023 May 11]. Available from: https://assets.publishing.service.gov.uk/government/uploads/system/uploads/attachment_data/file/1086903/non_domestic_need_data_framework_2022.pdf

[10] Greenhouse gas reporting: conversion factors 2022 [Internet]. Gov.uk. 2022 [cited 2023 May 11]. Available from: https://www.gov.uk/government/publications/greenhouse-gas-reporting-conversion-factors-2022

[11] Transport Statistics Great Britain: 2021 [Internet]. GOV.UK (www.gov.uk). 2021 December 16 [cited May 11 2023]. Available from: https://www.gov.uk/government/statistics/transport-statistics-great-britain-2021/transport-statistics-great-britain-2021

[12] Commuting trends in England 1988-2015 [Internet]. GOV.UK. 2017 November 7 [cited 2023 May 11]. Available from: https://www.gov.uk/government/publications/commuting-trends-in-england-1988-to-2015

[13] Kayla Wiles. Turn off that camera during virtual meetings, environmental study says [Internet]. Purdue University News. 2021 January 14 [cited 2023 May 11]. Available from: https://www.purdue.edu/newsroom/releases/2021/Q1/turn-off-that-camera-during-virtual-meetings,-environmental-study-says.html

[14] Gov.UK Government conversion factors for company reporting of greenhouse gas emissions 2012 – Annex 13 (with consideration of 2020 inflation rates)

[15] WWF. 2018. Food in a warming world [Internet]. [cited 2023 May 11]. Available from: https://www.wwf.org.uk/sites/default/files/2018-03/Food_in_a_warming_world_report.PDF?source=post_page---------------------------

[16] Goellner et al. The International Journal of Life Cycle Assessment. Vol 19, pp 611-619 (2014)

[17] Weber et al. Journal of Industrial Ecology, “The Energy and Climate Change Impacts Of Different Music Delivery Methods”. Vol 14, Issue 5, pg. 754-769 (2010)

[18] What’s the carbon footprint of an email? [Internet]. Mail.com blog. 202 April 21 [cited 2023 May 11]. Available from: https://www.mail.com/blog/posts/email-carbon-footprint/9/

[19] Justin Adamson. Carbon and the Cloud. Hard facts about data storage [Internet]. Stanford Magazine. 2017 May 15 [cited 2023 May 11]. Available from: https://medium.com/stanford-magazine/carbon-and-the-cloud-d6f481b79dfe

[20] Srilatha Manne. Examining the Carbon Footprint of Devices [Internet]. Microsoft.com. 2020 November 23 [cited 2023 May 11. Available from: https://devblogs.microsoft.com/sustainable-software/examining-the-carbon-footprint-of-devices/

[21] Obringer, R., Rachunok, B., Maia-Silva, D., Arbabzadeh, M., Nateghi, R., & Madani, K. (2021). The overlooked environmental footprint of increasing Internet use. *Resources, Conservation and Recycling*, *167*, [105389]. https://doi.org/10.1016/j.resconrec.2020.105389

[22] Apple watch SE. Product Environmental Report – Apple [Internet]. [cited 2023 May 11]. Available from: https://www.apple.com/by/environment/pdf/products/watch/Apple_Watch_SE_PER_sept2020.pdf

[23] Care pathways carbon footprint calculator [Internet]. Sustainable Healthcare Coalition. [cited 2023 May 11]. Available from: https://shcpathways.org/full-calculator/

[24] McAlister S, McGain F, Petersen M, Story D, Charlesworth K, Ison G, Barratt A. The carbon footprint of hospital diagnostic imaging in Australia. Lancet Reg Health West Pac. 2022 May 3;24:100459. doi: 10.1016/j.lanwpc.2022.100459. PMID: 35538935; PMCID: PMC9079346.

[25] Radiotherapy for breast cancer [Internet]. Macmillan.org. [cited 2023 May 11]. Available from: https://www.macmillan.org.uk/cancer-information-and-support/treatments-and-drugs/radiotherapy-for-breast-cancer

[26] Radiotherapy for advanced prostate cancer [Internet]. Prostate Cancer UK. [cited 2023 May 11]. Available from: https://prostatecanceruk.org/prostate-information-and-support/treatments/radiotherapy-for-advanced-prostate-cancer

[27] J. Cummings, C. Taylor, R. Chuter1. PO-1058 Estimating the carbon footprint of the radiotherapy pathway and changes in response to COVID-19. Radiotherapy and oncology. 2022. DOI:10.1016/S0167-8140(22)03022-5

For peer review only - http://bmjopen.bmj.com/site/about/guidelines.xhtml

FUNDED BY NIHR | National Institute for Health and Care Research

[28] SHC care pathway calculator guidance, GP consultation module, page 18, 2015. Available from: https://shcoalition.org/sustainable-care-pathways-guidance/
[29] McAlister, S., Barratt, A.L., Bell, K.J. and McGain, F. (2020), The carbon footprint of pathology testing. Med. J. Aust., 212: 377-382. https://doi.org/10.5694/mja2.50583
[30] Health Building Note (HBN) 12, page 32. NHS Estates. 2004 [cited 2023 May 11]. Available from: https://www.england.nhs.uk/wp-content/uploads/2021/05/HBN_12.pdf
[31] Health Technical Memorandum 07-02: EnCO2de 2015 – making energy work in healthcare. Department of Health. 2015 [cited 2023 May 11]. Available from: https://www.england.nhs.uk/wp-content/uploads/2021/05/HTM_07-02_Part_A_FINAL.pdf
[32] Freezer challenge blog. Did you know? [Internet]. Freezerchallenge.org. 2020 June 19 [cited 2023 May 11]. Available from: https://www.freezerchallenge.org/fc-blog/did-you-know

Acknowledgements

This work was produced through funding from the NIHR entitled **NIHR Clinical Trials Unit Support Funding Opportunity – Supporting efficient / innovative delivery of NIHR research**

Authors: Jessica Griffiths, Lisa Fox, Paula Williamson

On behalf of the Low Carbon Clinical Trials working group: Fiona Adshead, Rustam Al-Shahi Salman, Craig Anderson, Emma Bedson, Judith Bliss, Ana Boshoff, Xiaoying Chen, Denise Cranley, Peter Doran, Carrol Gamble, Kerenza Hood, Naomi McGregor, Carolyn McNamara, Elis Midha, Keith Moore, Alexis M Perkins, Sarah Pett, Matthew R Sydes.

Grateful thanks to Environmental Resources Management for their technical support and expertise in the development of the method.

For peer review only

For peer review only - http://bmjopen.bmj.com/site/about/guidelines.xhtml

Enabling lower carbon clinical trials: Development and prototype testing of a method to quantify the carbon footprint of clinical trials to inform future lower carbon clinical trial design

Guidance and method to calculate the carbon footprint of a clinical trial

For peer review only

Data collation quick guide and worksheet

This guidance provides information on how to carbon footprint a clinical trial for the purposes of the NIHR-funded project ‘enabling lower carbon clinical trials.’

Within the guidance, clinical trial processes have been sub-divided into the following modules:

1. Trial set up
2. CTU emissions
3. Trial specific meetings and travel
4. Treatment intervention
5. Data collection and exchange
6. Trial supplies and equipment
7. Trial specific patient assessments
8. Samples
9. Laboratory
10. Trial close out

This list is not exhaustive, and it is expected that further activities and modules may need to be added to account for specialist processes in all clinical trial types.

NB: analysis of data does not need to be calculated separately, it is covered by the emissions attributed to trial staff FTE in “CTU emissions” and calculations included within “Data Collection and exchange”.

For peer review only - http://bmjopen.bmj.com/site/about/guidelines.xhtml

FUNDED BY NIHR | National Institute for Health and Care Research

LIVERPOOL CLINICAL TRIALS CENTRE LCTC

ICR

In addition to this quick guide and worksheet, we have produced a detailed guidance and method document defining the project scope, limitations and assumptions. The detailed guidance contains a more in depth look and explanation of the calculations found in this document, including emission factor and benchmark data sources, and should be referred to when using this worksheet.

Introduction to calculating carbon footprint

A carbon footprint is a measure of greenhouse gases, usually quoted in kg or tonnes of carbon dioxide equivalent (CO_2e). To calculate the carbon footprint of a particular clinical trial process, both 'activity data' and 'emission factors' are required.

An emission factor, also known as a conversion factor, "is a coefficient which allows you to convert activity data into greenhouse gas emissions. It is the average **emission** rate of a given source, relative to units of activity or process/processes."[1]

The activity data is provided by the user and multiplied by the emission factors provided in this guidance document.

Data collation quick guide and worksheet

This data collation quick guide should be used in conjunction with the "Enabling lower carbon clinical trials: Development and prototype testing of a method to quantify the carbon footprint of clinical trials to inform future lower carbon clinical trial design - Detailed Guidance and method to calculate the carbon footprint of a clinical trial". The guidance document provides the detailed explanation of how calculations should be considered and calculated. This quick guide should be used to collate the trial-specific processes, necessary activity data and to record the subsequent calculations. It is important to avoid double-counting activities i.e., modules must not include activities already covered elsewhere in the clinical trial process map. Please complete this worksheet for each trial to be carbon footprinted.

NB: We are using the term 'CTU' to describe the organisation that manages all aspects of central trial management. For some institutions some of those tasks maybe done by groups outside the CTU team e.g., sponsor office/CRO etc.

For peer review only - http://bmjopen.bmj.com/site/about/guidelines.xhtml

FUNDED BY NIHR | National Institute for Health and Care Research

Module	Examples	Trial activity data	Calculation	Total
**1. Trial set-up**				
1.1. Production of trial documentation to be sent to sites or participants	E.g. Site Investigator File and contents, Site Pharmacy File and contents, CRF Folder and contents, PIS/Cs, GP letters	Number of pages:<br><br>Number of folders used to send trial documentation:<br><br>**For eTMF:**<br>GB required for data storage and transmission:<br>Duration of data storage:	**Paper:**<br>[no. of page] x 0.005 = paper weight (kg)<br><br>b/w printing: Kg of paper x 0.22438 = kgCO_2e<br>Colour printing: Kg of paper x 0.31786 = kgCO_2e<br>Materials (paper): Kg of paper x 0.919 = kgCO_2e<br><br>**Folders**:<br>Kg (of cardboard) x 0.821 = kgCO_2e<br>Assumption: Weight of lever arch = 0.5kg<br>Assumption: Weight of ring binder = 0.3kg<br><br>**eTMF:**<br>Estimate 1.365 kg CO_2e per GB per year.	

For peer review only - http://bmjopen.bmj.com/site/about/guidelines.xhtml

FUNDED BY NIHR | National Institute for Health and Care Research
LIVERPOOL CLINICAL TRIALS CENTRE LCTC
ICR

1.2. Provision/postage of trial documentation to sites<br><br>1.3. Provision/postage of documentation to participants by CTU or participating sites<br><br>1.4. Provision/postage of incentives to participant	E.g. Site Investigator File and contents, Site Pharmacy File and contents, CRF Folder and contents, PIS/Cs, GP letters	Estimated weight of delivery (road):<br><br>Estimated distance of delivery (road):<br><br>Estimated weight of delivery (air):<br><br>Estimated distance of delivery (air):	Delivery weight (tonnes) x distance (km) = t.km<br><br>For road freight: t.km x 0.19443 = kgCO_2e<br><br>For air freight: t.km x required emission factor below = kgCO_2e<br><br>▪ Domestic (to/from UK) = 4.98549<br>▪ Short-haul (to/from UK) = 2.55439<br>▪ Long-haul (to/from UK) = 1.13047<br>▪ International (to/from non-UK) = 1.13047<br><br>For delivery of trial supplies to patients or GP, if unknown, use 17.4km as distance from hospital to patient, or hospital to GP.	
**2. CTU emissions**				
2.1. Energy consumption at CTU according to trial staff FTE	E.g. energy consumption per square metre of air-conditioned office space	Trial duration:<br>Trial staff FTE:	**Energy consumption for 1 FTE for 1 year** = 193.3 kgCO_2e<br><br>Multiply by the number of years and FTE applicable	

For peer review only

FUNDED BY NIHR | National Institute for Health and Care Research

LIVERPOOL CLINICAL TRIALS CENTRE LCTC

ICR

2.2. Heating	E.g. energy consumption at coordination centre attributed to heating (natural gas), homeworking	Trial duration:<br>Trial staff FTE:	**Heating for 1 FTE for 1 year =** 431.56 $kgCO_2e$<br><br>Multiply by the number of years and FTE applicable<br><br>**Homeworking =** 0.34075 $kgCO_2e$ per FTE working hour (includes electricity for office equipment and heating).<br><br>Total FTE working hours x 0.34075 = $kgCO_2e$	
2.3. Trial team commuting	E.g. Car, rail, bus, walking etc	Trial duration:<br>Trial staff FTE:	For 1 FTE for 1 year, total average commuting emissions = 1027.8 $kgCO_2e$<br><br>**Multiply by the number of years and FTE applicable**<br><br>If you can avoid using averages, refer to section 2.3 of the detailed guidance for the emission factors of individual modes of transport.	
**3. Trial specific meetings and travel**				
3.1. Visits and travel to site<br>3.2. Travel to meetings	E.g. Feasibility, site initiation and monitoring visits, audits, inspections, TMG, TSC, IDMC, and	Estimated distance travelled:<br><br>Number of passengers:	Number of passengers x total distance (km) = p.km	

For peer review only

	investigator meetings, PPIE, conferences, scientific meetings etc		For national rail: p.km x 0.04441= kgCO_2e<br><br>For flights: p.km x relevant emission factor below<br><br>- Domestic (average) to/from UK: 0.27278<br>- Short-haul (average) to/from UK: 0.17034<br>- Long-haul (average) to/from UK: 0.21423<br>- International (average) to/from non-UK: 0.20373<br><br>NB: Distances may be calculated using google maps and calculated from CTU to destination<br><br>**Videoconferencing** = 157 grams CO_2e per hour.	
3.3. Hotel stays	E.g. monitoring visits, audits, inspections etc	Number of rooms:<br>Number of nights:	For UK: number of hotel rooms x number of nights x 13.9 = kgCO_2e<br><br>For UK (London) = number of rooms x number of nights x 13.8 = kgCO_2e.	

For peer review only

FUNDED BY NIHR | National Institute for Health and Care Research

			For other countries use conversion factors from orange 'hotel stay' tab: conversion-factors-2021-full-set-advanced-users.xlsm (live.com)<br><br>Alternatively, you may use cost-based method:<br>£ spent x 0.358 = $kgCO_2e$	
3.4. Sustenance	E.g. meeting lunches, hotel dinners	Number of lunches or dinners:	Meeting lunches or hotel dinners (vegetarian) = 2.6 $kgCO_2e$ per meal per person<br><br>Meeting lunches or hotel dinners (meat) = 5.92 $kgCO_2e$ per meal per person	
**4. Intervention***<br>**4.1. Physical (IMP)**<br>**4.2. Clinical (non-IMP)**<br>**4.3. Other (not captured above)**<br>**Please fill out the section most relevant to the intervention being investigated.**				
**4.1. Physical**				
4.1.1, 4.1.2. Movement of intervention, or materials	E.g. movement of intervention from manufacturing site to	Estimated weight and distance of delivery (t.km):	Carry out freight calculation as described in section 1.2.	

* As per assumptions detailed in the guidance, manufacture of the intervention is considered out of scope. This section defines all processes relating to providing and delivering the trial intervention that are over and above routine care.

For peer review only - http://bmjopen.bmj.com/site/about/guidelines.xhtml

required to deliver the intervention	distribution site, shipment of IMP to participating sites or direct to participant		For refrigerated freight, increase the total $kgCO_2e$ associated with freight by 15%.<br><br>Frozen freight:<br>Dry ice has a carbon footprint of **1.81kg CO_2e for 1 kg dry ice** produced/used.<br><br>When calculating the overall emissions of frozen freight, as well as the 1.81 $kgCO_2e$ per 1kg attributed to manufacture, include the weight (kg) of dry ice used in the weight of the freight calculation in section 1.2.<br><br>In the absence of activity data, assume 1kg of dry ice is used per individual sample shipping box.	
4.1.3. Materials required for the packaging and shipment of IMP	E.g. cardboard, cold storage boxes, polystyrene	Number of cold storage boxes:<br><br>Kg of cardboard/ polystyrene:	Single use sample cold storage box = 25.2 $kgCO_2e$ per box<br><br>Reusable sample cold storage box = 2.2 $kgCO_2e$ per box<br><br>Kg (cardboard) x 0.821 = $kgCO_2e$	

For peer review only

			Kg (polystyrene) x 3.778 = kgCO_2e	
4.1.4. Destruction of overage	E.g. incineration of IMP	Estimated weight of overage incinerated:	Kg of waste x 2.4252 = kgCO_2e	
**4.2. Clinical** e.g., radiotherapy, device, surgical.<br>NB: not all calculations will be relevant to all interventions. **This section of the method will be further developed as we carbon footprint more trials, so please inform us if your protocol specifies an activity that has not been included, and we will help to determine the associated carbon footprint.**				
4.2.1 Movement of the intervention, or resources required to deliver the intervention	E.g. movement of intervention from manufacturing site to distribution site, shipment of intervention to participating sites.	Estimated weight and distance of delivery (t.km):	Please refer to section 1.2 and 4.1.1, 4.1.2.	
4.2.2 Materials required for the packaging and shipment of the intervention	E.g. cardboard, cold storage boxes, polystyrene	Number of cold storage boxes:<br><br>Kg of cardboard/ polystyrene:	Please refer to section 4.1.3.	
4.2.3 Utilities required for delivery of the intervention	E.g. Hospital utilities if the intervention is delivered within a hospital	Hospital staff FTE required:	Please refer to section 7.3 to calculate the emissions attributed to hospital utilities if required to deliver the intervention.	

For peer review only - http://bmjopen.bmj.com/site/about/guidelines.xhtml

FUNDED BY NIHR | National Institute for Health and Care Research

LIVERPOOL CLINICAL TRIALS CENTRE LCTC

ICR

4.2.4 Activities or resources required/relating to delivery of the intervention	E.g. consumables, surgical time, specialist equipment, incineration of surgical waste		Please refer to section 7.2 for consumables, surgery and other activities that may be relevant to the delivery of the intervention, but please take care to avoid double counting.<br><br>To calculate the emissions attributed to incineration, e.g. of surgical waste, please refer to section 4.1.4.	
**4.3. Other**<br>NB: not all calculations will be relevant to all interventions. **This section of the method will be further developed as we carbon footprint more trials, so please inform us if your protocol specifies an activity that has not been included, and we will help to determine the associated carbon footprint.**				
4.3.1 Movement of the intervention to the participant or participating site	E.g. shipment of intervention to participating sites or direct to participant	Estimated weight and distance of delivery (t.km):	Please refer to section 1.2.	
4.3.2 Materials required for packaging and shipment of the intervention	E.g. cardboard, cold storage boxes, polystyrene	Number of cold storage boxes:<br><br>Kg of cardboard/ polystyrene:	Please refer to section 4.1.3.	
4.3.3 Materials or resources required for delivery of the intervention	E.g. software, booklets, specialist equipment		For printing and paper, please refer to section 1.1.	
4.3.4 Travel required to facilitate delivery of the intervention	E.g. to deliver training, conduct interviews etc	Estimated distance travelled, number of passengers (p.km):	Please refer to section 3.1, 3.2.	
**5. Data collection and exchange**				

For peer review only - http://bmjopen.bmj.com/site/about/guidelines.xhtml

NB: analysis of data does not need to be calculated separately, it is covered by the emissions attributed to trial staff FTE in "CTU emissions" and calculations included within "Data Collection and exchange".				
5.1. Data collection and query exchange between CTU and sites	E.g. CRFs, EDC completion and query resolution, scans copied to CDs	Estimated weight and distance of deliveries (t.km):<br><br>Number of CDs and time taken to copy scans:<br><br>Number of emails:<br>NB: this is an estimate of all emails exchanged between CTU and participating sites throughout the study lifetime, including data query resolution emails.<br><br>GB required for data storage and transmission:<br>Duration of data storage:<br><br>NB: Web-based data entry at sites, e.g. CRF completion, will be accounted for in the time a hospital worker spends on the trial and the carbon footprint of the trial databases.	For postage of materials, please refer to section 1.2 (freight).<br><br>The carbon footprint of manufacturing a CD = 0.83 kg $C0_2e$ per CD (833 g $C0_2e$)<br><br>The carbon footprint of copying the scans on to a CD using a computer = 0.18079 kg $C0_2e$ per hour<br><br>An email without an attachment = 10g CO_2e. Double this for an email with a one-megabyte attachment.<br><br>Data storage and transmission: estimate 1.365 kg CO_2e per GB per year.	
5.2. Data sent direct from participants to CTU or participating sites	E.g. Questionnaires, patient diaries, wearables	Estimated weight, and distance of delivery (t.km):<br><br>Device used and time taken to complete electronic questionnaires:	For paper questionnaires, please refer to section 1.1. for the carbon footprint of producing the materials and section 1.2. for postage (freight).	

For peer review only - http://bmjopen.bmj.com/site/about/guidelines.xhtml

FUNDED BY NIHR | National Institute for Health and Care Research
LIVERPOOL CLINICAL TRIALS CENTRE LCTC
ICR

			For use of smart watches and other devices see section 6.3.<br><br>**Electronic questionnaires**<br>Add the emissions attributed to data storage and transmission to the emissions attributed to using a device to complete the questionnaire.<br><br>Web surfing (data storage and transmission) = 9.441 g CO2e/hr (10 mins = 1.57 g CO_2e)<br><br>Choose from the below:<br>- Desktop computer = 0.18079 kg CO_2e per hour<br>- Laptop = 0.028719 kg CO_2e per hour<br>- Tablet = 0.027397 kg CO_2e per hour<br>- Smartphone = 0.015068 kg CO_2e per hour	

For peer review only

FUNDED BY NIHR | National Institute for Health and Care Research

5.3. Data from labs to CTU<br>5.4. Data from other collaborators to CTU	E.g. Laboratory patient results, data linkage	GB required for data storage and transmission:<br>Duration of data storage:<br><br>Total £ spent on computer services:	For electronic storage, estimate 1.365 kgCO_2e per GB per year.<br><br>For computer services such as data linkage:<br>£ x 0.149 = kgCO_2e	
**6. Trial supplies and equipment**				
6.1. Equipment used by CTU	E.g. computers, laptops, printers, software	Total £ spent on office machinery and computers for trial:	For any new office machinery and computers purchased specifically for trial:<br>£ x 0.387 = kgCO_2e	
6.2. Equipment and supplies used by participating sites supplied by CTU	E.g. centrifuge, fridge, freezer	Estimated weight and distance of delivery(t.km):	For the shipment of equipment to participating sites, please refer to section 1.2.<br><br>For the use of a centrifuge, please refer to section 9.2., for a fridge or freezer please refer to section 9.3.	
6.3. Equipment and supplies provided to participants specifically for the trial	E.g. wearables, smartphone, tablet	Number of devices and duration of their usage:<br><br>Estimated weight and distance of deliveries (t.km):	**Smartphone =** 55 kgCO_2e from manufacture and add 5.5 kgCO_2e per year of usage.<br><br>**Tablet =** 119 kgCO_2e from manufacture and add 10 kgCO_2e per year of usage.	

For peer review only

			**Wearables/smart watch** = 30.1 kg CO_2e for manufacture and add 1.633 kg CO_2e per year of usage.<br><br>**To calculate the carbon footprint associated with shipment of the devices, please refer to section 1.2.**	
**7. Trial specific patient assessments**				
7.1. Patient travel for study visits that are in addition to standard of care	E.g. Eligibility and screening assessments, trial-specific assessments and procedures	Number of times patient is required to travel (in addition to standard of care):<br><br>Number of patients:	Emissions associated with one patient visit to hospital (UK) = 5.8 kgCO_2e (this includes both the out and back journeys)<br><br>Emissions associated with one patient visit to GP surgery (UK) = 1.12 kgCO_2e (this includes both the out and back journeys)	
7.2. Materials and activities required for study assessments that are in addition to standard of care	E.g. Laboratory tests, imaging assessments, clinical activities relating to intervention for example administering of study drug, biopsy.	Patient schedule of assessments:	Consumables = 0.30 kgCO_2e per patient per trial appointment where consumables (such as gloves) required<br><br>▪ 1 MRI = 24.7 kg CO_2e<br>▪ 1 CT scan = 9.2 kgCO_2e<br>▪ 1 Chest X-Ray = 0.8 kgCO_2e	

For peer review only - http://bmjopen.bmj.com/site/about/guidelines.xhtml

FUNDED BY NIHR | National Institute for Health and Care Research — LIVERPOOL CLINICAL TRIALS CENTRE LCTC — ICR

			▪ 1 hour in surgery = 53 kg CO_2e<br>▪ 1 low intensity (general ward) bed day = 37.9 kg CO_2e<br>▪ 1 high intensity (ICU) bed day = 103 kgCO_2e<br>▪ 15 sessions of breast radiotherapy = 5.7 kg CO_2e<br>▪ 20 sessions of prostate radiotherapy = 15.3 kg CO_2e<br><br>Blood tests:<br>▪ 82 g CO_2e for coagulation profile<br>▪ 116 g CO_2e for full blood examination<br>▪ 49 g CO_2e for arterial gas assessment<br>▪ 99 g CO_2e for urea and electrolyte assessment<br>▪ 0.5 g CO_2e for C-reactive protein<br><br>**Please note that the above figures for blood tests include the materials and consumables required for sample collection, phlebotomy and analysis, as**	

For peer review only - http://bmjopen.bmj.com/site/about/guidelines.xhtml

FUNDED BY NIHR | National Institute for Health and Care Research

			**well as power consumption by pathology analysers.**	
7.3. Utilities required for study assessments that are in addition to standard of care	E.g. energy consumption per square metre of hospital space according to trial staff FTE, taking into account time required for CRF completion and study assessments, consent etc	Trial duration:<br>Trial staff FTE:	1 FTE 1 year = 364.9 $kgCO_2e$<br><br>**Multiply by the number of years and FTE applicable**<br><br>Heating: 1 FTE, 1 year = 685.7 $kgCO_2e$<br>**Multiply by the number of years and FTE applicable**	
**8. Samples**				
8.1. Materials involved	E.g. sample collection kit and packaging for shipment	Kg of material:	The emissions attributed to sample collection consumables for common blood tests are included in the blood tests listed in section 7.2.<br><br>To calculate the carbon footprint of other common materials, multiply the weight in kg by the relevant emission factor below to produce $kgCO_2e$.<br>- Average plastics: 3.116<br>- Plastics (average film): 2.754<br>- Plastics (Average rigid): 3.277	

For peer review only - http://bmjopen.bmj.com/site/about/guidelines.xhtml

FUNDED BY NIHR | National Institute for Health and Care Research · LIVERPOOL CLINICAL TRIALS CENTRE LCTC · ICR

			- Plastics (PP): 3.105<br>- Plastics (PET): 4.032<br>- Glass: 1.403<br>- Paper: 0.919<br>- Board: 0.821<br><br>Example: 100 slide mailing containers made of polypropene = 1.02 kg<br>1.02kg x 3.105 = 3.2 $kgCO_2e$	
8.2. Movement of sample kit materials from manufacturer to CTU<br>8.3. Movement of sample kits from CTU/distributor to participating Sites<br>8.4. Movement of samples from participating sites or patients to central laboratory.	E.g. shipment of blood tubes for sample kits to CTU	Estimated weight of and distance of delivery (t.km):	Please refer to section 1.2. for freight and 4.1. for refrigerated or frozen freight.	
**9. Laboratory**				
9.1. Emissions attributed to lab utilities according to staff FTE	E.g. energy consumption per square metre of laboratory space according to trial staff FTE	Trial duration:<br>Trial staff FTE:	**Electricity:**<br>1747.2 $kgCO_2e$ per FTE per year<br>Multiply 1747.2 $kgCO_2e$ by the number of years and FTE applicable<br><br>**Heating:**	

For peer review only

FUNDED BY NIHR | National Institute for Health and Care Research — Liverpool Clinical Trials Centre LCTC — ICR

			1376 kgCO_2e per FTE per year<br>Multiply 1376 kgCO_2e by the number of years and FTE applicable	
9.2. Materials/equipment/consumables used in processing and analysis of samples	E.g. centrifuges, refrigerators	kWh usage of equipment:<br><br>Kg of material:	To avoid double counting, use of equipment will be included in lab staff FTE if calculated.<br><br>If the trial does not involve a central lab, but there is still sample processing on site, please see below. For storage of samples, please see section 9.3.<br><br>To calculate the emissions of a piece of equipment, multiply the power consumption by hours used to get a kWh value. Finally multiply kWh by the electricity emission factor (0.273).<br><br>Example: use of a 310-Watt centrifuge for 15 minutes<br>- 310 Watts x 0.25 (hours) = 77.5 kWh<br>- 77.5 kWh x 0.273 = 21.2 kgco_2e	

For peer review only - http://bmjopen.bmj.com/site/about/guidelines.xhtml

FUNDED BY NIHR | National Institute for Health and Care Research

			Consider the centrifuge capacity and multiply by the number of uses required.	
9.3. Storage of samples	E.g. utilities and ultra-low temperature freezer	Duration of storage:<br><br>Amount of refrigerator/freezer space required:	Storage in fridge/-20 freezer:<br>- 298.9 kgCO_2e per year<br>- Multiply by number of years stored<br><br>Storage in an ultra-low/-80 freezer:<br>- 2192.2 kgCO_2e per year<br>- Multiply by number of years stored<br><br>NB: this is for a whole freezer; you will need to make an assumption about the amount of space in the freezer that the trial samples take up.	
**10. Trial close out**				
10.1. Storage and archiving of essential trial documentation and data	E.g. Hospital files, lab files, trial guidance documents etc	Duration of storage:<br><br>Amount of space required for storage:	Carbon footprint associated with $1m^2$ for 1 year:<br>- Office: 16.1 kg$C0_2$e<br>- Laboratory: 43.7 kg$c0_2$e<br>- Warehouse: 7.4 kg$C0_2$e<br>- Health building: 22.1 kgCO_2e	

For peer review only

For peer review only - http://bmjopen.bmj.com/site/about/guidelines.xhtml

FUNDED BY NIHR | National Institute for Health and Care Research   LIVERPOOL CLINICAL TRIALS CENTRE LCTC   ICR

			Choose the most suitable building type and multiply by number of years and m^2 necessary.<br><br>**Heating**<br>Carbon footprint associated with 1 m^2 for 1 year:<br>- Office: 35.69 $kgCO_2e$<br>- Laboratory: 34.4 $kgCO_2e$<br>- Warehouse: 13.3 $kgCO_2e$<br>- Health building: 41.5 $kgCO_2e$<br><br>Choose the most suitable building type and multiply by number of years and m^2 necessary.<br><br>**Electronic data and information storage:**<br>estimate 1.365 kg CO_2e per GB per year.	
10.2. Storage and destruction of biological samples	E.g. blood, tissue, urine etc	M^2 required for storage:<br><br>Duration of storage:	See section 9.3. for storage of refrigerated or frozen samples.<br><br>See section 10.1 for storage of ambient samples.	

For peer review only

FUNDED BY NIHR | National Institute for Health and Care Research LIVERPOOL CLINICAL TRIALS CENTRE LCTC ICR

10.3. Return of equipment and supplies from participating sites to CTU	E.g. wearables, unused or expired equipment and supplies	Estimated weight and distance of delivery (t.km):	See section 1.2. (freight).	

For peer review only

Carbon footprint summary

Module	$KgCO_2e$
Trial set up	
CTU emissions	
Trial staff meetings and travel	
Treatment intervention	
Data collection and exchange	
Trial supplies and equipment	
Trial specific patient assessments	
Samples	
Laboratory	
Analysis and trial close out	
**Total =**	

For peer review only - http://bmjopen.bmj.com/site/about/guidelines.xhtml

FUNDED BY NIHR | National Institute for Health and Care Research

LIVERPOOL CLINICAL TRIALS CENTRE LCTC

ICR

References

[1]What is an emission factor? [Internet]. Climfoot-project.eu. [cited 2023 May 11]. Available from: https://climfoot-project.eu/en/what-emission-factor

For all emission factor and benchmark data sources, please refer to the accompanying "Detailed Guidance and method to calculate the carbon footprint of a clinical trial."

For peer review only

For peer review only - http://bmjopen.bmj.com/site/about/guidelines.xhtml

FUNDED BY NIHR | National Institute for Health and Care Research

Enabling lower carbon clinical trials: Development and prototype testing of a method to quantify the carbon footprint of clinical trials to inform future lower carbon clinical trial design

Guidance and method to calculate the carbon footprint of a clinical trial

For peer review only

Data collation quick guide and worksheet

This guidance provides information on how to carbon footprint a clinical trial for the purposes of the NIHR-funded project 'enabling lower carbon clinical trials.'

Within the guidance, clinical trial processes have been sub-divided into the following modules:

1. Trial set up
2. CTU emissions
3. Trial specific meetings and travel
4. Treatment intervention
5. Data collection and exchange
6. Trial supplies and equipment
7. Trial specific patient assessments
8. Samples
9. Laboratory
10. Trial close out

This list is not exhaustive, and it is expected that further activities and modules may need to be added to account for specialist processes in all clinical trial types.

NB: analysis of data does not need to be calculated separately, it is covered by the emissions attributed to trial staff FTE in "CTU emissions" and calculations included within "Data Collection and exchange".

For peer review only - http://bmjopen.bmj.com/site/about/guidelines.xhtml

In addition to this quick guide and worksheet, we have produced a detailed guidance and method document defining the project scope, limitations and assumptions. The detailed guidance contains a more in depth look and explanation of the calculations found in this document, including emission factor and benchmark data sources, and should be referred to when using this worksheet.

Introduction to calculating carbon footprint

A carbon footprint is a measure of greenhouse gases, usually quoted in kg or tonnes of carbon dioxide equivalent (CO_2e). To calculate the carbon footprint of a particular clinical trial process, both ‘activity data’ and ‘emission factors’ are required.

An emission factor, also known as a conversion factor, “is a coefficient which allows you to convert activity data into greenhouse gas emissions. It is the average **emission** rate of a given source, relative to units of activity or process/processes.”[1]

The activity data is provided by the user and multiplied by the emission factors provided in this guidance document.

Data collation quick guide and worksheet

This data collation quick guide should be used in conjunction with the “Enabling lower carbon clinical trials: Development and prototype testing of a method to quantify the carbon footprint of clinical trials to inform future lower carbon clinical trial design - Detailed Guidance and method to calculate the carbon footprint of a clinical trial”. The guidance document provides the detailed explanation of how calculations should be considered and calculated. This quick guide should be used to collate the trial-specific processes, necessary activity data and to record the subsequent calculations. It is important to avoid double-counting activities i.e., modules must not include activities already covered elsewhere in the clinical trial process map. Please complete this worksheet for each trial to be carbon footprinted.

NB: We are using the term ‘CTU’ to describe the organisation that manages all aspects of central trial management. For some institutions some of those tasks maybe done by groups outside the CTU team e.g., sponsor office/CRO etc.

For peer review only - http://bmjopen.bmj.com/site/about/guidelines.xhtml

FUNDED BY NIHR | National Institute for Health and Care Research

LIVERPOOL CLINICAL TRIALS CENTRE LCTC

ICR

CASPS carbon footprint calculations

CASPS is an international phase II trial of CEDIRANIB in the treatment of patients with alveolar soft part sarcoma. The trial enrolled 47 patients across twelve sites in the UK, Spain, and Australia and involved an internationally shipped IMP, completion and shipment of paper CRF, on site visits for site initiation, monitoring visits and audits, additional hospital visits for patients for trial-specific patient assessments and provision of samples.

Module	Examples	Trial activity data	Calculation	Results
**1. Trial set-up**				
1.1. Production of trial documentation to be sent to sites or participants	E.g. Site Investigator File and contents, Site Pharmacy File and contents, CRF Folder and contents, PIS/Cs, GP letters	12 sites, 47 patients<br><br>SIF: 401 pages, 12 sent (4812 pages)<br>Site pharmacy file: 301 pages, 11 sent (3311 pages)<br>CRF folder: 76 pages in set up pack, 17 sent (1292 pages)<br>PIS/C 1 = 17 pages, 27 patients (799 pages)<br>PIS/C 2 = 16 pages, 47 patients (762)<br>Letters inc. GP = 8 pages, 47 patients (376)<br><br>Total = 11352 pages<br><br>Number of folders used to send trial documentation: estimate 23 large ring binders (0.5kg) and 18 small (0.3kg)	**Paper:**<br>[no. of page] x 0.005 = paper weight (kg)<br><br>b/w printing: Kg of paper x 0.22438 = $kgCO_2e$<br>Colour printing: Kg of paper x 0.31786 = $kgCO_2e$<br>Materials (paper): Kg of paper x 0.919 = $kgCO_2e$<br><br>Folders: Kg (of cardboard) x 0.821 = $kgCO_2e$<br>Assumption: Weight of lever arch = 0.5kg<br>Assumption: Weight of ring binder = 0.3kg<br><br>Paper:<br>11352 pages x 0.005 = 56.76kg<br>Printing: 56.76 kg x 0.22438 = 12.7 $kgCO_2e$<br>Materials: 56.76 kg x 0.919 = 52.2 $kgCO_2e$<br><br>Folders:<br>(23 x 0.5) + (18 x 0.3) = 16.9kg<br>16.9kg x 0.821 = 13.9 $kgCO_2e$	78.8 $kgCO_2e$

For peer review only - http://bmjopen.bmj.com/site/about/guidelines.xhtml

FUNDED BY NIHR | National Institute for Health and Care Research

1.2. Provision/postage of trial documentation to sites<br><br>1.3. Provision/postage of documentation to participants by CTU or participating sites<br><br>1.4. Provision/postage of incentives to participant	E.g. Site Investigator File and contents, Site Pharmacy File and contents, CRF Folder and contents, PIS/Cs, GP letters	Weight and distance of deliveries (t.km):<br>UK sites<br>- RMH - 0.00519 tonnes x 24.1km = 0.125079 t.km<br>- Christie - 0.0092 x 355.7km = 3.275997 t.km<br>- Bristol - 0.0063 tonnes x 196.3 km= 1.23669 t.km<br>- UCLH - 0.00519 tonnes x 25.7km = 0.133383 t.km<br>- Newcastle - 0.008725 tonnes x 506.9km = 4.4227 t.km<br>- Nottingham - 0.00519 tonnes x 259.1km = 1.344729 t.km<br>Australia<br>0.00587 tonnes x (17018.5km+16544km) = 197 t.km<br><br>Spain<br>0.00519 x (1147km + 1244km + 1091km) = 18.07 t.km<br><br>GP letter<br>Weight of GP letter:<br>2x5g + envelope (7g) = 17g /0.000017tonnes<br>0.000017 tonnes x 47 patients = 0.000799 tonnes	Delivery weight (tonnes) x distance (km) = t.km<br><br>For road freight: t.km x 0.19443 = $kgCO_2e$<br><br>For air freight: t.km x required emission factor below = $kgCO_2e$<br>▪ Domestic (to/from UK) = 4.98549<br>▪ Short haul (to/from UK) = 2.55439<br>▪ Long-haul (to/from UK) = 1.13047<br>▪ International (to/from non-UK) = 1.13047<br><br>UK<br>Total t.km = 10.539289 x 0.19443 = 2.05 $kgCO_2e$<br><br>Australia<br>197 t.km x 1.13047 = 222.7 $kgCO_2e$<br><br>Spain<br>18.07 t.km x 2.55439 = 46.2 $kgCO_2e$<br><br>GP letter postage:<br>For delivery of trial supplies to patients or GP, if unknown, use 17.4km as distance from hospital to patient, or hospital to GP.<br><br>0.000799 tonnes x 17.4km = 0.014 t.km<br>0.014 t.km x 0.19443 = 0.002722 $kgCO_2e$	271 $kgCO_2e$

For peer review only - http://bmjopen.bmj.com/site/about/guidelines.xhtml

FUNDED BY NIHR | National Institute for Health and Care Research — LIVERPOOL CLINICAL TRIALS CENTRE LCTC — ICR

			6 letters sent to each patient - 0.002722 x 6 = 0.016 $kgCO_2e$*	
**2. CTU emissions**				
2.1. Energy consumption at CTU according to trial staff FTE	E.g. energy consumption per square metre of air-conditioned office space	Trial duration: 6 years<br><br>Trial staff FTE: UK - 1, Spain - 0.3, Australia - 0.5	**Energy consumption for 1 FTE for 1 year** = 193.28 $kgCO_2e$<br>Multiply by the number of years and FTE applicable<br><br>UK CTU emissions = 193.28 $kgCO_2e$ x 6 = 1159.7 $kgCO_2e$<br><br>Australia CTU<br>- location: Victoria<br>- Office building energy consumption = 231.39 kwh/m^2 source: baseline-energy-consumption-part_1-report-2012.pdf. energy split is 90% electricity, 10% gas. Therefore electricity = 208.251 kwh/m^2<br>- Energy usage per staff (per 12 m^2) = 12 m^2 x 208.251 kWh =2499.012 kwh<br>- Victoria electricity emission factor = 0.98 kg CO2-e/kWh (Source: EERS release 2020–21 (cleanenergyregulator.gov.au))<br>- 2499.012 kWh x 0.98 = 2449$kgCO_2e$ per FTE per year<br>- 2449 $kgCO_2e$ x 0.5 (FTE) x 6 years = 7347$kgCO_2e$	8854.6 $kgCO_2e$

* Assumption: as the international sites are also located in cities, the same patient to GP distance was used for Australia and Spain

For peer review only - http://bmjopen.bmj.com/site/about/guidelines.xhtml

FUNDED BY NIHR | National Institute for Health and Care Research

LIVERPOOL CLINICAL TRIALS CENTRE LCTC

ICR

			Spain trial staff<br>- UK benchmark data used as a proxy for average office building energy consumption because like the UK, Spain has a reasonably contribution from renewables.<br>- 193.284 $kgCO_2e$ x 6 x 0.3 = 347.9 $kgCO_2e$	
2.2. Heating	E.g. energy consumption at coordination centre attributed to heating (natural gas)	Trial duration: 6 years<br><br>Trial staff FTE: UK - 1, Spain - 0.3, Australia - 0.5	**Heating for 1 FTE for 1 year =** 431.56 $kgCO_2e$<br>Multiply by the number of years and FTE applicable<br>UK = 431.56 $kgCO_2e$ x 6 = 2589.4$kgCO_2e$<br><br>Australia<br>- In office buildings energy split is 90% electricity, 10% gas. Office gas intensity = 23.139 kwh/m^2<br>- Energy usage per staff (per 12 m^2) = 12m^2 x 23.139 kWh = 277.668 kWh<br>- 277.668 kWh = 1GJ<br>- [Australian National Greenhouse Accounts Factors (dcceew.gov.au)] Emission factor for natural gas: Scope 1 = 51.3 $kgCO_2e$/GJ, Scope 3 = 4.0 $kgCO_2e$/GJ<br>- (1x 51.53 $kgCO_2e$/GJ) + (1x4.0 $kgCO_2e$/GJ)<br>- = 55.53 $kgCO_2e$	3532.8 $kgCO_2e$

For peer review only

For peer review only - http://bmjopen.bmj.com/site/about/guidelines.xhtml

FUNDED BY NIHR National Institute for Health and Care Research · LIVERPOOL CLINICAL TRIALS CENTRE LCTC · ICR

			- 55.53 $kgCO_2e$ x 0.5 (FTE) x 6 years = Total = 166.59 $kgCO_2e$<br>Spain<br>- UK benchmark and emission factor data used as a proxy for average office building energy consumption.<br>- 431.56 $kgCO_2e$ x 0.3 x 6 = 776.8 $kgCO_2e$	
2.3. Trial team commuting	E.g. Car, rail, bus, walking etc	Trial duration: 6 years<br><br>Trial staff FTE: UK - 1, Spain - 0.3, Australia - 0.5	For 1 FTE for 1 year, total average commuting emissions = 1027.8 $kgCO_2e$ † Multiply by the number of years and FTE applicable<br><br>UK = 1027.8 x 6 = 6166.8 $kgCO_2e$<br>Australia = 1027.8 $kgCO_2e$ x 0.5 x 6 years = 3083.4 $kgCO_2e$<br>Spain = 1027.8 $kgCO_2e$ x 0.3 x 6 years = 1850 $kgCO_2e$	11100.2 $kgCO_2e$
**3. Trial specific meetings and travel**				
3.1. Visits and travel to site<br>3.2. Travel to meetings	E.g. Feasibility, site initiation and monitoring visits, audits, inspections, Trial Management Group (TMG), Trial Steering Committee (TSC), Independent Data Monitoring Committee (IDMC),	Australia feasibility visit:<br>- Flight from Melbourne to Brisbane<br>- Estimated distance travelled = 1375 km<br>- Number of passengers = 2<br><br>Site Initiation visits:<br>Assumption: Rail used for all travel UK site visits	Number of passengers x total distance (km) = p.km<br><br>For national rail: p.km x 0.04441= $kgCO_2e$<br><br>For flights: p.km x relevant emission factor below:<br>- Domestic (average): 0.27278<br>- Short-haul (average) to/from UK: 0.17034	19433 $kgCO_2e$

† Assumption: the average commuting statistics for the UK were found to be similar to those for Spain and Australia and therefore also applied to the international CTUs.

For peer review only - http://bmjopen.bmj.com/site/about/guidelines.xhtml

FUNDED BY NIHR | National Institute for Health and Care Research

	and investigator meetings, Patient and Public Involvement and Engagement (PPIE), conferences, scientific meetings etc	UK total p.km = 3868.6 p.km<br>Australia total p.km = 9736 p.km<br>Spain: 6 teleconferences, 34 total attendees<br><br><u>Monitoring visits:</u><br>UK total p.km = 12957.4 p.km<br>Australia total p.km = 15310 p.km<br>Spain total p.km = 3280 p.km<br><br><u>Audits</u><br>Long haul to/from UK flights = 29442 p.km<br>Short haul = 13464 p.km<br>International = 11444 p.km<br><br><u>Other</u><br>- In person visit to Spanish CTU, 2 ICR-CTSU travelled to Barcelona: 2x2x(1309km) = 5236 p.km<br>- RMH lab visit, 115.8 p.km<br><br><u>Meetings</u><br>Joint IDMC/TSC: all teleconferences, 5 meetings, 3 external attendees<br>TMG: 5 meetings, all teleconferences, 16 attendees<br><br><u>Conferences:</u>	- Long-haul (average) to/from UK: 0.21423<br>- International (average) to/from non-UK: 0.20373<br><br>NB: Distances may be calculated using google maps and calculated from CTU to destination<br><br>**Videoconferencing** = 157 grams CO_2e per hour.<br><br><u>Feasibility visits</u><br>Australia:<br>- 2 x (2 x 1375) = 5500 p.km<br>- 5500 p.km x 0.20373 = 1120.5 $kgCO_2e$<br><br><u>Site initiation and pharmacy visits</u><br>UK: 3868.6 p.km x 0.0441 = 171.8 $kgCO_2e$<br>1 teleconference , 4 attendees – 0.157 $kgCO_2e$/hr x 4 = 0.628 $kgCO_2e$<br>Australia: 9736 p.km x 0.20373 = 1983.5 $kgCO_2e$<br>Spain (all teleconference) = 34 x 0.157 $kgCO_2e$/hr = 5.3 $kgCO_2e$<br><br><u>Monitoring visits</u><br>UK: 12957.4 p.km x 0.04441 = 575.4 $kgCO_2e$<br>Australia: 11074 p.km x 0.20373 = 3119.1 $kgCO_2e$<br>Spain: 3280 p.km x 0.17034 = 558.7 $kgCO_2e$<br><br><u>Audits</u>	

For peer review only - http://bmjopen.bmj.com/site/about/guidelines.xhtml

		- 2 attendees to a conference in Bristol, p.km = 785.2<br>- 1 attendee to conference in Nottingham, p.km = 518.2	Long haul to/from UK flights = 29422 p.km x 0.21423 = 6303.1 $kgCO_2e$<br>Short-haul = 13464 p.km x 0.17034 = 2293.5 $kgCO_2e$<br>International = 11444 p.km x 0.20373 = 2331.5 $kgCO_2e$<br><br>Other<br>- ICR-CTSU trial staff visit to Barcelona: 5236 p.km x 0.17034 = 891.9 $kgCO_2e$<br>- Lab visit: 115.8p.km x 0.04441 = 5.14 $kgCO_2e$<br><br>Meetings<br>IDMC/TSC: 5x3x 0.157 $kgCO_2e$/hr = 2.4 $kgCO_2e$<br>TMG: 5 x 18 x 0.157 $kgCO_2e$/hr = 12.6 $kgCO_2e$<br><br>Conferences<br>- Bristol: 785.2 p.km x0.04441 = 34.9 $kgCO_2e$<br>- Nottingham: 518.2 p.km = 23 $kgCO_2e$	
3.3. Hotel stays	E.g. monitoring visits, audits, inspections etc	UK hotel stays: 30 stays<br>Assumption: Hotel stays for all visits to Christie, Nottingham and Newcastle (same day visits to RMH, Bristol and UCLH)<br><br>Visit to Spain CTU: 1 room, 7 nights<br><br>Australia: 1 room, 3 nights	For UK: number of hotel rooms x number of nights x 13.9 = $kgCO_2e$<br><br>For UK (London) = number of rooms x number of nights x 13.8 = $kgCO_2e$.<br><br>For other countries use conversion factors from orange 'hotel stay' tab: conversion-factors-2021-full-set-advanced-users.xlsm (live.com)	675.7 $kgCO_2e$

For peer review only

FUNDED BY NIHR | National Institute for Health and Care Research

			Alternatively, you may use cost-based method:<br>£ spent x 0.358 = $kgCO_2e$<br><br>Trial calculations:<br><u>UK hotel stays for monitoring visits:</u><br>13.9 x 30 = 417 $kgCO_2e$<br><br><u>Visits to Spain CTU:</u><br>7 x 18.7 = 130.9 $kgCO_2e$<br><br><u>Australia hotel stays:</u><br>42.6 x 3 = 127.8 $kgCO_2e$	
3.4. Sustenance	E.g. meeting lunches, hotel dinners	Number of lunches or dinners:<br><br>Or<br><br>£ spent: £503.65	Meeting lunches or hotel dinners (vegetarian) = 2.6 $kgCO_2e$ per meal per person<br><br>Meeting lunches or hotel dinners (meat) = 5.92 $kgCO_2e$ per meal per person<br><br>Or cost-based approach = £ x 0.708 = $kgCO_2e$<br>£503.65 x 0.708 = 356.6 $kgCO_2e$	356.6 $kgCO_2e$
**4. Intervention[‡]**<br>**4.1. Physical (IMP)**<br>**4.2. Clinical (non-IMP)**<br>**4.3. Other (not captured above)**<br>**Please fill out the section most relevant to the intervention being investigated.**				
**4.1. Physical**				

[‡]As per assumptions detailed in the guidance, manufacture of the intervention is considered out of scope. This section defines all processes relating to providing and delivering the trial intervention that are over and above routine care.

For peer review only

For peer review only - http://bmjopen.bmj.com/site/about/guidelines.xhtml

4.1.1, 4.1.2. Movement of intervention, or materials required to deliver the intervention	E.g. movement of intervention from manufacturing site to distribution site, shipment of IMP to participating sites or direct to participant	NB: all shipping of IMP ambient<br><br>Movement of IMP from manufacturing site to QP site for QP declaration:<br>Assumption: 1 shipment<br>Assumption: 1 pill bottle contains 35 tablets - weight ~30g<br>Assumption: ~4000 IMP bottles shipped<br>Flight distance from Puerto Rico to QP site = 6697 km<br>Shipment weight = 4000x 30g = 120000g = 0.118 tonnes<br><br>Movement of IMP from QP site to distribution/packaging site:<br>Distance from QP site to distribution/packaging site = 352.4 km<br>Shipment weight = 0.118 tonnes<br>Movement of IMP from distribution/packaging site to participating sites:<br>UK deliveries: 7.7 t.km<br>Spain deliveries: 13 t.km<br>Australia deliveries: 184.6 t.km	Carry out freight calculation as described in section 1.2.<br><br>Movement of IMP from manufacturing site to Macclesfield for QP declaration:<br>▪ Long-haul (to/from UK) = 1.13047<br>▪ 0.118 tonnes x 6697km = 790.246 t.km<br>▪ 790.246 t.km x 1.13047 = 893.4 $kgCO_2e$<br><br>Movement of IMP from manufacturing site to distribution/packaging site (Fisher Clinical services):<br>▪ 0.118 tonnes x 352.4km = 41.5832 t.km<br>▪ 41.5832 t.km x 0.19443 = 8.1 $kgCO_2e$<br><br>Movement of IMP from distribution/packaging site to participating sites:<br>UK deliveries: 7.7 t.km x 0.19443 = 1.5 $kgCO_2e$<br>Spain deliveries:<br>▪ short - haul (to/from UK) = 2.55439<br>▪ 13 t.km x 2.55439 = 33.3 $kgCO_2e$<br>Australia deliveries:<br>▪ Long-haul (to/from UK) = 1.13047<br>▪ 184.6 t.km x 1.13047 = 208.7 $kgCO_2e$	1145 $kgCO_2e$
4.1.3. Materials required for the packaging and shipment of IMP	E.g. cardboard, cold storage boxes, polystyrene	Kg of cardboard/ polystyrene:	E.g. Single use sample cold storage box = 25.2 $kgCO_2e$ per box	Data for IMP shipment packaging

For peer review only - http://bmjopen.bmj.com/site/about/guidelines.xhtml

FUNDED BY NIHR | National Institute for Health and Care Research

LIVERPOOL CLINICAL TRIALS CENTRE LCTC

ICR

			E.g. Reusable sample cold storage box = 2.2 $kgCO_2e$ per box<br><br>E.g. Kg (cardboard) x 0.821 = $kgCO_2e$<br>E.g. Kg (polystyrene) x 3.778 = $kgCO_2e$	unobtainable from trial records. Item not included for CASPS.
4.1.4. Destruction of overage	E.g. incineration of IMP	Estimated weight of overage incinerated:<br>- Total bottles destroyed = 1464<br>- 1464 x 30g = 43.92 kg	Kg of waste x 2.4252 = $kgCO_2e$<br>43.92 kg x 2.4252 =106.5 $kgCO_2e$	106.5 $kgCO_2e$
**5. Data collection and exchange**<br>NB: analysis of data does not need to be calculated separately, it is covered by the emissions attributed to trial staff FTE in "CTU emissions" and calculations included within "Data Collection and exchange".				
5.1. Data collection and query exchange between CTU and sites	E.g. CRFs, EDC completion and query resolution, scans copied to CDs	CASPS hospital files<br>35 x large folders (500 pages)<br>- 500 x 35 = 17500<br>104 x small (150 pages)<br>- 104 x 150 = 15600<br>Total pages = 17500 + 15600 = 33100 pages<br><br>Scans<br>3 scans copied onto a CD per patient, 47 patients total<br>5 mins laptop time to burn CD per patient<br><br>Postage of scans:<br>UK: 0.278671 t.km	CASPS hospital files<br>33100 x 0.005kg = 165.5 kg<br>Paper (printing) = 165.5kg x 0.22438= 37 $kgCO_2e$<br>Paper (materials) = 165.5kg x 0.919 = 152.1 $kgCO_2e$<br><br>Scans<br>The carbon footprint of manufacturing a CD = 0.83 $kgCO_2e$ per CD<br>0.83 $kgCO_2e$ x 47 = 39.01 $kgCO_2e$<br><br>The carbon footprint of copying the scans on to a CD using a computer = 0.18079 $kgCO_2e$ per hour<br>5mins x 47 = 235 minutes = 3.91666667 hours	558.3 $kgCO_2e$

For peer review only - http://bmjopen.bmj.com/site/about/guidelines.xhtml

		Australia: 3.25 t.km<br>Spain: 0.28 t.km<br><br>CRFs<br>Postage of CRF booklets to sites:<br>UK sites, total t.km = 4.41 t.km<br>Australia sites, total t.km = 102.2 t.km<br>Spain sites, total t.km= 8.58963 t.km<br><br>Postage of CRFs from sites to CTU:<br>UK sites, total t.km = 6.37<br>Australia sites, total t.km = 132.9<br>Spain sites, total t.km = 13.8	0.18079 kgCO_2e x 3.91666667 hours = 0.71 kgCO_2e<br><br>Postage of scans:<br>UK: 0.26115 t.km x 0.19443 = 0.05 kgCO_2e<br>Australia: 3.25 t.km x 1.13047 = 3.7 kgCO_2e<br>Spain: 0.28 t.km x 2.55439 = 0.72 kgCO_2e<br><br>CRFs<br>Postage of CRF booklets to sites:<br>UK: 4.41 t.km x 0.19443 = 0.86 kgCO_2e<br>Australia: 102.2 t.km x 1.13047 = 115.5 kgCO_2e<br>Spain: 8.58963 t.km x 2.55439 = 21.9 kgCO_2e<br><br>Postage of CRFs from sites to CTU:<br>UK: 6.37 t.km x 0.19443 = 1.2 kgCO_2e<br>Australia: 132.9 t.km x 1.13047 = 150.2 kgCO_2e<br>Spain: 13.8 t.km x 2.55439 = 35.3 kgCO_2e	
5.2. Data sent direct from participants to CTU or participating sites	E.g. Questionnaires, patient diaries, wearables	Estimated weight and distance of delivery (t.km):<br><br>Device used and time taken to complete electronic questionnaires:	For paper questionnaires, please refer to section 1.1. for the carbon footprint of producing the materials and section 1.2. for postage (freight).<br><br>For use of smart watches and other devices see section 6.3.<br><br>**Electronic questionnaires**<br>Add the emissions attributed to data storage and transmission to the emissions attributed to using a device to complete the questionnaire.	N/A

For peer review only

For peer review only - http://bmjopen.bmj.com/site/about/guidelines.xhtml

FUNDED BY NIHR | National Institute for Health and Care Research · LIVERPOOL CLINICAL TRIALS CENTRE LCTC · ICR

			Web surfing (data storage and transmission) = 9.441 g CO2e/hr (10 mins = 1.57 g CO_2e)<br><br>Choose from the below:<br>- Desktop computer = 0.18079 kg CO_2e per hour<br>- Laptop = 0.028719 kg CO_2e per hour<br>- Tablet = 0.027397 kg CO_2e per hour<br>- Smartphone = 0.015068 kg CO_2e per hour	
5.3. Data from labs to CTU<br>5.4. Data from other collaborators to CTU	E.g. Laboratory patient results, data linkage	GB required for data storage/transmission:<br>Duration of data storage:<br><br>Total £ spent on computer services:	Data storage and transmission = estimate 1.365 kg CO_2e per GB per year.<br><br>For computer services such as data linkage:<br>£ x 0.149 = $kgCO_2e$	N/A
**6. Trial supplies and equipment**				
6.1. Equipment used by CTU	E.g. computers, laptops, printers, software	Total £ spent on office machinery and computers for trial: £1350	For any new office machinery and computers purchased specifically for trial:<br>£ x 0.387 = $kgCO_2e$<br><br>£1350 x 0.387 = 522.45 $kgCO_2e$	522.45 $kgCO_2e$
6.2. Equipment and supplies used by participating sites supplied by CTU	E.g. centrifuge, fridge, freezer	Estimated weight and distance of deliveries (t.km):	For the shipment of equipment to participating sites, please refer to section 1.2.<br><br>For the use of a centrifuge, please refer to section 9.2., for a fridge or freezer please refer to section 9.3.	N/A

For peer review only - http://bmjopen.bmj.com/site/about/guidelines.xhtml

FUNDED BY NIHR | National Institute for Health and Care Research

6.3. Equipment and supplies provided to participants specifically for the trial	E.g. wearables, smartphone, tablet	Number of devices and duration of their usage:<br>Estimated weight and distance of deliveries (t.km):	**Smartphone =** 55 kgCO_2e from manufacture and add 5.5 kgCO_2e per year of usage.<br>**Tablet =** 119 kgCO_2e from manufacture and add 10 kgCO_2e per year of usage.<br><br>**Wearables/smart watch** = 30.1 kg CO_2e for manufacture and add 1.633 kg CO_2e per year of usage.<br><br>**To calculate the carbon footprint associated with shipment of the devices, please refer to section 1.2.**	N/A
**7. Trial specific patient assessments**				
7.1. Patient travel for study visits that are in addition to standard of care	E.g. Eligibility and screening assessments, trial-specific assessments and procedures	Number of patients: 47<br>On average patients were on trial 72 weeks.<br><br>Number of times patient were required to travel (in addition to standard of care): 880 total patient visits (496 total visits by patients receiving IMP, 384 total visits by patients receiving placebo)	Emissions associated with one patient visit to hospital (UK) = 5.8 kgCO_2e (this includes both the out and back journeys)<br><br>Emissions associated with one patient visit to GP surgery (UK) = 1.12 kgCO_2e (this includes both the out and back journeys)<br><br>5.8 kgCO_2e x 880 = 5104 kgCO_2e	5104 kgCO_2e
7.2. Materials and activities required for study assessments that are in addition to standard of care	E.g. Laboratory tests, imaging assessments, clinical activities relating to intervention for	On average patients were on trial 72 weeks. Within that time:<br>- CT scans per patient<br>- 754 total visits requiring consumables	Consumables = 0.30 kgCO_2e per patient per trial appointment where consumables (such as gloves) required<br><br>▪ 1 MRI = 24.7 kg CO_2e<br>▪ 1 CT scan = 9.2 kgCO_2e	4117.8 kgCO_2e

For peer review only - http://bmjopen.bmj.com/site/about/guidelines.xhtml

FUNDED BY NIHR | National Institute for Health and Care Research

	example administering of study drug, biopsy.		▪ 1 hour in surgery = 53 kg CO_2e<br>▪ 1 low intensity (general ward) bed day = 37.9 kg CO_2e<br>▪ 1 high intensity (ICU) bed day = 103 kgCO_2e<br>▪ 15 sessions of breast radiotherapy = 5.7 kg CO_2e<br>▪ 20 sessions of prostate radiotherapy = 15.3 kg CO_2e<br><br>Blood tests:<br>▪ 82 g CO_2e for coagulation profile<br>▪ 116 g CO_2e for full blood examination<br>▪ 49 g CO_2e for arterial gas assessment<br>▪ 99 g CO_2e for urea and electrolyte assessment<br>▪ 0.5 g CO_2e for C-reactive protein<br><br>**Please note that the above figures for blood tests include the materials and consumables required for sample collection, phlebotomy and analysis, as well as power consumption by pathology analysers.**<br><br>**Trial calculations:**<br><br>CT scans: 47 x 9 x 9.2 kgCO_2e = 3891.6 kgCO_2e<br>Consumables: 0.30 kgCO_2e x 754 = 226.2 kgCO_2e	
7.3. Utilities required for study assessments	E.g. energy consumption per	Trial staff FTE (FTEs added up over whole trial duration):	1 FTE 1 year = 364.9 kgCO_2e	2076.6 kgCO_2e

For peer review only - http://bmjopen.bmj.com/site/about/guidelines.xhtml

FUNDED BY NIHR | National Institute for Health and Care Research

LIVERPOOL CLINICAL TRIALS CENTRE LCTC

ICR

that are in addition to standard of care	square metre of hospital space according to trial staff FTE, taking into account time required for CRF completion and study assessments, consent etc	UK – 0.85<br>Spain – 0.25<br>Australia – 0.23<br>Australia benchmark and emission factor data:<br>- Australian average hospital energy intensity = 1676 MJ = 465.55 kWh/m2.<br>- Energy intensity per employee 16.5m^2 x 465.55 = 7681.6 kWh<br>-50:50 Electricity/gas split, therefore 3840.8 kWh electricity, 3840.8 kWh natural gas<br>- Australia hospital sites located in NSW and QLD where electricity emission factor is 0.81 kgCO_2e/kWh.<br>- Australian National Greenhouse Accounts Factors (dcceew.gov.au)<br>Emission factor for natural gas: Scope 1 = 51.3 kgCO_2e/GJ, scope 3 = 13.1 kgCO_2e/GJ	Multiply by the number of years and FTE applicable<br><br>UK: 364.9 kgCO_2e x 0.85 = 310.2 kgCO_2e<br>Spain: 364.9 kgCO_2e x 0.25 = 91.2 kgCO_2e<br>Australia: 3840.8 kWh x 0.81 x 0.23 (FTE) = 715.5 kgCO_2e<br><br>Heating: 1 FTE, 1 year = 685.7 kgCO_2e<br>Multiply by the number of years and FTE applicable<br>UK: 685.7 kgCO_2e x 0.85 = 582.8 kgCO_2e<br>Spain: 685.7 kgCO_2e x 0.25 = 171.4kgCO_2e<br>Australia: 3840.8 kWh = 13.82688 GJ<br>- (13.82688 GJ x 51.53 kgCO_2e/GJ) + (13.82688 GJ x 13.1 kgCO_2e/GJ)<br>- = 893.6 kgCO_2e<br>- x 0.23 (FTE) = 205.5 kgCO_2e	
**8. Samples**				
8.1. Materials involved	E.g. sample collection kit and packaging for shipment	Kg of material:<br>- Safety kit (average plastic): 3kg<br>- Cryotubes (PP): 6.88kg<br>- Falcon tubes (PP): 6.88kg<br>- PAXgene tube (PET): 6kg<br>- Sea airbags (PET): 1.31kg<br>- Labels (PET): 1.6kg	The emissions attributed to sample collection consumables for common blood tests are included in the blood tests listed in section 7.2.<br><br>To calculate the carbon footprint of other common materials, multiply the weight in kg by the relevant emission factor below to produce kgCO_2e.	989.2 kgCO_2e

For peer review only

For peer review only - http://bmjopen.bmj.com/site/about/guidelines.xhtml

FUNDED BY NIHR | National Institute for Health and Care Research
LIVERPOOL CLINICAL TRIALS CENTRE LCTC
ICR

		- CTAD tubes (glass): 3.6335 kg<br>- CPT (glass): 3.2kg<br>- Jiffy bags (paper): 6kg<br>- Paper: 1.53kg<br>- Dry ice: 85kg<br>- Sample cold storage box: 29 single use boxes	- Average plastics: 3.116<br>- Plastics (average film): 2.754<br>- Plastics (Average rigid): 3.277<br>- Plastics (PP): 3.105<br>- Plastics (PET): 4.032<br>- Glass: 1.403<br>- Paper: 0.919<br>- Board: 0.821<br>- Dry ice: **1.81kg CO_2e for 1 kg dry ice** produced/used<br>- Sample cold storage box (SU): 25.2 kgCO_2e per cold storage box<br><br>Safety kit: 3kg x 3.116 = 9.35 kgCO_2e<br>Cryotubes + falcon tubes: 13.76kg x 3.105 = 42.7 kgCO_2e<br>PAXgene, sea airbags and labels: 8.9 1kg x 4.032 = 35.9 kgCO_2e<br>CTAD+CPT: 6.8225 kg x 3.413 = 9.57 kgCO_2e<br>Jiffy bags: 6kg x 0.881 (paper and board mixed) = 5.29 kgCO_2e<br>Paper: 1.53kg x 0.919 = 1.4 kgCO_2e<br>Paper (printing): 1.53kg x 0.22438 = 0.34 kgCO_2e<br>Dry ice: 85kg x 1.81 = 153.85 kgCO_2e<br>Sample boxes: 29 boxes x 25.2 kgCO_2e = 730.8 kgCO_2e	
8.2. Movement of sample kit materials from manufacturer to CTU	E.g. shipment of blood tubes for sample kits to CTU	8.2. Movement of sample kit materials from manufacturer to CTU	Please refer to section 1.2. for freight and 4.1. for refrigerated or frozen freight.	941.3 kgCO_2e

For peer review only

FUNDED BY NIHR | National Institute for Health and Care Research | LIVERPOOL CLINICAL TRIALS CENTRE LCTC | ICR

8.3. Movement of sample kits from CTU/distributor to participating Sites<br>8.4. Movement of samples from participating sites or patients to central laboratory.		- T.km = 11.9<br>8.3. Movement of sample kits from CTU/distributor to participating Sites:<br>▪ UK sites, total t.km = 11.9 t.km<br>▪ Australia, total t.km = 19.8528 t.km<br>▪ Spain, total t.km = 10.2 t.km<br>8.4. Movement of samples from participating sites to laboratory.<br>▪ UK sites, total t.km = 32.7 t.km<br>▪ Australia, total t.km = 663.65 t.km<br>▪ Spain, total t.km = 51.486 t.km<br>Movement of samples between laboratories:<br>- 0.8676 t.km	Movement of sample kit materials from manufacturer to CTU:<br>- 11.9 t.km X 0.19443 = 2.31 kgCO_2e<br><br>Movement of sample kits from CTU/distributor to participating Sites:<br>▪ UK sites:11.9 t.km x 0.19443 = 2.3 kgCO_2e<br>▪ Australia: 19.8528 t.km x 1.13047 = 22.4 kgCO_2e<br>▪ Spain: 10.2 t.km x 2.55439 = 26 kgCO_2e<br><br>8.4. Movement of samples from participating sites to laboratory.<br>- UK sites: 32.7 t.km x 0.19443 = 6.4 kgCO_2e<br>- Australia: 663.65 t.km x 1.13047 = 750.2 kgCO_2e<br>- Spain: 51.486 t.km x 2.55439 = 131.5 kgCO_2e<br><br>Movement of samples between laboratories:<br>- 0.8676 t.km x 0.19443 = 0.17 kgCO_2e	
**9. Laboratory**				
9.1. Emissions attributed to lab utilities according to staff FTE	E.g. energy consumption per square metre of laboratory space according to trial staff FTE	Trial duration:<br><br>Trial staff FTE:	**Electricity:**<br>1747.2 kgCO_2e per FTE per year<br>Multiply 1747.2 kgCO_2e by the number of years and FTE applicable<br><br>**Heating:**<br>1376 kgCO_2e per FTE per year	N/A - No central labs within the scope of this trial. This module refers to the

For peer review only

FUNDED BY NIHR | National Institute for Health and Care Research

LIVERPOOL CLINICAL TRIALS CENTRE LCTC

ICR

			Multiply 1376 $kgCO_2e$ by the number of years and FTE applicable	site laboratories where initial processing of samples and sample storage occurs.
9.2. Materials/equipment /consumables used in processing and analysis of samples	E.g. centrifuges, refrigerators	Centrifuges required for processing the following samples:<br><br>CTAD samples<br>- Samples centrifuged for 15 mins (0.25 hours)<br>- 310 watts<br>- 310 x 0.25 = 77.5 kWh<br>- 313 samples<br><br>CPT samples<br>- Samples centrifuged for 45 mins (0.583 hours)<br>- 310 Watts<br>- 310 x 0.583 = 180.73 kWh<br>- 261 samples	To avoid double counting, use of equipment will be included in lab staff FTE if calculated.<br><br>If the trial does not involve a central lab, but there is still sample processing on site, please see below. For storage of samples, please see section 9.3.<br><br>To calculate the emissions of a piece of equipment, multiply the power consumption by hours used to get a kWh value. Finally multiply kWh by the electricity emission factor (0.273).<br><br>Consider the centrifuge capacity and multiply by the number of uses required.<br><br>Centrifuge – CTAD Samples:<br>- 77.5 kWh x 0.273 = 21.2 $kgCO_2e$ / 24 = 0.881$kgCO_2e$ per sample (Assumption: centrifuge capacity is 24 samples)<br>- 0.881 x 313 = 275.9 $kgCO_2e$<br><br>Centrifuge – CPT samples:	812.5 $kgCO_2e$

For peer review only - http://bmjopen.bmj.com/site/about/guidelines.xhtml

FUNDED BY NIHR | National Institute for Health and Care Research

			- 180.73 kWh x 0.273 = 49.33929 $kgCO_2e$ / 24 = 2.1 $kgCO_2e$ per sample (Assumption: centrifuge capacity is 24 samples)<br>- 2.1 x 261 samples = 536.6 $kgCO_2e$<br><br>Total = 275.9 kgCO2e + 536.6 $kgCO_2e$ = 812.5 $kgCO_2e$	
9.3. Storage of samples	E.g. utilities and ultra-low temperature freezer	Storage of blood samples in fridge/freezer:<br>Length of storage = 10 years<br>Amount of refrigerator/freezer space required = 1/5 of a fridge, 1/3 of a freezer<br><br>Storage of ambient tissue:<br>- 10 years, 2011-2021<br>- Assumption: tissue samples occupy $1m^2$	Storage in fridge/-20 freezer:<br>- 298.9 $kgCO_2e$ per year<br>- Multiply by number of years stored<br>Storage in an ultra-low/-80 freezer:<br>- 2192.2 $kgCO_2e$ per year<br>- Multiply by number of years stored<br><br>NB: this is for a whole freezer; you will need to make an assumption about the amount of space in the freezer that the trial samples take up.<br><br>Fridge/-20:<br>298.9 $kgCO_2e$ x 10 years = 2989 $kgCO_2e$<br>2989 $kgCO_2e$ x 0.20 = 597.8 $kgCO_2e$<br><br>Storage in an ultra-low/-80 freezer:<br>2192.2 $kgCO_2e$ per year x 10 = 21922 $kgCO_2e$<br>21922 $kgCO_2e$ x 0.333 = 7307.3 $kgCO_2e$<br><br>Ambient storage of tissue:<br>Electricity:<br>- Health building = 22.1 $kgCO_2e/ m^2$<br>- 22.1 $kgCO_2e/ m^2$ x 10 = 221 $kgCO_2e$	8541.1 $kgCO_2e$

For peer review only

FUNDED BY NIHR | National Institute for Health and Care Research

LIVERPOOL CLINICAL TRIALS CENTRE LCTC

ICR

			Heating:<br>41.5 $kgCO_2e$ x 10 = 415 $kgCO_2e$	
**10. Trial close out**				
10.1. Storage and archiving of essential trial documentation and data	E.g. Hospital files, lab files, trial guidance documents etc	Duration of storage: 10 years<br><br>Amount of space required for storage (m^2): Assumption: archiving documents requires $4m^2$<br><br>Storage location: CTU (office)	Carbon footprint associated with $1m^2$ for 1 year:<br>- Office: 16.1 $kgCO_2e$<br>- Laboratory: 43.7 $kgCO_2e$<br>- Warehouse: 7.4 $kgCO_2e$<br>- Health building: 22.1 $kgCO_2e$<br><br>Choose the most suitable building type and multiply by number of years and m^2 necessary.<br><br>16.1 $kgC0_2e$ x 4 x 10 = 644 $kgCO_2e$<br><br>**Heating**<br>Carbon footprint associated with 1 m^2 for 1 year:<br>- Office: 35.69 $kgCO_2e$<br>- Laboratory: 34.4 $kgCO_2e$<br>- Warehouse: 13.3 $kgCO_2e$<br>- Health building: 41.5 $kgCO_2e$<br><br>Choose the most suitable building type and multiply by number of years and m^2 necessary.<br><br>35.69 $kgCO_2e$ x 4 x 10 = 1427.6 $kgCO_2e$	2085.3 $kgCO_2e$

For peer review only

FUNDED BY NIHR | National Institute for Health and Care Research
LIVERPOOL CLINICAL TRIALS CENTRE LCTC
ICR

			**For electronic storage,** estimate 1.35 kgCO_2e per GB per year.<br><br>Size of CASPS on server = ~1GB<br>1.365 kgCO_2e x 10 = 13.65 kgCO_2e	
10.2. Storage and destruction of biological samples	E.g. blood, tissue, urine etc	Duration of storage: 10 years<br><br>Amount of space required for storage (m^2): 1<br><br>Storage location: hospital/health building	See section 9.3. for storage of refrigerated or frozen samples.<br><br>See section 10.1 for storage of ambient samples.<br><br>Health building<br>Electricity: 22.1 kgCO_2e x 10 = 221 kgCO_2e<br>Heating: 41.5 kgCO_2e x 10 = 415 kgCO_2e	636 kgCO_2e
10.3. Return of equipment and supplies from participating sites to CTU	E.g. wearables, unused or expired equipment and supplies	Estimated weight and distance of delivery (t.km):	See section 1.2. (freight).	N/A

For peer review only

FUNDED BY NIHR | National Institute for Health and Care Research
LIVERPOOL CLINICAL TRIALS CENTRE LCTC
ICR

CASPS carbon footprint summary

Module	$KgCO_2e$
Trial set up	349.8
CTU emissions	23487.6
Trial staff meetings and travel	20465.3
Treatment intervention	1251.5
Data collection and exchange	558.3
Trial supplies and equipment	522.5
Trial specific patient assessments	11298.4
Samples	1930.5
Laboratory	9353.6
Analysis and trial close out	2721.3
**Total =**	71938.8 $kgCO_2e$ / 71.9 $tonnesCO_2e$

References

[1] What is an emission factor? [Internet]. Climfoot-project.eu. [cited 2023 May 11]. Available from: https://climfoot-project.eu/en/what-emission-factor

For all emission factor and benchmark data sources, please refer to the accompanying "Detailed Guidance and method to calculate the carbon footprint of a clinical trial."

For peer review only - http://bmjopen.bmj.com/site/about/guidelines.xhtml

For peer review only

FUNDED BY NIHR | National Institute for Health and Care Research

Enabling lower carbon clinical trials: Development and prototype testing of a method to quantify the carbon footprint of clinical trials to inform future lower carbon clinical trial design

Guidance and method to calculate the carbon footprint of a clinical trial

For peer review only

Data collation quick guide and worksheet

This guidance provides information on how to carbon footprint a clinical trial for the purposes of the NIHR-funded project ‘enabling lower carbon clinical trials.’

Within the guidance, clinical trial processes have been sub-divided into the following modules:

1. Trial set up
2. CTU emissions
3. Trial specific meetings and travel
4. Treatment intervention
5. Data collection and exchange
6. Trial supplies and equipment
7. Trial specific patient assessments
8. Samples
9. Laboratory
10. Trial close out

This list is not exhaustive, and it is expected that further activities and modules may need to be added to account for specialist processes in all clinical trial types.

NB: analysis of data does not need to be calculated separately, it is covered by the emissions attributed to trial staff FTE in “CTU emissions” and calculations included within “Data Collection and exchange”.

For peer review only - http://bmjopen.bmj.com/site/about/guidelines.xhtml

In addition to this quick guide and worksheet, we have produced a detailed guidance and method document defining the project scope, limitations and assumptions. The detailed guidance contains a more in depth look and explanation of the calculations found in this document, including emission factor and benchmark data sources, and should be referred to when using this worksheet.

Introduction to calculating carbon footprint

A carbon footprint is a measure of greenhouse gases, usually quoted in kg or tonnes of carbon dioxide equivalent (CO_2e). To calculate the carbon footprint of a particular clinical trial process, both 'activity data' and 'emission factors' are required.

An emission factor, also known as a conversion factor, "is a coefficient which allows you to convert activity data into greenhouse gas emissions. It is the average **emission** rate of a given source, relative to units of activity or process/processes."[1]

The activity data is provided by the user and multiplied by the emission factors provided in this guidance document.

Data collation quick guide and worksheet

This data collation quick guide should be used in conjunction with the "Enabling lower carbon clinical trials: Development and prototype testing of a method to quantify the carbon footprint of clinical trials to inform future lower carbon clinical trial design - Detailed Guidance and method to calculate the carbon footprint of a clinical trial". The guidance document provides the detailed explanation of how calculations should be considered and calculated. This quick guide should be used to collate the trial-specific processes, necessary activity data and to record the subsequent calculations. It is important to avoid double-counting activities i.e., modules must not include activities already covered elsewhere in the clinical trial process map. Please complete this worksheet for each trial to be carbon footprinted.

NB: We are using the term 'CTU' to describe the organisation that manages all aspects of central trial management. For some institutions some of those tasks maybe done by groups outside the CTU team e.g., sponsor office/CRO etc.

For peer review only - http://bmjopen.bmj.com/site/about/guidelines.xhtml

FUNDED BY NIHR | National Institute for Health and Care Research — LIVERPOOL CLINICAL TRIALS CENTRE LCTC — ICR

PRIMETIME carbon footprint calculations

PRIMETIME is a post-operative avoidance of radiotherapy trial which recruited 1962 patients across 64 sites in the UK. The trial used electronic data capture for collection of data, data linkage with NHS routine data sources and involved shipment of tissue samples for immunohistochemistry testing and subsequent long-term sample storage.

Module	Examples	Trial activity data	Calculation	Results
**1. Trial set-up**				
1.1. Production of trial documentation to be sent to sites or participants	E.g. Site Investigator File and contents, Site Pharmacy File and contents, CRF Folder and contents, PIS/Cs, GP letters	67 sites initially set up (64 recruited patients)<br><br>3123 patients registered to pre-screening, 1962 patients consented and registered to main study<br><br>SIF: 260 pages, 67 sent (17420 total pages)<br>GP letter: 2 pages, 1962 (3924 total pages)<br>PIS/C form 1: 6 pages, 3123 patients (18738 total pages)<br>PIS/C form 2: 15 pages, 1962 (29430 total pages)<br><br>Number of folders used to send trial documentation = 67 large ring binders (0.5kg)	**Paper:**<br>[no. of page] x 0.005 = paper weight (kg)<br><br>b/w printing: Kg of paper x 0.22438 = $kgCO_2e$<br>Colour printing: Kg of paper x 0.31786 = $kgCO_2e$<br>Materials (paper): Kg of paper x 0.919 = $kgCO_2e$<br><br>Folders: Kg (of cardboard) x 0.821 = $kgCO_2e$<br>Assumption: Weight of lever arch = 0.5kg<br>Assumption: Weight of ring binder = 0.3kg<br><br>SIF:<br>17420 pages x 0.005= 87.1 kg<br>Printing: 87.1 kg x 0.22438 = 19.5 $KgCO_2e$<br>Materials (paper): 87.1 kg x 0.919 = 80 $kgCO_2e$<br><br>Materials (folder): 0.5 kg x 67= 33.5kg<br>33.5 kg x 0.821 = 27.5 $kgCO_2e$<br><br>PIS/C:<br>48168 x 0.005 kg = 240.8 kg<br>Printing: 240.8 kg x 0.31786 = 76.5 $kgCO_2e$<br>Materials: 240.8 kg x 0.919 = 221.3 $kgCO_2e$	447.2 kg CO_2e

For peer review only - http://bmjopen.bmj.com/site/about/guidelines.xhtml

FUNDED BY NIHR | National Institute for Health and Care Research
LIVERPOOL CLINICAL TRIALS CENTRE LCTC
ICR

			GP letter:<br>3924 x 0.005 kg = 19.6 kg<br>Printing: 19.6 kg x 0.22438 = 4.4 $kgCO_2e$<br>Materials: 19.6 kg x 0.919 = 18 $kgCO_2e$	
1.2. Provision/postage of trial documentation to sites<br><br>1.3. Provision/postage of documentation to participants by CTU or participating sites<br><br>1.4. Provision/postage of incentives to participant	E.g. Site Investigator File and contents, Site Pharmacy File and contents, CRF Folder and contents, PIS/Cs, GP letters	Estimated weight of SIF:<br>(260 pages x 0.005kg) + 0.5 kg = 1.8kg / 0.00177 tonnes<br><br>Estimated distance of deliveries:<br>Average site distance from ICR-CTSU is 301.8 km<br><br>Number of deliveries: SIF delivered to 67 sites	Delivery weight (tonnes) x distance (km) = t.km<br><br>For road freight: t.km x 0.19443 = $kgCO_2e$<br><br>For air freight: t.km x required emission factor below = $kgCO_2e$<br><br>- Domestic (to/from UK) = 4.98549<br>- Short-haul (to/from UK) = 2.55439<br>- Long-haul (to/from UK) = 1.13047<br>- International (to/from non-UK) = 1.13047<br><br>For delivery of trial supplies to patients or GP, if unknown, use 17.4km as distance from hospital to patient, or hospital to GP.<br><br>Calculations:<br>SIF delivery:<br>- 0.00177 x 301.8km = 0.53 t.km<br>- 0.53 x 0.19443 = 0.103<br>- x 67 sites = 6.9 $kgCO_2e$<br><br>GP letter:<br>Weight of letter = 2x5g + envelope (7g)= 17g	7.01 $kgCO_2e$

For peer review only - http://bmjopen.bmj.com/site/about/guidelines.xhtml

FUNDED BY NIHR | National Institute for Health and Care Research — LIVERPOOL CLINICAL TRIALS CENTRE LCTC — ICR

			x1962 patients = 33354g/0.033 tonnes<br>0.033 x 17.4km = 0.5742 t.km<br>0.5742 t.km x 0.19443 = 0.11 $kgCO_2e$	
**2. CTU emissions**				
2.1. Energy consumption at CTU according to trial staff FTE	E.g. energy consumption per square metre of air-conditioned office space	Trial duration: 10 years<br><br>Trial staff FTE: the total FTE over the 10-year trial period was 7.1	**Energy consumption for 1 FTE for 1 year** = 193.28 $kgCO_2e$<br><br>Multiply by the number of years and FTE applicable<br><br>7.1 X 193.28 $kgCO_2e$ = 1372.3 $kgCO_2e$	1372.3 $kgCO_2e$
2.2. Heating	E.g. energy consumption at coordination centre attributed to heating (natural gas)	Trial duration: 10 years<br><br>Trial staff FTE: the total FTE over the 10-year trial period was 7.1	**Heating for 1 FTE for 1 year =** 431.56 $kgCO_2e$<br><br>Multiply by the number of years and FTE applicable<br><br>7.1 x 431.56 $kgCO_2e$ = 3064 $kgCO_2e$	3064 $kgCO_2e$
2.3. Trial team commuting	E.g. Car, rail, bus, walking etc	Trial duration: 10 years<br><br>Trial staff FTE: the total FTE over the 10-year trial period was 7.1	For 1 FTE for 1 year, total average commuting emissions = 1027.8 $kgCO_2e$<br><br>Multiply by the number of years and FTE applicable<br><br>7.1 x 1027.8 $kgCO_2e$ = 7297 $kgCO_2e$	7297 $kgCO_2e$
**3. Trial specific meetings and travel**				
3.1. Visits and travel to site<br>3.2. Travel to meetings	E.g. Feasibility, site initiation and monitoring visits,	No feasibility visits, monitoring, audits, or conferences.	Number of passengers x total distance (km) = p.km	579.3 $kgCO_2e$

For peer review only

For peer review only - http://bmjopen.bmj.com/site/about/guidelines.xhtml

FUNDED BY NIHR | National Institute for Health and Care Research

	audits, inspections, Trial Management Group (TMG), Trial Steering Committee (TSC), Independent Data Monitoring Committee (IDMC), and investigator meetings, Patient and Public Involvement and Engagement (PPIE), conferences, scientific meetings etc	<u>Site initiation visits</u><br>66 virtual (+6 refresher sessions), 1 in person<br><br><u>IDMC meetings</u><br>5 IDMC meetings, all virtual, total of 31 instances of attendance<br><br><u>TMG meetings</u><br>Total distances travelled by participants or number of virtual attendees:<br>- Meeting 1- 1760.6km<br>- Meeting 2- 1437.3km<br>- Meeting 3 – virtual, 12 attendees<br>- Meeting 4 – 1624.8km<br>- Meeting 5 – virtual, 25 attendees<br>- Meeting 6 – virtual, 18 attendees<br>- Meeting 7 – virtual, 23 attendees<br><br><u>TSC meetings</u><br>5 attendees to each TSC meeting, 1 hour duration, some teleconference and some in person. Distance staff travelled from CTU = 22.5km	For national rail: p.km x 0.04441= $kgCO_2e$<br><br>For flights: p.km x relevant emission factor below:<br>- Domestic (average): 0.27278<br>- Short-haul (average) to/from UK: 0.17034<br>- Long-haul (average) to/from UK: 0.21423<br>- International (average) to/from non-UK: 0.20373<br><br>NB: Distances may be calculated using google maps and calculated from CTU to destination<br><br>**Videoconferencing** = 157 grams CO_2e per hour.<br><br>Calculations:<br><u>Site Initiation Visits:</u><br>Virtual SIVs: 72 X 157 g CO2e/hr = 11304g/11.3 $kgCO_2e$<br><br>1 in person SIV: 1 attendee, 24.1KM.<br>1 x 2 x 24.1 km = 48.2 p.km<br>48.2 p.km x 0.04441= 2.1 $kgCO_2e$<br><br><u>IDMC</u><br>IDMC: 31 x 157 g CO2e/hr = 4867 g/4.9 $kgCO_2e$<br><br><u>TMG meetings</u> - assumption: rail used to travel	

For peer review only - http://bmjopen.bmj.com/site/about/guidelines.xhtml

			TMG meeting 1: 1x2x1760.6km = 3521.2 p.km.<br>3521.2 p.km x 0.04441 = 156.4 $kgCO_2e$<br>TMG meeting 2: 1x2x1437.3km= 2874.6 p.km<br>2874.6 p.km x 0.04441 = 127.7 $kgCO_2e$<br>TMG meeting 3: teleconference, 2 hours, 12 external attendees. 157 g CO2e/hr x 2 x 12 = 3768g/3.77 $kgCO_2e$<br>TMG meeting 4: 1 x 2 x 1624.8km =<br>3249.6 p.km x 0.04441 = 144.3 $kgCO_2e$<br>TMG meeting 5: 13-14:30 via zoom, 25 attendees. 157 g CO2e/hr x 1.5 x 25 = 5887.5 g/5.89 $kgCO_2e$<br>TMG meeting 6: 157 g CO2e/hr x 1.5 x 18 = 4239 g/4.24 $kgCO_2e$<br>TMG meeting 7: 1hr, Teams meeting, 23 attendees - 157 g CO2e/hr x 23 = 3611g/3.6 $kgCO_2e$<br><br>TSC<br>2016, teleconference: 157 g CO2e/hr x 5 = 785g/0.8 $kgCO_2e$<br>2017: 5x(2x22.5km) = 225 p.km x 0.04441= 10 $kgCO_2e$<br>2018 = 10 $kgCO_2e$<br>2019 = 10 $kgCO_2e$<br>2021 = 10 $kgCO_2e$<br><br>Other:<br>➢ 2 virtual conferences: 157g CO2e/hr x 2 = 0.314 $kgCO_2e$	

For peer review only

For peer review only - http://bmjopen.bmj.com/site/about/guidelines.xhtml

FUNDED BY NIHR | National Institute for Health and Care Research

LIVERPOOL CLINICAL TRIALS CENTRE LCTC

ICR

			➢ Public Health England (PHE) teleconference, 2 attendees: 2 x 157gCO_2e/hr = 0.314 kgCO_2e<br>➢ Sample management and logistics planning meeting, 5 attendees: 5 x 157 g CO2e/hr = 0.785 kgCO_2e<br>➢ Data management meetings, 20 instances of teleconferencing: 20 x 157 g CO2e/hr = 3140g/3.14 kgCO_2e<br>➢ LCRN Meeting re CPMS Uploads, 9 attendees - 9 x 157 g CO2e/hr = 1.413 kgCO_2e<br>➢ Primetime Team meetings, 132 instances of people teleconferencing for 1 hour. 132 x 157 = 20724 g/20.7 kgCO_2e<br>➢ CI_TC updates, 303 instances of people teleconferencing for 1 hour: 303 x 157 g CO2e/hr = 47571 g/47.6 kgCO_2e	
3.3. Hotel stays	E.g. TMG meetings, monitoring visits, audits, inspections etc	Number of rooms: 8 rooms (for one person for one night)	For UK: number of hotel rooms x number of nights x 13.9 = kgCO_2e<br><br>For UK (London) = number of rooms x number of nights x 13.8 = kgCO_2e.<br><br>For other countries use conversion factors from orange 'hotel stay' tab: conversion-factors-2021-full-set-advanced-users.xlsm (live.com)<br>Alternatively, you may use cost-based method: £ spent x 0.388 = kgCO_2e	250.2 kgCO_2e

For peer review only

FUNDED BY NIHR | National Institute for Health and Care Research
LIVERPOOL CLINICAL TRIALS CENTRE LCTC
ICR

			Assumption: 8 stays (for TMG meetings)<br>8 x 13.9 = 250.2 $kgCO_2e$	
3.4. Sustenance	E.g. meeting lunches, hotel dinners	Number of lunches or dinners:<br>3 in-person TMG meetings, 52 total attendees<br>4 in person TSC meetings, 20 total attendees<br><br>Total = 72 lunches for TMG and TSC meeting participants	Meeting lunches or hotel dinners (vegetarian) = 2.6 $kgCO_2e$ per meal per person<br><br>Meeting lunches or hotel dinners (meat) = 5.92 $kgCO_2e$ per meal per person<br><br>Assumption: unable to determine if meat or vegetarian therefore assumed 50/50<br>(36 x 2.6) + (36 x 5.92) = 306.7 $kgCO_2e$	306.7 $kgCO_2e$
**4. Intervention*** **4.1. Physical (IMP)**<br>**4.2. Clinical (non-IMP)**<br>**4.3. Other (not captured above)**<br>**Please fill out the section most relevant to the intervention being investigated.**				
**4.1. Physical**				
4.1.1, 4.1.2. Movement of intervention, or materials required to deliver the intervention	E.g. movement of intervention from manufacturing site to distribution site, shipment of IMP to participating sites or direct to participant	Total weight of deliveries:<br><br>Total distance of deliveries:	Carry out freight calculation as described in section 1.2.<br><br>For refrigerated freight, increase the total $kgCO_2e$ associated with freight by 15%.<br><br>Frozen freight:	N/A – Avoidance of radiotherapy trial

* As per assumptions detailed in the guidance, manufacture of the intervention is considered out of scope. This section defines all processes relating to providing and delivering the trial intervention that are over and above routine care.

For peer review only - http://bmjopen.bmj.com/site/about/guidelines.xhtml

FUNDED BY NIHR | National Institute for Health and Care Research
LIVERPOOL CLINICAL TRIALS CENTRE LCTC
ICR

			Dry ice has a carbon footprint of **1.81kg CO_2e for 1 kg dry ice** produced/used.<br><br>When calculating the overall emissions of frozen freight, as well as the 1.81 kgCO_2e per 1kg attributed to manufacture, include the weight (kg) of dry ice used in the weight of the freight calculation in section 1.2.<br><br>In the absence of activity data, assume 1kg of dry ice is used per individual sample shipping box.	
4.1.3. Materials required for the packaging and shipment of IMP	E.g. cardboard, cold storage boxes, polystyrene	Number of cold storage boxes:<br><br>Kg of cardboard/ polystyrene:	Single use sample cold storage box = 25.2 kgCO_2e per box<br><br>Reusable sample cold storage box = 2.2 kgCO_2e per box<br><br>Kg (cardboard) x 0.821 = kgCO_2e<br>Kg (polystyrene) x 3.778 = kgCO_2e	N/A – avoidance of radiotherapy trial
4.1.4. Destruction of overage	E.g. incineration of IMP	Estimated weight of overage incinerated:	Kg of waste x 2.4252 = kgCO_2e	N/A – avoidance of radiotherapy trial
**4.2. Clinical** e.g., radiotherapy, device, surgical.<br>NB: not all calculations will be relevant to all interventions. **This section of the method will be further developed as we carbon footprint more trials, so please inform us if your protocol specifies an activity that has not been included, and we will help to determine the associated carbon footprint.**				
4.2.1 Movement of the intervention, or resources	E.g. movement of intervention from	Estimated total weight of delivery:	Please refer to section 1.2 and 4.1.1, 4.1.2.	N/A – avoidance of

For peer review only - http://bmjopen.bmj.com/site/about/guidelines.xhtml

FUNDED BY NIHR | National Institute for Health and Care Research
LIVERPOOL CLINICAL TRIALS CENTRE LCTC
ICR

required to deliver the intervention	manufacturing site to distribution site, shipment of intervention to participating sites.	Estimated total distance of deliveries:		radiotherapy trial
4.2.2 Materials required for the packaging and shipment of the intervention	E.g. cardboard, cold storage boxes, polystyrene	Number of cold storage boxes:<br><br>Kg of cardboard/ polystyrene:	Please refer to section 4.1.3.	N/A – avoidance of radiotherapy trial
4.2.3 Utilities required for delivery of the intervention	E.g. Hospital utilities if the intervention is delivered within a hospital	Hospital staff FTE required:	Please refer to section 7.3 to calculate the emissions attributed to hospital utilities if required to deliver the intervention.	N/A – avoidance of radiotherapy trial
4.2.4 Activities or resources required/relating to delivery of the intervention	E.g. consumables, surgical time, specialist equipment, incineration of surgical waste		Please refer to section 7.2 for consumables, surgery and other activities that may be relevant to the delivery of the intervention, but please take care to avoid double counting.<br><br>To calculate the emissions attributed to incineration, e.g. of surgical waste, please refer to section 4.1.4.	N/A – avoidance of radiotherapy trial
**4.3. Other**<br>NB: not all calculations will be relevant to all interventions. **This section of the method will be further developed as we carbon footprint more trials, so please inform us if your protocol specifies an activity that has not been included, and we will help to determine the associated carbon footprint.**				
4.3.1 Movement of the intervention to the participant or participating site	E.g. shipment of intervention to participating sites or direct to participant	Estimated weight of delivery:<br><br>Estimated distance of deliveries:	Please refer to section 1.2.	N/A – avoidance of radiotherapy trial
4.3.2 Materials required for packaging and	E.g. cardboard, cold storage boxes, polystyrene	Number of cold storage boxes:<br><br>Kg of cardboard/ polystyrene:	Please refer to section 4.1.3.	N/A – avoidance of

For peer review only - http://bmjopen.bmj.com/site/about/guidelines.xhtml

shipment of the intervention				radiotherapy trial
4.3.3 Materials or resources required for delivery of the intervention	E.g. software, booklets, specialist equipment		For printing and paper, please refer to section 1.1.	N/A – avoidance of radiotherapy trial
4.3.4 Travel required to facilitate delivery of the intervention	E.g. to deliver training, conduct interviews etc	Estimated distance travelled, and number of passengers (p.km):	Please refer to section 3.1, 3.2.	N/A – avoidance of radiotherapy trial
**5. Data collection and exchange**<br>NB: analysis of data does not need to be calculated separately, it is covered by the emissions attributed to trial staff FTE in "CTU emissions" and calculations included within "Data Collection and exchange".				
5.1. Data collection and query exchange between CTU and sites	E.g. CRFs, EDC completion and query resolution, scans copied to CDs	Postage from sites to ICR-CTSU:<br>Only the eligibility, pre-screening and registration checklists posted to CTU from sites.<br>-Total pages from all sites = 14094.<br>-Average site distance from ICR-CTSU: 301.8 km<br><br>Printing by sites for their SIF:<br>Estimate 419 pages printed by each site for their SIF, 63 sites = 26397 total pages<br><br>Paper CRFs:<br>28 folders/box files containing paper CRFs in ICR-CTSU.	Postage of documents from sites:<br>14094 x 0.005 = 70.47 kg / 0.07 tonnes<br>0.07 x 301.8 = 21.126 t.km<br>21.126 t.km x 0.19443 = 4.1 $kgCO_2e$<br><br>Printing by sites for their SIF:<br>26397 x 0.005 = 131.985 kg<br>Printing: 131.985 kg x 0.22438 = 29.6 $kgCO_2e$<br>Material: 131.985 kg x 0.919 = 121.3 $kgCO_2e$<br><br>Paper CRFs<br>14000 x 0.005 = 70kg<br>Printing: 70 kg x 0.22438 = 15.7 $kgCO_2e$<br>Materials: 70kg x 0.919 = 64.3 $kgCO_2e$<br><br>Site documents stored in ICR-CTSU:<br>6300 x 0.005= 31.5 kg<br>Printing: 31.5 kg x 0.22438 = 7.1 $kgCO_2e$	271.1 $kgCO_2e$

For peer review only - http://bmjopen.bmj.com/site/about/guidelines.xhtml

FUNDED BY NIHR | National Institute for Health and Care Research

		Assumption: box file contains 500 pieces of paper<br>500 x 28 = 14000 total pages<br><br>Site documents stored in ICR-CTSU:<br>18 folders with TMFs and site documents stored in CTU.<br>Assumption: folder holds 350 pages.<br>18 x 350 = 6300 total pages	Materials: 31.5 kg x 0.919 = 29 kgCO_2e	
5.2. Data sent direct from participants to CTU or participating sites	E.g. Questionnaires, patient diaries, wearables	Estimated weight and distance of delivery (t.km):<br><br>Device used and time taken to complete electronic questionnaires:	For paper questionnaires, please refer to section 1.1. for the carbon footprint of producing the materials and section 1.2. for postage (freight).<br><br>For use of smart watches and other devices see section 6.3.<br><br>**Electronic questionnaires**<br>Add the emissions attributed to data storage and transmission to the emissions attributed to using a device to complete the questionnaire.<br><br>Web surfing (data storage and transmission) = 9.441 g CO2e/hr (10 mins = 1.57 g CO_2e)<br><br>Choose from the below:<br>- Desktop computer = 0.18079 kg CO_2e per hour<br>- Laptop = 0.028719 kg CO_2e per hour	N/A

For peer review only - http://bmjopen.bmj.com/site/about/guidelines.xhtml

FUNDED BY NIHR | National Institute for Health and Care Research

			- Tablet = 0.027397 kg CO_2e per hour<br>- Smartphone = 0.015068 kg CO_2e per hour	
5.3. Data from labs to CTU<br>5.4. Data from other collaborators to CTU	E.g. Laboratory patient results, data linkage	GB required for data storage/transmission:<br>Duration of data storage:<br><br>Total £ spent on computer services: £49000	Data storage and transmission = estimate 1.365 kg CO_2e per GB per year.<br><br>For computer services such as data linkage:<br>£ x 0.149 = kgCO_2e<br><br>£49000 x 0.149 = 7301 kg$C0_2e$	7301 kg$C0_2e$
**6. Trial supplies and equipment**				
6.1. Equipment used by CTU	E.g. computers, laptops, printers, software	Total £ spent on office machinery and computers for trial: £4500	For any new office machinery and computers purchased specifically for trial:<br>£ x 0.387 = kgCO_2e<br>£4500 x 0.387 = 1741.5 kgCO_2e	1741.5 kgCO_2e
6.2. Equipment and supplies used by participating sites supplied by CTU	E.g. centrifuge, fridge, freezer	Estimated weight and distance of deliveries (t.km):	For the shipment of equipment to participating sites, please refer to section 1.2.<br><br>For the use of a centrifuge, please refer to section 9.2., for a fridge or freezer please refer to section 9.3.	N/A
6.3. Equipment and supplies provided to participants specifically for the trial	E.g. wearables, smartphone, tablet	Number of devices and duration of their usage:<br><br>Estimated weight and distance of deliveries (t.km):	**Smartphone =** 55 kgCO_2e from manufacture and add 5.5 kgCO_2e per year of usage.	N/A

For peer review only

For peer review only - http://bmjopen.bmj.com/site/about/guidelines.xhtml

FUNDED BY NIHR | National Institute for Health and Care Research

			**Tablet =** 119 $kgCO_2e$ from manufacture and add 10 $kgCO_2e$ per year of usage.<br><br>**Wearables/smart watch** = 30.1 kg CO_2e for manufacture and add 1.633 kg CO_2e per year of usage.<br><br>**To calculate the carbon footprint associated with shipment of the devices, please refer to section 1.2.**	
**7. Trial specific patient assessments**				
7.1. Patient travel for study visits that are in addition to standard of care	E.g. Eligibility and screening assessments, trial-specific assessments and procedures	Number of times patient is required to travel (in addition to standard of care): 1623 patients had to travel for 5 additional above standard of care mammograms	Emissions associated with one patient visit to hospital (UK) = 5.8 $kgCO_2e$ (this includes both the out and back journeys)<br>Emissions associated with one patient visit to GP surgery (UK) = 1.12 $kgCO_2e$ (this includes both the out and back journeys)<br><br>5.8 $kgCO_2e$ x 5 x 1623 = 47067 $kgCO_2e$	47067 $kgCO_2e$
7.2. Materials and activities required for study assessments that are in addition to standard of care	E.g. Laboratory tests, imaging assessments, clinical activities relating to intervention for example administering of study drug, biopsy.	5 above standard of care mammograms for each patient in very low risk and no RT group (1623)<br><br>Assumption: 2 visits where consumables required	Consumables = 0.30 $kgCO_2e$ per patient per trial appointment where consumables (such as gloves) required<br><br>- 1 MRI = 24.7 kg CO_2e<br>- 1 CT scan = 9.2 $kgCO_2e$<br>- 1 CXR = 0.8 $kgCO_2e$/scan<br>- 1 hour in surgery = 53 kg CO_2e	7977.3 $kgCO_2e$

For peer review only - http://bmjopen.bmj.com/site/about/guidelines.xhtml

FUNDED BY NIHR | National Institute for Health and Care Research

LIVERPOOL CLINICAL TRIALS CENTRE LCTC

ICR

			- 1 low intensity (general ward) bed day = 37.9 kg CO_2e<br>- 1 high intensity (ICU) bed day = 103 $kgCO_2e$<br>- 15 sessions of breast radiotherapy = 5.7 kg CO_2e<br>- 20 sessions of prostate radiotherapy = 15.3 kg CO_2e<br><br>Blood tests:<br>- 82 g CO_2e for coagulation profile<br>- 116 g CO_2e for full blood examination<br>- 49 g CO_2e for arterial gas assessment<br>- 99 g CO_2e for urea and electrolyte assessment<br>- 0.5 g CO_2e for C-reactive protein<br><br>**Please note that the above figures for blood tests include the materials and consumables required for sample collection, phlebotomy and analysis, as well as power consumption by pathology analysers.**<br><br><u>Calculations:</u><br>Consumables:<br>- 0.30 $kgCO_2e$ x 2989 (number of patients who received ki67 testing) = 896.7 $kgCO_2e$<br>- 0.30 x 1962 (for physical appointment for those registered to study) = 588.6 $kgCO_2e$	

For peer review only

FUNDED BY NIHR | National Institute for Health and Care Research

			- Total = 1485.3 $kgCO_2e$<br><br>Scans: 5 x 0.8 $kgCO_2e$ x 1623 = 6,492 $kgCO_2e$	
7.3. Utilities required for study assessments that are in addition to standard of care	E.g. energy consumption per square metre of hospital space according to trial staff FTE, taking into account time required for CRF completion and study assessments, consent etc	Hospital trial staff FTE: total estimated FTE = 5.5 from 2017 - 2022[†]	Electricity: 1 FTE 1 year = 364.9 $kgCO_2e$ Multiply by the number of years and FTE applicable<br><br>364.9 $kgCO_2e$ x 5.5 = 2007 $kgCO_2e$<br><br>Heating: 1 FTE, 1 year = 685.7 $kgCO_2e$ Multiply by the number of years and FTE applicable<br><br>685.7 $kgCO_2e$ x 5.5 = 3771.4 $kgCO_2e$	5778.4 $kgCO_2e$
**8. Samples**				
8.1. Materials involved	E.g. sample collection kit and packaging for shipment	Kg of material:<br>- 30.6 kg slide container (pp)<br>- 243.8 kg jiffy bag<br>- 39 kg paper	The emissions attributed to sample collection consumables for common blood tests are included in the blood tests listed in section 7.2.<br><br>To calculate the carbon footprint of other common materials, multiply the weight in kg by the relevant emission factor below to produce $kgCO_2e$.<br>- Average plastics: 3.116<br>- Plastics (average film): 2.754<br>- Plastics (Average rigid): 3.277<br>- Plastics (PP): 3.105	397.9 $kgCO_2e$

[†] Hospital trial staff FTE was estimated over 6 years rather than the 10-year trial duration because the final 4 years are follow up via routine data sources.

For peer review only - http://bmjopen.bmj.com/site/about/guidelines.xhtml

FUNDED BY NIHR | National Institute for Health and Care Research

			- Plastics (PET): 4.032<br>- Glass: 3.413<br>- Paper: 0.919<br>- Board: 0.821<br><br>Slide containers = 30.6 kg x 3.105 (pp) = 95 $kgCO_2e$<br><br>Paper (materials) = 39kg x 0.919 = 35.8 $kgCO_2e$<br>Paper (printing) = 39kg x 0.22438 = 8.8 $kgCO_2e$<br><br>Jiffy bag = 243.8 kg x 0.919= 224.1 $kgCO_2e$<br><br>Tracking slip: 2 copies of each tracking slip on top of one included in the kit– 2989 x 2 = 5978 pages<br>Printing: 5978 pages x 5g = 29890g/29.89kg<br>29.89 kg x 0.22438 = 6.7 $kgCO_2e$<br>26.9kg x 0.919 = 27.5 $kgCO_2e$	
8.2. Movement of sample kit materials from manufacturer to CTU<br>8.3. Movement of sample kits from CTU/distributor to participating Sites<br>8.4. Movement of samples from participating sites or	E.g. shipment of blood tubes for sample kits to CTU	8.2. Movement of sample kit materials from manufacturer to CTU<br>Envelopes:<br>- Weight = 0.004 tonnes, total<br>- Total distance transported = 849.6km (141.6km x 6 deliveries)<br><br>Slide mailing containers:<br>-Weight = 0.00204 tonnes	Please refer to section 1.2. for freight and 4.1. for refrigerated or frozen freight.<br><br>**8.2: Movement of sample kit materials from manufacturer to CTU**<br><br>Envelopes:<br>0.004 tonnes x 849.6km = 3.3984 t.km<br>3.3984 t.km x 0.19443 = 0.66 $kgCO_2e$	40.26 $kgCO_2e$

For peer review only - http://bmjopen.bmj.com/site/about/guidelines.xhtml

FUNDED BY NIHR | National Institute for Health and Care Research

patients to central laboratory.		-Total distance transported = 6910.3 km (363 km x 19 deliveries)<br><br>8.3. Movement of sample kits from CTU to participating Sites:<br>-Average distance from CTU to participating site = 301.8km<br>- Shipment weight = 0.00053 tonnes<br>- 630 sample kit shipments<br><br>8.4. Movement of samples from participating sites to laboratory.<br>-Cambridge lab – average distance to participating site = 239.5km, 938 shipments<br>-RMH lab - average distance to participating site = 258km, 1094 shipments<br>-Glasgow lab - average distance to participating site = 467.8km, 957 shipments	Slide mailing containers:<br>0.00204 tonnes x 6910.3 km = 14.097 t.km<br>14.097 t.km x 0.19443 = 2.7 kgCO_2e<br><br>**8.3. Movement of sample kits from CTU/distributor to participating Sites**<br><br>0.00053 x (630 x 295.88) = 100.77 t.km x 0.19443 = 19.6 kgCO_2e<br><br>**8.4. Movement of samples from participating sites or patients to central laboratory.**<br><br>Cambridge laboratory: 0.0000935 x (239.5km x 938) x 0.19443 = 4.1 kgCO_2e<br>RMH laboratory: 0.0000935 X (258 km X 1094) X 0.19443 = 5.1 kgCO_2e<br>Glasgow laboratory: 0.0000935 x (467.8km x 957) x 0.19443 = 8.1 kgCO_2e	
**9. Laboratory**				
9.1. Emissions attributed to lab utilities according to staff FTE	E.g. energy consumption per square metre of laboratory space	Trial staff FTE and trial duration: 3 labs, 1 staff member in each. Trial is 5% of each of their FTE for 4 years and 2% for 1 year.	**Electricity:**<br>1747.2 kgCO_2e per FTE per year<br>Multiply 1747.2 kgCO_2e by the number of years and FTE applicable	2061.4 kgCO_2e

For peer review only - http://bmjopen.bmj.com/site/about/guidelines.xhtml

FUNDED BY NIHR | National Institute for Health and Care Research

LIVERPOOL CLINICAL TRIALS CENTRE LCTC

ICR

	according to trial staff FTE		1747.2 $kgCO_2e$ x 0.05 = 87.36 $kgCO_2e$<br>87.36 $kgCO_2e$ x 4 years = 349.44 $kgCO_2e$<br>1747. 2 $kgCO_2e$ x 0.02 = 34.944 $kgCO_2e$<br>349.44 + 34.944 = 384.4 $kgCO_2e$<br>384.4 x 3 lab staff = 1153.2 $kgCO_2e$<br><br>**Heating:**<br>1376 $kgCO_2e$ per FTE per year<br>Multiply 1376 $kgCO_2e$ by the number of years and FTE applicable<br><br>1376 $kgCO_2e$ x 0.05 = 68.8 $kgCO_2e$<br>68.8 $kgCO_2e$ x 4 = 275.2 $kgCO_2e$<br>1376 x 0.02 $kgCO_2e$ = 27.52 $kgCO_2e$<br>275.2 $kgCO_2e$ + 27.52 $kgCO_2e$ = 302.72 $kgCO_2e$<br>Total = 302.72 $kgCO_2e$ x 3 = 908.16 $kgCO_2e$	
9.2. Materials/equipment /consumables used in processing and analysis of samples	E.g. centrifuges, refrigerators	kWh usage of equipment, hours used:<br><br>Kg of material:	To avoid double counting, use of equipment will be included in lab staff FTE if calculated.<br><br>If the trial does not involve a central lab, but there is still sample processing on site, please see below. For storage of samples, please see section 9.3.<br><br>To calculate the emissions of a piece of equipment, multiply the power consumption by hours used to get a kWh value. Finally multiply kWh by the electricity emission factor (0.273).	N/A

For peer review only

			Example: use of a 310-Watt centrifuge for 15 minutes<br>- 310 Watts x 0.25 (hours) = 77.5 kWh<br>- 77.5 kWh x 0.273 = 21.2 $kgco_2e$<br><br>Consider the centrifuge capacity and multiply by the number of uses required.	
9.3. Storage of samples	E.g. utilities and ultra-low temperature freezer	Length of storage:<br><br>Amount of refrigerator/freezer space required:	Storage in fridge/-20 freezer:<br>- 298.9 $kgCO_2e$ per year<br>- Multiply by number of years stored<br><br>Storage in an ultra-low/-80 freezer:<br>- 2192.2 $kgCO_2e$ per year<br>- Multiply by number of years stored<br><br>NB: this is for a whole freezer; you will need to make an assumption about the amount of space in the freezer that the trial samples take up.	N/A
**10. Trial close out**				
10.1. Storage and archiving of essential trial documentation and data	E.g. Hospital files, lab files, trial guidance documents etc	Duration of storage: 10 years<br><br>Amount of space required for storage (m^2): $4m^2$<br><br>Storage location: CTU (office)	Carbon footprint associated with $1m^2$ for 1 year:<br>- Office: 16.1 $kgC0_2e$<br>- Laboratory: 43.7 $kgc0_2e$<br>- Warehouse: 7.4 $kgC0_2e$<br>- Health building: 22.1 $kgCO_2e$<br><br>Choose the most suitable building type and multiply by number of years and m^2 necessary.<br><br>**Heating**	2126.6 $kgCO_2e$

For peer review only - http://bmjopen.bmj.com/site/about/guidelines.xhtml

FUNDED BY NIHR | National Institute for Health and Care Research | LIVERPOOL CLINICAL TRIALS CENTRE LCTC | ICR

			Carbon footprint associated with 1 m^2 for 1 year:<br><br>- Office: 36 $kgCO_2e$<br>- Laboratory: 34.4 $kgCO_2e$<br>- Warehouse: 13.3 $kgCO_2e$<br>- Health building: 41.6 $kgCO_2e$<br><br>Choose the most suitable building type and multiply by number of years and m^2 necessary.<br><br>**For electronic storage,** estimate 1.365 kg CO_2e per GB per year.<br><br>Calculations:<br>Electricity: 16.1 $kgCO_2e$ x 4 x 10 = 644 $kgCO_2e$<br><br>Heating: 36 $kgCO_2e$ x 4 x 10 years = 1428 $kgCO_2e$<br><br>Electronic storage:<br>PRIMETIME trial database = ~ 4GB<br>Assumption: storage for 10 years<br>1.365 kg CO_2e x 4 x 10 = 54.6 $kgCO_2e$	
10.2. Storage and destruction of biological samples	E.g. blood, tissue, urine etc	Duration of storage: 10 years<br><br>Amount of space required for storage (m^2): 2m^2<br><br>Storage location: Health building	See section 9.3. for storage of refrigerated or frozen samples.<br><br>See section 10.1 for storage of ambient samples.	1274 $kgCO_2e$

For peer review only - http://bmjopen.bmj.com/site/about/guidelines.xhtml

			Electricity:<br>22.1 $kgCO_2e$ x2 x 10 = 442 $kgCO_2e$<br><br>Heating:<br>41.6 $kgCO_2e$ x 2 x 10 = 832 $kgCO_2e$	
10.3. Return of equipment and supplies from participating sites to CTU	E.g. wearables, unused or expired equipment and supplies	Estimated weight and distance of delivery (t.km):	See section 1.2. (freight).	N/A

PRIMETIME carbon footprint summary

Module	$KgCO_2e$
Trial set up	454.2
CTU emissions	11733.3
Trial staff meetings and travel	1136.2
Treatment intervention	0
Data collection and exchange	7572.1
Trial supplies and equipment	1741.5
Trial specific patient assessments	60822.7
Samples	438.2
Laboratory	2061.4

For peer review only - http://bmjopen.bmj.com/site/about/guidelines.xhtml

Analysis and trial close out	3400.6
**Total =**	89360.2 kgCO_2e/ 89.4 tonnes

References

[1] What is an emission factor? [Internet]. Climfoot-project.eu. [cited 2023 May 11]. Available from: https://climfoot-project.eu/en/what-emission-factor

For all emission factor and benchmark data sources, please refer to the accompanying "Detailed Guidance and method to calculate the carbon footprint of a clinical trial."

For peer review only

For peer review only - http://bmjopen.bmj.com/site/about/guidelines.xhtml
